# Association of key species of vaginal bacteria of recurrent bacterial vaginosis patients before and after oral metronidazole therapy with short- and long-term clinical outcomes

**DOI:** 10.1371/journal.pone.0272012

**Published:** 2022-07-28

**Authors:** Ashomathi Mollin, Mounika Katta, Jack D. Sobel, Robert A. Akins

**Affiliations:** 1 Department of Biochemistry, Microbiology, & Immunology, Wayne State University School of Medicine, Detroit, Michigan, United States of America; 2 Division of Infectious Diseases, Wayne State University School of Medicine, Detroit, Michigan, United States of America; University of Minnesota Twin Cities, UNITED STATES

## Abstract

Bacterial vaginosis (BV) is associated with a state of vaginal dysbiosis typically involving depletion of otherwise dominant populations of *Lactobacillus*. The causes of this microbial succession are not known; there may be multiple causes. Standard treatment includes oral metronidazole, which typically restores *Lactobacillus* species to dominance. However, recurrence rates are high; recurrent BV patients recur 3–4 times annually and are often refractory to treatment. Our previous qPCR-based study of recurrent BV patients pointed to putatively more virulent species of *Gardnerella* that were associated with refractory responses to oral metronidazole, and less robust recovery of *Lactobacillus* species associated with recurrence after an initial period of remission. However, these associations did not account for outcomes in all patients, suggesting that other bacterial species were involved. In this follow-up study, we sequenced the V4 domain of 16S rRNA sequences of 41of these same patients pre- and posttreatment. Overall compositions among pretreatment clinical outcome groups were not different, although alpha diversity significantly decreased: refractory > recurrent > remission. Combinations of key species were associated with and prognostic for outcome. Higher pretreatment abundance of *Megasphaera lornae* together with lower abundance of *Gardnerella Gsp07* and *Finegoldia magna* predicted long term remission after oral metronidazole. Furthermore, a subset of refractory patients that did not have high levels of *Gardnerella Gsp07*, instead had elevated levels of alternative species including *Atopobium vaginae*, *Mageeibacillus indolicus (BVAB3)*, and *Prevotella timonensis*. Patients who recurred after transient remission had elevated abundance of species including *Atopobium vaginae*, *Gardnerella*, and *Aerococcus christensenii*, compared to long-term remission patients. Core bacterial species among refractory patients did not change in abundance after metronidazole, suggesting resistance or tolerance, in contrast to the loss in abundance of the same species among recurrent or remission patients. These findings have potential prognostic and therapeutic implications.

## Introduction

Bacterial vaginosis (BV) is a state of dysbiosis of vaginal microbiota in which typically dominant species of *Lactobacillus* are displaced by an array of BV-associated species [1, 2]. There is no known cause for BV or for the displacement of lactobacilli, although some believe that some species of *Gardnerella* play a perhaps necessary but not sufficient role in initiating the succession [3–5]. There is some uncertainty because subpopulations of healthy (asymptomatic) women host non-*Lactobacillus* dominant vaginal microbiota [6–8], and at least some among these do not seem to be in transition to or from symptomatic BV [9]. The issue is further complicated in that recurrent BV, defined as 3 or more episodes per year, may have different causes than sporadic or incident BV [10, 11]. Nevertheless, women who achieve remission from BV upon treatment, typically with oral metronidazole, almost always concomitantly acquire a *Lactobacillus*-dominant vaginal microbiome [12–16].

For decades, *Gardnerella vaginalis* was the only species classified under the genus *Gardnerella*. As this species was found in 87% of healthy women and therefore did not satisfy Koch’s postulates as the cause of BV, heterogeneity among the species was suspected [17]. Numerous cultivation–dependent and cultivation—independent studies have established that *G*. *vaginalis* is genetically and phenotypically heterogeneous [18–36]. Polymorphisms in its cpn60 gene cluster *G*. *vaginalis* strains into two groups (A & B) and corresponding clades (Clades 1–4) [23, 24, 37]. More recently, whole genome sequencing and next generation sequencing studies have identified 9–13 distinct genomospecies under the genus *Gardnerella* [25, 26]. Associations of individual *Gardnerella* clades or species with BV pathogenesis have been inconsistent across different studies. However, many have observed that the presence of multiple clades is associated with BV [15, 24, 28, 38].

Metagenomic studies in parallel with diagnostic tools have shown that while healthy and asymptomatic Caucasian and Asian women tend to have vaginal compositions that are *Lactobacillus* dominant, a subset of healthy, asymptomatic African American and Hispanic women tend to have vaginal compositions that resemble BV. Together, these studies have also indicated that a given microbial profile is not a definitive indicator for BV; species composition is not enough to distinguish BV-positive from BV-negative patients, even as defined by Amsel criteria [6, 8, 9, 39–41]. Anaerobic species present in high prevalence in BV positive women can also be present at lower prevalence in healthy women. Furthermore, species that are detected at low prevalence in healthy women are poor markers for BV diagnosis or prognosis because they are not sufficiently prevalent in symptomatic BV patients [41]. Finally, changes by orders of magnitude occur daily in vaginal bacterial compositions [42–46]. These are some of the factors that complicate the identification of bacterial species that are diagnostic of BV or prognostic of recurrence.

Individual studies vary as to what bacteria are considered important in the diagnosis or prognosis of BV. Broadly, NGS and qPCR studies have proposed that species under the genera *Atopobium*, *Dialister*, *Gardnerella*, *Lactobacillus* (*L*. *iners*), and *Prevotella* are be associated with incidental BV; *Corynebacterium*, *Lachnospiraceae_1(BVAB1)*, and *Ruminococcaceae_2(BVAB2)* are associated with recurrent BV; and *Megasphaera lornae* is associated with both incidental and persistent BV [8, 41, 47–55]. These variations may be attributed to different demographic factors such as geographical location, incidence of sex, ethnicity, and personal hygiene, but they also suggest that there may be many paths to BV. Commercial BV qPCR tests are available and have varying targets. For example, LabCorp offers a research-lab semi-quantitative test for *Atopobium vaginae*, *Ruminococcaceae_2(BVAB2)*, and *Megasphaera lornae*, Diagnostic Laboratory of Oklahoma tests for *Atopbium vaginae*, *Gardnerella vaginalis*, *Mycoplasma genitalium*, *Mycoplasma hominis*, and *Ureaplasma urealyticum*, and the BD Max system Vaginal Panel is a qPCR test fo*r Lactobacillus spp*. *(L*. *crispatus* and *L*. *jensenii)*, *Gardnerella vaginalis*, *Atopobium vaginae*, *Ruminococcaceae_2(BVAB2)*, *and Megasphaera lornae*.

The most widely used therapy for BV in the United States is oral or vaginal metronidazole, followed by oral clindamycin. Yet, the recurrence rate after metronidazole is as high as 80 percent [10, 56–58]. Several factors may contribute to this. One may be reinfection, although this may be more relevant in incident BV rather than recurrent BV [57, 59–62]. Recurrence may occur due to metronidazole resistance; several species or strains within species are resistant in vitro [11, 49, 57, 63–66]. Another recurrence mechanism may be inactivation or sequestration of metronidazole by one or several species [67] or by sequestering bacterial populations within biofilms, which are likely to have initiated with *Gardnerella* species [27, 28, 35, 36, 68, 69]. A cultivation–dependent study proposed that the clinical response post–metronidazole treatment is highly dependent on the pretreatment abundance of *Lactobacillus spp*. versus BV–associated species, particularly *Gardnerella*. Employing the concept of bacterial competition, this study suggested that elevated pretreatment abundance of *Lactobacillus spp*. relative to BV–associated species lowered the efficacy of metronidazole, and patients who had higher pretreatment abundance of BV–associated species relative to *Lactobacillus spp*. experienced a boosted metronidazole efficacy leading to a favorable outcome [67].

This study focuses on recurrent BV patients as an extension of our study initially reported in 2019 in which we saw three basic responses to treatment with oral metronidazole: refractory, recurrent, and remission [15, 70]. This qPCR-based study found that *Gardnerella Gsp07* and *G*. *swidsinskii* / *G*. *leopoldii* were strongly associated with refractory and recurrent responses, and a more robust recovery of *Lactobacillus* species, most often *L*. *iners*, was associated with remission patients compared to recurrent patients, even though both groups were in remission at the posttreatment visit [70]. The current study examined a subset of the initial patient samples by next-generation sequencing of the V4 domain of the 16S ribosomal RNA to provide a deeper understanding of changes to the vaginal microbiome before and after oral metronidazole. Our hypothesis is that there are key species differences in the pretreatment vaginal microbiota of remission patients, in addition to lower abundance of the virulent *Gardnerella* species, that are associated with a more enduring remission, and that key species differences at the posttreatment visit are prognostic of remission versus recurrent outcomes.

## Materials and methods

### Patient cohort

This study employed a portion of the patient cohort involved in prior studies conducted by the laboratories of Dr. Robert Akins and Dr. Jack Sobel at the School of Medicine and the Vaginitis Clinic at Wayne State University [70, 71]. This study used de-identified samples from women who had signed written, informed consent after discussion and witnessing by the research nurse. This and post-study data analysis using these samples were approved by the WSU IRB board, approval number 040314M1F. The following requirements were fulfilled when enrolling recurrent BV patients: three or more episodes of symptomatic BV in the preceding year, presence of acute symptoms of BV at enrollment, minimum age of 18, premenopausal, absence of mixed vaginal infections, withholding of vaginal products during the course of the trial, heterosexuality, use of condoms during sexual intercourse during the trial period, disclosure of any unprotected sexual activity, voluntary sexual refrainment 48 hours prior to any visit, and alcohol abstinence. A patient was further considered a BV–positive if they tested positive for at least three of the four Amsel criteria [72], and if they presented with symptoms such as malodor, vaginal discharge, and vaginal itching. (S1 Table in S1 File). Other exclusions are described in the original studies. The standard of care (SOC) treatment called for oral metronidazole therapy, twice a day, over a span of 7 days. These patients then returned for clinical assessment approximately 7–14 days post treatment for their second visit, and then once every month for up to 9 months. Along with these monthly visits, patients were required to perform daily vaginal swabs and communicate any engagement in sexual intercourse, use of sexual protection, and time of menses [70].

Patients with persistent BV symptoms and who tested positive for three out of four Amsel criteria at the second visit were classified as refractory BV patients. Patients who showed no BV symptoms at the second visit but later returned with BV symptoms and a positive Amsel criteria score were recognized as recurrent BV patients. Lastly, those who neither showed BV symptoms nor satisfied the Amsel criteria and were able to maintain this state for at least three months, were considered to have reached long-term remission.

**Fig 1:**
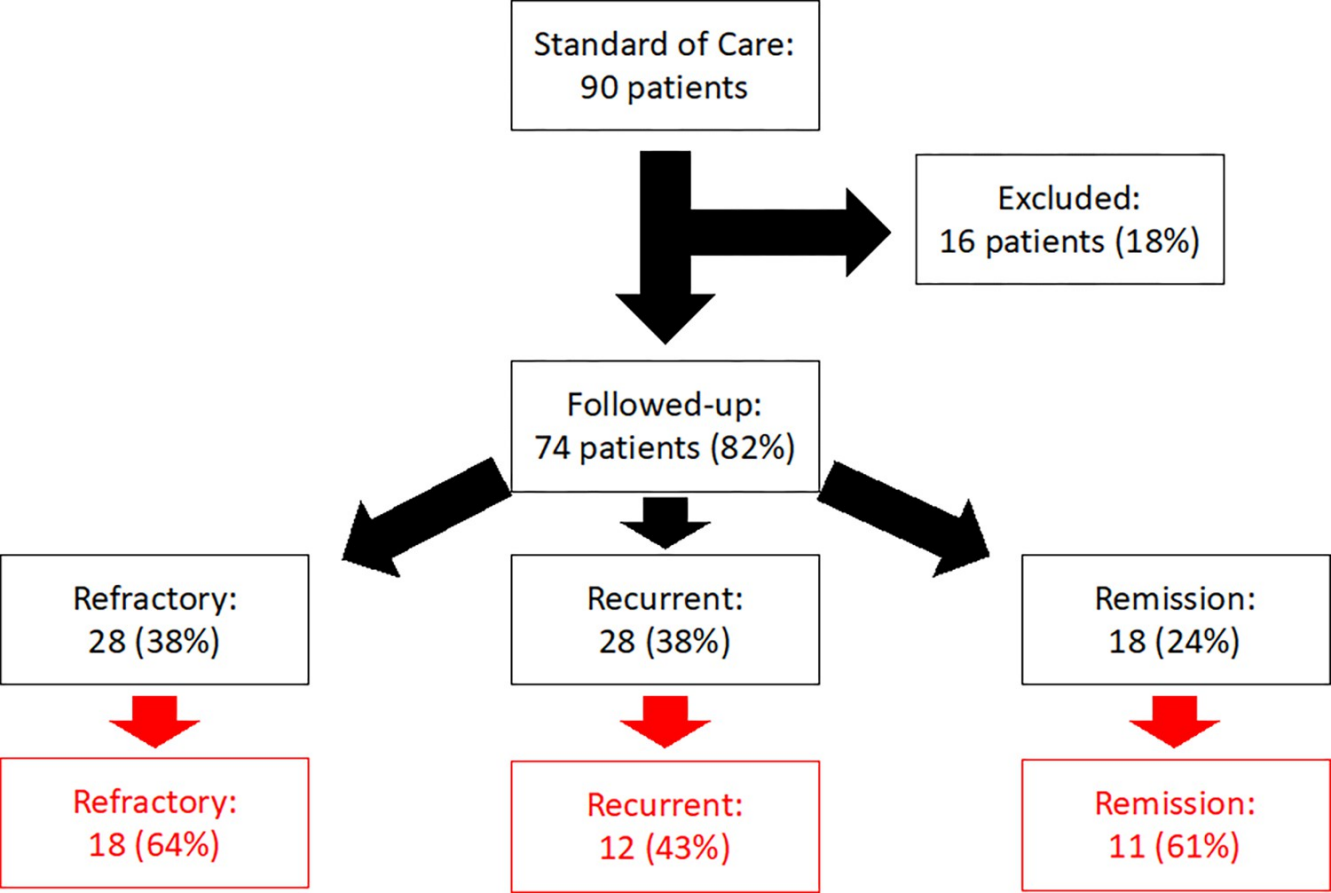
Patient enrollment flow diagram. This study sequenced 41 of 74 patients (represented by the red boxes) from the Akins-Sobel study [71]. Refractory patients returned to the second visit with BV symptoms and tested positive for 3 out of the 4 Amsel criteria. Recurrent patients briefly achieved remission, at the second visit, prior to recurrence. Remission patients reached clinical remission, at visit two, and were able to maintain this state for at least 3 months.

Our initial study utilized 43 (18 refractory, 16 recurrent, and 9 remission) of the 74 patients from the Akins-Sobel study to assess the patient clinical outcomes during days 1–14 (7 days of treatment and 7 days posttreatment) in terms of *Gardnerella* and *Lactobacillus* species relative abundance by qPCR [70]. This study will be making use of 41 patients (18 refractory, 12 recurrent, and 11 remission) to assess the vaginal microbiome relative to patient clinical outcomes before and after treatment (Fig 1). Limited resources prevented us from using all available patient samples for these analyses.

### DNA sequencing

DNA from vaginal swabs was prepared as described [15]. Next-generation sequencing of the 16S ribosomal RNA gene, V4 region. Sequencing was performed at the Michigan State University Research Technology Support Facility Genomics Core (Lansing MI). The V4 hypervariable region of the 16S rRNA gene was amplified using dual indexed Illumina compatible primers 515f/806r as described [73]. PCR products were normalized using Invitrogen SequalPrep DNA Normalization plates and the products recovered from the plates were pooled. This pool was size-selected with AMPureXP magnetic SPRI beads and tested for quality and quantity using a combination of Qubit dsDNA HS, Caliper LabChipGX HS DNA and Kapa Illumina Library Quantification qPCR assays. Sequencing of the pooled amplicons was performed on an Illumina MiSeq v2 standard flow cell using a 500 cycle v2 reagent cartridge. Custom Sequencing and index primers were added to appropriate wells of the reagent cartridge as described [73]. Base calling was done by Illumina Real Time Analysis (RTA) v1.18.54 and output of RTA was demultiplexed and converted to FastQ format with Illumina Bcl2fastq v2.18.0. There was an average of 34,154 SD 13,754 paired-end reads per sample with QC scores >30, typically 251–253 bp per trimmed sequence.

The processing of these sequences from FASTQ files was performed with Mothur software V1.35.0 [74, 75] following the default protocol for the MiSeq platform, including pairing, trimming of ends, exclusion of low-quality reads, removal of chimeras, de novo clustering into operational taxonomic units (OTU’s) at a 3% variation threshold, and identification of a representative sequences of each OTU at the midpoint of polymorphemic sequences within the OTU. 5011 OTU’s were assigned to genus or species levels in most cases, using the *assignTaxonomy* function from the DADA2 package within the R programming language [76] using the reference Silva training set file, “silva_nr99_v138.1_wSpecies_train_set.fa.gz” [77, 78], downloaded from the Github website [79] classified 2673 OTUs (out of 5011) to the species level.

To assign the best possible classification to the remaining OTUs, they were grouped and aligned by phylum to the 16S rRNA database in the Ribosomal Database Project (RDP) [80]. These sequences, along with the public representative isolates from the RDP, were then incorporated into phylogenic trees using the Maximum Likelihood (ML) algorithm, bootstrap phylogenetic method, and the Tamura-Nei model within the MEGA X software [81, 82]. A total of 16 different phylogenic trees were made–one for each phylum assessed (S1 Fig). Once these trees were made, the unassigned OTUs were renamed according to their location on the phylogenic tree relative to nearby cultured representative isolates. A summary table of all OTU sequences and taxonomic labels, related sequences in the databases, and a phylogenetic tree that positions all included reads and best hits are provided as S2 Table in S1 File and S2 Fig. Representative reads that were not assigned to species level were assigned a species or lowest possible taxonomic label if the RDP alignment was >97% to a reference species. In instances where multiple related species shared the same V4 sequence, or where there was heterogeneity within a phylogenetic branch, a representative species was used along with an asterisk symbol (*); species included by these * symbols are provided in S3 Table in S1 File. Phylogenies that were less characterized, e.g. Acidobacteria, were grouped by the RDP Classifier tool [83] with a confidence threshold marker of 60%. OTUs that could be assigned to the species level were classified to the lowest higher taxonomy level and labeled with a number (Taxa_#) to indicate that it belonged to a distinct phylogenetic branch with that level. The numbers were assigned at the end of the taxa to indicate separate groups of the higher order. Once renaming of the unassigned OTUs to the lowest taxonomy level was completed, these RDP Seqmatch results were compared to the classifications done by the *assignTaxonomy* algorithm. For those names, where both naming algorithms disagreed, a decision was made based on the RDP Classifier tool and the NCBI MOLE-BLAST tool. The highest common taxonomy level was retained if a decision using the different tool could not be made.

Together, the newly formed taxonomy table, NGS counts table, and patient metadata table associated with the pretreatment and posttreatment samples were loaded into MicrobiomeAnalyst [84, 85], MetaboAnalyst 5.0 [86, 87], and SPSS Statistics (Version 27–28, Armonk, NY: IBM Corp.). Within Microbiome Analyst, data was uploaded in two ways. In the first, the OTU counts data were filtered to condense OTUs identified as a single species into a representative OTU, yielding 865 OTUs (S4 Table in S1 File). These data, after filtering out OTU’s with low abundance, prevalence, and variance in Microbiomeanalyst, yielded 96 species that were the basis for comparative analysis (S5 Table in S1 File). We also input all 5011 OTU counts with a taxonomic classification scheme that filled all levels with the best identification, e.g. if an OTU was identified only to Genus, the species name given was Genus_sp. These OTUs were subjected within Microbiomeanalyst to a stricter filter: minimum 4 reads per OTU, 20% minimum prevalence, and 10% minimum variance among samples, leaving 51 OTUs (S6 Table in S1 File). We used unfiltered data from the 5011 OTU input file for alpha diversity analysis; all other analysis used the 865 OTU input dataset. Filtered raw counts were then normalized, using Total Sum Scaling (TSS), within the software. TSS normalizes the raw counts by dividing individual counts with the maximum sum of all counts and then multiply it with ten million for convenience. Data input into MetaboAnalyst 5.0 was generated in Microbiome Analyst as normalized abundance data, which we rescaled to the mean number of reads of all samples (21724), and log10 transformed. Both analytical software packages generate multiple analytical models from which it was determined whether overall bacterial compositions of the patient samples, or key species, at either visit, were associated with clinical outcome.

#### Statistics

Species that held statistical significance (p–value <0.05 and FDR < 0.1) were flagged by the different analytical tools such as classical univariate analysis, sparse high–throughput sequencing analysis, differential abundance analysis, and linear discriminate analysis effect size, and random forest in MicrobiomeAnalyst as potential diagnostic and prognostic markers. Using these flagged species, univariate and multivariate receiver operating characteristic (ROC) analyses were conducted in MetaboAnalyst for each outcome comparison. Species were removed multivariate ROC analysis, one at a time, eliminated if the exclusion did not result in a lower area under the curve (AUC) value, but replaced if it did lower the AUC value. This process was repeated until a maximum AUC value was achieved. This process identified the smallest combination of species that provided the largest significant AUC values. ROC–derived thresholds, sometimes adjusted manually, were determined for each species contributing to the optimized multivariant ROC analysis. Patient samples that were above or below these threshold values were summed and analyzed by the Fisher’s exact tests, conducted in Graph Pad Prism, which determined prognostic values (version 6.07 for Windows, GraphPad Software, La Jolla California USA, www.graphpad.com).

## Results

### Differences in alpha diversity associated with clinical outcome in both pre- and posttreatment samples

We detected 329 species among all 39 pretreatment patient samples, averaging 57 SD 18 species per sample. Among all 38 posttreatment patients we detected 298 species, for an average of 49 SD 17 species per sample.

**Fig 2:**
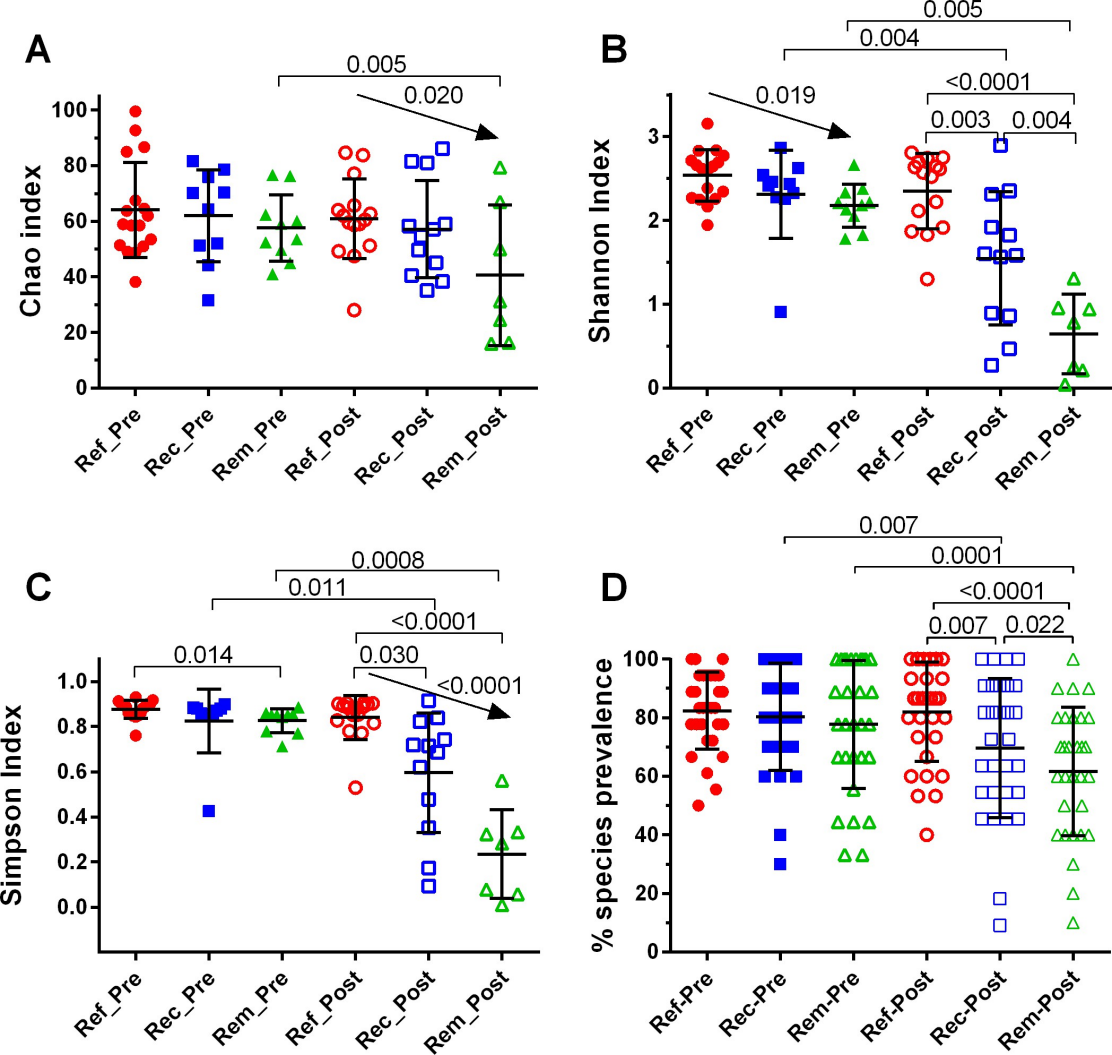
Alpha diversity indices among clinical outcome groups pre- and post-treatment. Index values for Chao, Shannon, and Simpson were determined using Microbiomeanalyst.ca with the unfiltered 5011 OTU dataset and Total Sum Scaling (TSS). % species prevalence was calculated based on the percent of species-positive samples versus total samples within each group and treatment, including only the 30 most prevalent species. Scatterplots showing mean and standard deviation, and statistical analyses were performed in GraphPad Prism; only significant p values or trends are shown. Between outcome comparisons of normally distributed Chao indices were analyzed by ANOVA with Holm-Sidak’s multiple comparisons test; non-normal Shannon and Simpson indices were analyzed by Kruskal-Wallis tests, with Dunn’s multiple comparisons tests. Pretreatment versus posttreatment comparisons were performed for Chao and % species prevalence by paired t-tests and for Shannon and Simpson by Wilcoxon matched-pairs signed rank tests (GraphPad Prism).

Species richness, the estimated median number of species by the Chao index (Fig 2A) ranged from 31–68 species. The Chao index distributions were not significantly different between outcome groups, but the downward trend among posttreatment samples, refractory>recurrent>remission, was significant. There was a significant posttreatment decrease in the Chao index only among remission patients. The Shannon index comparisons among pretreatment patients, while not significantly different by ANOVA, showed a significant downward trend, refractory > recurrent > remission (Fig 2B). This decrease was more pronounced and achieved significance at the posttreatment visit. Shannon indices decreased significantly from pre- to posttreatment for both recurrent and remission patient samples, but not for refractory patient samples. Comparisons with the Simpson index generated conclusions like those from the Shannon index (Fig 2C). Another metric of richness, the % prevalence of the top 30 most prevalent species, was also like comparisons using the Shannon index, except that pretreatment outcome groups were not differentiated (Fig 2D)

Differences in diversity metrics were sufficiently pronounced to have moderate prognostic value. Pretreatment patients above a ROC-based Shannon index threshold of 2.23 with an AUC of 0.815 (Metaboanalyst.ca), that is, those with elevated diversity and evenness, were predicted to have a refractory or recurrent outcome with 84% accuracy (95% CI 68% to 94%, Fisher’s exact test, MedCalc software). Recurrent or remission posttreatment patient samples above a ROC-based Shannon index threshold of 1.49 were predicted to have a recurrent rather than a remission outcome with an accuracy of 76% (95% CI 53% to 92%). That is, those with elevated diversity and evenness, were predicted to have a refractory or recurrent outcome with 84% accuracy, 95% CI 68% to 94%, by Fisher’s exact test [88]. Recurrent or remission posttreatment patient samples above a ROC-based Shannon index threshold of 1.43 (AUC = 0.845, Metaboanalyst.ca) were predicted to have a recurrent rather than a remission outcome with an accuracy of 79%, 95% CI 54% to 94 [88] (S7 Table in S2 File).

### Overall differences in composition (beta diversity) were only evident between posttreatment outcome groups

**Fig 3:**
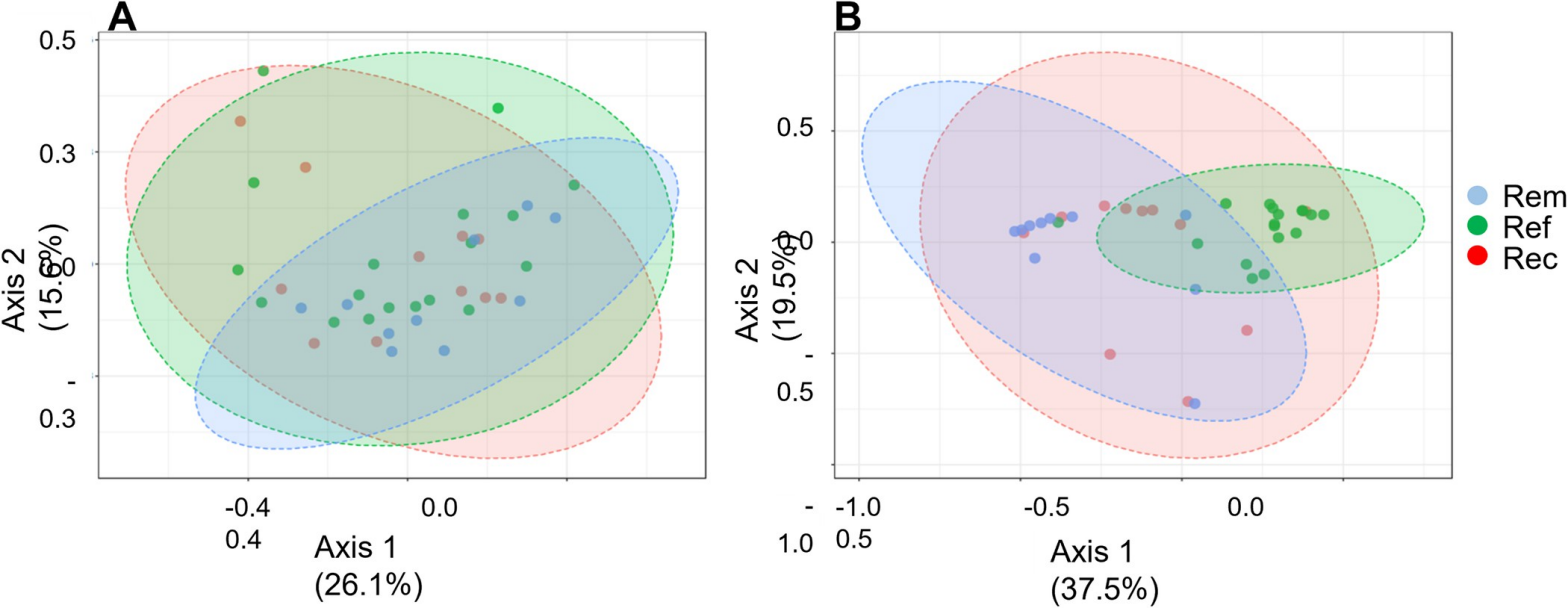
Grouping by overall compositions of patients by PCoA. PCoA analyses were performed separately for pretreatment (A.) and posttreatment (B.) using the MicrobiomeAnalyst.ca platform, inputting raw counts, normalizing with Total Sum Scaling (TSS) without further transformation, and labeling samples after plotting by clinical outcome group. Sample distance was measured using the non-phylogenic Bray-Curtis distance method. The shaded zones represent the range of two-component values with the 95% confidence intervals of observed data for each outcome group. Statistical significance was measured using Permutational ANOVA (PERMANOVA). (A) F-value: 0.96757;R-squared: 0.051012; p-value < 0.504 (top left). (B) F-value: 5.6556;R-squared: 0.24424; p-value < 0.001 (top right).

Principal coordinate analysis (PCoA) of pretreatment samples did not separate patients into distinct groups consistent with clinical outcomes (Fig 3A). By PCoA, only 6 of 39 samples from refractory and recurrent patient samples were placed outside of a shared single cluster (95% confidence interval zone) that included the remaining 33 patient samples from all outcome groups. In contrast, most posttreatment refractory and remission samples were readily separable into two distinct groups by PCoA (Fig 3B). Samples from recurrent patients were split, approximately half clustered with refractory and half with remission samples.

**Fig 4:**
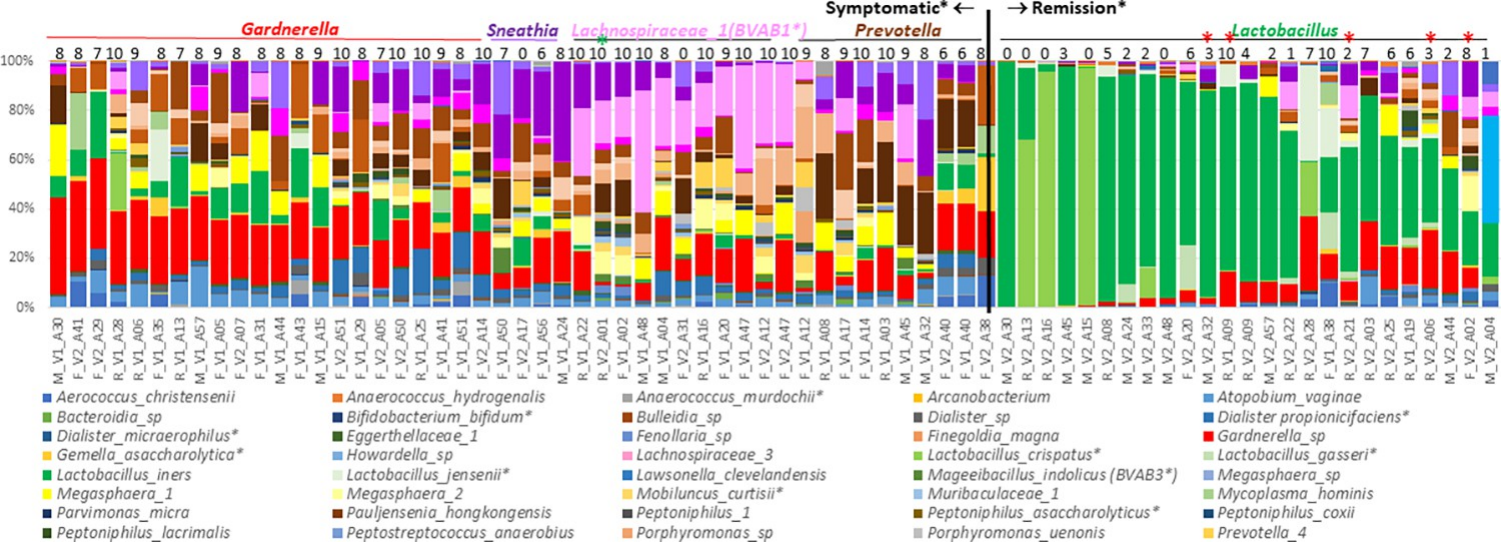
Manual clustering of patient samples by their most abundant genus (MAG). Relative abundances of genera were plotted as a stacked barchart in Excel and sorted by the 5 most MAG’s, color coded as shades of their title fonts. Within each group, samples were sorted by relative abundance of the MAG. Nugent scores are displayed above each sample. *: exceptional sample, Amsel-negative but non-*Lactobacillus* MAG sample. *: exceptional samples, symptomatic *Lactobacillus* MAG samples. Label codes: F = refractory patient, V1 = pretreatment sample, V2 = posttreatment sample, A## = patient identifier.

In an alternative approach to investigate diversity among outcome groups, we manually classified patients by their most abundant genus (MAG; Fig 4). This process identified 5 MAG groups. *Gardnerella*, *Prevotella*, *Shuttleworthia* (including *Lachnospiraceae_1(BVAB1)*, and *Sneathia* groups were almost exclusively from symptomatic patients, but the 4 groups were not associated with clinical outcome. In contrast, the fifth group, *Lactobacillus*, was composed mostly of samples from asymptomatic posttreatment patients. Within this group, recurrent patients were typically those in the lower range of % *Lactobacillus* compared to remission patient samples. However, there was no association of incidence of clinical outcome with pretreatment MAGs (chi sq p = 0.876). This association at posttreatment was significant (chi sq p = 0.002) and this was driven by the high incidence of Amsel-negative recurrent and remission patients in the *Lactobacillus* MAG: 9 of 10 posttreatment recurrent patients, and 10 of 10 posttreatment remission patients, were in the *Lactobacillus* MAG group. We observed no significant differences of Nugent scores between the MAG categories per outcome groups at pre- or post-treatment (Kruskal-Wallis, Dunn’s multiple comparisons tests). Mean Nugent scores of post-treatment Lactobacillus MAG groups varied (refractory 7 SD 1.4; recurrent 3.5 SD 2.7; remission 1.5 SD 1.2) but these differences were not significant due to intragroup variability and low numbers of refractory patients.

Collectively, these data indicate that overall compositions of symptomatic recurrent BV patients at pretreatment were not predictive of clinical outcome.

### Partial least squares discriminant analysis (PLSDA) identifies candidate biomarkers of treatment outcome

We removed species whose variation was not associated with clinical outcome to focus on a short list of species that were associated by performing reiterative PLSDA analysis. For pretreatment samples, supervised classification modeling approaches, such as partial least squares discriminant analysis (PLSDA) or sparse PLSDA, did not differentiate samples by outcome group, as they had misclassification rates greater than 59%, indicating poor cross-validation (S8a Table in S3 File). While reiterative linear discriminate modelling by sPLSDA (repeating the analysis using the most differentiating species from the initial analysis) yielded some improvements, classification error rates were still > 41%) (S8b Table in S3 File).

**Fig 5:**
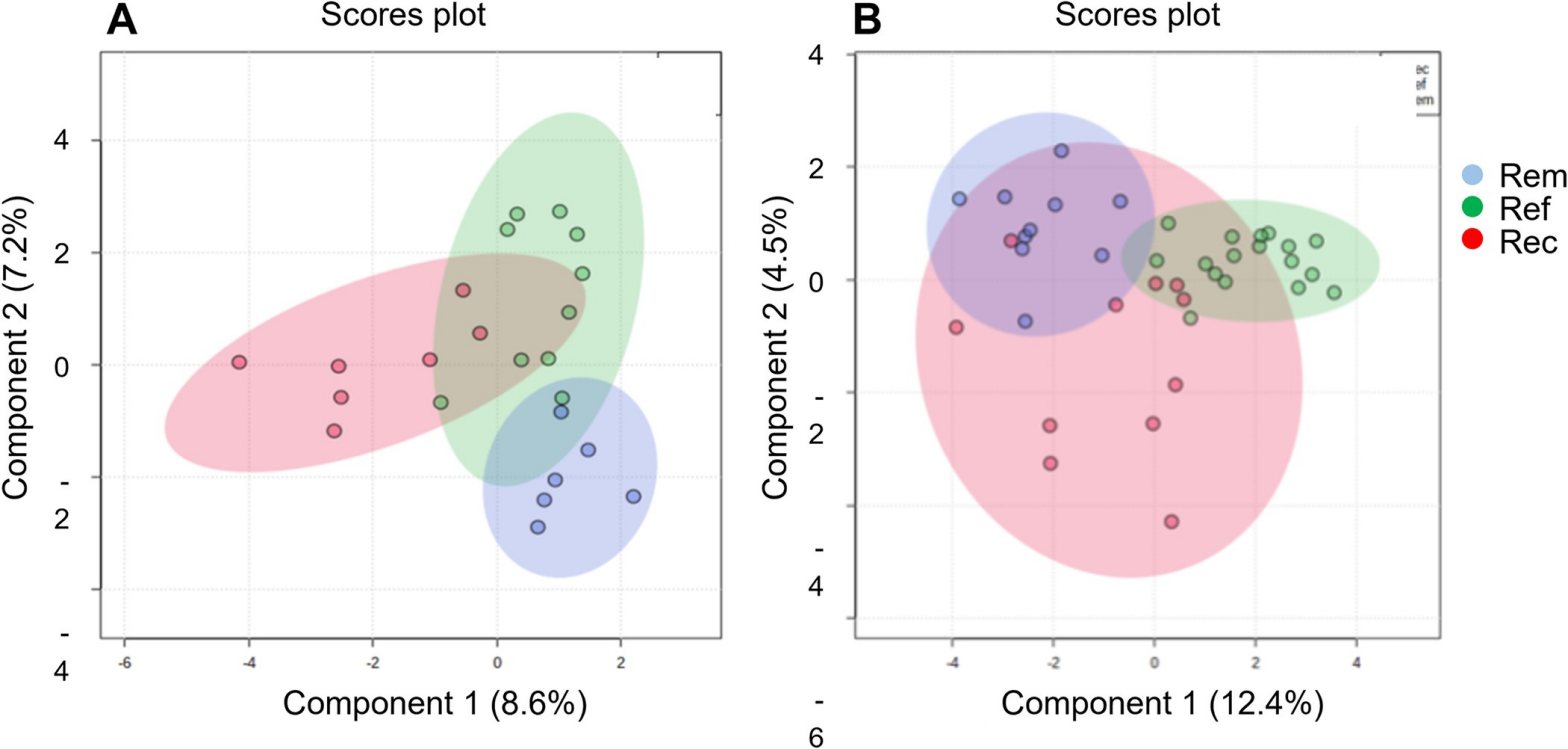
Sparse-PLSDA analysis of pre- and posttreatment samples by clinical outcome group. Normalized, unfiltered data was log10 transformed in MetaboAnalyst.ca platform. (A) pretreatment reiterative sPLSDA analysis using the integration of the major *Gardnerella* species. The top 23 species S8a Table in S3 File from the initial sPLSDA analysis were input into a second sPLSDA analysis to generate the result shown here. (B) posttreatment reiterative sPLSDA analysis using the unintegrated NGS counts dataset. The shaded zones represent the range of two-component values with the 95% confidence intervals of observed data for each outcome group.

We sought to improve discrimination of pretreatment samples by integrating data on the proportion of total *Gardnerella*, identified by V4 sequences, with previous qPCR data that partially resolved *Gardnerella* to species levels. In our previous qPCR study [15], clade-specific primers targeting the *Gardnerella* cpn60 gene were able to quantify individual or subsets of genomospecies among some of the sample samples subjected to NGS sequencing in the current study. To incorporate the qPCR data into our sequencing dataset, normalized relative abundance per patient was calculated as the relative abundance by qPCR of each *Gsp* species relative to the total bacterial abundance, based on qPCR with broad-spectrum primers [15], times the normalized *Gardnerella* counts present in our sequence reads (S9 Table in S1 File). Performing two cycles of sPLSDA modeling on this integrated dataset, differentiated pretreatment patient samples by outcome group and yielded a sPLSDA classification error rate of 26% (Fig 5A). Key species for this resolution were *Gardnerella*_Gsp07, *Megasphaera lornae*, and *Aerococcus christensenii*. (S8c, S8d Table in S3 File). These conclusions of pretreatment samples are provisional, however, due to low sample numbers; posttreatment sample numbers were too low in the integrated *Gsp* dataset to allow an analysis using these major *Gardnerella* species at the posttreatment visit, could not be performed.

In contrast to pretreatment samples, sparse PLSDA separated refractory from most recurrent posttreatment patient samples without resorting to the *Gsp* subset of data (Fig 5B). This analysis had a classification error rate of only 15.8% and pointed to a small number of species that best differentiated the outcome groups. A subset, but not all, of these species, *A*. *vaginae*, *M*. *hominis*, *Gardnerella*, *Dialister sp*, Gemella asaccharolytica*, and *L*. jensenii* were components of multivariant biomarkers that were prognostic of clinical outcomes. Differentiation of refractory patient samples at posttreatment was expected, since recurrent and remission patients were both in clinical remission and typically show prevalence or dominance of one or two species of *Lactobacillus*. However, this early detection of elevated abundance of species of patients who were transiently in remission versus those who achieved long-term remission was not a given; examples include *Lachnospiraceae_1(BVAB1)*, *Corynebacterium spp*, and *Ureaplasma urealyticum* (Fig 5B, S8e, S8f Table in S3 File).

### Overview of core microbiota by clinical outcome and treatment status

**Fig 6:**
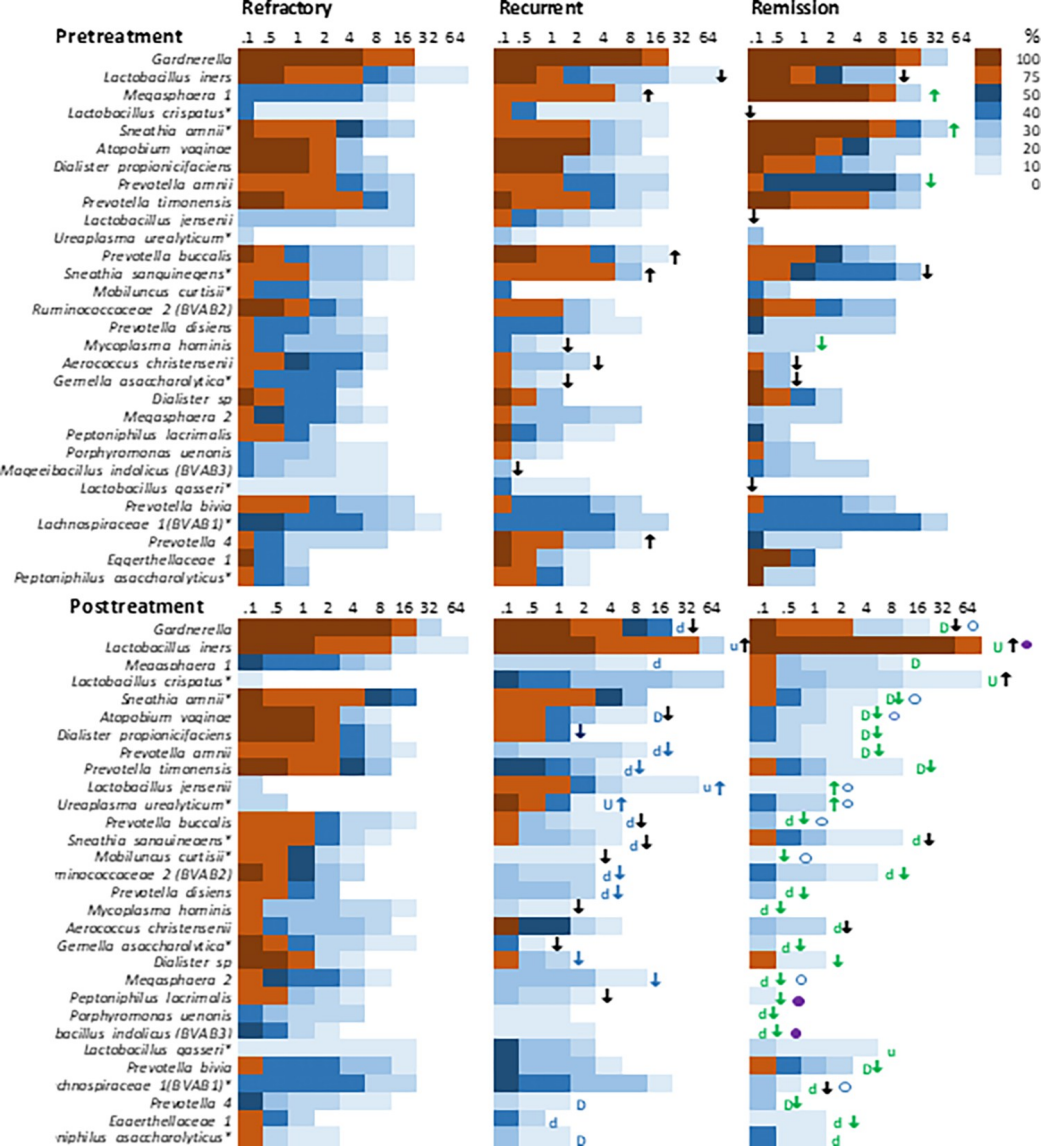
Heat maps of abundance and prevalence of core microbiota of refractory, recurrent and remission patient samples pre- and posttreatment. The color scale classifies upper limits of % prevalence categories. Percent abundance is binned as indicated on the x-axis. Outcome group comparisons (horizontal): ↑ or ↓ indicates a large visual up or down change in either abundance or prevalence of the species relative to that seen in the refractory patient, color coded if the difference was significant (p value <0.05 by abundance using the Kruskal Wallis test, Bonferroni corrected, or by prevalence using ChiSq or Fisher’s exact tests, SPSS). The open circle indicates a large visual change in abundance or prevalence of a remission sample species relative to recurrent samples; a closed circle indicates that the difference was significant (p <0.05, by abundance using Kruskal Wallis test, Bonferroni corrected, or by prevalence using ChiSq or Fisher’s exact tests, SPSS). Pre- versus posttreatment comparisons of change in relative abundance (top versus bottom panel): U and D denote large up or down changes, capitalization indicates the difference was significant (p < 0.05 by abundance assessed by Wilconox rank match pairs tests or by prevalence assessed by paired samples proportions tests with Wald’s test of significance (SPSS).

We characterized the core microbiomes of these groups, defined as species present in at least 20% of samples a > 1% of sample abundance for any outcome group, using heat maps that categorize prevalence levels at varying ranges of percent abundance (Fig 6). For example, 75–100% of pretreatment refractory patient samples have *Gardnerella* at ≥ 0.1% abundance but none of these samples have *Gardnerella* at ≥ 32% prevalence. First, overall compositions of the 3 outcome groups at pretreatment were similar, since only 4 species differed significantly among remission versus refractory patients; other species varied but did not achieve statistical significance. Secondly, overall changes in composition among refractory patients did not change significantly pre- versus posttreatment. In contrast, many species among remission patients declined in prevalence and/or abundance after treatment, and recurrent patient samples were intermediate, where many species decreased posttreatment but were less likely than remission patients to achieve statistical significance. Thirdly, many species were less prevalent and/or less abundant among posttreatment recurrent patients and especially those in remission, compared to refractory patients, and many were also lower among remission than recurrent patients.

Looking at a more detailed level at the most abundant species, *Gardnerella* was not differentially distributed among outcome groups at pretreatment, and the observed reductions among posttreatment recurrent and remission patients relative to refractory patients did not achieve statistical significance. *L*. *iners* trended higher among pretreatment refractory patients but not significantly so; increases at posttreatment were significant among remission patients, but intermediate among recurrent patients. Among less abundance species at pretreatment, the biggest difference in species composition between outcome groups was the higher prevalence of abundant *Megasphaera lornae* among pretreatment recurrent and especially remission compared to refractory patient samples. Also of note was the higher prevalence and abundance of *Sneathia amnii*, and lower prevalence of Prevotella amnii*, among pretreatment remission patients compared to refractory and recurrent patients. In pretreatment samples, several species were less abundant or prevalent among recurrent and remission patient samples compared to refractory, but none of these achieved statistical significance.

Among posttreatment samples, there was a significant downward trend in the relative abundance of *Dialister sp* and *M*. curtisii* among the posttreatment refractory>recurrent>remission samples. Specifically, the relative abundance of *Dialister sp* was significantly higher in refractory patients compared to the other two outcome groups and the relative abundances of *M*. curtisii* and *G*. asaccharolytica* were significantly higher in refractory patients than in remission patients. While *Megasphaera lornae* was reduced in prevalence among posttreatment remission patients, the difference between remission and recurrent patients was statistically significant. Though the relative abundance of *A*. *vaginae* and *A*. *christensenii* were lower among remission patients compared to recurrent patients, the difference was not statistically significant at the posttreatment visit. However, there was a significant downward trend in the abundance of A.vaginae at the posttreatment visit, across refractory>recurrent>remission samples. Like *A*. *vaginae*, other signature species of symptomatic BV, such as *Prevotella spp* and *Sneathia spp* were differentially distributed refractory > recurrent > remission patient samples. Several species dropped from detectable at pretreatment to below the limits of detection among all posttreatment remission samples, including *M*. *hominis*, *Dialister* 1, and *P*. *uenonis*, however, most species were not eliminated. Conversely, *L*. *crispatus*, and *L*. *jensenii* were below detection among almost all pretreatment remission patient samples but were detected at posttreatment, most dramatically *L*. *crispatus*: in ~10% of the posttreatment samples *L*. *crispatus* had risen to 64% of total bacteria. At the posttreatment visit, while *L*. *jensenii* was reduced in remission patients, there was no significant difference compared to the recurrent patients.

### Differential response of common species to metronidazole between outcome groups

No species in refractory patient samples changed dramatically in abundance or prevalence after metronidazole treatment, whereas those same species typically decreased among recurrent and especially remission patient samples (Fig 6). Consistently, alpha diversity metrics showed that there were no significant changes in pre- versus posttreatment refractory patient samples, whereas overall prevalence of species among posttreatment recurrent and remission samples decreased significantly, by 15–20% (Fig 2). This point is augmented in before-after plots of individual species per refractory patient (S4 Fig). Among refractory patient samples, no species showed a robust or prevalent up or down trend following treatment. However, some of these same species shifted dramatically after treatment among recurrent and remission patient samples. Most notable among remission patient samples were decreases in abundance of *Gardnerella*, *Megasphaera lornae*, *Sneathia amnii*, with less dramatic but significant decreases in *A*.*vaginae*, *Prevotella timonensis*, and *Dialister sp*, and robust and pervasive increases in *L*. *iners*. These changes were also noted among recurrent samples, but to lesser extents and incomplete prevalence. While no upward or downward trend was found in the abundance of *U*. *urealyticum* and *P*. asaccharolytica* among refractory and remission patients, these two species increased and decreased significantly, respectively, after metronidazole treatment among recurrent patients. Species that persistently decreased or did not increase after treatment in association with remission but not recurrence: *U*. *urealyticum*, *Gardnerella*, *S*. *amnii*, and *Megasphaera lornae*, along with a more robust increase in *L*. *iners*. Species that persistently decreased in association with a non-refractory response: *A*. *vaginae*, *Prevotella_4*, *Gardnerella*, *S*. *amnii*, *Megasphaera lornae*, *P*. timonensis*, and *P*. asaccharolytica*.

### Key species were associated with outcome among pretreatment sample groups

Despite the lack of distinction of overall compositions of outcome groups at pretreatment (Fig 6), small numbers of key species were significantly different or trended differently and were therefore candidates for prognostic markers or etiologic agents, alone or in combinations. These species were preliminarily identified by multiple statistical approaches since no single approach is definitive (see Methods) and by visualizations on scatterplots (S3 Fig) and on heat tree maps if species comparisons generated a Wilcoxon Rank Sum test p value ≤ 0.05 (S10 Table in S4 File). We combined species identified by all approaches with those from a filtering approach in which 96 species derived by filtering 868 vaginal species in Microbiomeanalyst with the criteria of 2 reads minimum, 10% minimum prevalence, and 5% minimum variance across samples. These species were analyzed by multivariate ROC analysis; species were removed from inclusion in the multivariate ROC analysis one at a time and eliminated if the exclusion did not result in a lower AUC value. Patient samples above or below ROC thresholds were counted as input for Fisher’s exact tests to generate the prognostic statistics in Table 1. Data indicated that pretreatment abundance of 3 species (*M*. *indolicus*, *M*. curtisii*, and *M*. *hominis*) above thresholds predicted a refractory response with 80.6% accuracy. Pretreatment elevated levels of *Finegoldia magna* and *Gardnerella Gsp07*, combined with lower levels of *Megasphaera lornae*, predicted a refractory or recurrent response with 87% accuracy. At posttreatment, elevated levels of *Gardnerella*, *Dialister sp*, *G*. asaccharolytica*, and *M*. curtisii*, combined with lower levels of *L*. *iners*, were diagnostic of the refractory response to treatment with 97.3% accuracy. Among Amsel-negative patients at posttreatment, elevated levels of 5 species, including *Gardnerella* and *A*. *vaginae*, with lower levels of *Megasphaera lornae*, predicted a subsequent recurrence with 95% accuracy.

**Table 1:** Species combinations with significant prognostic value.

	Pretreatment				Posttreatment		
Group 1	Ref^1^	RefRec^1^	Ref Hi^1^		Ref^2^		Rec^2^
Group 2	RecRem	Rem	Ref Lo		RecRem		Rem
Species 1	*Mageeibacillus indolicus(BVAB3) (0*.*003)*	*Finegoldia magna (0)*	*Atopobium vaginae (2*.*3)*	*Mageeibacillus indolicus(BVAB3) (3*.*49)*	*Mageeibacillus indolicus(BVAB3) (3*.*49)*	*Dialister sp (1*.*4)*	*Aerococcus christensenii (0*.*092)*
Species 2	Mobiluncus curtisii* 0.*228)*	*Gardnerella Gsp07 (0*.*007)*	***Megasphaera 1 (0*.*009)***	*Anaerococcus mediterraneensis (2*.*51)*	*Prevotella timonensis (5*.*63)*	*Gardnerella (9*.*2)*	*Atopobium vaginae (0*.*460)*
Species 3	*Mycoplasma hominis (0*.*003)*	***Megasphaera1 (8*.*3)***	*Prevotella timonensis (4*.*6)*			Gemella asaccharolytica* (0.*276)*	*Gardnerella (6*.*9)*
Species 4			***Sneathia sanguinegens (0*.*921)***			***Lactobacillus iners (27*.*6)***	Lactobacillus jensenii* (0.*092)*
Species 5						Mobiluncus curtisii* (0.*138)*	***Megasphaera 1 (0*.*018)***
Species 6	* *	* *	* *	* *	* *	* *	*Megasphaera 2 (0)*
ROC AUC	0.759	0.964	0.689	0.93	0.887	0.947	0.86
95% CI	0.553–0.94	0.816–1	0.327–1	0.492–1	0.333–1	0.833–1	0.561–1
Sensitivity	0.824	0.882	1	1	0.833	0.938	1
95% CI	0.566–0.962	0.636–0.985	0.715–1	0.541–1	0.359–0.996	0.698–0.998	0.715–1
Specificity	0.789	0.833	0.833	1	1	1	0.9
95% CI	0.544–0.939	.359-.996	0.359–0.996	0.398–1	0.398–1	0.839–1	0.555–0.997
PPV	0.778	0.938	0.917	1	1	1	0.917
95% CI	0.524–0.936	0.698–0.998	0.615–0.998	0.541–1	0.478–1	0.782–1	0.615–0.998
NPV	0.833	0.714	1	1	0.8	0.955	1
95% CI	0.586–0.964	0.290–0.963	0.478–1	0.398–1	0.284–0.995	0.772–0.999	0.664–1
Odds Ratio	17.5	37.5	84.3	117	33	444	146
95% CI	0.31–92.5	2.77–508	2.93–2425	1.94–7067	1.06–1024	16.9–11660	5.29–4008
Accuracy	0.806	0.870	0.941	1.00	0.9	0.973	0.952
95% CI	0.640–0.918	0.664–0.972	0.713–0.999	0.69–1	0.55–1.00	0.858–0.999	0.762–0.999
p-value	0.0006	0.0034	0.001	0.0048	0.0476	<0.0001	<0.0001

Thresholds in () after each species is the % abundance value from ROC analysis used to count patient samples; normal font of species indicates that Group 1 abundance is ≥ the threshold and bold font indicates that group 1 abundance is < the threshold. ROC analysis performed in Metaboanalyst.ca. Accuracies were calculated at MedCalc. Fisher tests and statistics were performed with GraphPad Prism. ^1^ Combination of two out of total number of key species identified to give best predictive values. ^2^ Combination of three out of total number of key species identified to give best predictive values. Three alternative sets of biomarker species were determined for the Ref Hi versus Ref Lo groups. Abbreviations: Ref: refractory; Rec: recurrent; Rem: remission; RefRec: patients from both refractory and recurrent outcome groups; RecRem: patients from both recurrent and remission outcome groups; Ref Hi: subset of refractory patients with high abundance (>1%) of Gardnerella Gsp07; Ref Lo: subset of refractory patients with low abundance (≤1%) of Gardnerella Gsp07; CI: confidence interval. Three alternative sets of biomarker species were determined for the Ref Hi versus Ref Lo groups.

Previously, we reported that higher pretreatment abundance of *Gardnerella Gsp07* was associated with refractory or recurrent outcomes based on qPCR data [15]. This we confirmed with our current sequencing data (S9 Table in S1 File). Specifically, *Gardnerella Gsp07* was significantly more abundant among refractory samples than in remission samples and there was a downward trend in the abundance of *Gardnerella Gsp07* from refractory>recurrent>remission samples (S3 Fig). However, there were exceptions, i.e. some refractory patients presented with low *Gardnerella Gsp07* abundance similar to recurrent or remission patients [15]. In this study, we saw that other species were differentially distributed among the refractory patients with abundant *Gardnerella Gsp07* (GspHi, >1% relative abundance) versus those low, <1% Gsp07 (GspLo). sPLSDA modelling revealed a clear distinction between the two groups, with classification error rates as low as 20%, implying significant amounts of variation between the two groups (S8g Table in S3 File). Notably, refractory GspLo patients had higher abundance levels of *Mageeibacillus indolicus (BVAB3)*, *P*. *timonensis*, and *Anaerococcus mediterraneensis*, and reduced abundance of *Gardnerella swidsinski_leopoldii* and, by definition, lower *Gardnerella Gsp07*. These patients may have an alternative form of BV in which species other than Gsp07 marks or determines their status and their refractory response.

### Increased relative abundance of *L*. *iners* or total *Lactobacillus* species at the posttreatment visit was associated with satisfactory early clinical response but did not predict later recurrence

**Fig 7:**
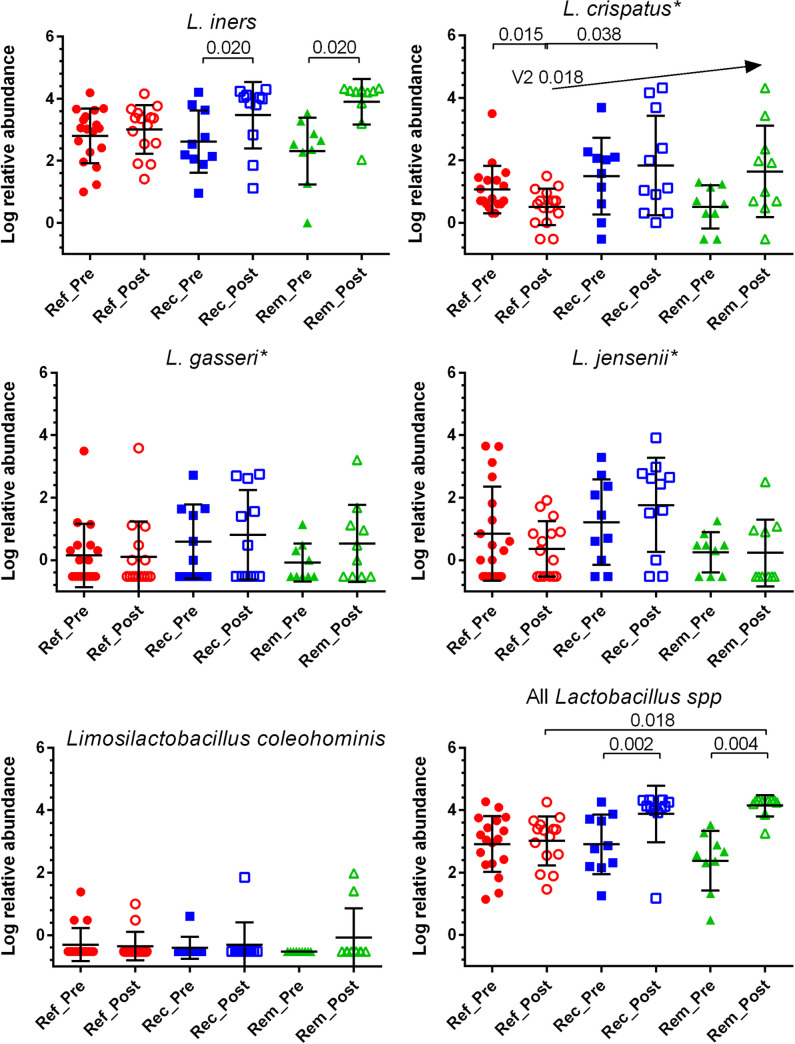
Relative abundance of *Lactobacillus* species pre- and posttreatment among outcome groups. Normalized relative abundances of all *Lactobacillus* species were summed per outcome group. Pre- versus posttreatment samples for each of the 3 outcome groups were compared by the related samples Wilconox signed rank test. Cross-group comparisons among pre- and posttreatment samples were analyzed by Kruskal-Wallis tests of independent samples with post-hoc pairwise tests; p values were adjusted for multiple comparisons with the Bonferroni correction. * indicates significant difference. Analyses were performed with SPSS V28, graphed with GraphPad Prism V6.07.

The relative abundance of *L*. *iners* and combined *Lactobacillus* species increased significantly after treatment among recurrent and remission patient samples by 3.5 to 21-fold, respectively, but not among refractory samples (Fig 7). Mean abundance of *L*. *crispatus* trended upward after treatment among remission and recurrent patient samples but did not achieve statistical significance; *L*. *crispatus* decreased significantly among refractory patient samples after treatment. Although mean abundance among remission posttreatment patient samples was higher than recurrent posttreatment samples, the differences were not significant (p = 0.343) and therefore this metric alone was not prognostic for recurrence. Importantly, species of *Lactobacillus* individually or collectively were not significantly different in abundance among outcome groups at the pretreatment visit, indicating that these levels were not a major determinant of whether a patient will have a successful outcome to treatment. Additionally, pretreatment *Gardnerella/Lactobacillus* ratios did not generate significant or useful predictive values, in contrast to other studies [67]. Other individual species of *Lactobacillus* were not significantly different in abundance among outcome groups or visits.

## Discussion

This study found many associations of potential therapeutic significance and with implications for pathobiology involved in poor responses to oral metronidazole, summarized in Table 2. Decreased evenness, that is, presence of fewer and more dominant species, was associated with better clinical outcomes of both pretreatment and posttreatment samples, indicating that increased dysbiosis is less likely to respond well to treatment. This is consistent with results of an independent study [89]. PCoA data indicated that overall composition of pretreatment samples was not associated with clinical outcome, likely because variance of most species is not related to outcome. In contrast, this data indicated that overall composition of posttreatment samples differentiated refractory from remission as expected, showing also that some recurrent samples were remission-like, and others were refractory-like in composition. SPLSDA analysis, together with other approaches (Classical Univariate Analysis, Metagenomeseq, EdgeR, DEseq2, and LefSe) generated a short list from which key species were identified by multivariate ROC analysis to predict clinical outcome, with modest accuracy at the pretreatment visit, and with high accuracy in predicting recurrence at the posttreatment visit. These species constitute potential prognostic biomarkers. We also observed a lack of response of all core species to oral metronidazole among refractory patients, within the limits of our sample times. This and the biomarker species have implications for the pathogenesis of recurrent BV.

**Table 2:** Summary of major findings.

Outcome	Pretreatment	Posttreatment
**Refractory**	High species diversity and evenness	Persistently high species diversity and evenness
	**Prognostic (81%)**: ↑of 2 of 3 of *Mageeibacillus indolicus (BVAB3)*, *Mycoplasma hominis*, and Mobiluncus curtisii*	**Diagnostic (97%)**: Higher abundance in 3 of 5 of *Gardnerella*, *Dialister sp*, *G*. *asaccharolytica*, and Mobiluncus curtisii* with ↓ of *Lactobacillus iners*
	↑ of *Gardnerella Gsp07* OR ↑of *Mageeibacillus indolicus (BVAB3) with Prevotella timonensis* or *Anaerococcus mediterraneensis*	No decreases in abundance of core species, including *Lactobacillus spp*.
	Overall microbial composition not associated with clinical response	Distinct microbial composition from remission patients, overlapping composition with some recurrent patients
	*Lactobacillus spp*. composition not associated with clinical response	
**Recurrent**	High species diversity and intermediate evenness	Moderately decreased species diversity and evenness
	**Prognostic**: absence of refractory and remission prognostic biomarkers	**Prognostic (95%)**: ↑ in 3 of 5 species, including *Gardnerella*, *Atopobium vaginae*, *Aerococcus christensenii*, and *Megasphaera_2*, and ↓ of *Megasphaera lornae*
	Overall microbial composition not associated with clinical response	Intermediate decrease in abundance of many species which were unchanged in refractory patients; associated with an increased abundance of *Ureaplasma urealyticum* and a decreased abundance of Peptoniphilus asaccharolytica*
	*Lactobacillus spp*. composition not associated with clinical response	Intermediate increase in abundance of *Lactobacillus iners*
		Species that persistently decreased in association with a non-refractory response: *Atopobium vaginae*, *Prevotella_4*, *Gardnerella*, *Sneathia amnii*, *Megasphaera lornae*, Prevotella timonensis*, and Peptoniphilus asaccharolytica*
**Remission**		
	High species diversity and less evenness	Largest decrease in species diversity and evenness
	**Prognostic (87%)**: ↑ of *Megasphaera lornae* and lower abundance of *Gardnerella Gsp07* or *Finegoldia magna*	**Prognostic (95%)**: ↓ in 3 of 5 species, including *Gardnerella*, *Atopobium vaginae*, *Aerococcus christensenii*, and *Megasphaera_2*, and ↑ of *Megasphaera lornae*
	**Prognostic (78%)**: ↑ of *Megasphaera lornae* alone predicts a remission response	Largest decreases in species abundance, uniquely *Ureaplasma urealyticum*, *Gardnerella*, *Sneathia amnii*, and *Megasphaera lornae*
	Overall microbial composition not associated with clinical response	Distinct microbial composition from refractory patients, overlapping composition with some recurrent patients
	*Lactobacillus spp*. composition not associated with clinical response	Highest increase in abundance of *Lactobacillus iners*

↑: higher abundance relative to other outcome groups; ↓: lower abundance relative to other outcome groups. Increase or decrease: posttreatment changes in abundance relative to pretreatment. Times New Roman font: true of both posttreatment recurrent and remission samples. Prognostic or diagnostic features and % accuracies in bold.

At two extremes of this cohort, we have patients who were refractory to oral metronidazole versus those who achieved long term remission, despite their history of recurrent BV. A signature, prognostic feature of most refractory patients before treatment was a combination of higher prevalence and abundance of *Mageeibacillus indolicus (BVAB3)*, *Mycoplasma hominis*, or Mobiluncus curtisii* compared to recurrent or remission patients. A signature, prognostic feature of pretreatment remission patients was higher abundance of *Megasphaera lornae* and lower abundance of *Gardnerella Gsp07* and *Finegoldia magna*. Most of these species considered alone were not accurate predictors of clinical outcome at pretreatment; the sole exception was that elevated *Megasphaera lornae* predicted remission with 78% accuracy.

This study adds perspective with the finding that patients achieving remission were characterized at pretreatment by higher abundance and 100% prevalence of *Megasphaera Type 1*, now reclassified as *Megasphaera lornae* sp. nov. [90]. This is partly supported by a previous report that *Megasphaera* was associated with a successful clinical outcome to vaginal metronidazole, although this study used intravaginal metronidazole, was short-term, and did not focus on recurrent BV patients [54]. As a working model, we suggest that there are at least two subtypes of recurrent BV; recurrent BV among remission patients is driven by *Megasphaera lornae* and perhaps *Sneathia amnii*, rather than by more virulent *Gardnerella* species. One mechanism by which *Megasphaera lornae* may drive symptomatic BV is its ability to deplete vaginal lactic acid, shown in vitro [90]. This a property shared with species of *Megasphaera* in the gut [91, 92] or in vaginal models [93] and would reverse the lactic acid-mediated inhibition of most BV-associated species normally imposed by *Lactobacillus* [94–97]. *Megasphaera lornae* might also interfere with biofilm production, likely initiated by *Gardnerella*, which would make vaginal species more susceptible to metronidazole [7–12]. However, evidence for involvement of *Megasphaera* in vaginal biofilm is limited and sporadic [98, 99]. Patients whose BV is marked by *Megasphaera lornae* may be more likely to achieve long term remission because it is more susceptible to metronidazole than *Gardnerella* species [68, 100–102], evidenced here by its decline in pairwise comparisons of posttreatment to pretreatment abundance (Fig 7). Conversely, refractory patients are more likely to fail to respond to oral metronidazole because their BV is caused by more virulent and resistant strains such as *Gardnerella Gsp07*, instead of by *Megasphaera lornae*; consistently, *Gardnerella* species did not decline in our pairwise comparisons. Virulent *Gardnerella* species, or a covariant, may also contribute to resistance in novel ways. Recurrent patients are intermediates in this model; their lower abundance of *Gardnerella* and *Megasphaera lornae* allowed only transient remission.

It was a surprising feature of the comparisons of pre- versus posttreatment abundance, that no species showed significant declines among refractory patients whereas many of these same species decreased significantly among recurrent or remission patients after treatment. Since samples were not analyzed over the ~14 days between pretreatment and posttreatment, species in refractory samples may have declined then rebounded; these would then be uniformly tolerant rather than resistant. It is possible, but seems unlikely, that all species in these refractory patients were resistant or tolerant to metronidazole and were of different lineages than the same species in remission patients. It is more plausible that metronidazole in refractory patients was being inactivated or sequestered, as previously suggested [67]. However, in our cohort there was no correlation between abundance of pretreatment *Lactobacillus* abundance and clinical outcome, so that aspect of the published model does not apply here. We suggest that instead the inactivation/sequestration of metronidazole may be due to species or combinations of species that were notably more abundant and prevalent in the refractory group. *Gardnerella Gsp07* was significantly more prevalent and abundant in pretreatment refractory patients [15], so it or its covariants may be responsible for this process. Multivariate ROC analysis suggests that a combination of *Mageeibacillus indolicus (BVAB3)*, *Mycoplasma hominis*, or Mobilucus curtisii* are possible sources of this activity. Of these, *Gardnerella* was shown to be capable of inactivation of metronidazole in vitro [67]. However, *Gardnerella Gsp07* alone cannot account for the lack of responsiveness of core species to metronidazole, because it was observed in both the Gsp07Hi and Gsp07Lo refractory patient subgroups.

Others have reported that the relative abundance of pretreatment *Lactobacillus*, or the ratio of *Gardnerella* to *Lactobacillus* 16S DNA [67, 103] or RNA [104], was associated with clinical outcome. Neither of these associations were significant in our cohort, nor in our larger cohort analyzed by qPCR [15]; abundance of total *Lactobacillus spp* or *L*. *iners* at pretreatment or among Amsel-negative posttreatment patients was not prognostic of clinical outcome. In another study, BV recurrence at one month was associated with *Enterococcus*, *L*. *crispatus*, *Ureaplasma*, *Aerococcus*, and *L*. *jensenii* [55], only minimally overlapping our associated key species, We attribute these differences to the apples to oranges comparison of the studies; cohorts in the published studies did not have prior histories of BV recurrence whereas ours were recurrent BV patients, and we are assigning outcomes based on much longer monitoring (3–9 months) of recurrent and remission status. Similarly, our comparisons of posttreatment patient samples obtained from refractory versus combined Amsel-negative patients in recurrent and remission outcome groups, is very different from comparing healthy women to BV patients who acquire BV only sporadically.

We also saw at posttreatment that combinations of key species differed between recurrent and remission patients, both in clinical remission at that time. Combinations of higher levels of 3 of 5 species, including *Gardnerella* and *A*. *vaginae*, and lower levels of *Megasphaera lornae*, predicted later recurrence with 95% accuracy. Species in recurrent samples at this stage were also significantly more evenly distributed. Upon validation, these biomarkers could be used to continue or modify the standard treatment regimen.

This study has limitations, chiefly in the small numbers and single location of patients in each group, which means that conclusions, while significant, are contingent on confirmation in a larger and multi-site study. The patients are predominantly African-American with histories of frequent recurrence, so conclusions may not apply to other groups. Our recurrent patients were slightly older than remission patients, so an argument that they are different not by vaginal bacterial composition but by age-related factors will need to be addressed when data from more patients in each group are available. The OTU-based approach to species clustering ignores sequence heterogeneity within single or related OTU’s, which could mask associations particularly among less abundant variants. The 16S rRNA, V4 domain sequence does not resolve all vaginal species, although it is likely that species that share V4 sequences are related. ROC-based analysis of the association of key species combinations with clinical outcome is susceptible to overfitting, something which will require a larger and more diverse study to resolve. Any contributory role of vaginal epithelial cell biofilm in women with BV was not explored. This study was focused on oral metronidazole therapy, so conclusions cannot be extrapolated to patients treated with vaginal metronidazole or oral clindamycin. The study was limited to heterosexual women due to power considerations, given our limited enrollment, and therefore conclusions cannot be extrapolated to homosexual or bisexual women. We did not have measures to ensure compliance to the patient protocol, so it is possible that refractory patients simply did not adhere to the oral metronidazole regimen; this is made less likely given that these patients performed and submitted daily swab samples in this interval, along with information such as menses and coitus. Finally, the arguments pertaining to the apparent lack of response to metronidazole among refractory patients are limited by lack of sequencing data on daily samples from the end of treatment at day 7 to our “posttreatment” visit at day 14. This limitation means that we cannot distinguish between resistance, which would have resulted in no decreases in relative abundance all throughout the 7 day course of oral metronidazole, from tolerance, which could result in reduced relative abundance during treatment, but followed by recovery beginning after treatment through the second clinic visit at day 14.

Evidence is presented that vaginal microbial composition or microbiota measured during florid BV and immediately following recommended metronidazole therapy has prognostic significance and is potentially useful in selecting therapeutic antimicrobial agents, both with regard to dose and duration of therapy. Of note is evidence of antimicrobial resistance or tolerance suggestive of metronidazole inactivation or sequestration among refractory patients.

## Supporting information

S1 Fig Phylogenetic trees of 16 individual phyla. (PDF)

S2 Fig Phylogenetic trees all representative OTUs. (PDF)

S3 Fig Relative pre- and post-treatment abundances. (PDF)

S4 Fig Percent abundance of species pre- & posttreatment. (PDF)

S1 File S1 Meta Data, S2 Phylogenetic trees all representative OTUs, S3 Lists of species included in species* groups, S4 Counts of sequences condensed to 865 species/OTUs for compositional analyses, S5 Counts of sequences filtered to 96 species/OTUs for comparative analyses, S6 Counts of sequences filtered to 51 species/OTUs for comparative analyses, S9 Counts of sequences of a subset of pretreatment samples that distribute *Gardnerella* counts into groups of *Gardnerella* species. (XLSX)

S2 File (XLSX)

S3 File (XLSX)

S4 File (XLSX)
