# Supplementary material for: Association of key species of vaginal bacteria of recurrent bacterial vaginosis patients before and after oral metronidazole therapy with short- and long-term clinical outcomes
Source: PLoS One. 2022 Jul 28;17(7):e0272012. doi: 10.1371/journal.pone.0272012 (PMC9333308; doi:10.1371/journal.pone.0272012)
Supplement: S1 Fig — (PDF) [file pone.0272012.s001.pdf]

S1 Fig. Phylogenetic Trees of 16 individual phylum

Evolutionary analysis by Maximum Likelihood method
The evolutionary history was inferred by using the Maximum Likelihood method and Tamura-Nei model [1]. The percentage of trees in which the associated taxa clustered together is shown next to the branches. Initial tree(s) for the heuristic search were obtained automatically by applying Neighbor-Join and BioNJ algorithms to a matrix of pairwise distances estimated using the Tamura-Nei model, and then selecting the topology with superior log likelihood value. The tree is drawn to scale, with branch lengths measured in the number of substitutions per site (next to the branches). All positions with less than 95% site coverage were eliminated, i.e., fewer than 5% alignment gaps, missing data, and ambiguous bases were allowed at any position (partial deletion option). Evolutionary analyses were conducted in MEGA X [2]

1. Tamura K. and Nei M. (1993). Estimation of the number of nucleotide substitutions in the control region of mitochondrial DNA in humans and chimpanzees. Molecular Biology and Evolution 10:512-526.

2. Kumar S., Stecher G., Li M., Knyaz C., and Tamura K. (2018). MEGA X: Molecular Evolutionary Genetics Analysis across computing platforms. Molecular Biology and Evolution 35:1547-1549.

Disclaimer: Although utmost care has been taken to ensure the correctness of the caption, the caption text is provided "as is" without any warranty of any kind. Authors advise the user to carefully check the caption prior to its use for any purpose and report any errors or problems to the authors immediately (www.megasoftware.net). In no event shall the authors and their employers be liable for any damages, including but not limited to special, consequential, or other damages. Authors specifically disclaim all other warranties expressed or implied, including but not limited to the determination of suitability of this caption text for a specific purpose, use, or application.

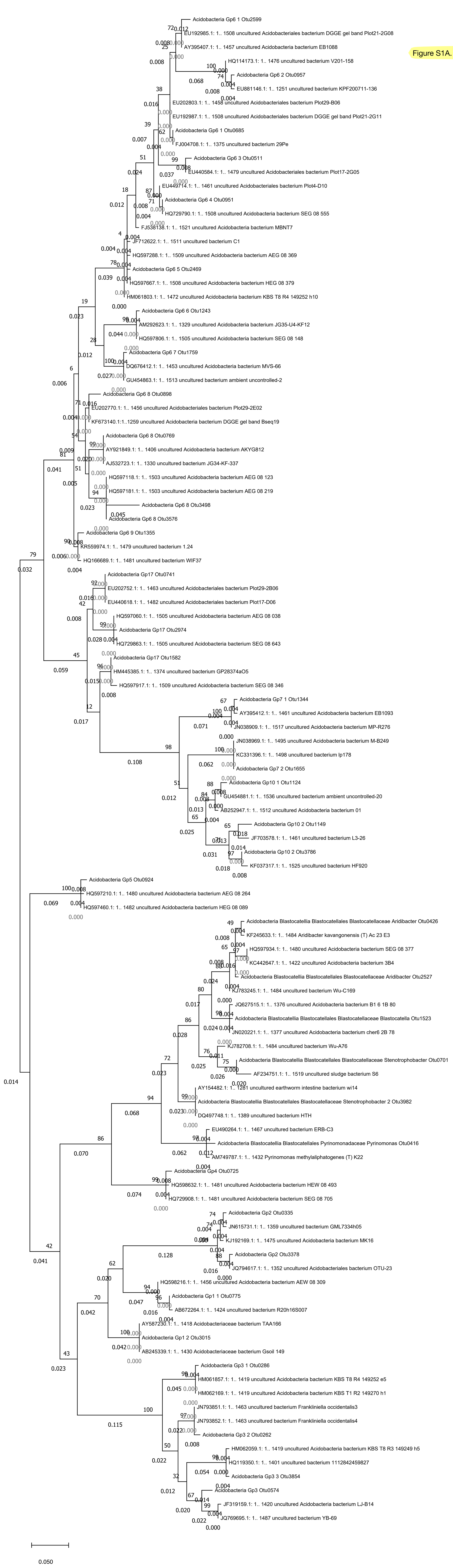


Figure S1A. *Acidobacteria* Phylogenetic tree

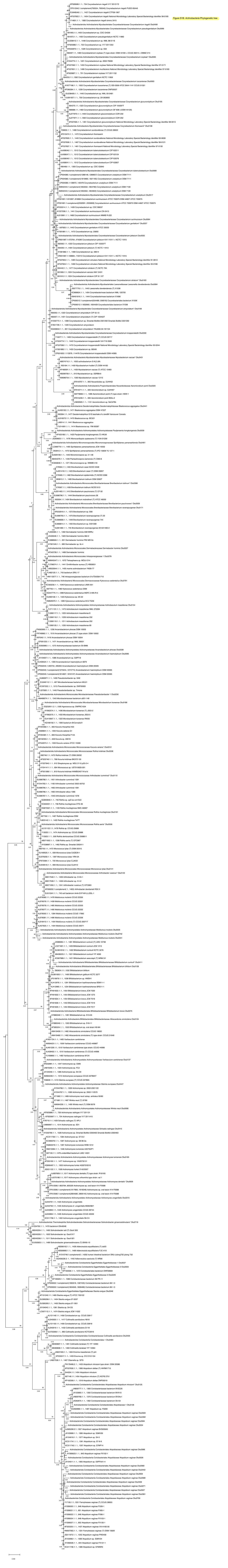

Figure S1B. *Actinobacteria* Phylogenetic tree

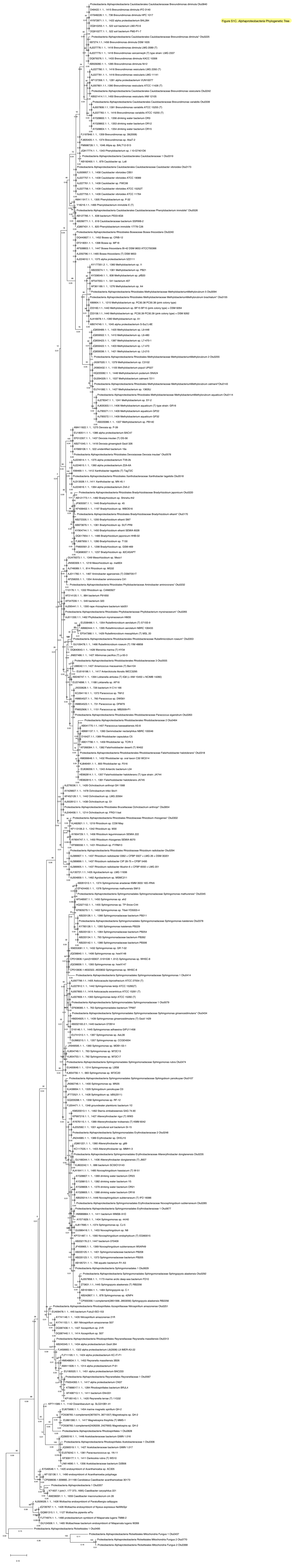

Figure S1C: Alphaproteobacteria Phylogenetic Tree

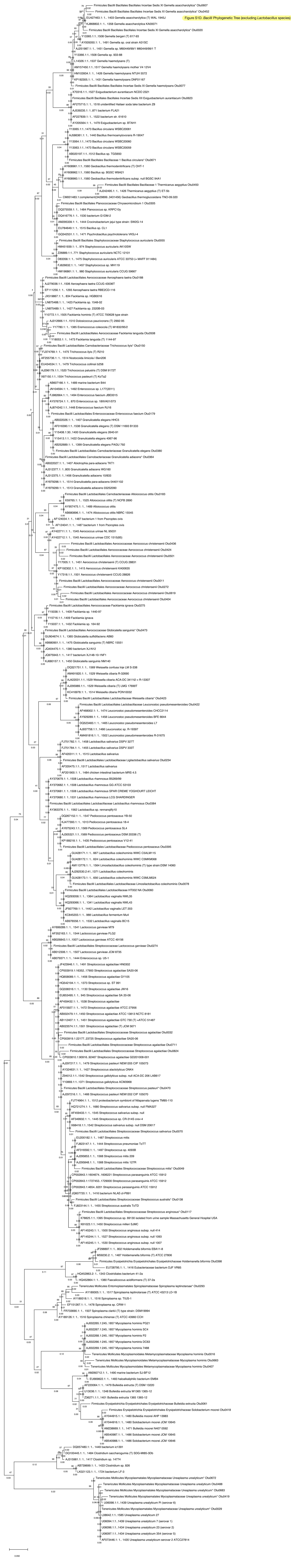

Figure S1D. Bacilli Phylogenetic Tree (excluding Lactobacillus species)

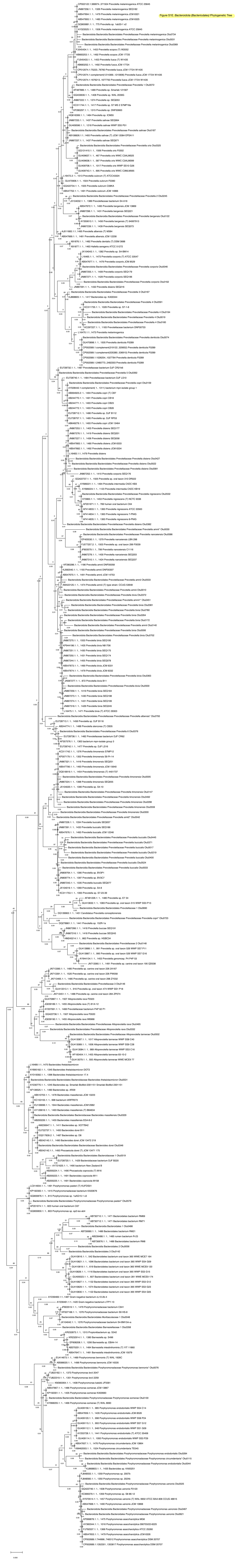

Figure S1E. Bacteroidota (Bacteroidales) Phylogenetic Tree

Figure S1F. *Bacteroidota* Phylogenetic Tree (excluding *Bacteroidales*)

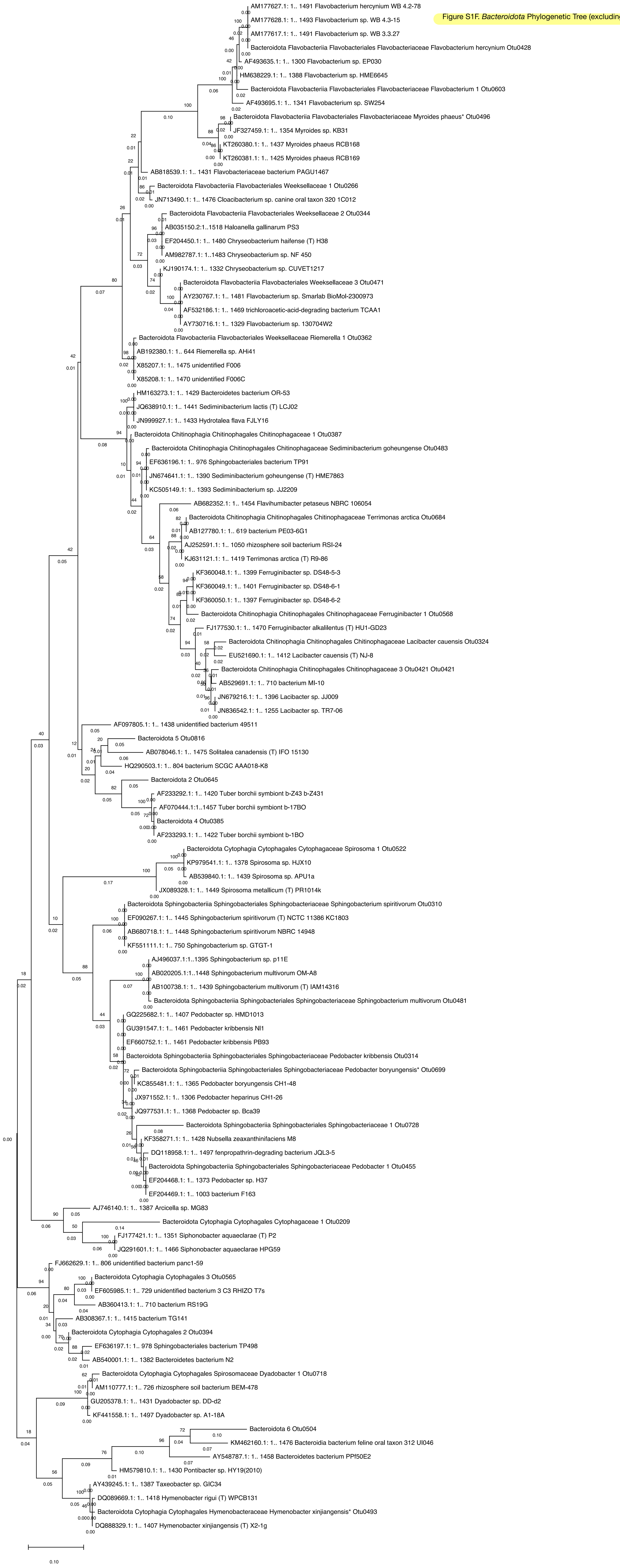

Figure S1G. *Clostridiales* Phylogenetic Tree

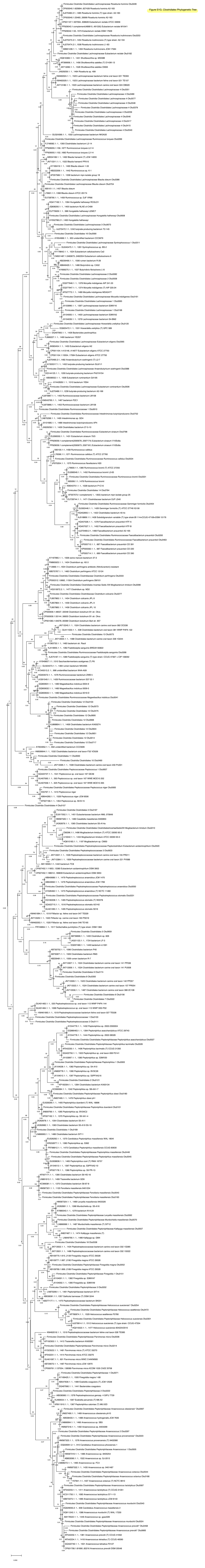

Figure S1H. *Clostridiales* Phylogenetic Tree (excluding *Peptococcus* species and *Lachnospiraceae* species)

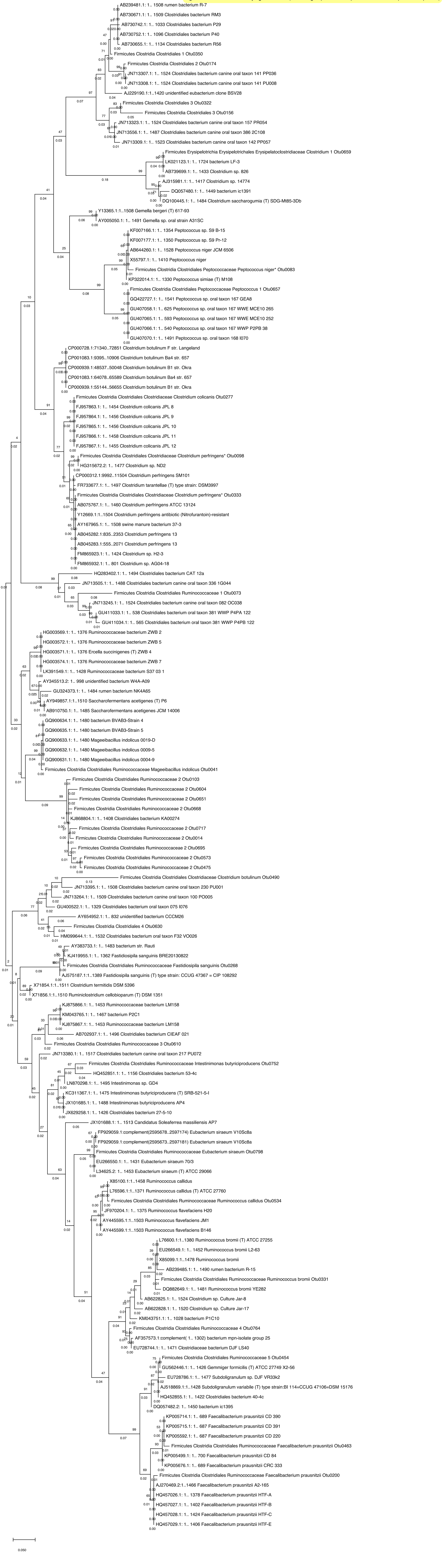

Figure S1I. *Fusobacteria* Phylogenetic Tree

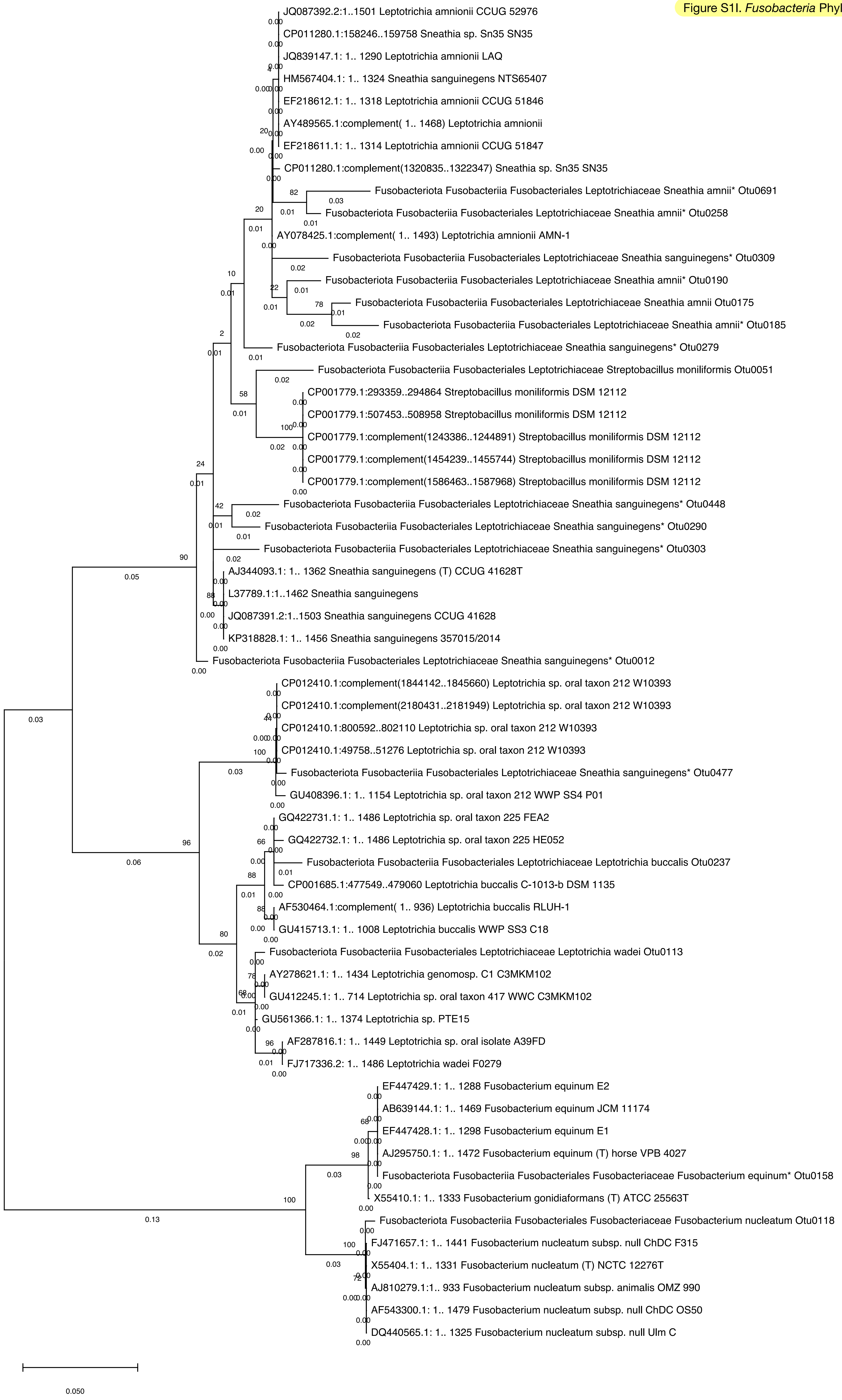

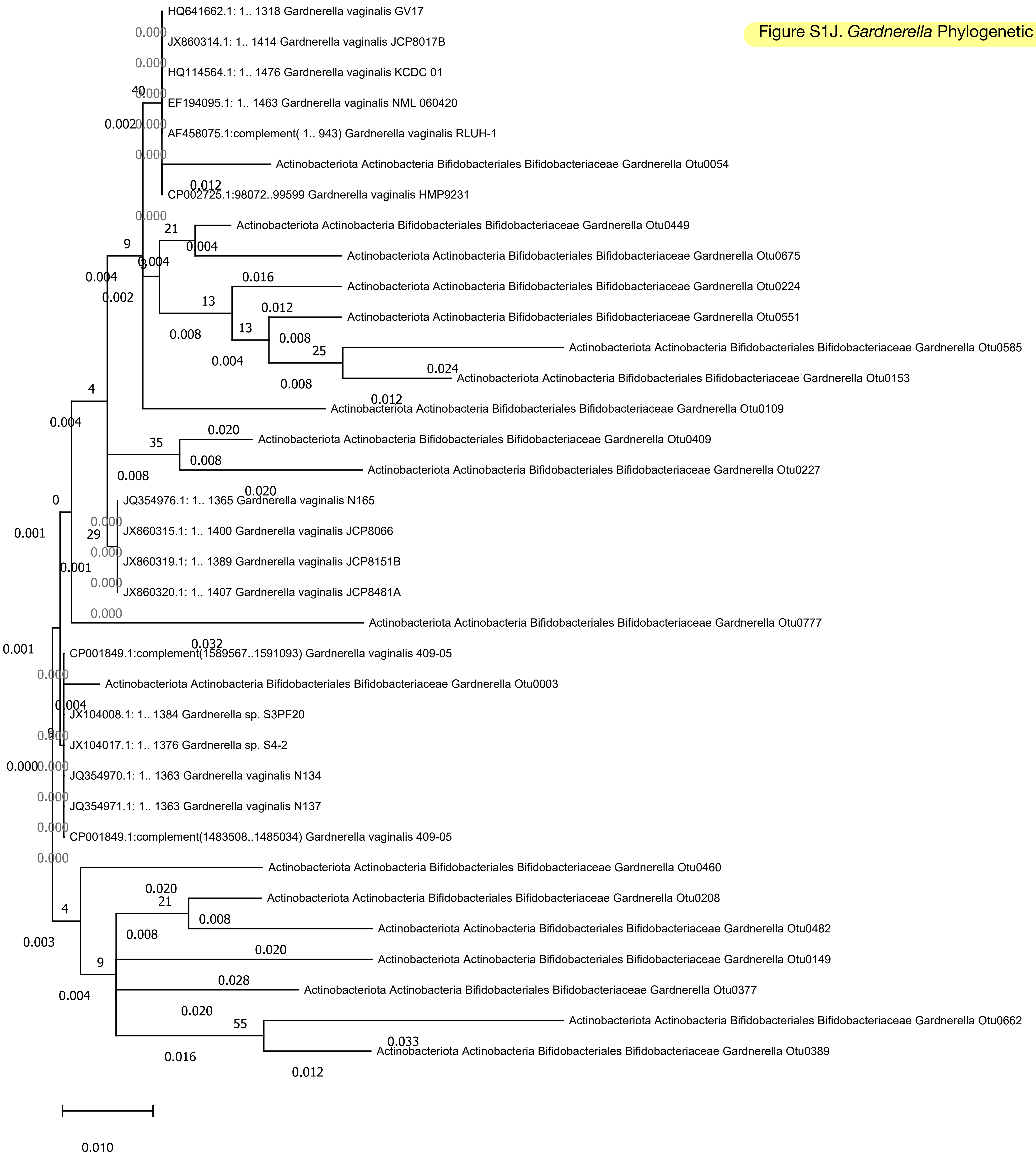


Figure S1J. *Gardnerella* Phylogenetic Tree

Figure S1K. *Lachnospiraceae* phylogenetic tree

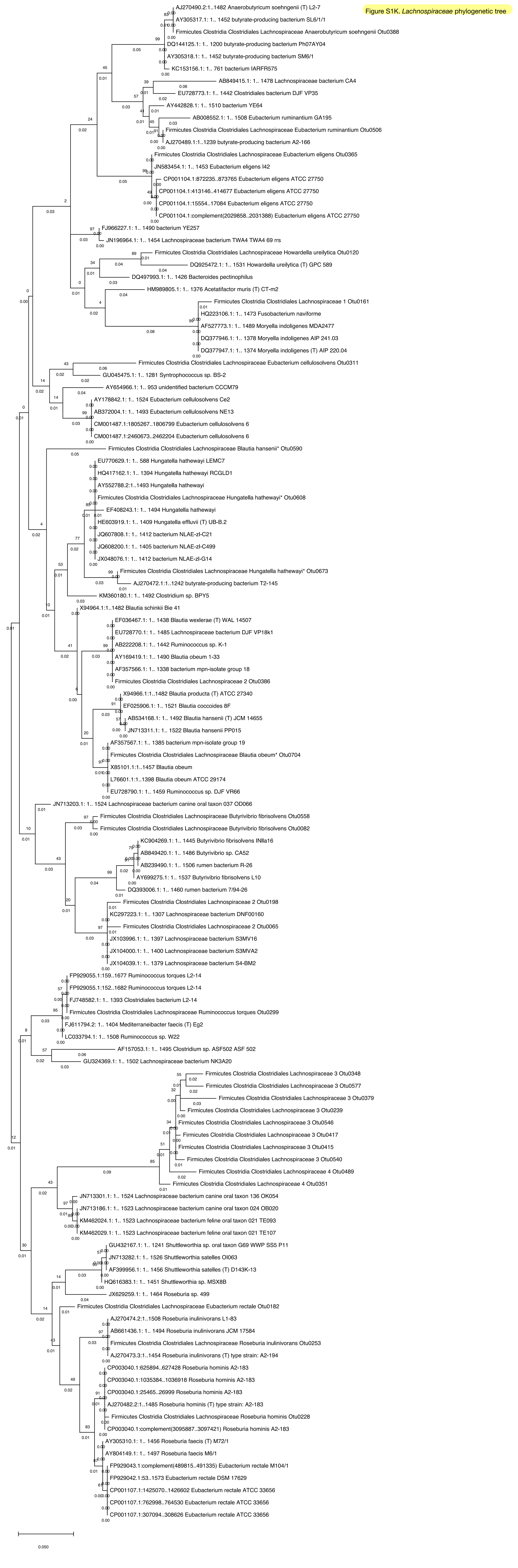

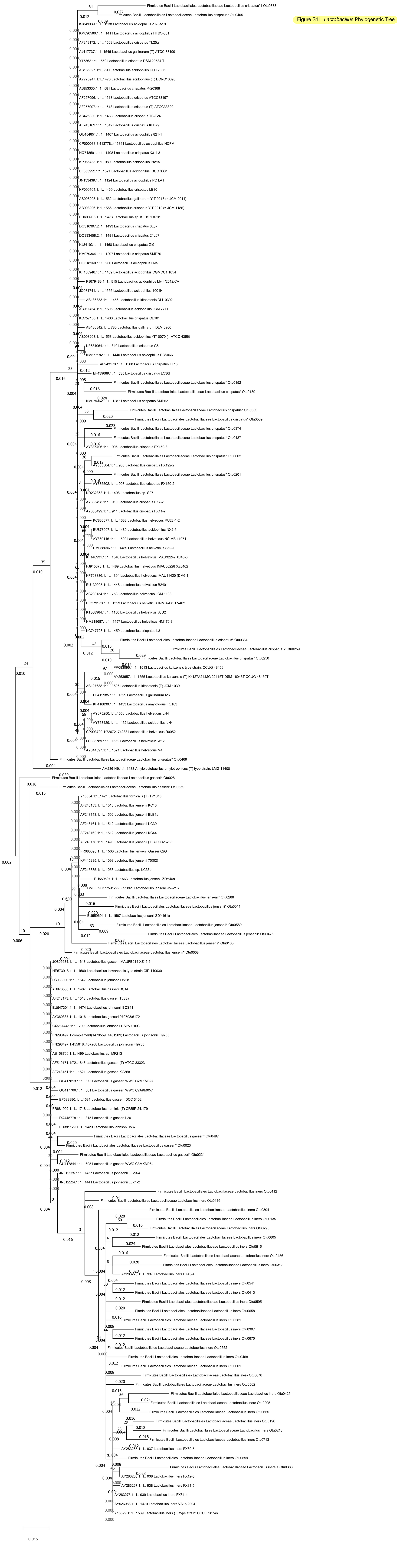

Figure S1L. *Lactobacillus* Phylogenetic Tree

Figure S1M. Miscellaneous Phylogenetic Tree

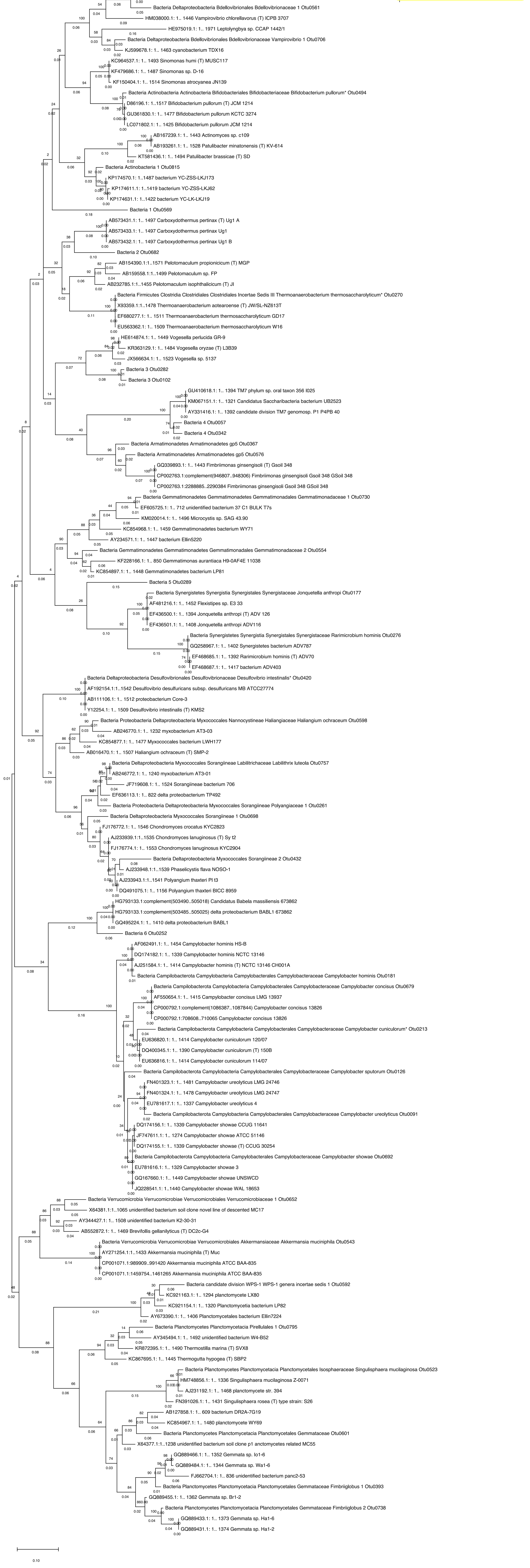

Figure S1N. *Negativicutes* Phylogenetic Tree

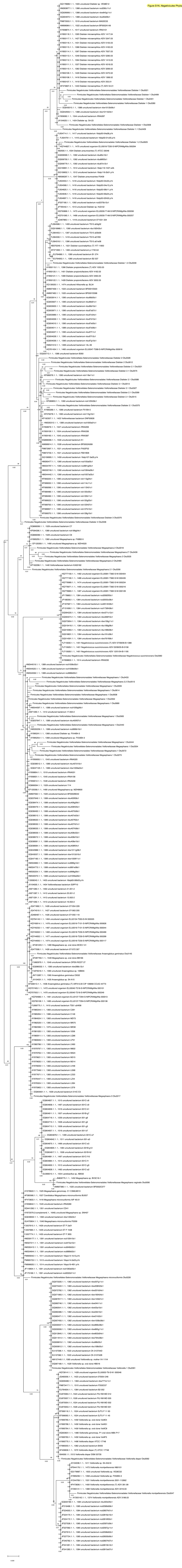

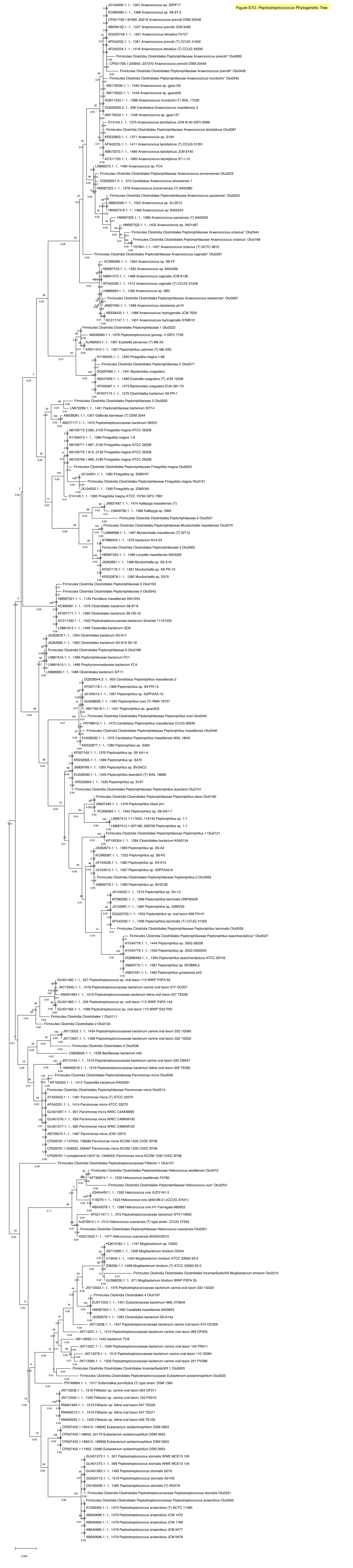

Figure S1O. *Peptostreptococcus* Phylogenetic Tree

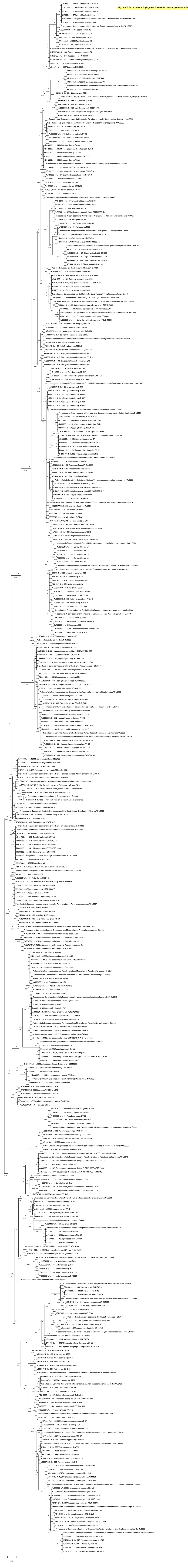

Figure S1P. *Proteobacteria* Phylogenetic Tree (excluding *Alphaproteobacteria* specie)
