# Supplementary material for: Association of key species of vaginal bacteria of recurrent bacterial vaginosis patients before and after oral metronidazole therapy with short- and long-term clinical outcomes
Source: PLoS One. 2022 Jul 28;17(7):e0272012. doi: 10.1371/journal.pone.0272012 (PMC9333308; doi:10.1371/journal.pone.0272012)
Supplement: S3 Fig — (PDF) [file pone.0272012.s003.pdf]

S3 Figure. Relative pre- and post-treatment abundance of all 96 species and relative pre-treatment abundance of the distributed *Gardnerella* species. Normalized relative abundance per patient was log transformed.

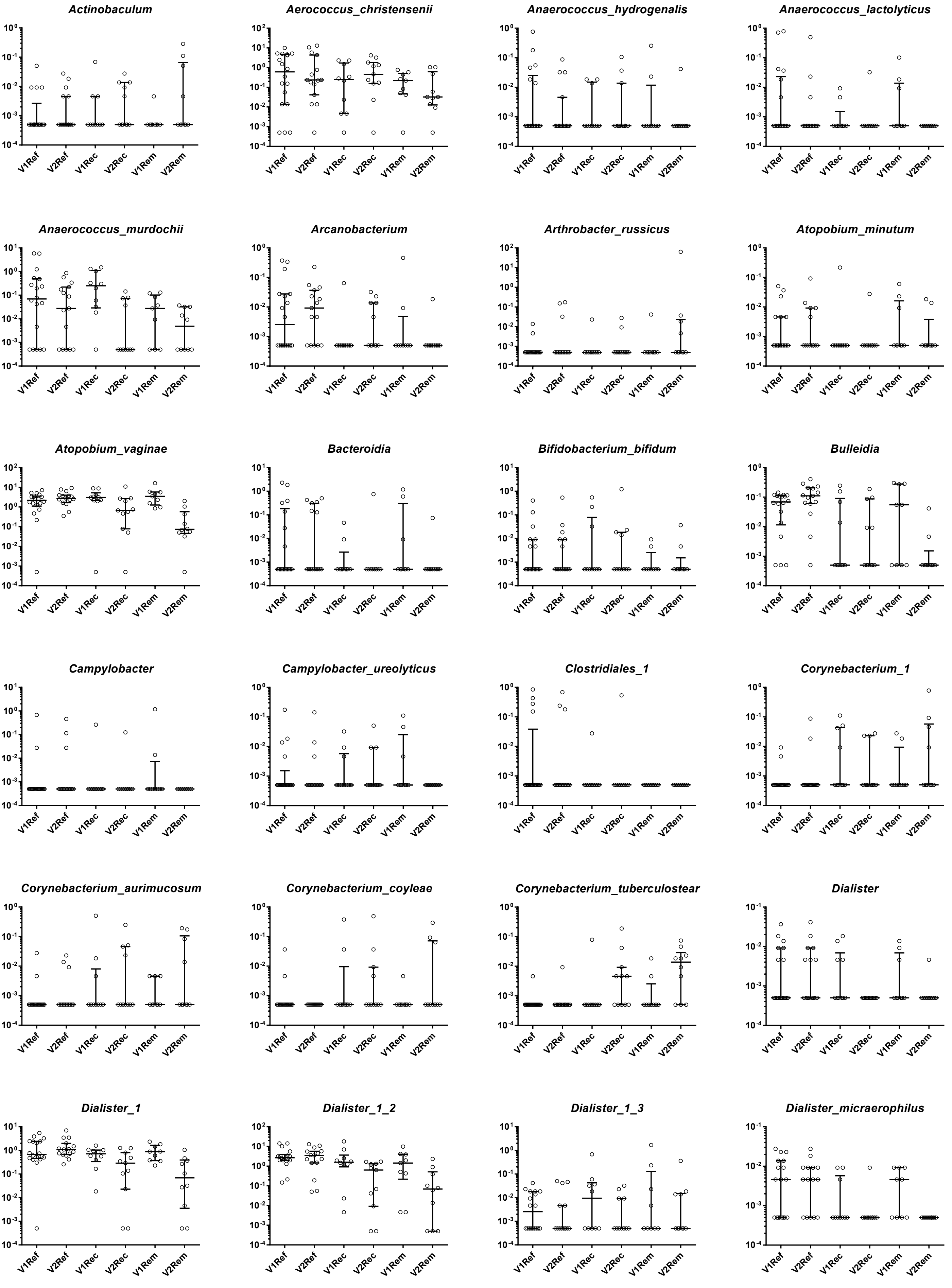
Actinobaculum
Aerococcus_christensenii
Anaerococcus_hydrogenalis
Anaerococcus_lactolyticus
Anaerococcus_murdochii
Arcanobacterium
Arthrobacter_russicus
Atopobium_minutum
Atopobium_vaginae
Bacteroidia
Bifidobacterium_bifidum
Bulleidia
Campylobacter
Campylobacter_ureolyticus
Clostridiales_1
Corynebacterium_1
Corynebacterium_aurimucosum
Corynebacterium_coyleae
Corynebacterium_tuberculostear
Dialister
Dialister_1
Dialister_1_2
Dialister_1_3
Dialister_micraerophilus
V1Ref
V2Ref
V1Rec
V2Rec
V1Rem
V2Rem

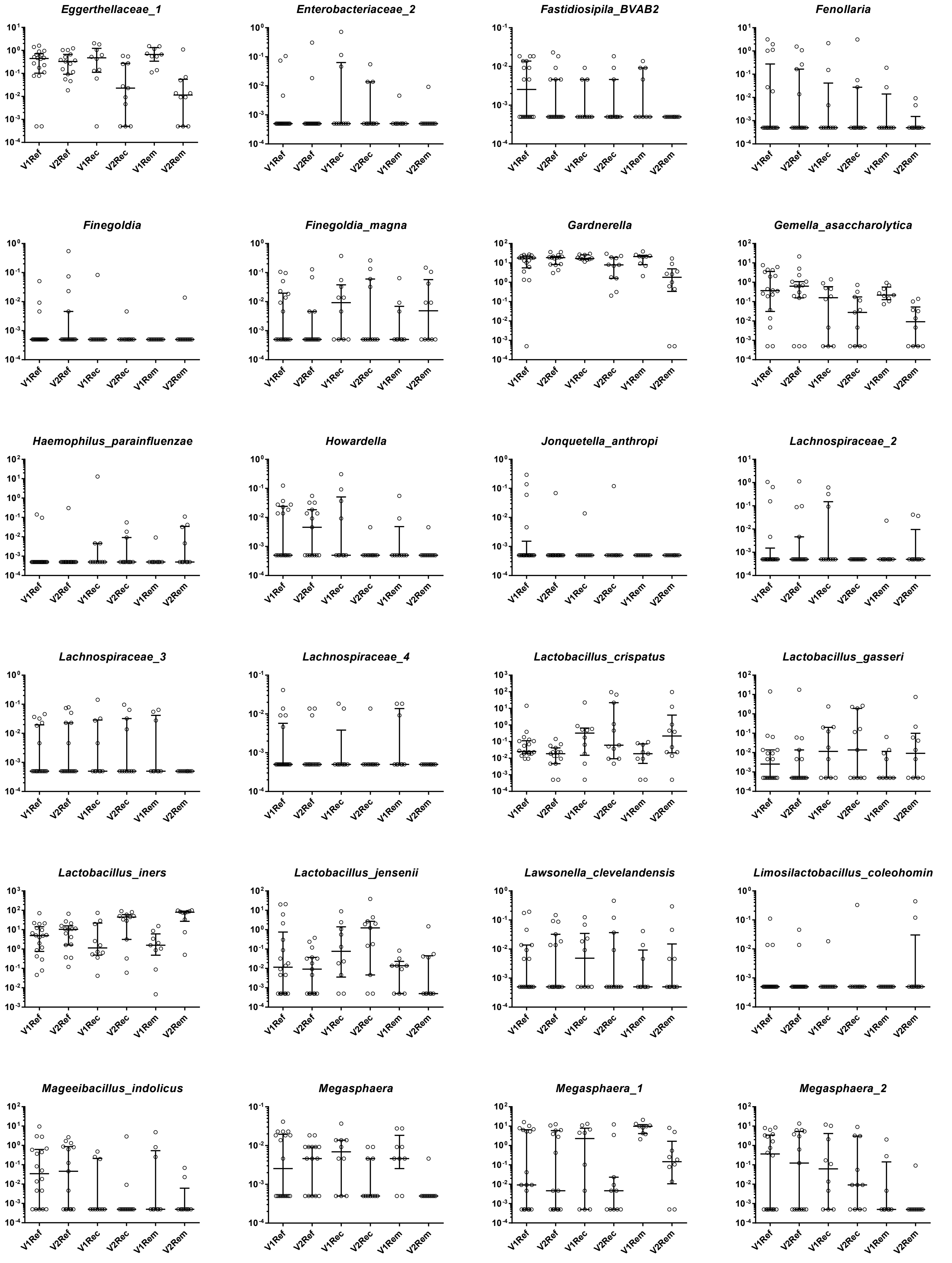
Eggerthellaceae_1
Enterobacteriaceae_2
Fastidiosipila_BVAB2
Fenollaria
Finegoldia
Finegoldia_magna
Gardnerella
Gemella_asaccharolytica
Haemophilus_parainfluenzae
Howardella
Jonquetella_anthropi
Lachnospiraceae_2
Lachnospiraceae_3
Lachnospiraceae_4
Lactobacillus_crispatus
Lactobacillus_gasseri
Lactobacillus_iners
Lactobacillus_jensenii
Lawsonella_clevelandensis
Limosilactobacillus_coleohomin
Mageeibacillus_indolicus
Megasphaera
Megasphaera_1
Megasphaera_2
V1Ref
V2Ref
V1Rec
V2Rec
V1Rem
V2Rem

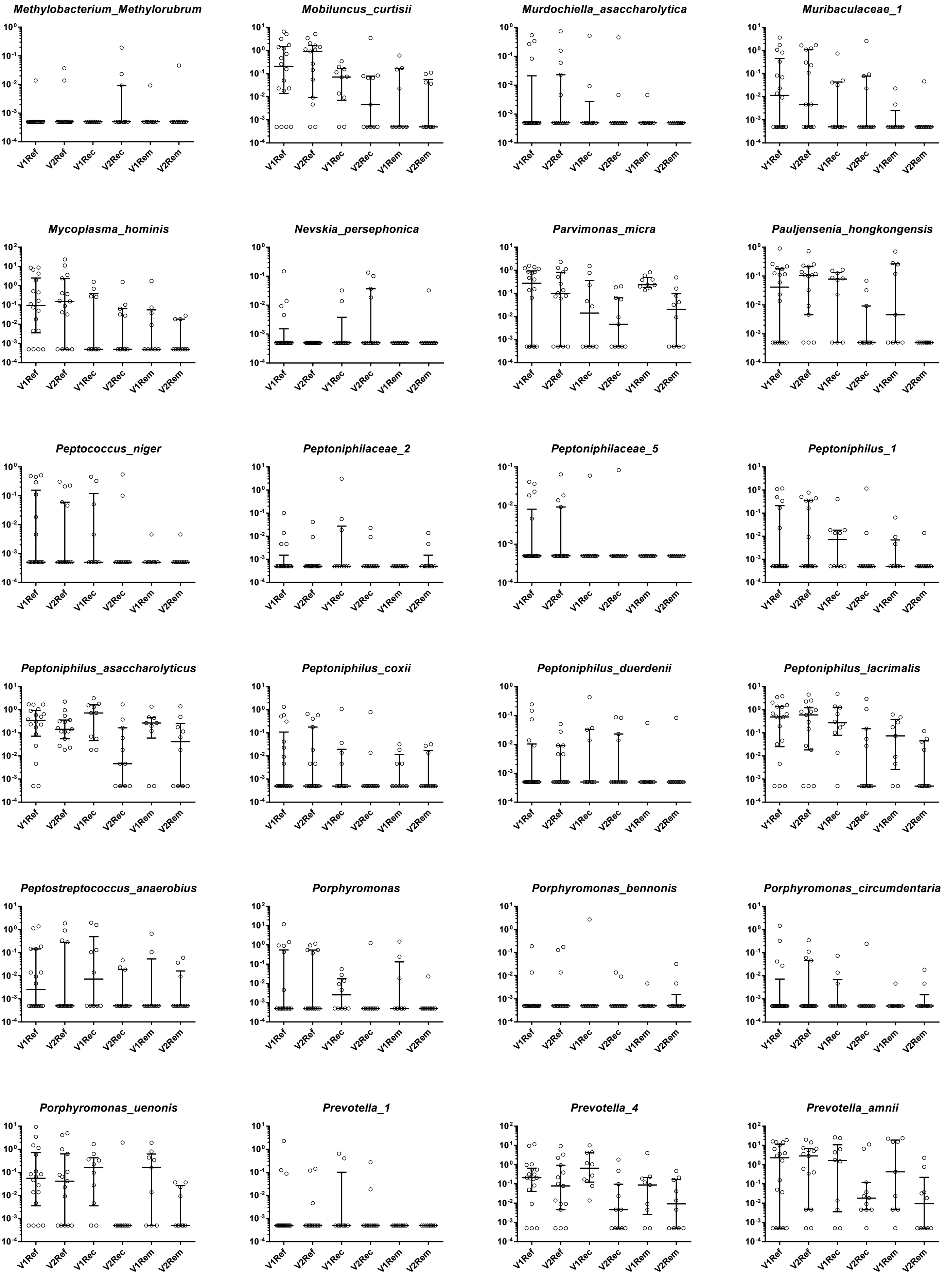
Methylobacterium_Methylorubrum
Mobiluncus_curtisii
Murdochiella_asaccharolytica
Muribaculaceae_1
Mycoplasma_hominis
Nevskia_persephonica
Parvimonas_micra
Pauljensenia_hongkongensis
Peptococcus_niger
Peptoniphilaceae_2
Peptoniphilaceae_5
Peptoniphilus_1
Peptoniphilus_asaccharolyticus
Peptoniphilus_coxii
Peptoniphilus_duerdenii
Peptoniphilus_lacrimalis
Peptostreptococcus_anaerobius
Porphyromonas
Porphyromonas_bennonis
Porphyromonas_circumdentaria
Porphyromonas_uenonis
Prevotella_1
Prevotella_4
Prevotella_amnii
V1Ref
V2Ref
V1Rec
V2Rec
V1Rem
V2Rem

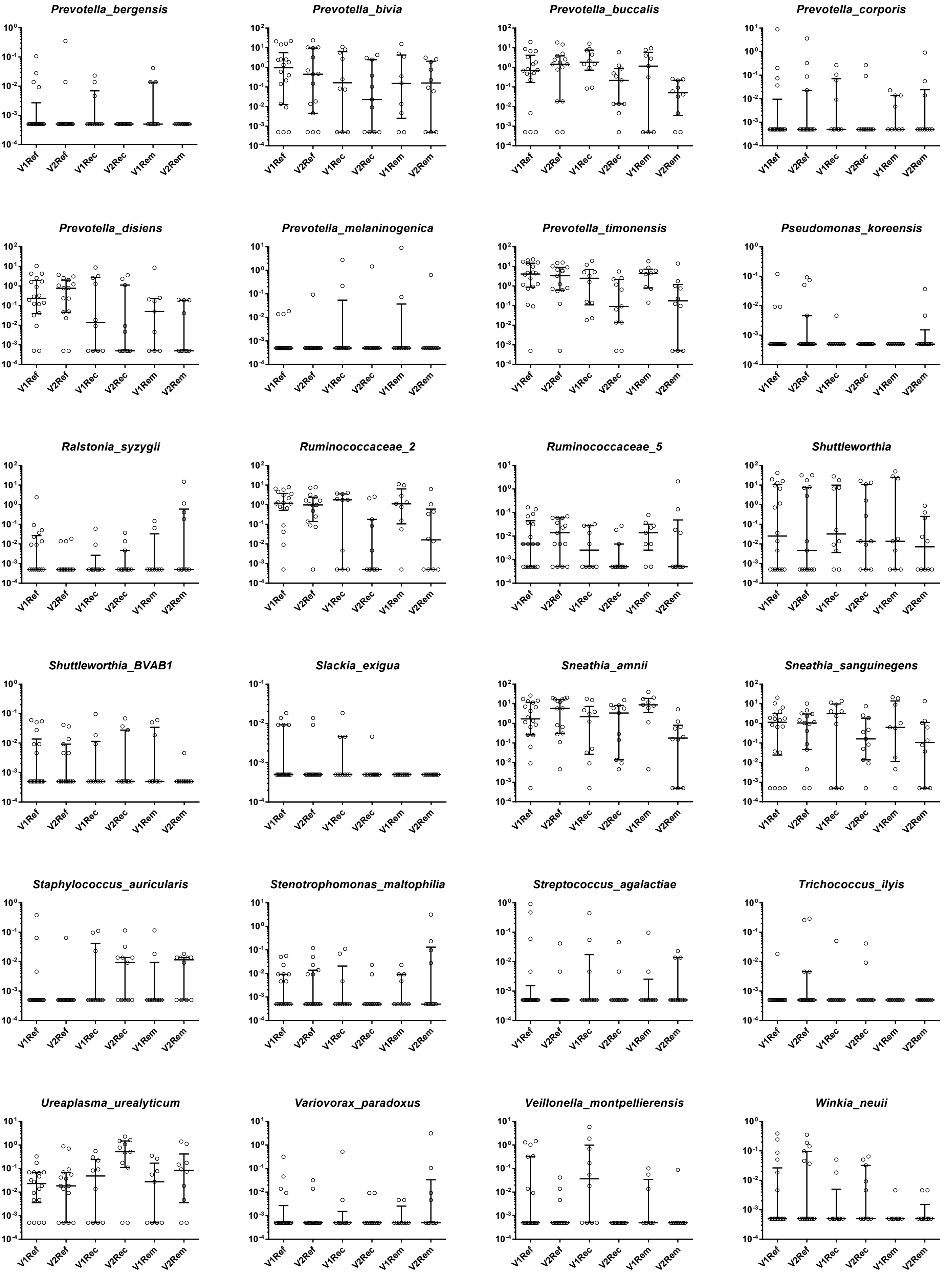
Prevotella_bergensis
Prevotella_bivia
Prevotella_buccalis
Prevotella_corporis
Prevotella_disiens
Prevotella_melaninogenica
Prevotella_timonensis
Pseudomonas_koreensis
Ralstonia_syzygii
Ruminococcaceae_2
Ruminococcaceae_5
Shuttleworthia
Shuttleworthia_BVAB1
Slackia_exigua
Sneathia_amnii
Sneathia_sanguinegens
Staphylococcus_auricularis
Stenotrophomonas_maltophilia
Streptococcus_agalactiae
Trichococcus_ilyis
Ureaplasma_urealyticum
Variovorax_paradoxus
Veillonella_montpellierensis
Winkia_neuii
V1Ref
V2Ref
V1Rec
V2Rec
V1Rem
V2Rem

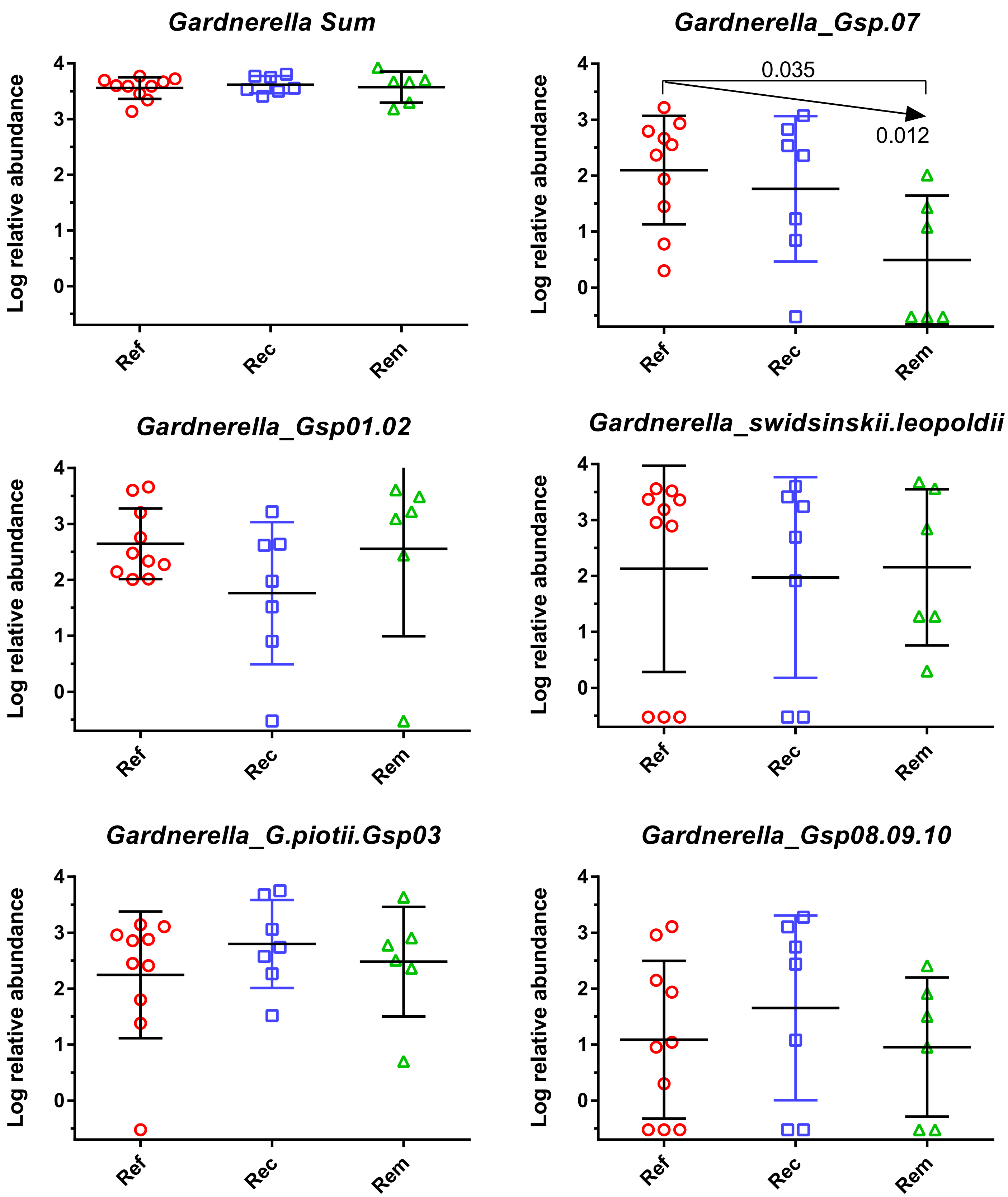
Gardnerella Sum
Log relative abundance
4
3
2
1
0
Ref
Rec
Rem
Gardnerella_Gsp.07
0.035
0.012
Gardnerella_Gsp01.02
Gardnerella_swidsinskii.leopoldii
Gardnerella_G.piotii.Gsp03
Gardnerella_Gsp08.09.10
