# Supplementary material for: Association of key species of vaginal bacteria of recurrent bacterial vaginosis patients before and after oral metronidazole therapy with short- and long-term clinical outcomes
Source: PLoS One. 2022 Jul 28;17(7):e0272012. doi: 10.1371/journal.pone.0272012 (PMC9333308; doi:10.1371/journal.pone.0272012)
Supplement: S4 Fig — (PDF) [file pone.0272012.s004.pdf]

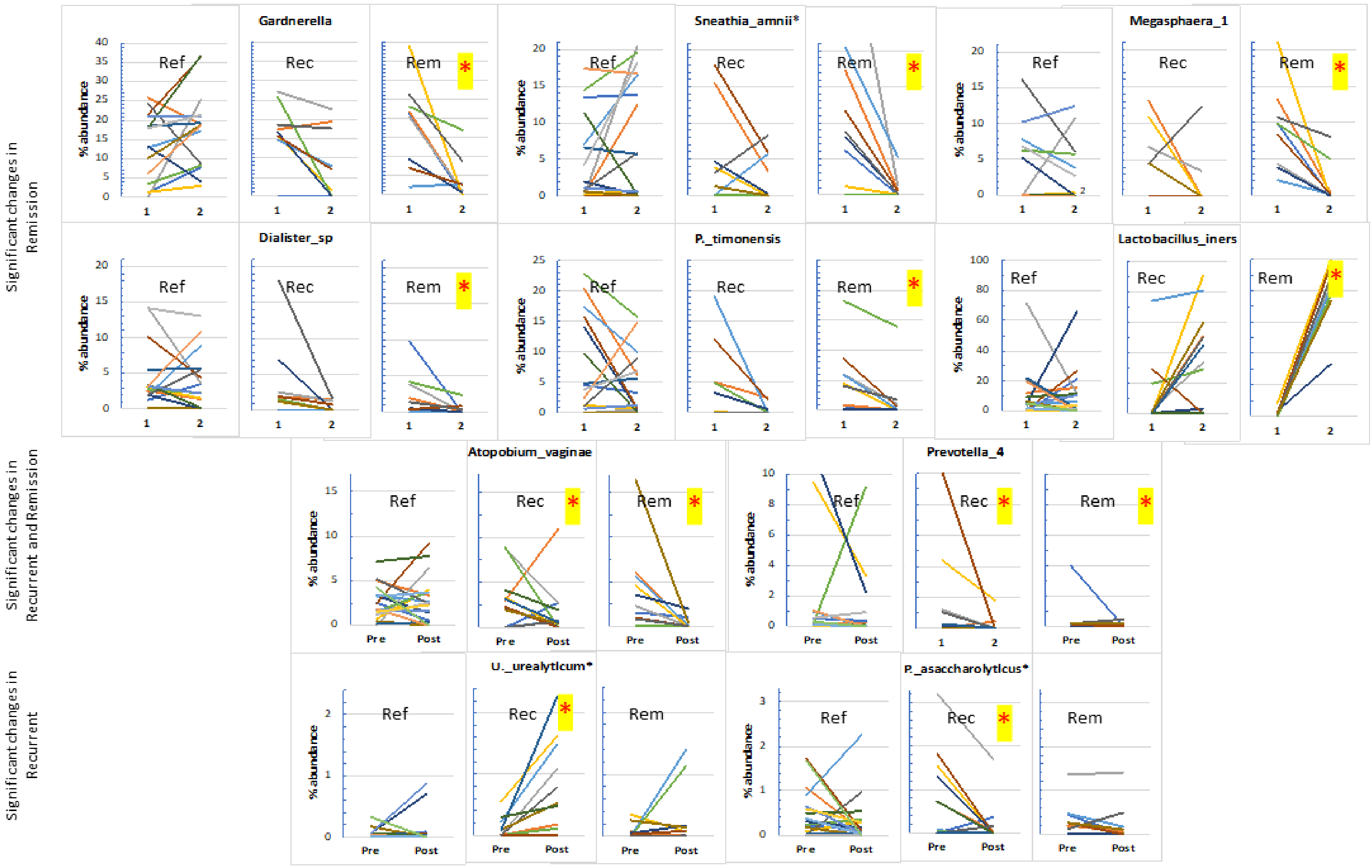

S4 Fig. Percent abundance of species per patient pre- and posttreatment per clinical outcome group. The % of each species of total bacteria at each visit was plotted for 10 core species with the most changes among the recurrent and remission clinical outcome group. The significance of the change was determined by Wilcoxon rank match pairs tests, * indicates $p < 0.05$. Among the 30 core species, 20 showed no significant change in any outcome group.
