# Supplementary material for: Association of key species of vaginal bacteria of recurrent bacterial vaginosis patients before and after oral metronidazole therapy with short- and long-term clinical outcomes
Source: PLoS One. 2022 Jul 28;17(7):e0272012. doi: 10.1371/journal.pone.0272012 (PMC9333308; doi:10.1371/journal.pone.0272012)

S2 Figure. Phylogenetic trees of the 16S rRNA V4 domain of representative OTUs from all pre- and post-treatment vaginal samples aligned to RDP and NCBI databases.

The evolutionary history was inferred by using the Maximum Likelihood method and Tamura-Nei model [1]. The tree with the highest log likelihood (-16801.54) is shown. The percentage of trees in which the associated taxa clustered together is shown next to the branches. Initial tree(s) for the heuristic search were obtained automatically by applying Neighbor-Join and BioNJ algorithms to a matrix of pairwise distances estimated using the Tamura-Nei model, and then selecting the topology with superior log likelihood value. The tree is drawn to scale, with branch lengths measured in the number of substitutions per site (next to the branches). This analysis involved 515 nucleotide sequences. All positions with less than 95% site coverage were eliminated, i.e., fewer than 5% alignment gaps, missing data, and ambiguous bases were allowed at any position (partial deletion option). There were a total of 252 positions in the final dataset. Evolutionary analyses were conducted in MEGA X [2]

1. Tamura K. and Nei M. (1993). Estimation of the number of nucleotide substitutions in the control region of mitochondrial DNA in humans and chimpanzees. Molecular Biology and Evolution 10:512-526.
2. Kumar S., Stecher G., Li M., Knyaz C., and Tamura K. (2018). MEGA X: Molecular Evolutionary Genetics Analysis across computing platforms. Molecular Biology and Evolution 35:1547-1549.

Disclaimer: Although utmost care has been taken to ensure the correctness of the caption, the caption text is provided "as is" without any warranty of any kind. Authors advise the user to carefully check the caption prior to its use for any purpose and report any errors or problems to the authors immediately (www.megasoftware.net). In no event shall the authors and their employers be liable for any damages, including but not limited to special, consequential, or other damages. Authors specifically disclaim all other warranties expressed or implied, including but not limited to the determination of suitability of this caption text for a specific purpose, use, or application.

Figure 2a. Phylogenetic tree of merged MicrobiomeAnalyst input 865 OTUs and all RDP hits.

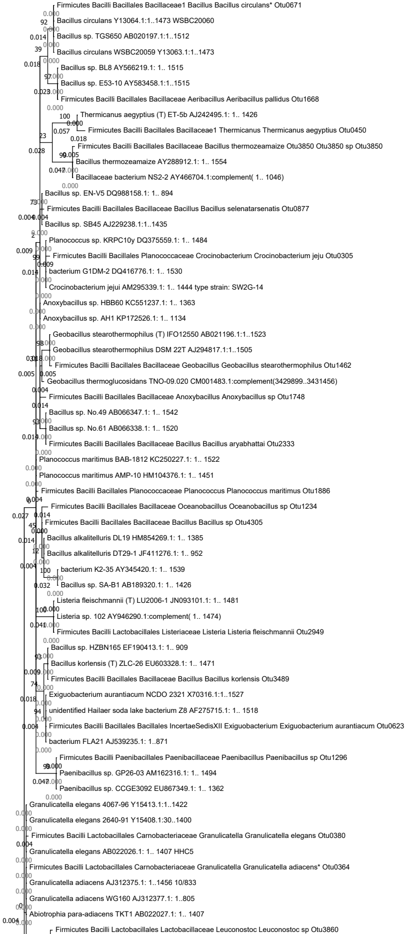

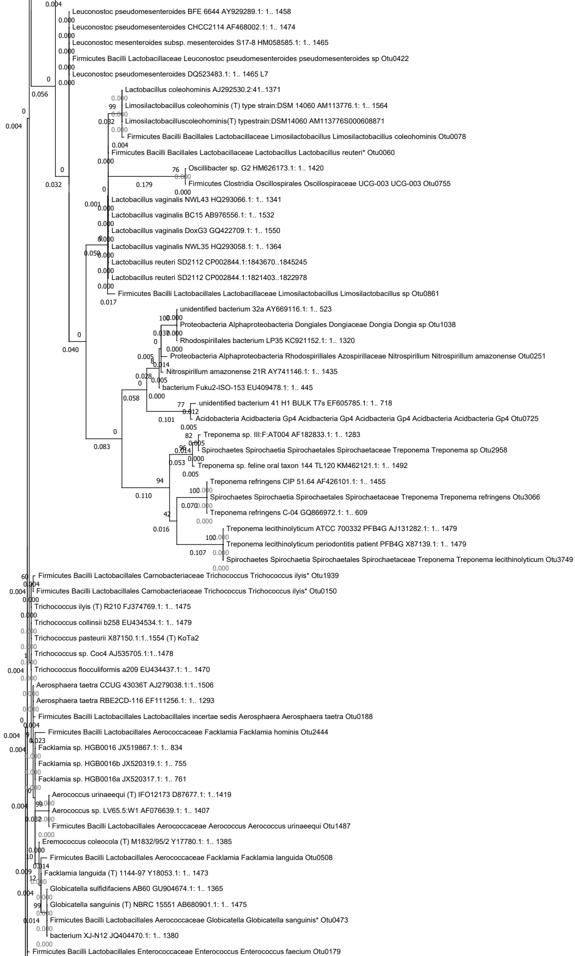

Leuconostoc pseudomesenteroides BFE 6644 AY929289.1: 1.. 1458
Leuconostoc pseudomesenteroides CHCC2114 AF468002.1: 1.. 1474
Leuconostoc mesenteroides subsp. mesenteroides S17-8 HM058585.1: 1.. 1465
Firmicutes Bacilli Lactobacillaceae Leuconostoc pseudomesenteroides pseudomesenteroides sp Otu0422
Leuconostoc pseudomesenteroides DQ523483.1: 1.. 1465 L7
Lactobacillus coleohominis AJ292530.2:41..1371
Limosilactobacillus coleohominis (T) type strain:DSM 14060 AM113776.1: 1.. 1564
Limosilactobacilluscoleohominis(T) typestrain:DSM14060 AM113776S000608871
Firmicutes Bacilli Bacillales Lactobacillaceae Limosilactobacillus Limosilactobacillus coleohominis Otu0078
Firmicutes Bacilli Bacillales Lactobacillaceae Lactobacillus Lactobacillus reuteri* Otu0060
Oscillibacter sp. G2 HM626173.1: 1.. 1420
Firmicutes Clostridia Oscillospirales Oscillospiraceae UCG-003 UCG-003 Otu0755
Lactobacillus vaginalis NWL43 HQ293066.1: 1.. 1341
Lactobacillus vaginalis BC15 AB976556.1: 1.. 1532
Lactobacillus vaginalis DoxG3 GQ422709.1: 1.. 1550
Lactobacillus vaginalis NWL35 HQ293058.1: 1.. 1364
Lactobacillus reuteri SD2112 CP002844.1:1843670..1845245
Lactobacillus reuteri SD2112 CP002844.1:1821403..1822978
Firmicutes Bacilli Lactobacillales Lactobacillaceae Limosilactobacillus Limosilactobacillus sp Otu0861
unidentified bacterium 32a AY669116.1: 1.. 523
Proteobacteria Alphaproteobacteria Dongiales Dongiaceae Dongia Dongia sp Otu1038
Rhodospirillales bacterium LP35 KC921152.1: 1.. 1320
Proteobacteria Alphaproteobacteria Rhodospirillales Azospirillaceae Nitrospirillum Nitrospirillum amazonense Otu0251
Nitrospirillum amazonense 21R AY741146.1: 1.. 1435
bacterium Fuku2-ISO-153 EU409478.1: 1.. 445
unidentified bacterium 41 H1 BULK T7s EF605785.1: 1.. 718
Acidobacteria Acidbacteria Gp4 Acidbacteria Gp4 Acidbacteria Gp4 Acidbacteria Acidbacteria Gp4 Otu0725
Treponema sp. III:F:AT004 AF182833.1: 1.. 1283
Spirochaetes Spirochaetia Spirochaetales Spirochaetaceae Treponema Treponema sp Otu2958
Treponema sp. feline oral taxon 144 TL120 KM462121.1: 1.. 1492
Treponema refringens CIP 51.64 AF426101.1: 1.. 1455
Spirochaetes Spirochaetia Spirochaetales Spirochaetaceae Treponema Treponema refringens Otu3066
Treponema refringens C-04 GQ866972.1: 1.. 609
Treponema lecithinolyticum ATCC 700332 PFB4G AJ131282.1: 1.. 1479
Treponema lecithinolyticum periodontitis patient PFB4G X87139.1: 1.. 1479
Spirochaetes Spirochaetia Spirochaetales Spirochaetaceae Treponema Treponema lecithinolyticum Otu3749
Firmicutes Bacilli Lactobacillales Carnobacteriaceae Trichococcus Trichococcus ilyis* Otu1939
Firmicutes Bacilli Lactobacillales Carnobacteriaceae Trichococcus Trichococcus ilyis* Otu0150
Trichococcus ilyis (T) R210 FJ374769.1: 1.. 1475
Trichococcus collinsii b258 EU434534.1: 1.. 1479
Trichococcus pasteurii X87150.1:1..1554 (T) KoTa2
Trichococcus sp. Coc4 AJ535705.1:1..1478
Trichococcus flocculiformis a209 EU434437.1: 1.. 1470
Aerosphaera taetra CCUG 43036T AJ279038.1:1..1506
Aerosphaera taetra RBE2CD-116 EF111256.1: 1.. 1293
Firmicutes Bacilli Lactobacillales Lactobacillales incertae sedis Aerosphaera Aerosphaera taetra Otu0188
Firmicutes Bacilli Lactobacillales Aerococcaceae Facklamia Facklamia hominis Otu2444
Facklamia sp. HGB0016 JX519867.1: 1.. 834
Facklamia sp. HGB0016b JX520319.1: 1.. 755
Facklamia sp. HGB0016a JX520317.1: 1.. 761
Aerococcus urinaeequi (T) IFO12173 D87677.1: 1..1419
Aerococcus sp. LV65.5:W1 AF076639.1: 1.. 1407
Firmicutes Bacilli Lactobacillales Aerococcaceae Aerococcus Aerococcus urinaeequi Otu1487
Eremococcus coleocola (T) M1832/95/2 Y17780.1: 1.. 1385
Firmicutes Bacilli Lactobacillales Aerococcaceae Facklamia Facklamia languida Otu0508
Facklamia languida (T) 1144-97 Y18053.1: 1.. 1473
Globicatella sulfidifaciens AB60 GU904674.1: 1.. 1365
Globicatella sanguinis (T) NBRC 15551 AB680901.1: 1.. 1475
Firmicutes Bacilli Lactobacillales Aerococcaceae Globicatella Globicatella sanguinis* Otu0473
bacterium XJ-N12 JQ404470.1: 1.. 1380
Firmicutes Bacilli Lactobacillales Enterococcaceae Enterococcus Enterococcus faecium Otu0179

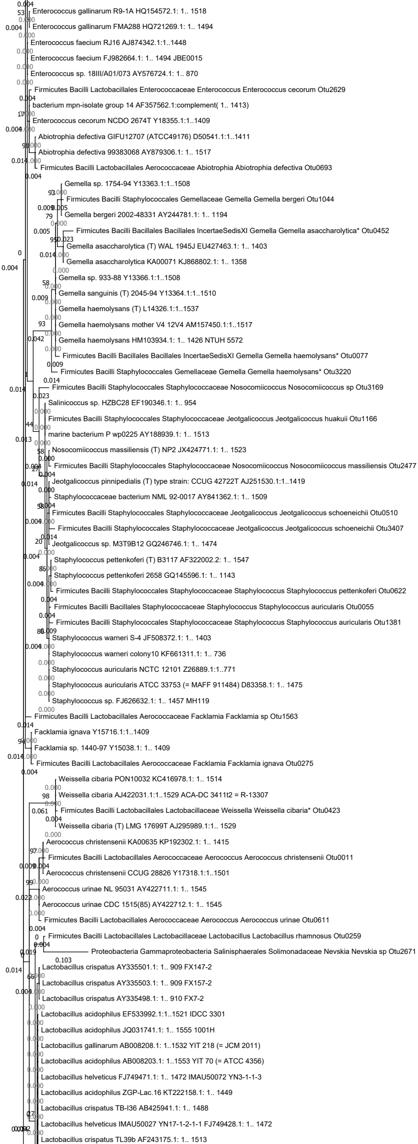

Enterococcus gallinarum R9-1A HQ154572.1: 1.. 1518
Enterococcus gallinarum FMA288 HQ721269.1: 1.. 1494
Enterococcus faecium RJ16 AJ874342.1:1..1448
Enterococcus faecium FJ982664.1: 1.. 1494 JBE0015
Enterococcus sp. 18III/A01/073 AY576724.1: 1.. 870
Firmicutes Bacilli Lactobacillales Enterococcaceae Enterococcus Enterococcus cecorum Otu2629
bacterium mpn-isolate group 14 AF357562.1:complement(1.. 1413)
Enterococcus cecorum NCDO 2674T Y18355.1:1..1409
Abiotrophia defectiva GIFU12707 (ATCC49176) D50541.1:1..1411
Abiotrophia defectiva 99383068 AY879306.1: 1.. 1517
Firmicutes Bacilli Lactobacillales Aerococcaceae Abiotrophia Abiotrophia defectiva Otu0693
Gemella sp. 1754-94 Y13363.1:1..1508
Firmicutes Bacilli Staphylococcales Gemellaceae Gemella Gemella bergeri Otu1044
Gemella bergeri 2002-48331 AY244781.1: 1.. 1194
Firmicutes Bacilli Bacillales Bacillales IncertaeSedisXI Gemella Gemella asaccharolytica* Otu0452
Gemella asaccharolytica (T) WAL 1945J EU427463.1: 1.. 1403
Gemella asaccharolytica KA00071 KJ868802.1: 1.. 1358
Gemella sp. 933-88 Y13366.1:1..1508
Gemella sanguinis (T) 2045-94 Y13364.1:1..1510
Gemella haemolysans (T) L14326.1:1..1537
Gemella haemolysans mother V4 12V4 AM157450.1:1..1517
Gemella haemolysans HM103934.1: 1.. 1426 NTUH 5572
Firmicutes Bacilli Bacillales Bacillales IncertaeSedisXI Gemella Gemella haemolysans* Otu0077
Firmicutes Bacilli Staphylococcales Gemellaceae Gemella Gemella haemolysans* Otu3220
Firmicutes Bacilli Staphylococcales Staphylococcaceae Nosocomiicoccus Nosocomiicoccus sp Otu3169
Salinicoccus sp. HZBC28 EF190346.1: 1.. 954
Firmicutes Bacilli Staphylococcales Staphylococcaceae Jeotgalicoccus Jeotgalicoccus huakuii Otu1166
marine bacterium P wp0225 AY188939.1: 1.. 1513
Nosocomiicoccus massiliensis (T) NP2 JX424771.1: 1.. 1523
Firmicutes Bacilli Staphylococcales Staphylococcaceae Nosocomiicoccus Nosocomiicoccus massiliensis Otu2477
Jeotgalicoccus pinnipedialis (T) type strain: CCUG 42722T AJ251530.1:1..1419
Staphylococcaceae bacterium NML 92-0017 AY841362.1: 1.. 1509
Firmicutes Bacilli Staphylococcales Staphylococcaceae Jeotgalicoccus Jeotgalicoccus schoeneichii Otu0510
Firmicutes Bacilli Staphylococcales Staphylococcaceae Jeotgalicoccus Jeotgalicoccus schoeneichii Otu3407
Jeotgalicoccus sp. M3T9B12 GQ246746.1: 1.. 1474
Staphylococcus pettenkoferi (T) B3117 AF322002.2: 1.. 1547
Staphylococcus pettenkoferi 2658 GQ145596.1: 1.. 1143
Firmicutes Bacilli Staphylococcales Staphylococcaceae Staphylococcus Staphylococcus pettenkoferi Otu0622
Firmicutes Bacilli Bacillales Staphylococcaceae Staphylococcus Staphylococcus auricularis Otu0055
Firmicutes Bacilli Staphylococcales Staphylococcaceae Staphylococcus Staphylococcus auricularis Otu1381
Staphylococcus warneri S-4 JF508372.1: 1.. 1403
Staphylococcus warneri colony10 KF661311.1: 1.. 736
Staphylococcus auricularis NCTC 12101 Z26889.1:1..771
Staphylococcus auricularis ATCC 33753 (= MAFF 911484) D83358.1: 1.. 1475
Staphylococcus sp. FJ626632.1: 1.. 1457 MH119
Firmicutes Bacilli Lactobacillales Aerococcaceae Facklamia Facklamia sp Otu1563
Facklamia ignava Y15716.1:1..1409
Facklamia sp. 1440-97 Y15038.1: 1.. 1409
Firmicutes Bacilli Lactobacillales Aerococcaceae Facklamia Facklamia ignava Otu0275
Weissella cibaria PON10032 KC416978.1: 1.. 1514
Weissella cibaria AJ422031.1:1..1529 ACA-DC 3411t2 = R-13307
Firmicutes Bacilli Lactobacillales Lactobacillaceae Weissella Weissella cibaria* Otu0423
Weissella cibaria (T) LMG 17699T AJ295989.1:1.. 1529
Aerococcus christensenii KA00635 KP192302.1: 1.. 1415
Firmicutes Bacilli Lactobacillales Aerococcaceae Aerococcus Aerococcus christensenii Otu0011
Aerococcus christensenii CCUG 28826 Y17318.1:1..1501
Aerococcus urinae NL 95031 AY422711.1: 1.. 1545
Aerococcus urinae CDC 1515(85) AY422712.1: 1.. 1545
Firmicutes Bacilli Lactobacillales Aerococcaceae Aerococcus Aerococcus urinae Otu0611
Firmicutes Bacilli Lactobacillales Lactobacillaceae Lactobacillus Lactobacillus rhamnosus Otu0259
Proteobacteria Gammaproteobacteria Salinisphaerales Solimonadaceae Nevskia Nevskia sp Otu2671
Lactobacillus crispatus AY335501.1: 1.. 909 FX147-2
Lactobacillus crispatus AY335503.1: 1.. 909 FX157-2
Lactobacillus crispatus AY335498.1: 1.. 910 FX7-2
Lactobacillus acidophilus EF533992.1:1..1521 IDCC 3301
Lactobacillus acidophilus JQ031741.1: 1.. 1555 1001H
Lactobacillus gallinarum AB008208.1: 1..1532 YIT 218 (= JCM 2011)
Lactobacillus acidophilus AB008203.1: 1..1553 YIT 70 (= ATCC 4356)
Lactobacillus helveticus FJ749471.1: 1.. 1472 IMAU50072 YN3-1-1-3
Lactobacillus acidophilus ZGP-Lac.16 KT222158.1: 1.. 1449
Lactobacillus crispatus TB-I36 AB425941.1: 1.. 1488
Lactobacillus helveticus IMAU50027 YN17-1-2-1-1 FJ749428.1: 1.. 1472
Lactobacillus crispatus TL39b AF243175.1: 1.. 1513

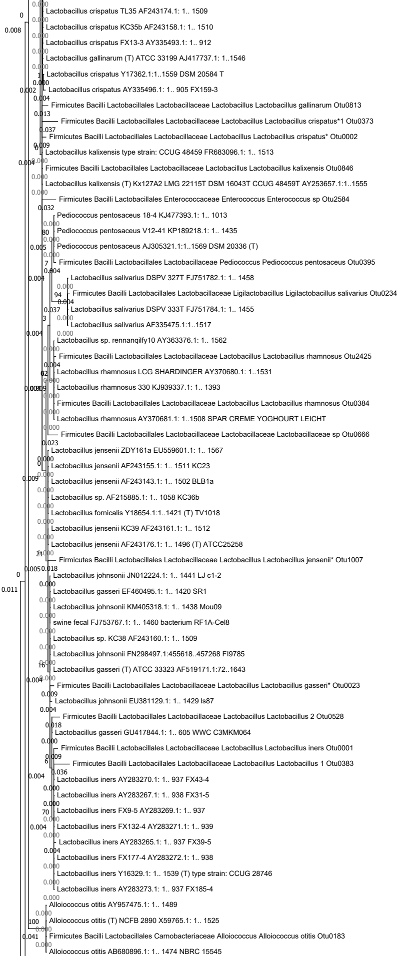
Lactobacillus crispatus TL35 AF243174.1: 1.. 1509
Lactobacillus crispatus KC35b AF243158.1: 1.. 1510
Lactobacillus crispatus FX13-3 AY335493.1: 1.. 912
Lactobacillus gallinarum (T) ATCC 33199 AJ417737.1: 1..1546
Lactobacillus crispatus Y17362.1:1..1559 DSM 20584 T
Lactobacillus crispatus AY335496.1: 1.. 905 FX159-3
Firmicutes Bacilli Lactobacillales Lactobacillaceae Lactobacillus Lactobacillus gallinarum Otu0813
Firmicutes Bacilli Lactobacillales Lactobacillaceae Lactobacillus Lactobacillus crispatus*1 Otu0373
Firmicutes Bacilli Lactobacillales Lactobacillaceae Lactobacillus Lactobacillus crispatus* Otu0002
Lactobacillus kalixensis type strain: CCUG 48459 FR683096.1: 1.. 1513
Firmicutes Bacilli Lactobacillales Lactobacillaceae Lactobacillus Lactobacillus kalixensis Otu0846
Lactobacillus kalixensis (T) Kx127A2 LMG 22115T DSM 16043T CCUG 48459T AY253657.1:1..1555
Firmicutes Bacilli Lactobacillales Enterococcaceae Enterococcus Enterococcus sp Otu2584
Pediococcus pentosaceus 18-4 KJ477393.1: 1.. 1013
Pediococcus pentosaceus V12-41 KP189218.1: 1.. 1435
Pediococcus pentosaceus AJ305321.1:1..1569 DSM 20336 (T)
Firmicutes Bacilli Lactobacillales Lactobacillaceae Pediococcus Pediococcus pentosaceus Otu0395
Lactobacillus salivarius DSPV 327T FJ751782.1: 1.. 1458
Firmicutes Bacilli Lactobacillales Lactobacillaceae Ligilactobacillus Ligilactobacillus salivarius Otu0234
Lactobacillus salivarius DSPV 333T FJ751784.1: 1.. 1455
Lactobacillus salivarius AF335475.1:1..1517
Lactobacillus sp. rennanqilfy10 AY363376.1: 1.. 1562
Firmicutes Bacilli Lactobacillales Lactobacillaceae Lactobacillus Lactobacillus rhamnosus Otu2425
Lactobacillus rhamnosus LCG SHARDINGER AY370680.1: 1..1531
Lactobacillus rhamnosus 330 KJ939337.1: 1.. 1393
Firmicutes Bacilli Lactobacillales Lactobacillaceae Lactobacillus Lactobacillus rhamnosus Otu0384
Lactobacillus rhamnosus AY370681.1: 1..1508 SPAR CREME YOGHOURT LEICHT
Firmicutes Bacilli Lactobacillales Lactobacillaceae Lactobacillaceae Lactobacillaceae sp Otu0666
Lactobacillus jensenii ZDY161a EU559601.1: 1.. 1567
Lactobacillus jensenii AF243155.1: 1.. 1511 KC23
Lactobacillus jensenii AF243143.1: 1.. 1502 BLB1a
Lactobacillus sp. AF215885.1: 1.. 1058 KC36b
Lactobacillus fornicalis Y18654.1:1..1421 (T) TV1018
Lactobacillus jensenii KC39 AF243161.1: 1.. 1512
Lactobacillus jensenii AF243176.1: 1.. 1496 (T) ATCC25258
Firmicutes Bacilli Lactobacillales Lactobacillaceae Lactobacillus Lactobacillus jensenii* Otu1007
Lactobacillus johnsonii JN012224.1: 1.. 1441 LJ c1-2
Lactobacillus gasseri EF460495.1: 1.. 1420 SR1
Lactobacillus johnsonii KM405318.1: 1.. 1438 Mou09
swine fecal FJ753767.1: 1.. 1460 bacterium RF1A-Cel8
Lactobacillus sp. KC38 AF243160.1: 1.. 1509
Lactobacillus johnsonii FN298497.1:455618..457268 FI9785
Lactobacillus gasseri (T) ATCC 33323 AF519171.1:72..1643
Firmicutes Bacilli Lactobacillales Lactobacillaceae Lactobacillus Lactobacillus gasseri* Otu0023
Lactobacillus johnsonii EU381129.1: 1.. 1429 ls87
Firmicutes Bacilli Lactobacillales Lactobacillaceae Lactobacillus Lactobacillus 2 Otu0528
Lactobacillus gasseri GU417844.1: 1.. 605 WWC C3MKM064
Firmicutes Bacilli Lactobacillales Lactobacillaceae Lactobacillus Lactobacillus iners Otu0001
Firmicutes Bacilli Lactobacillales Lactobacillaceae Lactobacillus Lactobacillus 1 Otu0383
Lactobacillus iners AY283270.1: 1.. 937 FX43-4
Lactobacillus iners AY283267.1: 1.. 938 FX31-5
Lactobacillus iners FX9-5 AY283269.1: 1.. 937
Lactobacillus iners FX132-4 AY283271.1: 1.. 939
Lactobacillus iners AY283265.1: 1.. 937 FX39-5
Lactobacillus iners FX177-4 AY283272.1: 1.. 938
Lactobacillus iners Y16329.1: 1.. 1539 (T) type strain: CCUG 28746
Lactobacillus iners AY283273.1: 1.. 937 FX185-4
Alloiococcus otitis AY957475.1: 1.. 1489
Alloiococcus otitis (T) NCFB 2890 X59765.1: 1.. 1525
Firmicutes Bacilli Lactobacillales Carnobacteriaceae Alloiococcus Alloiococcus otitis Otu0183
Alloiococcus otitis AB680896.1: 1.. 1474 NBRC 15545

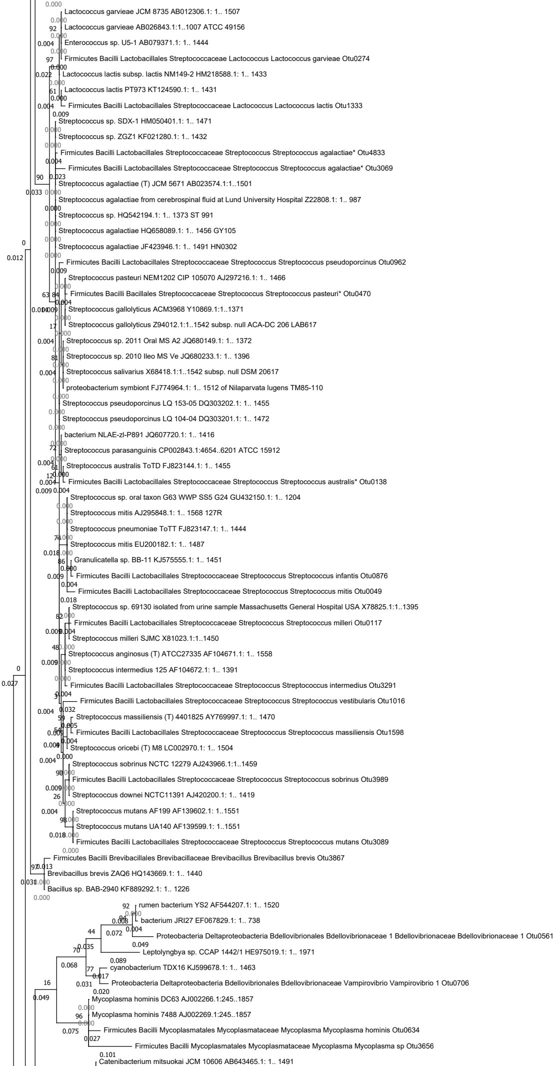
Lactococcus garvieae JCM 8735 AB012306.1: 1.. 1507
Lactococcus garvieae AB026843.1:1..1007 ATCC 49156
Enterococcus sp. U5-1 AB079371.1: 1.. 1444
Firmicutes Bacilli Lactobacillales Streptococcaceae Lactococcus Lactococcus garvieae Otu0274
Lactococcus lactis subsp. lactis NM149-2 HM218588.1: 1.. 1433
Lactococcus lactis PT973 KT124590.1: 1.. 1431
Firmicutes Bacilli Lactobacillales Streptococcaceae Lactococcus Lactococcus lactis Otu1333
Streptococcus sp. SDX-1 HM050401.1: 1.. 1471
Streptococcus sp. ZGZ1 KF021280.1: 1.. 1432
Firmicutes Bacilli Lactobacillales Streptococcaceae Streptococcus Streptococcus agalactiae* Otu4833
Firmicutes Bacilli Lactobacillales Streptococcaceae Streptococcus Streptococcus agalactiae* Otu3069
Streptococcus agalactiae (T) JCM 5671 AB023574.1:1..1501
Streptococcus agalactiae from cerebrospinal fluid at Lund University Hospital Z22808.1: 1.. 987
Streptococcus sp. HQ542194.1: 1.. 1373 ST 991
Streptococcus agalactiae HQ658089.1: 1.. 1456 GY105
Streptococcus agalactiae JF423946.1: 1.. 1491 HN0302
Firmicutes Bacilli Lactobacillales Streptococcaceae Streptococcus Streptococcus pseudoporcinus Otu0962
Streptococcus pasteuri NEM1202 CIP 105070 AJ297216.1: 1.. 1466
Firmicutes Bacilli Bacillales Streptococcaceae Streptococcus Streptococcus pasteuri* Otu0470
Streptococcus gallolyticus ACM3968 Y10869.1:1..1371
Streptococcus gallolyticus Z94012.1:1..1542 subsp. null ACA-DC 206 LAB617
Streptococcus sp. 2011 Oral MS A2 JQ680149.1: 1.. 1372
Streptococcus sp. 2010 Ileo MS Ve JQ680233.1: 1.. 1396
Streptococcus salivarius X68418.1:1..1542 subsp. null DSM 20617
proteobacterium symbiont FJ774964.1: 1.. 1512 of Nilaparvata lugens TM85-110
Streptococcus pseudoporcinus LQ 153-05 DQ303202.1: 1.. 1455
Streptococcus pseudoporcinus LQ 104-04 DQ303201.1: 1.. 1472
bacterium NLAE-zl-P891 JQ607720.1: 1.. 1416
Streptococcus parasanguinis CP002843.1:4654..6201 ATCC 15912
Streptococcus australis ToTD FJ823144.1: 1.. 1455
Firmicutes Bacilli Lactobacillales Streptococcaceae Streptococcus Streptococcus australis* Otu0138
Streptococcus sp. oral taxon G63 WWP SS5 G24 GU432150.1: 1.. 1204
Streptococcus mitis AJ295848.1: 1.. 1568 127R
Streptococcus pneumoniae ToTT FJ823147.1: 1.. 1444
Streptococcus mitis EU200182.1: 1.. 1487
Granulicatella sp. BB-11 KJ575555.1: 1.. 1451
Firmicutes Bacilli Lactobacillales Streptococcaceae Streptococcus Streptococcus infantis Otu0876
Firmicutes Bacilli Lactobacillales Streptococcaceae Streptococcus Streptococcus mitis Otu0049
Streptococcus sp. 69130 isolated from urine sample Massachusetts General Hospital USA X78825.1:1..1395
Firmicutes Bacilli Lactobacillales Streptococcaceae Streptococcus Streptococcus milleri Otu0117
Streptococcus milleri SJMC X81023.1:1..1450
Streptococcus anginosus (T) ATCC27335 AF104671.1: 1.. 1558
Streptococcus intermedius 125 AF104672.1: 1.. 1391
Firmicutes Bacilli Lactobacillales Streptococcaceae Streptococcus Streptococcus intermedius Otu3291
Firmicutes Bacilli Lactobacillales Streptococcaceae Streptococcus Streptococcus vestibularis Otu1016
Streptococcus massiliensis (T) 4401825 AY769997.1: 1.. 1470
Firmicutes Bacilli Lactobacillales Streptococcaceae Streptococcus Streptococcus massiliensis Otu1598
Streptococcus oricebi (T) M8 LC002970.1: 1.. 1504
Streptococcus sobrinus NCTC 12279 AJ243966.1:1..1459
Firmicutes Bacilli Lactobacillales Streptococcaceae Streptococcus Streptococcus sobrinus Otu3989
Streptococcus downei NCTC11391 AJ420200.1: 1.. 1419
Streptococcus mutans AF199 AF139602.1: 1..1551
Streptococcus mutans UA140 AF139599.1: 1..1551
Firmicutes Bacilli Lactobacillales Streptococcaceae Streptococcus Streptococcus mutans Otu3089
Firmicutes Bacilli Brevibacillales Brevibacillaceae Brevibacillus Brevibacillus brevis Otu3867
Brevibacillus brevis ZAQ6 HQ143669.1: 1.. 1440
Bacillus sp. BAB-2940 KF889292.1: 1.. 1226
rumen bacterium YS2 AF544207.1: 1.. 1520
bacterium JRI27 EF067829.1: 1.. 738
Proteobacteria Deltaproteobacteria Bdellovibrionales Bdellovibrionaceae 1 Bdellovibrionaceae Bdellovibrionaceae 1 Otu0561
Leptolyngbya sp. CCAP 1442/1 HE975019.1: 1.. 1971
cyanobacterium TDX16 KJ599678.1: 1.. 1463
Proteobacteria Deltaproteobacteria Bdellovibrionales Bdellovibrionaceae Vampirovibrio Vampirovibrio 1 Otu0706
Mycoplasma hominis DC63 AJ002266.1:245..1857
Mycoplasma hominis 7488 AJ002269.1:245..1857
Firmicutes Bacilli Mycoplasmatales Mycoplasmataceae Mycoplasma Mycoplasma hominis Otu0634
Firmicutes Bacilli Mycoplasmatales Mycoplasmataceae Mycoplasma Mycoplasma sp Otu3656
Catenibacterium mitsuokai JCM 10606 AB643465.1: 1.. 1491

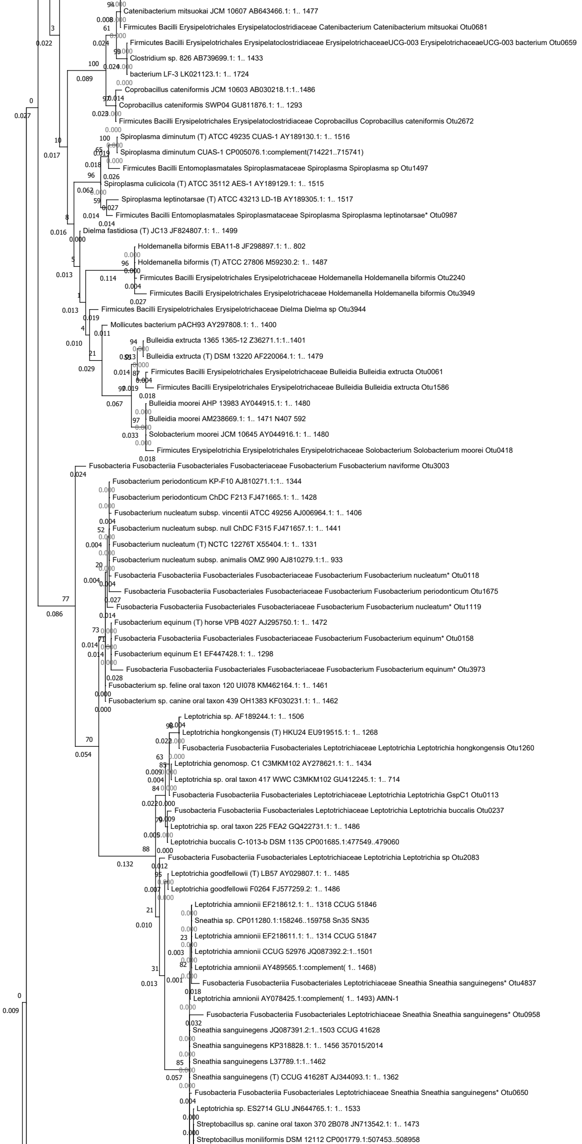
Catenibacterium mitsuokai JCM 10607 AB643466.1: 1.. 1477
Firmicutes Bacilli Erysipelotrichales Erysipelatoclostridiaceae Catenibacterium Catenibacterium mitsuokai Otu0681
Firmicutes Bacilli Erysipelotrichales Erysipelatoclostridiaceae ErysipelotrichaceaeUCG-003 ErysipelotrichaceaeUCG-003 bacterium Otu0659
Clostridium sp. 826 AB739699.1: 1.. 1433
bacterium LF-3 LK021123.1: 1.. 1724
Coprobacillus cateniformis JCM 10603 AB030218.1:1..1486
Coprobacillus cateniformis SWP04 GU811876.1: 1.. 1293
Firmicutes Bacilli Erysipelotrichales Erysipelatoclostridiaceae Coprobacillus Coprobacillus cateniformis Otu2672
Spiroplasma diminutum (T) ATCC 49235 CUAS-1 AY189130.1: 1.. 1516
Spiroplasma diminutum CUAS-1 CP005076.1:complement(714221..715741)
Firmicutes Bacilli Entomoplasmatales Spiroplasmataceae Spiroplasma Spiroplasma sp Otu1497
Spiroplasma culicicola (T) ATCC 35112 AES-1 AY189129.1: 1.. 1515
Spiroplasma leptinotarsae (T) ATCC 43213 LD-1B AY189305.1: 1.. 1517
Firmicutes Bacilli Entomoplasmatales Spiroplasmataceae Spiroplasma Spiroplasma leptinotarsae* Otu0987
Dielma fastidiosa (T) JC13 JF824807.1: 1.. 1499
Holdemanella biformis EBA11-8 JF298897.1: 1.. 802
Holdemanella biformis (T) ATCC 27806 M59230.2: 1.. 1487
Firmicutes Bacilli Erysipelotrichales Erysipelotrichaceae Holdemanella Holdemanella biformis Otu2240
Firmicutes Bacilli Erysipelotrichales Erysipelotrichaceae Holdemanella Holdemanella biformis Otu3949
Firmicutes Bacilli Erysipelotrichales Erysipelotrichaceae Dielma Dielma sp Otu3944
Mollicutes bacterium pACH93 AY297808.1: 1.. 1400
Bulleidia extructa 1365 1365-12 Z36271.1:1..1401
Bulleidia extructa (T) DSM 13220 AF220064.1: 1.. 1479
Firmicutes Bacilli Erysipelotrichales Erysipelotrichaceae Bulleidia Bulleidia extructa Otu0061
Firmicutes Bacilli Erysipelotrichales Erysipelotrichaceae Bulleidia Bulleidia extructa Otu1586
Bulleidia moorei AHP 13983 AY044915.1: 1.. 1480
Bulleidia moorei AM238669.1: 1.. 1471 N407 592
Solobacterium moorei JCM 10645 AY044916.1: 1.. 1480
Firmicutes Erysipelotrichia Erysipelotrichales Erysipelotrichaceae Solobacterium Solobacterium moorei Otu0418
Fusobacteria Fusobacteriia Fusobacteriales Fusobacteriaceae Fusobacterium Fusobacterium naviforme Otu3003
Fusobacterium periodonticum KP-F10 AJ810271.1:1.. 1344
Fusobacterium periodonticum ChDC F213 FJ471665.1: 1.. 1428
Fusobacterium nucleatum subsp. vincentii ATCC 49256 AJ006964.1: 1.. 1406
Fusobacterium nucleatum subsp. null ChDC F315 FJ471657.1: 1.. 1441
Fusobacterium nucleatum (T) NCTC 12276T X55404.1: 1.. 1331
Fusobacterium nucleatum subsp. animalis OMZ 990 AJ810279.1:1.. 933
Fusobacteria Fusobacteriia Fusobacteriales Fusobacteriaceae Fusobacterium Fusobacterium nucleatum* Otu0118
Fusobacteria Fusobacteriia Fusobacteriales Fusobacteriaceae Fusobacterium Fusobacterium periodonticum Otu1675
Fusobacteria Fusobacteriia Fusobacteriales Fusobacteriaceae Fusobacterium Fusobacterium nucleatum* Otu1119
Fusobacterium equinum (T) horse VPB 4027 AJ295750.1: 1.. 1472
Fusobacteria Fusobacteriia Fusobacteriales Fusobacteriaceae Fusobacterium Fusobacterium equinum* Otu0158
Fusobacterium equinum E1 EF447428.1: 1.. 1298
Fusobacteria Fusobacteriia Fusobacteriales Fusobacteriaceae Fusobacterium Fusobacterium equinum* Otu3973
Fusobacterium sp. feline oral taxon 120 UI078 KM462164.1: 1.. 1461
Fusobacterium sp. canine oral taxon 439 OH1383 KF030231.1: 1.. 1462
Leptotrichia sp. AF189244.1: 1.. 1506
Leptotrichia hongkongensis (T) HKU24 EU919515.1: 1.. 1268
Fusobacteria Fusobacteriia Fusobacteriales Leptotrichiaceae Leptotrichia Leptotrichia hongkongensis Otu1260
Leptotrichia genomosp. C1 C3MKM102 AY278621.1: 1.. 1434
Leptotrichia sp. oral taxon 417 WWC C3MKM102 GU412245.1: 1.. 714
Fusobacteria Fusobacteriia Fusobacteriales Leptotrichiaceae Leptotrichia Leptotrichia GspC1 Otu0113
Fusobacteria Fusobacteriia Fusobacteriales Leptotrichiaceae Leptotrichia Leptotrichia buccalis Otu0237
Leptotrichia sp. oral taxon 225 FEA2 GQ422731.1: 1.. 1486
Leptotrichia buccalis C-1013-b DSM 1135 CP001685.1:477549..479060
Fusobacteria Fusobacteriia Fusobacteriales Leptotrichiaceae Leptotrichia Leptotrichia sp Otu2083
Leptotrichia goodfellowii (T) LB57 AY029807.1: 1.. 1485
Leptotrichia goodfellowii F0264 FJ577259.2: 1.. 1486
Leptotrichia amnionii EF218612.1: 1.. 1318 CCUG 51846
Sneathia sp. CP011280.1:158246..159758 Sn35 SN35
Leptotrichia amnionii EF218611.1: 1.. 1314 CCUG 51847
Leptotrichia amnionii CCUG 52976 JQ087392.2:1..1501
Leptotrichia amnionii AY489565.1:complement(1.. 1468)
Fusobacteria Fusobacteriia Fusobacteriales Leptotrichiaceae Sneathia Sneathia sanguinegens* Otu4837
Leptotrichia amnionii AY078425.1:complement(1.. 1493) AMN-1
Fusobacteria Fusobacteriia Fusobacteriales Leptotrichiaceae Sneathia Sneathia sanguinegens* Otu0958
Sneathia sanguinegens JQ087391.2:1..1503 CCUG 41628
Sneathia sanguinegens KP318828.1: 1.. 1456 357015/2014
Sneathia sanguinegens L37789.1:1..1462
Sneathia sanguinegens (T) CCUG 41628T AJ344093.1: 1.. 1362
Fusobacteria Fusobacteriia Fusobacteriales Leptotrichiaceae Sneathia Sneathia sanguinegens* Otu0650
Leptotrichia sp. ES2714 GLU JN644765.1: 1.. 1533
Streptobacillus sp. canine oral taxon 370 2B078 JN713542.1: 1.. 1473
Streptobacillus moniliformis DSM 12112 CP001779.1:507453..508958

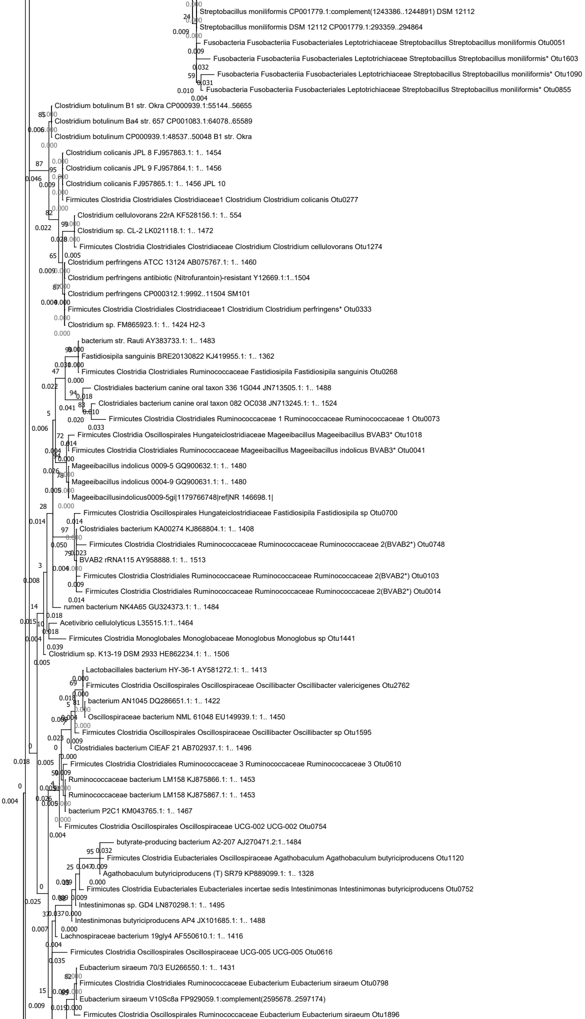
Streptobacillus moniliformis CP001779.1:complement(1243386..1244891) DSM 12112
Streptobacillus moniliformis DSM 12112 CP001779.1:293359..294864
Fusobacteria Fusobacteriia Fusobacteriales Leptotrichiaceae Streptobacillus Streptobacillus moniliformis Otu0051
Fusobacteria Fusobacteriia Fusobacteriales Leptotrichiaceae Streptobacillus Streptobacillus moniliformis* Otu1603
Fusobacteria Fusobacteriia Fusobacteriales Leptotrichiaceae Streptobacillus Streptobacillus moniliformis* Otu1090
Fusobacteria Fusobacteriia Fusobacteriales Leptotrichiaceae Streptobacillus Streptobacillus moniliformis* Otu0855
Clostridium botulinum B1 str. Okra CP000939.1:55144..56655
Clostridium botulinum Ba4 str. 657 CP001083.1:64078..65589
Clostridium botulinum CP000939.1:48537..50048 B1 str. Okra
Clostridium colicanis JPL 8 FJ957863.1: 1.. 1454
Clostridium colicanis JPL 9 FJ957864.1: 1.. 1456
Clostridium colicanis FJ957865.1: 1.. 1456 JPL 10
Firmicutes Clostridia Clostridiales Clostridiaceae1 Clostridium Clostridium colicanis Otu0277
Clostridium cellulovorans 22rA KF528156.1: 1.. 554
Clostridium sp. CL-2 LK021118.1: 1.. 1472
Firmicutes Clostridia Clostridiales Clostridiaceae Clostridium Clostridium cellulovorans Otu1274
Clostridium perfringens ATCC 13124 AB075767.1: 1.. 1460
Clostridium perfringens antibiotic (Nitrofurantoin)-resistant Y12669.1:1..1504
Clostridium perfringens CP000312.1:9992..11504 SM101
Firmicutes Clostridia Clostridiales Clostridiaceae1 Clostridium Clostridium perfringens* Otu0333
Clostridium sp. FM865923.1: 1.. 1424 H2-3
bacterium str. Rauti AY383733.1: 1.. 1483
Fastidiosipila sanguinis BRE20130822 KJ419955.1: 1.. 1362
Firmicutes Clostridia Clostridiales Ruminococcaceae Fastidiosipila Fastidiosipila sanguinis Otu0268
Clostridiales bacterium canine oral taxon 336 1G044 JN713505.1: 1.. 1488
Clostridiales bacterium canine oral taxon 082 OC038 JN713245.1: 1.. 1524
Firmicutes Clostridia Clostridiales Ruminococcaceae 1 Ruminococcaceae Ruminococcaceae 1 Otu0073
Firmicutes Clostridia Oscillospirales Hungateiclostridiaceae Mageeibacillus Mageeibacillus BVAB3* Otu1018
Firmicutes Clostridia Clostridiales Ruminococcaceae Mageeibacillus Mageeibacillus indolicus BVAB3* Otu0041
Mageeibacillus indolicus 0009-5 GQ900632.1: 1.. 1480
Mageeibacillus indolicus 0004-9 GQ900631.1: 1.. 1480
Mageeibacillusindolicus0009-5gi|1179766748|ref|NR 146698.1|
Firmicutes Clostridia Oscillospirales Hungateiclostridiaceae Fastidiosipila Fastidiosipila sp Otu0700
Clostridiales bacterium KA00274 KJ868804.1: 1.. 1408
Firmicutes Clostridia Clostridiales Ruminococcaceae Ruminococcaceae Ruminococcaceae 2(BVAB2*) Otu0748
BVAB2 rRNA115 AY958888.1: 1.. 1513
Firmicutes Clostridia Clostridiales Ruminococcaceae Ruminococcaceae Ruminococcaceae 2(BVAB2*) Otu0103
Firmicutes Clostridia Clostridiales Ruminococcaceae Ruminococcaceae Ruminococcaceae 2(BVAB2*) Otu0014
rumen bacterium NK4A65 GU324373.1: 1.. 1484
Acetivibrio cellulolyticus L35515.1:1..1464
Firmicutes Clostridia Monoglobales Monoglobaceae Monoglobus Monoglobus sp Otu1441
Clostridium sp. K13-19 DSM 2933 HE862234.1: 1.. 1506
Lactobacillales bacterium HY-36-1 AY581272.1: 1.. 1413
Firmicutes Clostridia Oscillospirales Oscillospiraceae Oscillibacter Oscillibacter valericigenes Otu2762
bacterium AN1045 DQ286651.1: 1.. 1422
Oscillospiraceae bacterium NML 61048 EU149939.1: 1.. 1450
Firmicutes Clostridia Oscillospirales Oscillospiraceae Oscillibacter Oscillibacter sp Otu1595
Clostridiales bacterium CIEAF 21 AB702937.1: 1.. 1496
Firmicutes Clostridia Clostridiales Ruminococcaceae 3 Ruminococcaceae Ruminococcaceae 3 Otu0610
Ruminococcaceae bacterium LM158 KJ875866.1: 1.. 1453
Ruminococcaceae bacterium LM158 KJ875867.1: 1.. 1453
bacterium P2C1 KM043765.1: 1.. 1467
Firmicutes Clostridia Oscillospirales Oscillospiraceae UCG-002 UCG-002 Otu0754
butyrate-producing bacterium A2-207 AJ270471.2:1..1484
Firmicutes Clostridia Eubacteriales Oscillospiraceae Agathobaculum Agathobaculum butyriciproducens Otu1120
Agathobaculum butyriciproducens (T) SR79 KP889099.1: 1.. 1328
Firmicutes Clostridia Eubacteriales Eubacteriales incertae sedis Intestinimonas Intestinimonas butyriciproducens Otu0752
Intestinimonas sp. GD4 LN870298.1: 1.. 1495
Intestinimonas butyriciproducens AP4 JX101685.1: 1.. 1488
Lachnospiraceae bacterium 19gly4 AF550610.1: 1.. 1416
Firmicutes Clostridia Oscillospirales Oscillospiraceae UCG-005 UCG-005 Otu0616
Eubacterium siraeum 70/3 EU266550.1: 1.. 1431
Firmicutes Clostridia Clostridiales Ruminococcaceae Eubacterium Eubacterium siraeum Otu0798
Eubacterium siraeum V10Sc8a FP929059.1:complement(2595678..2597174)
Firmicutes Clostridia Oscillospirales Ruminococcaceae Eubacterium Eubacterium siraeum Otu1896

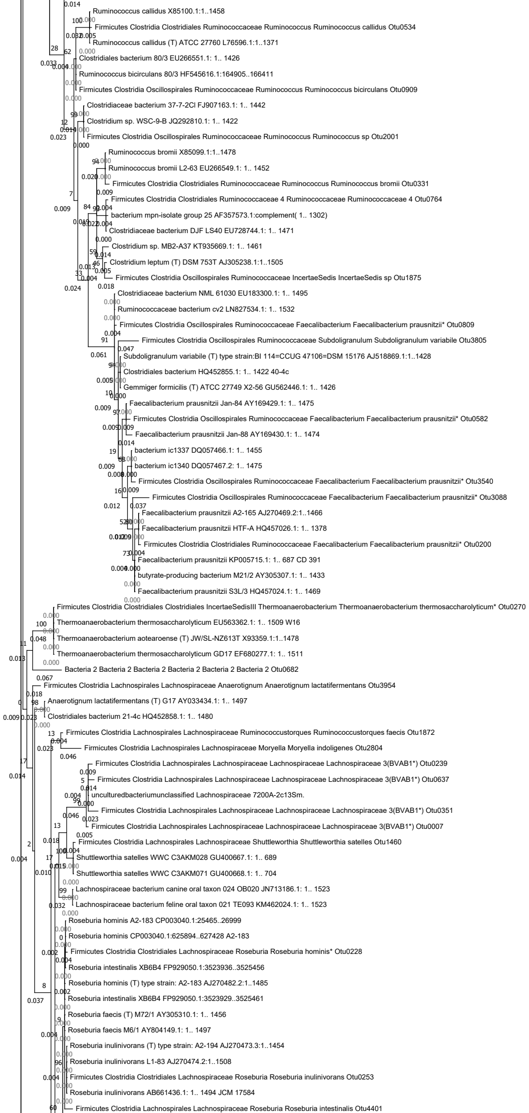

Ruminococcus callidus X85100.1:1..1458
Firmicutes Clostridia Clostridiales Ruminococcaceae Ruminococcus Ruminococcus callidus Otu0534
Ruminococcus callidus (T) ATCC 27760 L76596.1:1..1371
Clostridiales bacterium 80/3 EU266551.1: 1.. 1426
Ruminococcus bicirculans 80/3 HF545616.1:164905..166411
Firmicutes Clostridia Oscillospirales Ruminococcaceae Ruminococcus Ruminococcus bicirculans Otu0909
Clostridiaceae bacterium 37-7-2Cl FJ907163.1: 1.. 1442
Clostridium sp. WSC-9-B JQ292810.1: 1.. 1422
Firmicutes Clostridia Oscillospirales Ruminococcaceae Ruminococcus Ruminococcus sp Otu2001
Ruminococcus bromii X85099.1:1..1478
Ruminococcus bromii L2-63 EU266549.1: 1.. 1452
Firmicutes Clostridia Clostridiales Ruminococcaceae Ruminococcus Ruminococcus bromii Otu0331
Firmicutes Clostridia Clostridiales Ruminococcaceae 4 Ruminococcaceae Ruminococcaceae 4 Otu0764
bacterium mpn-isolate group 25 AF357573.1:complement(1.. 1302)
Clostridiaceae bacterium DJF LS40 EU728744.1: 1.. 1471
Clostridium sp. MB2-A37 KT935669.1: 1.. 1461
Clostridium leptum (T) DSM 753T AJ305238.1:1..1505
Firmicutes Clostridia Oscillospirales Ruminococcaceae IncertaeSedis IncertaeSedis sp Otu1875
Clostridiaceae bacterium NML 61030 EU183300.1: 1.. 1495
Ruminococcaceae bacterium cv2 LN827534.1: 1.. 1532
Firmicutes Clostridia Oscillospirales Ruminococcaceae Faecalibacterium Faecalibacterium prausnitzii* Otu0809
Firmicutes Clostridia Oscillospirales Ruminococcaceae Subdoligranulum Subdoligranulum variabile Otu3805
Subdoligranulum variabile (T) type strain:BI 114=CCUG 47106=DSM 15176 AJ518869.1:1..1428
Clostridiales bacterium HQ452855.1: 1.. 1422 40-4c
Gemmiger formicilis (T) ATCC 27749 X2-56 GU562446.1: 1.. 1426
Faecalibacterium prausnitzii Jan-84 AY169429.1: 1.. 1475
Firmicutes Clostridia Oscillospirales Ruminococcaceae Faecalibacterium Faecalibacterium prausnitzii* Otu0582
Faecalibacterium prausnitzii Jan-88 AY169430.1: 1.. 1474
bacterium ic1337 DQ057466.1: 1.. 1455
bacterium ic1340 DQ057467.2: 1.. 1475
Firmicutes Clostridia Oscillospirales Ruminococcaceae Faecalibacterium Faecalibacterium prausnitzii* Otu3540
Firmicutes Clostridia Oscillospirales Ruminococcaceae Faecalibacterium Faecalibacterium prausnitzii* Otu3088
Faecalibacterium prausnitzii A2-165 AJ270469.2:1..1466
Faecalibacterium prausnitzii HTF-A HQ457026.1: 1.. 1378
Firmicutes Clostridia Clostridiales Ruminococcaceae Faecalibacterium Faecalibacterium prausnitzii* Otu0200
Faecalibacterium prausnitzii KP005715.1: 1.. 687 CD 391
butyrate-producing bacterium M21/2 AY305307.1: 1.. 1433
Faecalibacterium prausnitzii S3L/3 HQ457024.1: 1.. 1469
Firmicutes Clostridia Clostridiales Clostridiales IncertaeSedisIII Thermoanaerobacterium Thermoanaerobacterium thermosaccharolyticum* Otu0270
Thermoanaerobacterium thermosaccharolyticum EU563362.1: 1.. 1509 W16
Thermoanaerobacterium aotearoense (T) JW/SL-NZ613T X93359.1:1..1478
Thermoanaerobacterium thermosaccharolyticum GD17 EF680277.1: 1.. 1511
Bacteria 2 Bacteria 2 Bacteria 2 Bacteria 2 Bacteria 2 Bacteria 2 Otu0682
Firmicutes Clostridia Lachnospirales Lachnospiraceae Anaerotignum Anaerotignum lactatifermentans Otu3954
Anaerotignum lactatifermentans (T) G17 AY033434.1: 1.. 1497
Clostridiales bacterium 21-4c HQ452858.1: 1.. 1480
Firmicutes Clostridia Lachnospirales Lachnospiraceae Ruminococcustorques Ruminococcustorques faecis Otu1872
Firmicutes Clostridia Lachnospirales Lachnospiraceae Moryella Moryella indoligenes Otu2804
Firmicutes Clostridia Lachnospirales Lachnospiraceae Lachnospiraceae Lachnospiraceae 3(BVAB1*) Otu0239
Firmicutes Clostridia Lachnospirales Lachnospiraceae Lachnospiraceae Lachnospiraceae 3(BVAB1*) Otu0637
unculturedbacteriumunclassified Lachnospiraceae 7200A-2c13Sm.
Firmicutes Clostridia Lachnospirales Lachnospiraceae Lachnospiraceae Lachnospiraceae 3(BVAB1*) Otu0351
Firmicutes Clostridia Lachnospirales Lachnospiraceae Lachnospiraceae Lachnospiraceae 3(BVAB1*) Otu0007
Firmicutes Clostridia Lachnospirales Lachnospiraceae Shuttleworthia Shuttleworthia satelles Otu1460
Shuttleworthia satelles WWC C3AKM028 GU400667.1: 1.. 689
Shuttleworthia satelles WWC C3AKM071 GU400668.1: 1.. 704
Lachnospiraceae bacterium canine oral taxon 024 OB020 JN713186.1: 1.. 1523
Lachnospiraceae bacterium feline oral taxon 021 TE093 KM462024.1: 1.. 1523
Roseburia hominis A2-183 CP003040.1:25465..26999
Roseburia hominis CP003040.1:625894..627428 A2-183
Firmicutes Clostridia Clostridiales Lachnospiraceae Roseburia Roseburia hominis* Otu0228
Roseburia intestinalis XB6B4 FP929050.1:3523936..3525456
Roseburia hominis (T) type strain: A2-183 AJ270482.2:1..1485
Roseburia intestinalis XB6B4 FP929050.1:3523929..3525461
Roseburia faecis (T) M72/1 AY305310.1: 1.. 1456
Roseburia faecis M6/1 AY804149.1: 1.. 1497
Roseburia inulinivorans (T) type strain: A2-194 AJ270473.3:1..1454
Roseburia inulinivorans L1-83 AJ270474.2:1..1508
Firmicutes Clostridia Clostridiales Lachnospiraceae Roseburia Roseburia inulinivorans Otu0253
Roseburia inulinivorans AB661436.1: 1.. 1494 JCM 17584
Firmicutes Clostridia Lachnospirales Lachnospiraceae Roseburia Roseburia intestinalis Otu4401

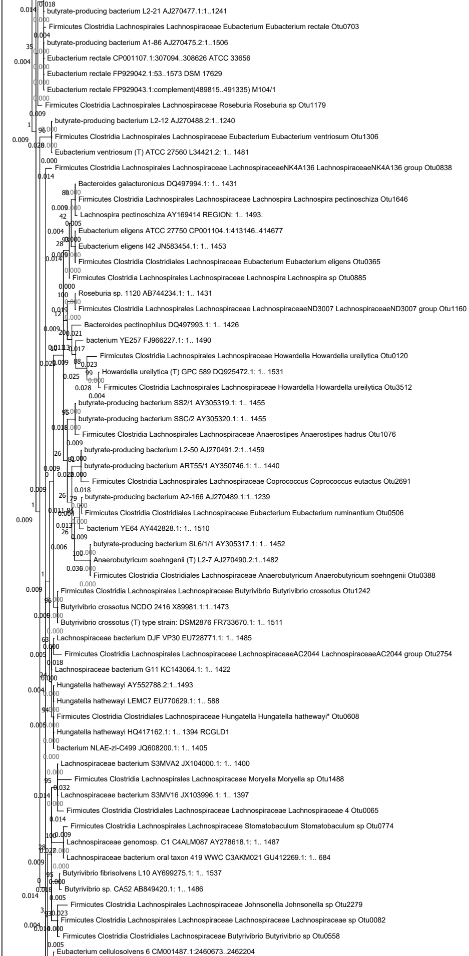
butyrate-producing bacterium L2-21 AJ270477.1:1..1241
Firmicutes Clostridia Lachnospirales Lachnospiraceae Eubacterium Eubacterium rectale Otu0703
butyrate-producing bacterium A1-86 AJ270475.2:1..1506
Eubacterium rectale CP001107.1:307094..308626 ATCC 33656
Eubacterium rectale FP929042.1:53..1573 DSM 17629
Eubacterium rectale FP929043.1:complement(489815..491335) M104/1
Firmicutes Clostridia Lachnospirales Lachnospiraceae Roseburia Roseburia sp Otu1179
butyrate-producing bacterium L2-12 AJ270488.2:1..1240
Firmicutes Clostridia Lachnospirales Lachnospiraceae Eubacterium Eubacterium ventriosum Otu1306
Eubacterium ventriosum (T) ATCC 27560 L34421.2: 1.. 1481
Firmicutes Clostridia Lachnospirales Lachnospiraceae LachnospiraceaeNK4A136 LachnospiraceaeNK4A136 group Otu0838
Bacteroides galacturonicus DQ497994.1: 1.. 1431
Firmicutes Clostridia Lachnospirales Lachnospiraceae Lachnospira Lachnospira pectinoschiza Otu1646
Lachnospira pectinoschiza AY169414 REGION: 1.. 1493.
Eubacterium eligens ATCC 27750 CP001104.1:413146..414677
Eubacterium eligens I42 JN583454.1: 1.. 1453
Firmicutes Clostridia Clostridiales Lachnospiraceae Eubacterium Eubacterium eligens Otu0365
Firmicutes Clostridia Lachnospirales Lachnospiraceae Lachnospira Lachnospira sp Otu0885
Roseburia sp. 1120 AB744234.1: 1.. 1431
Firmicutes Clostridia Lachnospirales Lachnospiraceae LachnospiraceaeND3007 LachnospiraceaeND3007 group Otu1160
Bacteroides pectinophilus DQ497993.1: 1.. 1426
bacterium YE257 FJ966227.1: 1.. 1490
Firmicutes Clostridia Lachnospirales Lachnospiraceae Howardella Howardella ureilytica Otu0120
Howardella ureilytica (T) GPC 589 DQ925472.1: 1.. 1531
Firmicutes Clostridia Lachnospirales Lachnospiraceae Howardella Howardella ureilytica Otu3512
butyrate-producing bacterium SS2/1 AY305319.1: 1.. 1455
butyrate-producing bacterium SSC/2 AY305320.1: 1.. 1455
Firmicutes Clostridia Lachnospirales Lachnospiraceae Anaerostipes Anaerostipes hadrus Otu1076
butyrate-producing bacterium L2-50 AJ270491.2:1..1459
butyrate-producing bacterium ART55/1 AY350746.1: 1.. 1440
Firmicutes Clostridia Lachnospirales Lachnospiraceae Coprococcus Coprococcus eutactus Otu2691
butyrate-producing bacterium A2-166 AJ270489.1:1..1239
Firmicutes Clostridia Clostridiales Lachnospiraceae Eubacterium Eubacterium ruminantium Otu0506
bacterium YE64 AY442828.1: 1.. 1510
butyrate-producing bacterium SL6/1/1 AY305317.1: 1.. 1452
Anaerobutyricum soehngenii (T) L2-7 AJ270490.2:1..1482
Firmicutes Clostridia Clostridiales Lachnospiraceae Anaerobutyricum Anaerobutyricum soehngenii Otu0388
Firmicutes Clostridia Lachnospirales Lachnospiraceae Butyrivibrio Butyrivibrio crossotus Otu1242
Butyrivibrio crossotus NCDO 2416 X89981.1:1..1473
Butyrivibrio crossotus (T) type strain: DSM2876 FR733670.1: 1.. 1511
Lachnospiraceae bacterium DJF VP30 EU728771.1: 1.. 1485
Firmicutes Clostridia Lachnospirales Lachnospiraceae LachnospiraceaeAC2044 LachnospiraceaeAC2044 group Otu2754
Lachnospiraceae bacterium G11 KC143064.1: 1.. 1422
Hungatella hathewayi AY552788.2:1..1493
Hungatella hathewayi LEMC7 EU770629.1: 1.. 588
Firmicutes Clostridia Clostridiales Lachnospiraceae Hungatella Hungatella hathewayi* Otu0608
Hungatella hathewayi HQ417162.1: 1.. 1394 RCGLD1
bacterium NLAE-zl-C499 JQ608200.1: 1.. 1405
Lachnospiraceae bacterium S3MVA2 JX104000.1: 1.. 1400
Firmicutes Clostridia Lachnospirales Lachnospiraceae Moryella Moryella sp Otu1488
Lachnospiraceae bacterium S3MV16 JX103996.1: 1.. 1397
Firmicutes Clostridia Clostridiales Lachnospiraceae Lachnospiraceae Lachnospiraceae 4 Otu0065
Firmicutes Clostridia Lachnospirales Lachnospiraceae Stomatobaculum Stomatobaculum sp Otu0774
Lachnospiraceae genomosp. C1 C4ALM087 AY278618.1: 1.. 1487
Lachnospiraceae bacterium oral taxon 419 WWC C3AKM021 GU412269.1: 1.. 684
Butyrivibrio fibrisolvens L10 AY699275.1: 1.. 1537
Butyrivibrio sp. CA52 AB849420.1: 1.. 1486
Firmicutes Clostridia Lachnospirales Lachnospiraceae Johnsonella Johnsonella sp Otu2279
Firmicutes Clostridia Lachnospirales Lachnospiraceae Lachnospiraceae Lachnospiraceae sp Otu0082
Firmicutes Clostridia Clostridiales Lachnospiraceae Butyrivibrio Butyrivibrio sp Otu0558
Eubacterium cellulosolvens 6 CM001487.1:2460673..2462204

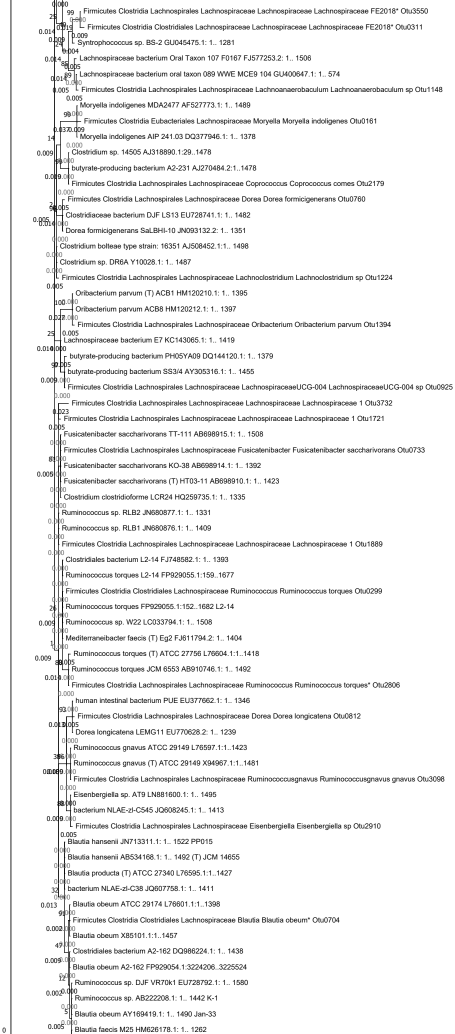
Firmicutes Clostridia Lachnospirales Lachnospiraceae Lachnospiraceae Lachnospiraceae FE2018* Otu3550
Firmicutes Clostridia Clostridiales Lachnospiraceae Lachnospiraceae Lachnospiraceae FE2018* Otu0311
Syntrophococcus sp. BS-2 GU045475.1: 1.. 1281
Lachnospiraceae bacterium Oral Taxon 107 F0167 FJ577253.2: 1.. 1506
Lachnospiraceae bacterium oral taxon 089 WWE MCE9 104 GU400647.1: 1.. 574
Firmicutes Clostridia Lachnospirales Lachnospiraceae Lachnoanaerobaculum Lachnoanaerobaculum sp Otu1148
Moryella indoligenes MDA2477 AF527773.1: 1.. 1489
Firmicutes Clostridia Eubacteriales Lachnospiraceae Moryella Moryella indoligenes Otu0161
Moryella indoligenes AIP 241.03 DQ377946.1: 1.. 1378
Clostridium sp. 14505 AJ318890.1:29..1478
butyrate-producing bacterium A2-231 AJ270484.2:1..1478
Firmicutes Clostridia Lachnospirales Lachnospiraceae Coprococcus Coprococcus comes Otu2179
Firmicutes Clostridia Lachnospirales Lachnospiraceae Dorea Dorea formicigenerans Otu0760
Clostridiaceae bacterium DJF LS13 EU728741.1: 1.. 1482
Dorea formicigenerans SaLBHI-10 JN093132.2: 1.. 1351
Clostridium bolteae type strain: 16351 AJ508452.1:1.. 1498
Clostridium sp. DR6A Y10028.1: 1.. 1487
Firmicutes Clostridia Lachnospirales Lachnospiraceae Lachnoclostridium Lachnoclostridium sp Otu1224
Oribacterium parvum (T) ACB1 HM120210.1: 1.. 1395
Oribacterium parvum ACB8 HM120212.1: 1.. 1397
Firmicutes Clostridia Lachnospirales Lachnospiraceae Oribacterium Oribacterium parvum Otu1394
Lachnospiraceae bacterium E7 KC143065.1: 1.. 1419
butyrate-producing bacterium PH05YA09 DQ144120.1: 1.. 1379
butyrate-producing bacterium SS3/4 AY305316.1: 1.. 1455
Firmicutes Clostridia Lachnospirales Lachnospiraceae LachnospiraceaeUCG-004 LachnospiraceaeUCG-004 sp Otu0925
Firmicutes Clostridia Lachnospirales Lachnospiraceae Lachnospiraceae Lachnospiraceae 1 Otu3732
Firmicutes Clostridia Lachnospirales Lachnospiraceae Lachnospiraceae Lachnospiraceae 1 Otu1721
Fusicatenibacter saccharivorans TT-111 AB698915.1: 1.. 1508
Firmicutes Clostridia Lachnospirales Lachnospiraceae Fusicatenibacter Fusicatenibacter saccharivorans Otu0733
Fusicatenibacter saccharivorans KO-38 AB698914.1: 1.. 1392
Fusicatenibacter saccharivorans (T) HT03-11 AB698910.1: 1.. 1423
Clostridium clostridioforme LCR24 HQ259735.1: 1.. 1335
Ruminococcus sp. RLB2 JN680877.1: 1.. 1331
Ruminococcus sp. RLB1 JN680876.1: 1.. 1409
Firmicutes Clostridia Lachnospirales Lachnospiraceae Lachnospiraceae Lachnospiraceae 1 Otu1889
Clostridiales bacterium L2-14 FJ748582.1: 1.. 1393
Ruminococcus torques L2-14 FP929055.1:159..1677
Firmicutes Clostridia Clostridiales Lachnospiraceae Ruminococcus Ruminococcus torques Otu0299
Ruminococcus torques FP929055.1:152..1682 L2-14
Ruminococcus sp. W22 LC033794.1: 1.. 1508
Mediterraneibacter faecis (T) Eg2 FJ611794.2: 1.. 1404
Ruminococcus torques (T) ATCC 27756 L76604.1:1..1418
Ruminococcus torques JCM 6553 AB910746.1: 1.. 1492
Firmicutes Clostridia Lachnospirales Lachnospiraceae Ruminococcus Ruminococcus torques* Otu2806
human intestinal bacterium PUE EU377662.1: 1.. 1346
Firmicutes Clostridia Lachnospirales Lachnospiraceae Dorea Dorea longicatena Otu0812
Dorea longicatena LEMG11 EU770628.2: 1.. 1239
Ruminococcus gnavus ATCC 29149 L76597.1:1..1423
Ruminococcus gnavus (T) ATCC 29149 X94967.1:1..1481
Firmicutes Clostridia Lachnospirales Lachnospiraceae Ruminococcusgnavus Ruminococcusgnavus gnavus Otu3098
Eisenbergiella sp. AT9 LN881600.1: 1.. 1495
bacterium NLAE-zl-C545 JQ608245.1: 1.. 1413
Firmicutes Clostridia Lachnospirales Lachnospiraceae Eisenbergiella Eisenbergiella sp Otu2910
Blautia hansenii JN713311.1: 1.. 1522 PP015
Blautia hansenii AB534168.1: 1.. 1492 (T) JCM 14655
Blautia producta (T) ATCC 27340 L76595.1:1..1427
bacterium NLAE-zl-C38 JQ607758.1: 1.. 1411
Blautia obeum ATCC 29174 L76601.1:1..1398
Firmicutes Clostridia Clostridiales Lachnospiraceae Blautia Blautia obeum* Otu0704
Blautia obeum X85101.1:1..1457
Clostridiales bacterium A2-162 DQ986224.1: 1.. 1438
Blautia obeum A2-162 FP929054.1:3224206..3225524
Ruminococcus sp. DJF VR70k1 EU728792.1: 1.. 1580
Ruminococcus sp. AB222208.1: 1.. 1442 K-1
Blautia obeum AY169419.1: 1.. 1490 Jan-33
Blautia faecis M25 HM626178.1: 1.. 1262
0

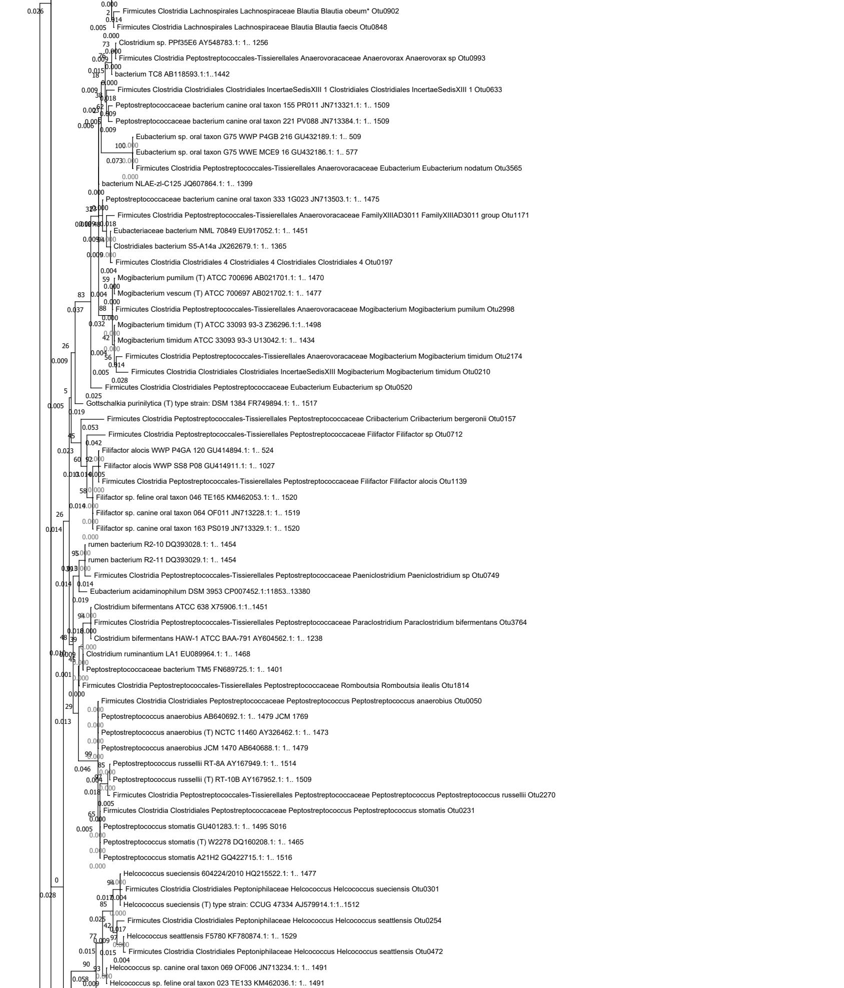
Firmicutes Clostridia Lachnospirales Lachnospiraceae Blautia Blautia obeum* Otu0902
Firmicutes Clostridia Lachnospirales Lachnospiraceae Blautia Blautia faecis Otu0848
Clostridium sp. PPf35E6 AY548783.1: 1.. 1256
Firmicutes Clostridia Peptostreptococcales-Tissierellales Anaerovoracaceae Anaerovorax Anaerovorax sp Otu0993
bacterium TC8 AB118593.1:1..1442
Firmicutes Clostridia Clostridiales Clostridiales IncertaeSedisXIII 1 Clostridiales Clostridiales IncertaeSedisXIII 1 Otu0633
Peptostreptococcaceae bacterium canine oral taxon 155 PR011 JN713321.1: 1.. 1509
Peptostreptococcaceae bacterium canine oral taxon 221 PV088 JN713384.1: 1.. 1509
Eubacterium sp. oral taxon G75 WWP P4GB 216 GU432189.1: 1.. 509
Eubacterium sp. oral taxon G75 WWE MCE9 16 GU432186.1: 1.. 577
Firmicutes Clostridia Peptostreptococcales-Tissierellales Anaerovoracaceae Eubacterium Eubacterium nodatum Otu3565
bacterium NLAE-zl-C125 JQ607864.1: 1.. 1399
Peptostreptococcaceae bacterium canine oral taxon 333 1G023 JN713503.1: 1.. 1475
Firmicutes Clostridia Peptostreptococcales-Tissierellales Anaerovoracaceae FamilyXIIIAD3011 FamilyXIIIAD3011 group Otu1171
Eubacteriaceae bacterium NML 70849 EU917052.1: 1.. 1451
Clostridiales bacterium S5-A14a JX262679.1: 1.. 1365
Firmicutes Clostridia Clostridiales 4 Clostridiales 4 Clostridiales Clostridiales 4 Otu0197
Mogibacterium pumilum (T) ATCC 700696 AB021701.1: 1.. 1470
Mogibacterium vescum (T) ATCC 700697 AB021702.1: 1.. 1477
Firmicutes Clostridia Peptostreptococcales-Tissierellales Anaerovoracaceae Mogibacterium Mogibacterium pumilum Otu2998
Mogibacterium timidum (T) ATCC 33093 93-3 Z36296.1:1..1498
Mogibacterium timidum ATCC 33093 93-3 U13042.1: 1.. 1434
Firmicutes Clostridia Peptostreptococcales-Tissierellales Anaerovoracaceae Mogibacterium Mogibacterium timidum Otu2174
Firmicutes Clostridia Clostridiales Clostridiales IncertaeSedisXIII Mogibacterium Mogibacterium timidum Otu0210
Firmicutes Clostridia Clostridiales Peptostreptococcaceae Eubacterium Eubacterium sp Otu0520
Gottschalkia purinilytica (T) type strain: DSM 1384 FR749894.1: 1.. 1517
Firmicutes Clostridia Peptostreptococcales-Tissierellales Peptostreptococcaceae Criibacterium Criibacterium bergeronii Otu0157
Firmicutes Clostridia Peptostreptococcales-Tissierellales Peptostreptococcaceae Filifactor Filifactor sp Otu0712
Filifactor alocis WWP P4GA 120 GU414894.1: 1.. 524
Filifactor alocis WWP SS8 P08 GU414911.1: 1.. 1027
Firmicutes Clostridia Peptostreptococcales-Tissierellales Peptostreptococcaceae Filifactor Filifactor alocis Otu1139
Filifactor sp. feline oral taxon 046 TE165 KM462053.1: 1.. 1520
Filifactor sp. canine oral taxon 064 OF011 JN713228.1: 1.. 1519
Filifactor sp. canine oral taxon 163 PS019 JN713329.1: 1.. 1520
rumen bacterium R2-10 DQ393028.1: 1.. 1454
rumen bacterium R2-11 DQ393029.1: 1.. 1454
Firmicutes Clostridia Peptostreptococcales-Tissierellales Peptostreptococcaceae Paeniclostridium Paeniclostridium sp Otu0749
Eubacterium acidaminophilum DSM 3953 CP007452.1:11853..13380
Clostridium bifermentans ATCC 638 X75906.1:1..1451
Firmicutes Clostridia Peptostreptococcales-Tissierellales Peptostreptococcaceae Paraclostridium Paraclostridium bifermentans Otu3764
Clostridium bifermentans HAW-1 ATCC BAA-791 AY604562.1: 1.. 1238
Clostridium ruminantium LA1 EU089964.1: 1.. 1468
Peptostreptococcaceae bacterium TM5 FN689725.1: 1.. 1401
Firmicutes Clostridia Peptostreptococcales-Tissierellales Peptostreptococcaceae Romboutsia Romboutsia ilealis Otu1814
Firmicutes Clostridia Clostridiales Peptostreptococcaceae Peptostreptococcus Peptostreptococcus anaerobius Otu0050
Peptostreptococcus anaerobius AB640692.1: 1.. 1479 JCM 1769
Peptostreptococcus anaerobius (T) NCTC 11460 AY326462.1: 1.. 1473
Peptostreptococcus anaerobius JCM 1470 AB640688.1: 1.. 1479
Peptostreptococcus russellii RT-8A AY167949.1: 1.. 1514
Peptostreptococcus russellii (T) RT-10B AY167952.1: 1.. 1509
Firmicutes Clostridia Peptostreptococcales-Tissierellales Peptostreptococcaceae Peptostreptococcus Peptostreptococcus russellii Otu2270
Firmicutes Clostridia Clostridiales Peptostreptococcaceae Peptostreptococcus Peptostreptococcus stomatis Otu0231
Peptostreptococcus stomatis GU401283.1: 1.. 1495 S016
Peptostreptococcus stomatis (T) W2278 DQ160208.1: 1.. 1465
Peptostreptococcus stomatis A21H2 GQ422715.1: 1.. 1516
Helcococcus sueciensis 604224/2010 HQ215522.1: 1.. 1477
Firmicutes Clostridia Clostridiales Peptoniphilaceae Helcococcus Helcococcus sueciensis Otu0301
Helcococcus sueciensis (T) type strain: CCUG 47334 AJ579914.1:1..1512
Firmicutes Clostridia Clostridiales Peptoniphilaceae Helcococcus Helcococcus seattlensis Otu0254
Helcococcus seattlensis F5780 KF780874.1: 1.. 1529
Firmicutes Clostridia Clostridiales Peptoniphilaceae Helcococcus Helcococcus seattlensis Otu0472
Helcococcus sp. canine oral taxon 069 OF006 JN713234.1: 1.. 1491
Helcococcus sp. feline oral taxon 023 TE133 KM462036.1: 1.. 1491

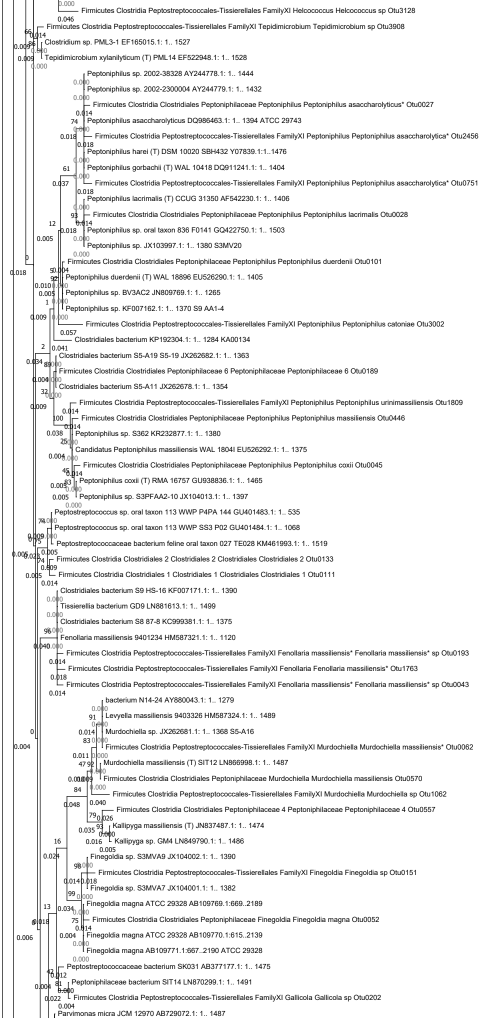
Firmicutes Clostridia Peptostreptococcales-Tissierellales FamilyXI Helcococcus Helcococcus sp Otu3128
Firmicutes Clostridia Peptostreptococcales-Tissierellales FamilyXI Tepidimicrobium Tepidimicrobium sp Otu3908
Clostridium sp. PML3-1 EF165015.1: 1.. 1527
Tepidimicrobium xylanilyticum (T) PML14 EF522948.1: 1.. 1528
Peptoniphilus sp. 2002-38328 AY244778.1: 1.. 1444
Peptoniphilus sp. 2002-2300004 AY244779.1: 1.. 1432
Firmicutes Clostridia Clostridiales Peptoniphilaceae Peptoniphilus Peptoniphilus asaccharolyticus* Otu0027
Peptoniphilus asaccharolyticus DQ986463.1: 1.. 1394 ATCC 29743
Firmicutes Clostridia Peptostreptococcales-Tissierellales FamilyXI Peptoniphilus Peptoniphilus asaccharolytica* Otu2456
Peptoniphilus harei (T) DSM 10020 SBH432 Y07839.1:1..1476
Peptoniphilus gorbachii (T) WAL 10418 DQ911241.1: 1.. 1404
Firmicutes Clostridia Peptostreptococcales-Tissierellales FamilyXI Peptoniphilus Peptoniphilus asaccharolytica* Otu0751
Peptoniphilus lacrimalis (T) CCUG 31350 AF542230.1: 1.. 1406
Firmicutes Clostridia Clostridiales Peptoniphilaceae Peptoniphilus Peptoniphilus lacrimalis Otu0028
Peptoniphilus sp. oral taxon 836 F0141 GQ422750.1: 1.. 1503
Peptoniphilus sp. JX103997.1: 1.. 1380 S3MV20
Firmicutes Clostridia Clostridiales Peptoniphilaceae Peptoniphilus Peptoniphilus duerdenii Otu0101
Peptoniphilus duerdenii (T) WAL 18896 EU526290.1: 1.. 1405
Peptoniphilus sp. BV3AC2 JN809769.1: 1.. 1265
Peptoniphilus sp. KF007162.1: 1.. 1370 S9 AA1-4
Firmicutes Clostridia Peptostreptococcales-Tissierellales FamilyXI Peptoniphilus Peptoniphilus catoniae Otu3002
Clostridiales bacterium KP192304.1: 1.. 1284 KA00134
Clostridiales bacterium S5-A19 S5-19 JX262682.1: 1.. 1363
Firmicutes Clostridia Clostridiales Peptoniphilaceae 6 Peptoniphilaceae Peptoniphilaceae 6 Otu0189
Clostridiales bacterium S5-A11 JX262678.1: 1.. 1354
Firmicutes Clostridia Peptostreptococcales-Tissierellales FamilyXI Peptoniphilus Peptoniphilus urinimassiliensis Otu1809
Firmicutes Clostridia Clostridiales Peptoniphilaceae Peptoniphilus Peptoniphilus massiliensis Otu0446
Peptoniphilus sp. S362 KR232877.1: 1.. 1380
Candidatus Peptoniphilus massiliensis WAL 1804I EU526292.1: 1.. 1375
Firmicutes Clostridia Clostridiales Peptoniphilaceae Peptoniphilus Peptoniphilus coxii Otu0045
Peptoniphilus coxii (T) RMA 16757 GU938836.1: 1.. 1465
Peptoniphilus sp. S3PFAA2-10 JX104013.1: 1.. 1397
Peptostreptococcus sp. oral taxon 113 WWP P4PA 144 GU401483.1: 1.. 535
Peptostreptococcus sp. oral taxon 113 WWP SS3 P02 GU401484.1: 1.. 1068
Peptostreptococcaceae bacterium feline oral taxon 027 TE028 KM461993.1: 1.. 1519
Firmicutes Clostridia Clostridiales 2 Clostridiales 2 Clostridiales Clostridiales 2 Otu0133
Firmicutes Clostridia Clostridiales 1 Clostridiales 1 Clostridiales Clostridiales 1 Otu0111
Clostridiales bacterium S9 HS-16 KF007171.1: 1.. 1390
Tissierellia bacterium GD9 LN881613.1: 1.. 1499
Clostridiales bacterium S8 87-8 KC999381.1: 1.. 1375
Fenollaria massiliensis 9401234 HM587321.1: 1.. 1120
Firmicutes Clostridia Peptostreptococcales-Tissierellales FamilyXI Fenollaria massiliensis* Fenollaria massiliensis* sp Otu0193
Firmicutes Clostridia Peptostreptococcales-Tissierellales FamilyXI Fenollaria Fenollaria massiliensis* Otu1763
Firmicutes Clostridia Peptostreptococcales-Tissierellales FamilyXI Fenollaria massiliensis* Fenollaria massiliensis* sp Otu0043
bacterium N14-24 AY880043.1: 1.. 1279
Levyella massiliensis 9403326 HM587324.1: 1.. 1489
Murdochiella sp. JX262681.1: 1.. 1368 S5-A16
Firmicutes Clostridia Peptostreptococcales-Tissierellales FamilyXI Murdochiella Murdochiella massiliensis* Otu0062
Murdochiella massiliensis (T) SIT12 LN866998.1: 1.. 1487
Firmicutes Clostridia Clostridiales Peptoniphilaceae Murdochiella Murdochiella massiliensis Otu0570
Firmicutes Clostridia Peptostreptococcales-Tissierellales FamilyXI Murdochiella Murdochiella sp Otu1062
Firmicutes Clostridia Clostridiales Peptoniphilaceae 4 Peptoniphilaceae Peptoniphilaceae 4 Otu0557
Kallipyga massiliensis (T) JN837487.1: 1.. 1474
Kallipyga sp. GM4 LN849790.1: 1.. 1486
Finegoldia sp. S3MVA9 JX104002.1: 1.. 1390
Firmicutes Clostridia Peptostreptococcales-Tissierellales FamilyXI Finegoldia Finegoldia sp Otu0151
Finegoldia sp. S3MVA7 JX104001.1: 1.. 1382
Finegoldia magna ATCC 29328 AB109769.1:669..2189
Firmicutes Clostridia Clostridiales Peptoniphilaceae Finegoldia Finegoldia magna Otu0052
Finegoldia magna ATCC 29328 AB109770.1:615..2139
Finegoldia magna AB109771.1:667..2190 ATCC 29328
Peptostreptococcaceae bacterium SK031 AB377177.1: 1.. 1475
Peptoniphilaceae bacterium SIT14 LN870299.1: 1.. 1491
Firmicutes Clostridia Peptostreptococcales-Tissierellales FamilyXI Gallicola Gallicola sp Otu0202
Parvimonas micra JCM 12970 AB729072.1: 1.. 1487

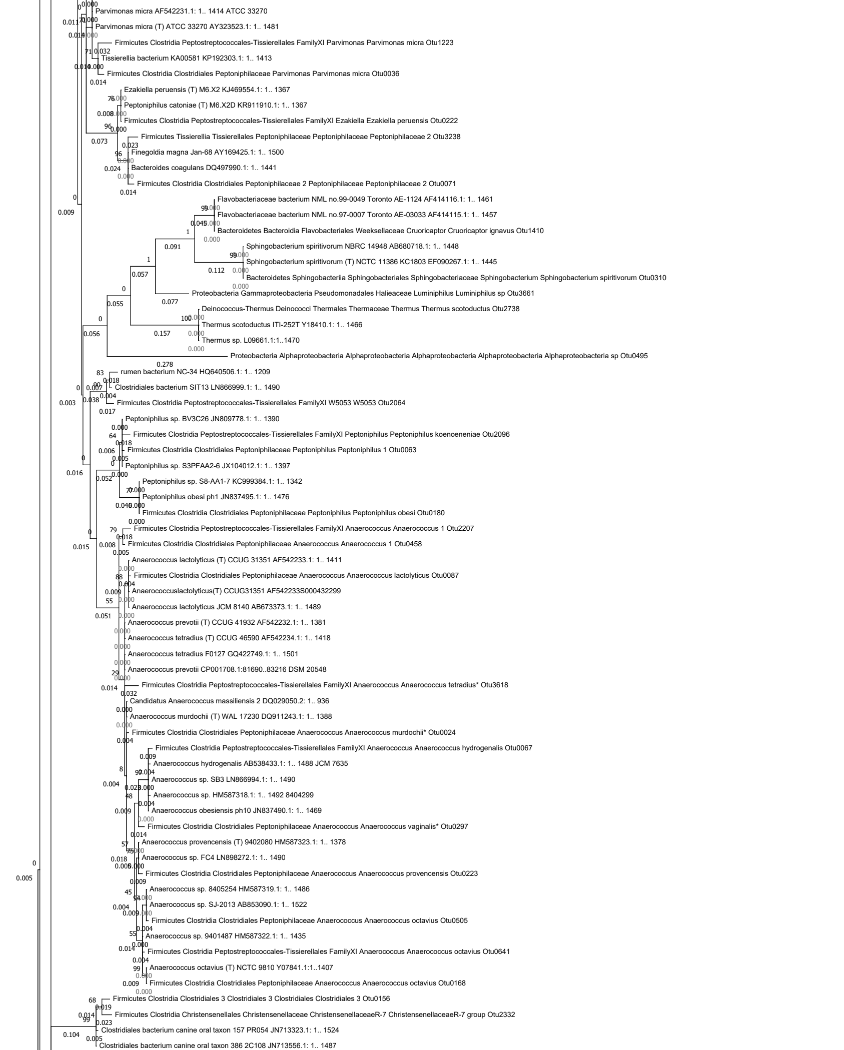
Parvimonas micra AF542231.1: 1.. 1414 ATCC 33270
Parvimonas micra (T) ATCC 33270 AY323523.1: 1.. 1481
Firmicutes Clostridia Peptostreptococcales-Tissierellales FamilyXI Parvimonas Parvimonas micra Otu1223
Tissierellia bacterium KA00581 KP192303.1: 1.. 1413
Firmicutes Clostridia Clostridiales Peptoniphilaceae Parvimonas Parvimonas micra Otu0036
Ezakiella peruensis (T) M6.X2 KJ469554.1: 1.. 1367
Peptoniphilus catoniae (T) M6.X2D KR911910.1: 1.. 1367
Firmicutes Clostridia Peptostreptococcales-Tissierellales FamilyXI Ezakiella Ezakiella peruensis Otu0222
Firmicutes Tissierellia Tissierellales Peptoniphilaceae Peptoniphilaceae Peptoniphilaceae 2 Otu3238
Finegoldia magna Jan-68 AY169425.1: 1.. 1500
Bacteroides coagulans DQ497990.1: 1.. 1441
Firmicutes Clostridia Clostridiales Peptoniphilaceae 2 Peptoniphilaceae Peptoniphilaceae 2 Otu0071
Flavobacteriaceae bacterium NML no.99-0049 Toronto AE-1124 AF414116.1: 1.. 1461
Flavobacteriaceae bacterium NML no.97-0007 Toronto AE-03033 AF414115.1: 1.. 1457
Bacteroidetes Bacteroidia Flavobacteriales Weeksellaceae Cruoricaptor Cruoricaptor ignavus Otu1410
Sphingobacterium spiritivorum NBRC 14948 AB680718.1: 1.. 1448
Sphingobacterium spiritivorum (T) NCTC 11386 KC1803 EF090267.1: 1.. 1445
Bacteroidetes Sphingobacteriia Sphingobacteriales Sphingobacteriaceae Sphingobacterium Sphingobacterium spiritivorum Otu0310
Proteobacteria Gammaproteobacteria Pseudomonadales Halieaceae Luminiphilus Luminiphilus sp Otu3661
Deinococcus-Thermus Deinococci Thermales Thermaceae Thermus Thermus scotoductus Otu2738
Thermus scotoductus ITI-252T Y18410.1: 1.. 1466
Thermus sp. L09661.1:1..1470
Proteobacteria Alphaproteobacteria Alphaproteobacteria Alphaproteobacteria Alphaproteobacteria Alphaproteobacteria sp Otu0495
rumen bacterium NC-34 HQ640506.1: 1.. 1209
Clostridiales bacterium SIT13 LN866999.1: 1.. 1490
Firmicutes Clostridia Peptostreptococcales-Tissierellales FamilyXI W5053 W5053 Otu2064
Peptoniphilus sp. BV3C26 JN809778.1: 1.. 1390
Firmicutes Clostridia Peptostreptococcales-Tissierellales FamilyXI Peptoniphilus Peptoniphilus koenoeneniae Otu2096
Firmicutes Clostridia Clostridiales Peptoniphilaceae Peptoniphilus Peptoniphilus 1 Otu0063
Peptoniphilus sp. S3PFAA2-6 JX104012.1: 1.. 1397
Peptoniphilus sp. S8-AA1-7 KC999384.1: 1.. 1342
Peptoniphilus obesi ph1 JN837495.1: 1.. 1476
Firmicutes Clostridia Clostridiales Peptoniphilaceae Peptoniphilus Peptoniphilus obesi Otu0180
Firmicutes Clostridia Peptostreptococcales-Tissierellales FamilyXI Anaerococcus Anaerococcus 1 Otu2207
Firmicutes Clostridia Clostridiales Peptoniphilaceae Anaerococcus Anaerococcus 1 Otu0458
Anaerococcus lactolyticus (T) CCUG 31351 AF542233.1: 1.. 1411
Firmicutes Clostridia Clostridiales Peptoniphilaceae Anaerococcus Anaerococcus lactolyticus Otu0087
Anaerococcuslactolyticus(T) CCUG31351 AF542233S000432299
Anaerococcus lactolyticus JCM 8140 AB673373.1: 1.. 1489
Anaerococcus prevotii (T) CCUG 41932 AF542232.1: 1.. 1381
Anaerococcus tetradius (T) CCUG 46590 AF542234.1: 1.. 1418
Anaerococcus tetradius F0127 GQ422749.1: 1.. 1501
Anaerococcus prevotii CP001708.1:81690..83216 DSM 20548
Firmicutes Clostridia Peptostreptococcales-Tissierellales FamilyXI Anaerococcus Anaerococcus tetradius* Otu3618
Candidatus Anaerococcus massiliensis 2 DQ029050.2: 1.. 936
Anaerococcus murdochii (T) WAL 17230 DQ911243.1: 1.. 1388
Firmicutes Clostridia Clostridiales Peptoniphilaceae Anaerococcus Anaerococcus murdochii* Otu0024
Firmicutes Clostridia Peptostreptococcales-Tissierellales FamilyXI Anaerococcus Anaerococcus hydrogenalis Otu0067
Anaerococcus hydrogenalis AB538433.1: 1.. 1488 JCM 7635
Anaerococcus sp. SB3 LN866994.1: 1.. 1490
Anaerococcus sp. HM587318.1: 1.. 1492 8404299
Anaerococcus obesiensis ph10 JN837490.1: 1.. 1469
Firmicutes Clostridia Clostridiales Peptoniphilaceae Anaerococcus Anaerococcus vaginalis* Otu0297
Anaerococcus provencensis (T) 9402080 HM587323.1: 1.. 1378
Anaerococcus sp. FC4 LN898272.1: 1.. 1490
Firmicutes Clostridia Clostridiales Peptoniphilaceae Anaerococcus Anaerococcus provencensis Otu0223
Anaerococcus sp. 8405254 HM587319.1: 1.. 1486
Anaerococcus sp. SJ-2013 AB853090.1: 1.. 1522
Firmicutes Clostridia Clostridiales Peptoniphilaceae Anaerococcus Anaerococcus octavius Otu0505
Anaerococcus sp. 9401487 HM587322.1: 1.. 1435
Firmicutes Clostridia Peptostreptococcales-Tissierellales FamilyXI Anaerococcus Anaerococcus octavius Otu0641
Anaerococcus octavius (T) NCTC 9810 Y07841.1:1..1407
Firmicutes Clostridia Clostridiales Peptoniphilaceae Anaerococcus Anaerococcus octavius Otu0168
Firmicutes Clostridia Clostridiales 3 Clostridiales 3 Clostridiales Clostridiales 3 Otu0156
Firmicutes Clostridia Christensenellales Christensenellaceae ChristensenellaceaeR-7 ChristensenellaceaeR-7 group Otu2332
Clostridiales bacterium canine oral taxon 157 PR054 JN713323.1: 1.. 1524
Clostridiales bacterium canine oral taxon 386 2C108 JN713556.1: 1.. 1487

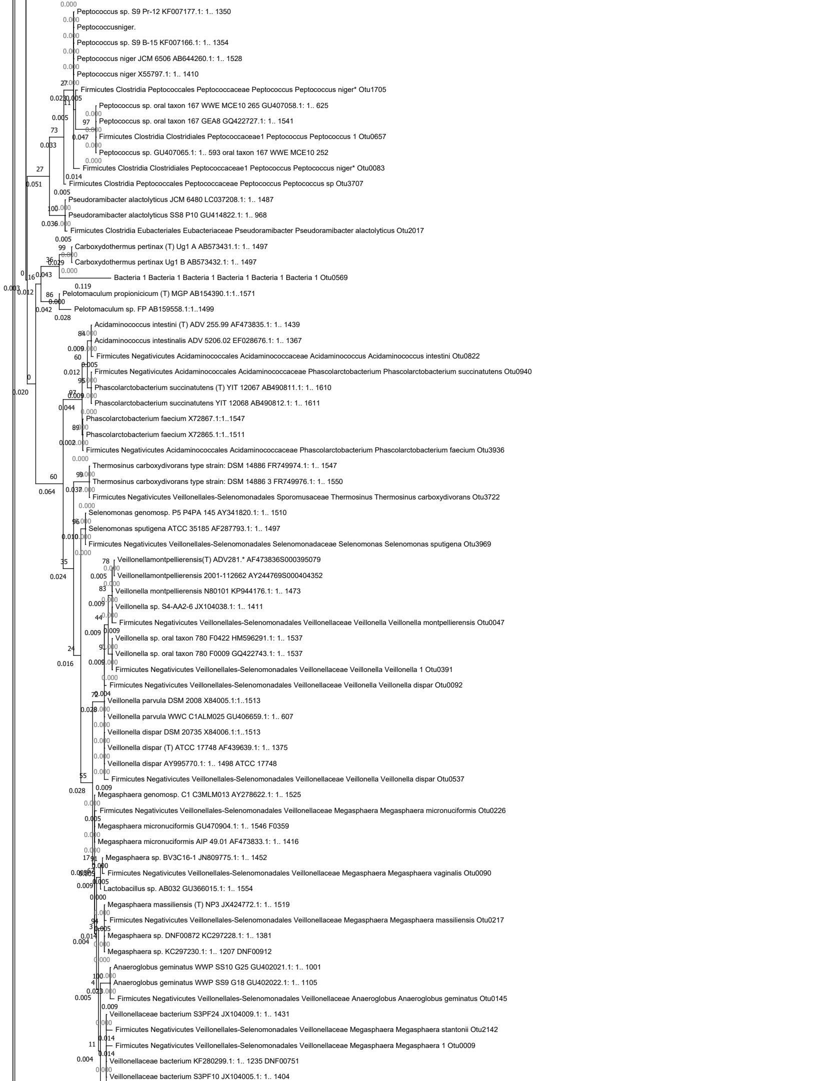
Peptococcus sp. S9 Pr-12 KF007177.1: 1.. 1350
Peptococcusniger.
Peptococcus sp. S9 B-15 KF007166.1: 1.. 1354
Peptococcus niger JCM 6506 AB644260.1: 1.. 1528
Peptococcus niger X55797.1: 1.. 1410
Firmicutes Clostridia Peptococcales Peptococcaceae Peptococcus Peptococcus niger* Otu1705
Peptococcus sp. oral taxon 167 WWE MCE10 265 GU407058.1: 1.. 625
Peptococcus sp. oral taxon 167 GEA8 GQ422727.1: 1.. 1541
Firmicutes Clostridia Clostridiales Peptococcaceae1 Peptococcus Peptococcus 1 Otu0657
Peptococcus sp. GU407065.1: 1.. 593 oral taxon 167 WWE MCE10 252
Firmicutes Clostridia Clostridiales Peptococcaceae1 Peptococcus Peptococcus niger* Otu0083
Firmicutes Clostridia Peptococcales Peptococcaceae Peptococcus Peptococcus sp Otu3707
Pseudoramibacter alactolyticus JCM 6480 LC037208.1: 1.. 1487
Pseudoramibacter alactolyticus SS8 P10 GU414822.1: 1.. 968
Firmicutes Clostridia Eubacteriales Eubacteriaceae Pseudoramibacter Pseudoramibacter alactolyticus Otu2017
Carboxydothermus pertinax (T) Ug1 A AB573431.1: 1.. 1497
Carboxydothermus pertinax Ug1 B AB573432.1: 1.. 1497
Bacteria 1 Bacteria 1 Bacteria 1 Bacteria 1 Bacteria 1 Bacteria 1 Otu0569
Pelotomaculum propionicicum (T) MGP AB154390.1:1..1571
Pelotomaculum sp. FP AB159558.1:1..1499
Acidaminococcus intestini (T) ADV 255.99 AF473835.1: 1.. 1439
Acidaminococcus intestinalis ADV 5206.02 EF028676.1: 1.. 1367
Firmicutes Negativicutes Acidaminococcales Acidaminococcaceae Acidaminococcus Acidaminococcus intestini Otu0822
Firmicutes Negativicutes Acidaminococcales Acidaminococcaceae Phascolarctobacterium Phascolarctobacterium succinatutens Otu0940
Phascolarctobacterium succinatutens (T) YIT 12067 AB490811.1: 1.. 1610
Phascolarctobacterium succinatutens YIT 12068 AB490812.1: 1.. 1611
Phascolarctobacterium faecium X72867.1:1..1547
Phascolarctobacterium faecium X72865.1:1..1511
Firmicutes Negativicutes Acidaminococcales Acidaminococcaceae Phascolarctobacterium Phascolarctobacterium faecium Otu3936
Thermosinus carboxydivorans type strain: DSM 14886 FR749974.1: 1.. 1547
Thermosinus carboxydivorans type strain: DSM 14886 3 FR749976.1: 1.. 1550
Firmicutes Negativicutes Veillonellales-Selenomonadales Sporomusaceae Thermosinus Thermosinus carboxydivorans Otu3722
Selenomonas genomosp. P5 P4PA 145 AY341820.1: 1.. 1510
Selenomonas sputigena ATCC 35185 AF287793.1: 1.. 1497
Firmicutes Negativicutes Veillonellales-Selenomonadales Selenomonadaceae Selenomonas Selenomonas sputigena Otu3969
Veillonellamontpellierensis(T) ADV281.* AF473836S000395079
Veillonellamontpellierensis 2001-112662 AY244769S000404352
Veillonella montpellierensis N80101 KP944176.1: 1.. 1473
Veillonella sp. S4-AA2-6 JX104038.1: 1.. 1411
Firmicutes Negativicutes Veillonellales-Selenomonadales Veillonellaceae Veillonella Veillonella montpellierensis Otu0047
Veillonella sp. oral taxon 780 F0422 HM596291.1: 1.. 1537
Veillonella sp. oral taxon 780 F0009 GQ422743.1: 1.. 1537
Firmicutes Negativicutes Veillonellales-Selenomonadales Veillonellaceae Veillonella Veillonella 1 Otu0391
Firmicutes Negativicutes Veillonellales-Selenomonadales Veillonellaceae Veillonella Veillonella dispar Otu0092
Veillonella parvula DSM 2008 X84005.1:1..1513
Veillonella parvula WWC C1ALM025 GU406659.1: 1.. 607
Veillonella dispar DSM 20735 X84006.1:1..1513
Veillonella dispar (T) ATCC 17748 AF439639.1: 1.. 1375
Veillonella dispar AY995770.1: 1.. 1498 ATCC 17748
Firmicutes Negativicutes Veillonellales-Selenomonadales Veillonellaceae Veillonella Veillonella dispar Otu0537
Megasphaera genomosp. C1 C3MLM013 AY278622.1: 1.. 1525
Firmicutes Negativicutes Veillonellales-Selenomonadales Veillonellaceae Megasphaera Megasphaera micronuciformis Otu0226
Megasphaera micronuciformis GU470904.1: 1.. 1546 F0359
Megasphaera micronuciformis AIP 49.01 AF473833.1: 1.. 1416
Megasphaera sp. BV3C16-1 JN809775.1: 1.. 1452
Firmicutes Negativicutes Veillonellales-Selenomonadales Veillonellaceae Megasphaera Megasphaera vaginalis Otu0090
Lactobacillus sp. AB032 GU366015.1: 1.. 1554
Megasphaera massiliensis (T) NP3 JX424772.1: 1.. 1519
Firmicutes Negativicutes Veillonellales-Selenomonadales Veillonellaceae Megasphaera Megasphaera massiliensis Otu0217
Megasphaera sp. DNF00872 KC297228.1: 1.. 1381
Megasphaera sp. KC297230.1: 1.. 1207 DNF00912
Anaeroglobus geminatus WWP SS10 G25 GU402021.1: 1.. 1001
Anaeroglobus geminatus WWP SS9 G18 GU402022.1: 1.. 1105
Firmicutes Negativicutes Veillonellales-Selenomonadales Veillonellaceae Anaeroglobus Anaeroglobus geminatus Otu0145
Veillonellaceae bacterium S3PF24 JX104009.1: 1.. 1431
Firmicutes Negativicutes Veillonellales-Selenomonadales Veillonellaceae Megasphaera Megasphaera stantonii Otu2142
Firmicutes Negativicutes Veillonellales-Selenomonadales Veillonellaceae Megasphaera Megasphaera 1 Otu0009
Veillonellaceae bacterium KF280299.1: 1.. 1235 DNF00751
Veillonellaceae bacterium S3PF10 JX104005.1: 1.. 1404

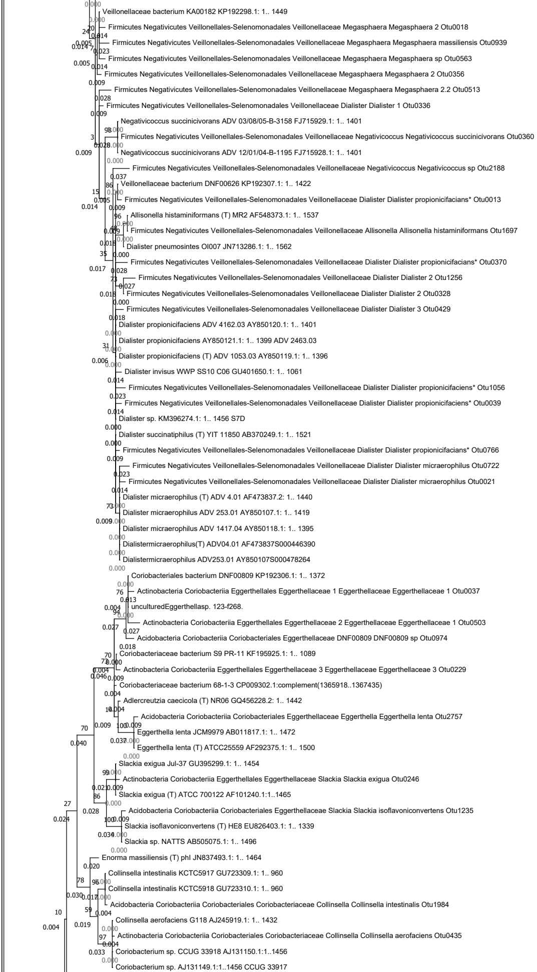
Veillonellaceae bacterium KA00182 KP192298.1: 1.. 1449
Firmicutes Negativicutes Veillonellales-Selenomonadales Veillonellaceae Megasphaera Megasphaera 2 Otu0018
Firmicutes Negativicutes Veillonellales-Selenomonadales Veillonellaceae Megasphaera Megasphaera massiliensis Otu0939
Firmicutes Negativicutes Veillonellales-Selenomonadales Veillonellaceae Megasphaera Megasphaera sp Otu0563
Firmicutes Negativicutes Veillonellales-Selenomonadales Veillonellaceae Megasphaera Megasphaera 2 Otu0356
Firmicutes Negativicutes Veillonellales-Selenomonadales Veillonellaceae Megasphaera Megasphaera 2.2 Otu0513
Firmicutes Negativicutes Veillonellales-Selenomonadales Veillonellaceae Dialister Dialister 1 Otu0336
Negativicoccus succinicivorans ADV 03/08/05-B-3158 FJ715929.1: 1.. 1401
Firmicutes Negativicutes Veillonellales-Selenomonadales Veillonellaceae Negativicoccus Negativicoccus succinicivorans Otu0360
Negativicoccus succinicivorans ADV 12/01/04-B-1195 FJ715928.1: 1.. 1401
Firmicutes Negativicutes Veillonellales-Selenomonadales Veillonellaceae Negativicoccus Negativicoccus sp Otu2188
Veillonellaceae bacterium DNF00626 KP192307.1: 1.. 1422
Firmicutes Negativicutes Veillonellales-Selenomonadales Veillonellaceae Dialister Dialister propionicifacians* Otu0013
Allisonella histaminiformans (T) MR2 AF548373.1: 1.. 1537
Firmicutes Negativicutes Veillonellales-Selenomonadales Veillonellaceae Allisonella Allisonella histaminiformans Otu1697
Dialister pneumosintes OI007 JN713286.1: 1.. 1562
Firmicutes Negativicutes Veillonellales-Selenomonadales Veillonellaceae Dialister Dialister propionicifacians* Otu0370
Firmicutes Negativicutes Veillonellales-Selenomonadales Veillonellaceae Dialister Dialister 2 Otu1256
Firmicutes Negativicutes Veillonellales-Selenomonadales Veillonellaceae Dialister Dialister 2 Otu0328
Firmicutes Negativicutes Veillonellales-Selenomonadales Veillonellaceae Dialister Dialister 3 Otu0429
Dialister propionicifaciens ADV 4162.03 AY850120.1: 1.. 1401
Dialister propionicifaciens AY850121.1: 1.. 1399 ADV 2463.03
Dialister propionicifaciens (T) ADV 1053.03 AY850119.1: 1.. 1396
Dialister invisus WWP SS10 C06 GU401650.1: 1.. 1061
Firmicutes Negativicutes Veillonellales-Selenomonadales Veillonellaceae Dialister Dialister propionicifaciens* Otu1056
Firmicutes Negativicutes Veillonellales-Selenomonadales Veillonellaceae Dialister Dialister propionicifaciens* Otu0039
Dialister sp. KM396274.1: 1.. 1456 S7D
Dialister succinatiphilus (T) YIT 11850 AB370249.1: 1.. 1521
Firmicutes Negativicutes Veillonellales-Selenomonadales Veillonellaceae Dialister Dialister propionicifacians* Otu0766
Firmicutes Negativicutes Veillonellales-Selenomonadales Veillonellaceae Dialister Dialister micraerophilus Otu0722
Firmicutes Negativicutes Veillonellales-Selenomonadales Veillonellaceae Dialister Dialister micraerophilus Otu0021
Dialister micraerophilus (T) ADV 4.01 AF473837.2: 1.. 1440
Dialister micraerophilus ADV 253.01 AY850107.1: 1.. 1419
Dialister micraerophilus ADV 1417.04 AY850118.1: 1.. 1395
Dialistermicraerophilus(T) ADV04.01 AF473837S000446390
Dialistermicraerophilus ADV253.01 AY850107S000478264
Coriobacteriales bacterium DNF00809 KP192306.1: 1.. 1372
Actinobacteria Coriobacteriia Eggerthellales Eggerthellaceae 1 Eggerthellaceae Eggerthellaceae 1 Otu0037
unculturedEggerthellasp. 123-f268.
Actinobacteria Coriobacteriia Eggerthellales Eggerthellaceae 2 Eggerthellaceae Eggerthellaceae 1 Otu0503
Acidobacteria Coriobacteriia Coriobacteriales Eggerthellaceae DNF00809 DNF00809 sp Otu0974
Coriobacteriaceae bacterium S9 PR-11 KF195925.1: 1.. 1089
Actinobacteria Coriobacteriia Eggerthellales Eggerthellaceae 3 Eggerthellaceae Eggerthellaceae 3 Otu0229
Coriobacteriaceae bacterium 68-1-3 CP009302.1:complement(1365918..1367435)
Adlercreutzia caecicola (T) NR06 GQ456228.2: 1.. 1442
Acidobacteria Coriobacteriia Coriobacteriales Eggerthellaceae Eggerthella Eggerthella lenta Otu2757
Eggerthella lenta JCM9979 AB011817.1: 1.. 1472
Eggerthella lenta (T) ATCC25559 AF292375.1: 1.. 1500
Slackia exigua Jul-37 GU395299.1: 1.. 1454
Actinobacteria Coriobacteriia Eggerthellales Eggerthellaceae Slackia Slackia exigua Otu0246
Slackia exigua (T) ATCC 700122 AF101240.1:1..1465
Acidobacteria Coriobacteriia Coriobacteriales Eggerthellaceae Slackia Slackia isoflavoniconvertens Otu1235
Slackia isoflavoniconvertens (T) HE8 EU826403.1: 1.. 1339
Slackia sp. NATTS AB505075.1: 1.. 1496
Enorma massiliensis (T) phI JN837493.1: 1.. 1464
Collinsella intestinalis KCTC5917 GU723309.1: 1.. 960
Collinsella intestinalis KCTC5918 GU723310.1: 1.. 960
Acidobacteria Coriobacteriia Coriobacteriales Coriobacteriaceae Collinsella Collinsella intestinalis Otu1984
Collinsella aerofaciens G118 AJ245919.1: 1.. 1432
Actinobacteria Coriobacteriia Coriobacteriales Coriobacteriaceae Collinsella Collinsella aerofaciens Otu0435
Coriobacterium sp. CCUG 33918 AJ131150.1:1..1456
Coriobacterium sp. AJ131149.1:1..1456 CCUG 33917

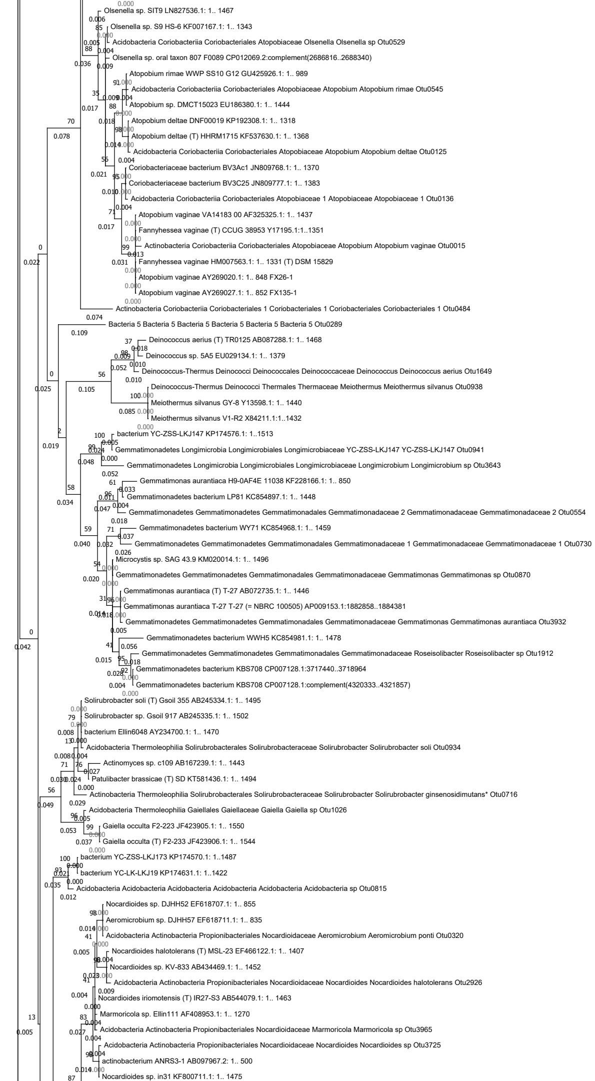
Olsenella sp. SIT9 LN827536.1: 1.. 1467
Olsenella sp. S9 HS-6 KF007167.1: 1.. 1343
Acidobacteria Coriobacteriia Coriobacteriales Atopobiaceae Olsenella Olsenella sp Otu0529
Olsenella sp. oral taxon 807 F0089 CP012069.2:complement(2686816..2688340)
Atopobium rimae WWP SS10 G12 GU425926.1: 1.. 989
Acidobacteria Coriobacteriia Coriobacteriales Atopobiaceae Atopobium Atopobium rimae Otu0545
Atopobium sp. DMCT15023 EU186380.1: 1.. 1444
Atopobium deltae DNF00019 KP192308.1: 1.. 1318
Atopobium deltae (T) HHRM1715 KF537630.1: 1.. 1368
Acidobacteria Coriobacteriia Coriobacteriales Atopobiaceae Atopobium Atopobium deltae Otu0125
Coriobacteriaceae bacterium BV3Ac1 JN809768.1: 1.. 1370
Coriobacteriaceae bacterium BV3C25 JN809777.1: 1.. 1383
Acidobacteria Coriobacteriia Coriobacteriales Atopobiaceae 1 Atopobiaceae Atopobiaceae 1 Otu0136
Atopobium vaginae VA14183 00 AF325325.1: 1.. 1437
Fannyhessea vaginae (T) CCUG 38953 Y17195.1:1..1351
Actinobacteria Coriobacteriia Coriobacteriales Atopobiaceae Atopobium Atopobium vaginae Otu0015
Fannyhessea vaginae HM007563.1: 1.. 1331 (T) DSM 15829
Atopobium vaginae AY269020.1: 1.. 848 FX26-1
Atopobium vaginae AY269027.1: 1.. 852 FX135-1
Actinobacteria Coriobacteriia Coriobacteriales 1 Coriobacteriales 1 Coriobacteriales Coriobacteriales 1 Otu0484
Bacteria 5 Bacteria 5 Bacteria 5 Bacteria 5 Bacteria 5 Bacteria 5 Otu0289
Deinococcus aerius (T) TR0125 AB087288.1: 1.. 1468
Deinococcus sp. 5A5 EU029134.1: 1.. 1379
Deinococcus-Thermus Deinococci Deinococcales Deinococcaceae Deinococcus Deinococcus aerius Otu1649
Deinococcus-Thermus Deinococci Thermales Thermaceae Meiothermus Meiothermus silvanus Otu0938
Meiothermus silvanus GY-8 Y13598.1: 1.. 1440
Meiothermus silvanus V1-R2 X84211.1:1..1432
bacterium YC-ZSS-LKJ147 KP174576.1: 1..1513
Gemmatimonadetes Longimicrobia Longimicrobiales Longimicrobiaceae YC-ZSS-LKJ147 YC-ZSS-LKJ147 Otu0941
Gemmatimonadetes Longimicrobia Longimicrobiales Longimicrobiaceae Longimicrobium Longimicrobium sp Otu3643
Gemmatimonas aurantiaca H9-0AF4E 11038 KF228166.1: 1.. 850
Gemmatimonadetes bacterium LP81 KC854897.1: 1.. 1448
Gemmatimonadetes Gemmatimonadetes Gemmatimonadales Gemmatimonadaceae 2 Gemmatimonadaceae Gemmatimonadaceae 2 Otu0554
Gemmatimonadetes bacterium WY71 KC854968.1: 1.. 1459
Gemmatimonadetes Gemmatimonadetes Gemmatimonadales Gemmatimonadaceae 1 Gemmatimonadaceae Gemmatimonadaceae 1 Otu0730
Microcystis sp. SAG 43.9 KM020014.1: 1.. 1496
Gemmatimonadetes Gemmatimonadetes Gemmatimonadales Gemmatimonadaceae Gemmatimonas Gemmatimonas sp Otu0870
Gemmatimonas aurantiaca (T) T-27 AB072735.1: 1.. 1446
Gemmatimonas aurantiaca T-27 T-27 (= NBRC 100505) AP009153.1:1882858..1884381
Gemmatimonadetes Gemmatimonadetes Gemmatimonadales Gemmatimonadaceae Gemmatimonas Gemmatimonas aurantiaca Otu3932
Gemmatimonadetes bacterium WWH5 KC854981.1: 1.. 1478
Gemmatimonadetes Gemmatimonadetes Gemmatimonadales Gemmatimonadaceae Roseisolibacter Roseisolibacter sp Otu1912
Gemmatimonadetes bacterium KBS708 CP007128.1:3717440..3718964
Gemmatimonadetes bacterium KBS708 CP007128.1:complement(4320333..4321857)
Solirubrobacter soli (T) Gsoil 355 AB245334.1: 1.. 1495
Solirubrobacter sp. Gsoil 917 AB245335.1: 1.. 1502
bacterium Ellin6048 AY234700.1: 1.. 1470
Acidobacteria Thermoleophilia Solirubrobacterales Solirubrobacteraceae Solirubrobacter Solirubrobacter soli Otu0934
Actinomyces sp. c109 AB167239.1: 1.. 1443
Patulibacter brassicae (T) SD KT581436.1: 1.. 1494
Actinobacteria Thermoleophilia Solirubrobacterales Solirubrobacteraceae Solirubrobacter Solirubrobacter ginsenosidimutans* Otu0716
Acidobacteria Thermoleophilia Gaiellales Gaiellaceae Gaiella Gaiella sp Otu1026
Gaiella occulta F2-223 JF423905.1: 1.. 1550
Gaiella occulta (T) F2-233 JF423906.1: 1.. 1544
bacterium YC-ZSS-LKJ173 KP174570.1: 1..1487
bacterium YC-LK-LKJ19 KP174631.1: 1..1422
Acidobacteria Acidobacteria Acidobacteria Acidobacteria Acidobacteria Acidobacteria sp Otu0815
Nocardioides sp. DJHH52 EF618707.1: 1.. 855
Aeromicrobium sp. DJHH57 EF618711.1: 1.. 835
Acidobacteria Actinobacteria Propionibacteriales Nocardioidaceae Aeromicrobium Aeromicrobium ponti Otu0320
Nocardioides halotolerans (T) MSL-23 EF466122.1: 1.. 1407
Nocardioides sp. KV-833 AB434469.1: 1.. 1452
Acidobacteria Actinobacteria Propionibacteriales Nocardioidaceae Nocardioides Nocardioides halotolerans Otu2926
Nocardioides iriomotensis (T) IR27-S3 AB544079.1: 1.. 1463
Marmoricola sp. Ellin111 AF408953.1: 1.. 1270
Acidobacteria Actinobacteria Propionibacteriales Nocardioidaceae Marmoricola Marmoricola sp Otu3965
Acidobacteria Actinobacteria Propionibacteriales Nocardioidaceae Nocardioides Nocardioides sp Otu3725
actinobacterium ANRS3-1 AB097967.2: 1.. 500
Nocardioides sp. in31 KF800711.1: 1.. 1475

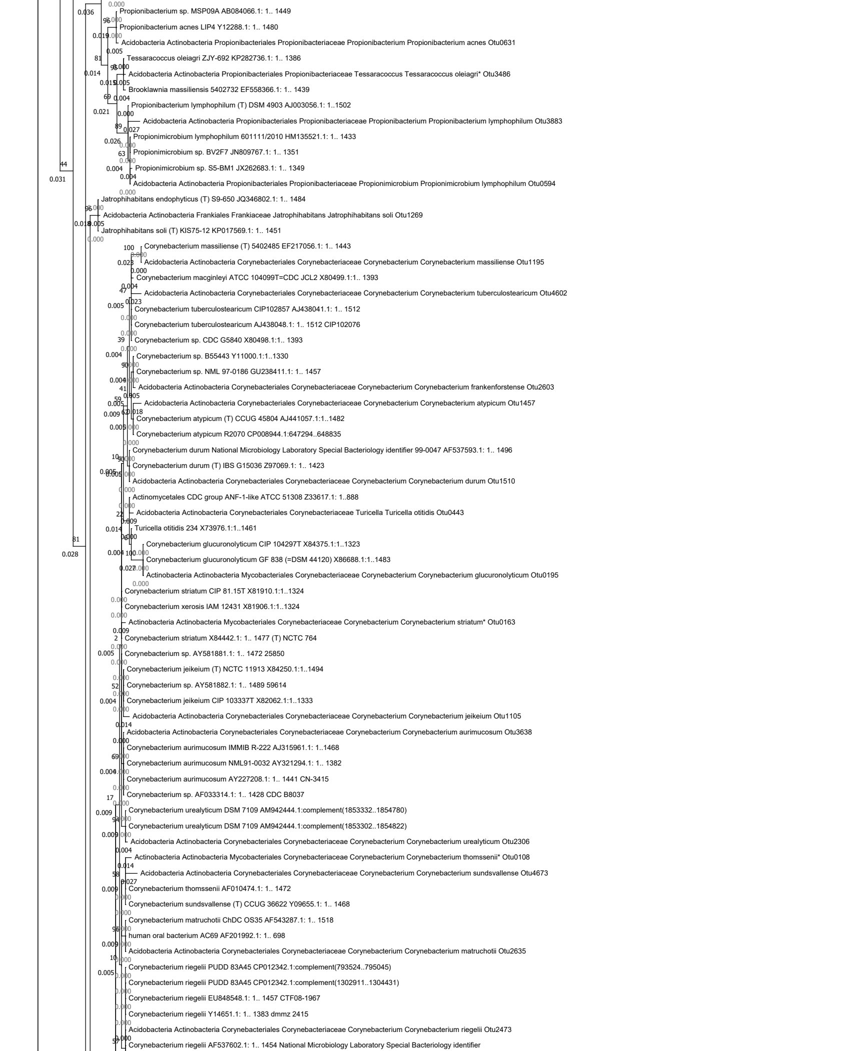
Propionibacterium sp. MSP09A AB084066.1: 1.. 1449
Propionibacterium acnes LIP4 Y12288.1: 1.. 1480
Acidobacteria Actinobacteria Propionibacteriales Propionibacteriaceae Propionibacterium Propionibacterium acnes Otu0631
Tessaracoccus oleiagri ZJY-692 KP282736.1: 1.. 1386
Acidobacteria Actinobacteria Propionibacteriales Propionibacteriaceae Tessaracoccus Tessaracoccus oleiagri* Otu3486
Brooklawnia massiliensis 5402732 EF558366.1: 1.. 1439
Propionibacterium lymphophilum (T) DSM 4903 AJ003056.1: 1..1502
Acidobacteria Actinobacteria Propionibacteriales Propionibacteriaceae Propionibacterium Propionibacterium lymphophilum Otu3883
Propionimicrobium lymphophilum 601111/2010 HM135521.1: 1.. 1433
Propionimicrobium sp. BV2F7 JN809767.1: 1.. 1351
Propionimicrobium sp. S5-BM1 JX262683.1: 1.. 1349
Acidobacteria Actinobacteria Propionibacteriales Propionibacteriaceae Propionimicrobium Propionimicrobium lymphophilum Otu0594
Jatrophihabitans endophyticus (T) S9-650 JQ346802.1: 1.. 1484
Acidobacteria Actinobacteria Frankiales Frankiaceae Jatrophihabitans Jatrophihabitans soli Otu1269
Jatrophihabitans soli (T) KIS75-12 KP017569.1: 1.. 1451
Corynebacterium massiliense (T) 5402485 EF217056.1: 1.. 1443
Acidobacteria Actinobacteria Corynebacteriales Corynebacteriaceae Corynebacterium Corynebacterium massiliense Otu1195
Corynebacterium macginleyi ATCC 104099T=CDC JCL2 X80499.1:1.. 1393
Acidobacteria Actinobacteria Corynebacteriales Corynebacteriaceae Corynebacterium Corynebacterium tuberculostearicum Otu4602
Corynebacterium tuberculostearicum CIP102857 AJ438041.1: 1.. 1512
Corynebacterium tuberculostearicum AJ438048.1: 1.. 1512 CIP102076
Corynebacterium sp. CDC G5840 X80498.1:1.. 1393
Corynebacterium sp. B55443 Y11000.1:1..1330
Corynebacterium sp. NML 97-0186 GU238411.1: 1.. 1457
Acidobacteria Actinobacteria Corynebacteriales Corynebacteriaceae Corynebacterium Corynebacterium frankenforstense Otu2603
Acidobacteria Actinobacteria Corynebacteriales Corynebacteriaceae Corynebacterium Corynebacterium atypicum Otu1457
Corynebacterium atypicum (T) CCUG 45804 AJ441057.1:1..1482
Corynebacterium atypicum R2070 CP008944.1:647294..648835
Corynebacterium durum National Microbiology Laboratory Special Bacteriology identifier 99-0047 AF537593.1: 1.. 1496
Corynebacterium durum (T) IBS G15036 Z97069.1: 1.. 1423
Acidobacteria Actinobacteria Corynebacteriales Corynebacteriaceae Corynebacterium Corynebacterium durum Otu1510
Actinomycetales CDC group ANF-1-like ATCC 51308 Z33617.1: 1..888
Acidobacteria Actinobacteria Corynebacteriales Corynebacteriaceae Turicella Turicella otitidis Otu0443
Turicella otitidis 234 X73976.1:1..1461
Corynebacterium glucuronolyticum CIP 104297T X84375.1:1..1323
Corynebacterium glucuronolyticum GF 838 (=DSM 44120) X86688.1:1..1483
Actinobacteria Actinobacteria Mycobacteriales Corynebacteriaceae Corynebacterium Corynebacterium glucuronolyticum Otu0195
Corynebacterium striatum CIP 81.15T X81910.1:1..1324
Corynebacterium xerosis IAM 12431 X81906.1:1..1324
Actinobacteria Actinobacteria Mycobacteriales Corynebacteriaceae Corynebacterium Corynebacterium striatum* Otu0163
Corynebacterium striatum X84442.1: 1.. 1477 (T) NCTC 764
Corynebacterium sp. AY581881.1: 1.. 1472 25850
Corynebacterium jeikeium (T) NCTC 11913 X84250.1:1..1494
Corynebacterium sp. AY581882.1: 1.. 1489 59614
Corynebacterium jeikeium CIP 103337T X82062.1:1..1333
Acidobacteria Actinobacteria Corynebacteriales Corynebacteriaceae Corynebacterium Corynebacterium jeikeium Otu1105
Acidobacteria Actinobacteria Corynebacteriales Corynebacteriaceae Corynebacterium Corynebacterium aurimucosum Otu3638
Corynebacterium aurimucosum IMMIB R-222 AJ315961.1: 1..1468
Corynebacterium aurimucosum NML91-0032 AY321294.1: 1.. 1382
Corynebacterium aurimucosum AY227208.1: 1.. 1441 CN-3415
Corynebacterium sp. AF033314.1: 1.. 1428 CDC B8037
Corynebacterium urealyticum DSM 7109 AM942444.1:complement(1853332..1854780)
Corynebacterium urealyticum DSM 7109 AM942444.1:complement(1853302..1854822)
Acidobacteria Actinobacteria Corynebacteriales Corynebacteriaceae Corynebacterium Corynebacterium urealyticum Otu2306
Actinobacteria Actinobacteria Mycobacteriales Corynebacteriaceae Corynebacterium Corynebacterium thomssenii* Otu0108
Acidobacteria Actinobacteria Corynebacteriales Corynebacteriaceae Corynebacterium Corynebacterium sundsvallense Otu4673
Corynebacterium thomssenii AF010474.1: 1.. 1472
Corynebacterium sundsvallense (T) CCUG 36622 Y09655.1: 1.. 1468
Corynebacterium matruchotii ChDC OS35 AF543287.1: 1.. 1518
human oral bacterium AC69 AF201992.1: 1.. 698
Acidobacteria Actinobacteria Corynebacteriales Corynebacteriaceae Corynebacterium Corynebacterium matruchotii Otu2635
Corynebacterium riegelii PUDD 83A45 CP012342.1:complement(793524..795045)
Corynebacterium riegelii PUDD 83A45 CP012342.1:complement(1302911..1304431)
Corynebacterium riegelii EU848548.1: 1.. 1457 CTF08-1967
Corynebacterium riegelii Y14651.1: 1.. 1383 dmmz 2415
Acidobacteria Actinobacteria Corynebacteriales Corynebacteriaceae Corynebacterium Corynebacterium riegelii Otu2473
Corynebacterium riegelii AF537602.1: 1.. 1454 National Microbiology Laboratory Special Bacteriology identifier

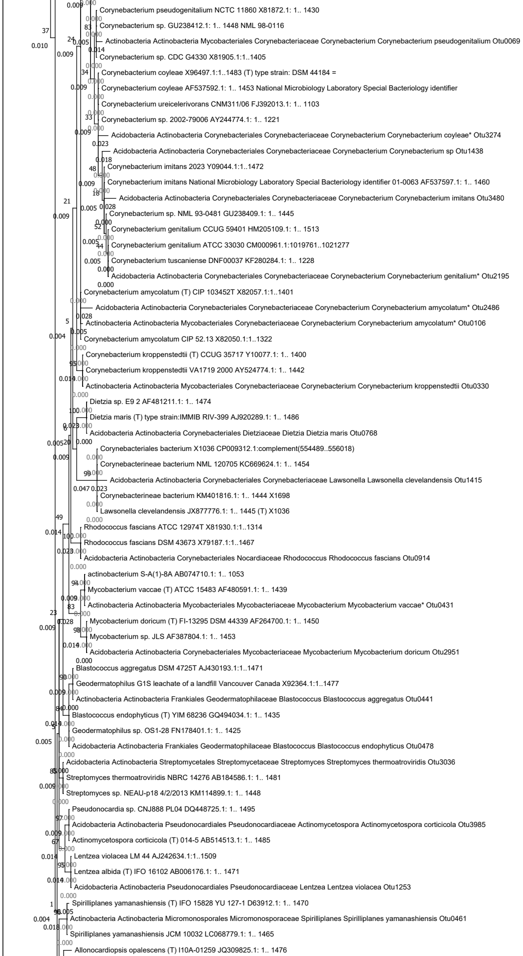
Corynebacterium pseudogenitalium NCTC 11860 X81872.1: 1.. 1430
Corynebacterium sp. GU238412.1: 1.. 1448 NML 98-0116
Actinobacteria Actinobacteria Mycobacteriales Corynebacteriaceae Corynebacterium Corynebacterium pseudogenitalium Otu0069
Corynebacterium sp. CDC G4330 X81905.1:1..1405
Corynebacterium coyleae X96497.1:1..1483 (T) type strain: DSM 44184 =
Corynebacterium coyleae AF537592.1: 1.. 1453 National Microbiology Laboratory Special Bacteriology identifier
Corynebacterium ureicelerivorans CNM311/06 FJ392013.1: 1.. 1103
Corynebacterium sp. 2002-79006 AY244774.1: 1.. 1221
Acidobacteria Actinobacteria Corynebacteriales Corynebacteriaceae Corynebacterium Corynebacterium coyleae* Otu3274
Acidobacteria Actinobacteria Corynebacteriales Corynebacteriaceae Corynebacterium Corynebacterium sp Otu1438
Corynebacterium imitans 2023 Y09044.1:1..1472
Corynebacterium imitans National Microbiology Laboratory Special Bacteriology identifier 01-0063 AF537597.1: 1.. 1460
Acidobacteria Actinobacteria Corynebacteriales Corynebacteriaceae Corynebacterium Corynebacterium imitans Otu3480
Corynebacterium sp. NML 93-0481 GU238409.1: 1.. 1445
Corynebacterium genitalium CCUG 59401 HM205109.1: 1.. 1513
Corynebacterium genitalium ATCC 33030 CM000961.1:1019761..1021277
Corynebacterium tuscaniense DNF00037 KF280284.1: 1.. 1228
Acidobacteria Actinobacteria Corynebacteriales Corynebacteriaceae Corynebacterium Corynebacterium genitalium* Otu2195
Corynebacterium amycolatum (T) CIP 103452T X82057.1:1..1401
Acidobacteria Actinobacteria Corynebacteriales Corynebacteriaceae Corynebacterium Corynebacterium amycolatum* Otu2486
Actinobacteria Actinobacteria Mycobacteriales Corynebacteriaceae Corynebacterium Corynebacterium amycolatum* Otu0106
Corynebacterium amycolatum CIP 52.13 X82050.1:1..1322
Corynebacterium kroppenstedtii (T) CCUG 35717 Y10077.1: 1.. 1400
Corynebacterium kroppenstedtii VA1719 2000 AY524774.1: 1.. 1442
Actinobacteria Actinobacteria Mycobacteriales Corynebacteriaceae Corynebacterium Corynebacterium kroppenstedtii Otu0330
Dietzia sp. E9 2 AF481211.1: 1.. 1474
Dietzia maris (T) type strain:IMMIB RIV-399 AJ920289.1: 1.. 1486
Acidobacteria Actinobacteria Corynebacteriales Dietziaceae Dietzia Dietzia maris Otu0768
Corynebacteriales bacterium X1036 CP009312.1:complement(554489..556018)
Corynebacterineae bacterium NML 120705 KC669624.1: 1.. 1454
Acidobacteria Actinobacteria Corynebacteriales Corynebacteriaceae Lawsonella Lawsonella clevelandensis Otu1415
Corynebacterineae bacterium KM401816.1: 1.. 1444 X1698
Lawsonella clevelandensis JX877776.1: 1.. 1445 (T) X1036
Rhodococcus fascians ATCC 12974T X81930.1:1..1314
Rhodococcus fascians DSM 43673 X79187.1:1..1467
Acidobacteria Actinobacteria Corynebacteriales Nocardiaceae Rhodococcus Rhodococcus fascians Otu0914
actinobacterium S-A(1)-8A AB074710.1: 1.. 1053
Mycobacterium vaccae (T) ATCC 15483 AF480591.1: 1.. 1439
Actinobacteria Actinobacteria Mycobacteriales Mycobacteriaceae Mycobacterium Mycobacterium vaccae* Otu0431
Mycobacterium doricum (T) FI-13295 DSM 44339 AF264700.1: 1.. 1450
Mycobacterium sp. JLS AF387804.1: 1.. 1453
Acidobacteria Actinobacteria Corynebacteriales Mycobacteriaceae Mycobacterium Mycobacterium doricum Otu2951
Blastococcus aggregatus DSM 4725T AJ430193.1:1..1471
Geodermatophilus G1S leachate of a landfill Vancouver Canada X92364.1:1..1477
Actinobacteria Actinobacteria Frankiales Geodermatophilaceae Blastococcus Blastococcus aggregatus Otu0441
Blastococcus endophyticus (T) YIM 68236 GQ494034.1: 1.. 1435
Geodermatophilus sp. OS1-28 FN178401.1: 1.. 1425
Acidobacteria Actinobacteria Frankiales Geodermatophilaceae Blastococcus Blastococcus endophyticus Otu0478
Acidobacteria Actinobacteria Streptomycetales Streptomycetaceae Streptomyces Streptomyces thermoatroviridis Otu3036
Streptomyces thermoatroviridis NBRC 14276 AB184586.1: 1.. 1481
Streptomyces sp. NEAU-p18 4/2/2013 KM114899.1: 1.. 1448
Pseudonocardia sp. CNJ888 PL04 DQ448725.1: 1.. 1495
Acidobacteria Actinobacteria Pseudonocardiales Pseudonocardiaceae Actinomycetospora Actinomycetospora corticicola Otu3985
Actinomycetospora corticicola (T) 014-5 AB514513.1: 1.. 1485
Lentzea violacea LM 44 AJ242634.1:1..1509
Lentzea albida (T) IFO 16102 AB006176.1: 1.. 1471
Acidobacteria Actinobacteria Pseudonocardiales Pseudonocardiaceae Lentzea Lentzea violacea Otu1253
Spirilliplanes yamanashiensis (T) IFO 15828 YU 127-1 D63912.1: 1.. 1470
Actinobacteria Actinobacteria Micromonosporales Micromonosporaceae Spirilliplanes Spirilliplanes yamanashiensis Otu0461
Spirilliplanes yamanashiensis JCM 10032 LC068779.1: 1.. 1465
Allonocardiopsis opalescens (T) I10A-01259 JQ309825.1: 1.. 1476

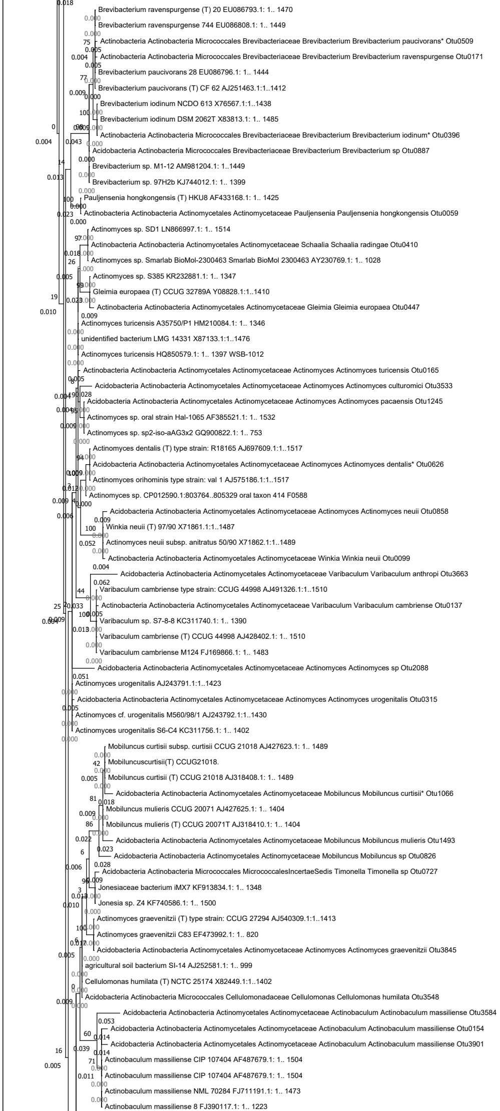
Brevibacterium ravenspurgense (T) 20 EU086793.1: 1.. 1470
Brevibacterium ravenspurgense 744 EU086808.1: 1.. 1449
Actinobacteria Actinobacteria Micrococcales Brevibacteriaceae Brevibacterium Brevibacterium paucivorans* Otu0509
Actinobacteria Actinobacteria Micrococcales Brevibacteriaceae Brevibacterium Brevibacterium ravenspurgense Otu0171
Brevibacterium paucivorans 28 EU086796.1: 1.. 1444
Brevibacterium paucivorans (T) CF 62 AJ251463.1:1..1412
Brevibacterium iodinum NCDO 613 X76567.1:1..1438
Brevibacterium iodinum DSM 2062T X83813.1: 1.. 1485
Actinobacteria Actinobacteria Micrococcales Brevibacteriaceae Brevibacterium Brevibacterium iodinum* Otu0396
Acidobacteria Actinobacteria Micrococcales Brevibacteriaceae Brevibacterium Brevibacterium sp Otu0887
Brevibacterium sp. M1-12 AM981204.1: 1..1449
Brevibacterium sp. 97H2b KJ744012.1: 1.. 1399
Pauljensenia hongkongensis (T) HKU8 AF433168.1: 1.. 1425
Actinobacteria Actinobacteria Actinomycetales Actinomycetaceae Pauljensenia Pauljensenia hongkongensis Otu0059
Actinomyces sp. SD1 LN866997.1: 1.. 1514
Actinobacteria Actinobacteria Actinomycetales Actinomycetaceae Schaalia Schaalia radingae Otu0410
Actinomyces sp. Smarlab BioMol-2300463 Smarlab BioMol 2300463 AY230769.1: 1.. 1028
Actinomyces sp. S385 KR232881.1: 1.. 1347
Gleimia europaea (T) CCUG 32789A Y08828.1:1..1410
Actinobacteria Actinobacteria Actinomycetales Actinomycetaceae Gleimia Gleimia europaea Otu0447
Actinomyces turicensis A35750/P1 HM210084.1: 1.. 1346
unidentified bacterium LMG 14331 X87133.1:1..1476
Actinomyces turicensis HQ850579.1: 1.. 1397 WSB-1012
Actinobacteria Actinobacteria Actinomycetales Actinomycetaceae Actinomyces Actinomyces turicensis Otu0165
Acidobacteria Actinobacteria Actinomycetales Actinomycetaceae Actinomyces Actinomyces culturomici Otu3533
Acidobacteria Actinobacteria Actinomycetales Actinomycetaceae Actinomyces Actinomyces pacaensis Otu1245
Actinomyces sp. oral strain Hal-1065 AF385521.1: 1.. 1532
Actinomyces sp. sp2-iso-aAG3x2 GQ900822.1: 1.. 753
Actinomyces dentalis (T) type strain: R18165 AJ697609.1:1..1517
Acidobacteria Actinobacteria Actinomycetales Actinomycetaceae Actinomyces Actinomyces dentalis* Otu0626
Actinomyces orihominis type strain: val 1 AJ575186.1:1..1517
Actinomyces sp. CP012590.1:803764..805329 oral taxon 414 F0588
Acidobacteria Actinobacteria Actinomycetales Actinomycetaceae Actinomyces Actinomyces neuii Otu0858
Winkia neuii (T) 97/90 X71861.1:1..1487
Actinomyces neuii subsp. anitratus 50/90 X71862.1:1..1489
Actinobacteria Actinobacteria Actinomycetales Actinomycetaceae Winkia Winkia neuii Otu0099
Acidobacteria Actinobacteria Actinomycetales Actinomycetaceae Varibaculum Varibaculum anthropi Otu3663
Varibaculum cambriense type strain: CCUG 44998 AJ491326.1:1..1510
Actinobacteria Actinobacteria Actinomycetales Actinomycetaceae Varibaculum Varibaculum cambriense Otu0137
Varibaculum sp. S7-8-8 KC311740.1: 1.. 1390
Varibaculum cambriense (T) CCUG 44998 AJ428402.1: 1.. 1510
Varibaculum cambriense M124 FJ169866.1: 1.. 1483
Acidobacteria Actinobacteria Actinomycetales Actinomycetaceae Actinomyces Actinomyces sp Otu2088
Actinomyces urogenitalis AJ243791.1:1..1423
Acidobacteria Actinobacteria Actinomycetales Actinomycetaceae Actinomyces Actinomyces urogenitalis Otu0315
Actinomyces cf. urogenitalis M560/98/1 AJ243792.1:1..1430
Actinomyces urogenitalis S6-C4 KC311756.1: 1.. 1402
Mobiluncus curtisii subsp. curtisii CCUG 21018 AJ427623.1: 1.. 1489
Mobiluncuscurtisii(T) CCUG21018.
Mobiluncus curtisii (T) CCUG 21018 AJ318408.1: 1.. 1489
Acidobacteria Actinobacteria Actinomycetales Actinomycetaceae Mobiluncus Mobiluncus curtisii* Otu1066
Mobiluncus mulieris CCUG 20071 AJ427625.1: 1.. 1404
Mobiluncus mulieris (T) CCUG 20071T AJ318410.1: 1.. 1404
Acidobacteria Actinobacteria Actinomycetales Actinomycetaceae Mobiluncus Mobiluncus mulieris Otu1493
Acidobacteria Actinobacteria Actinomycetales Actinomycetaceae Mobiluncus Mobiluncus sp Otu0826
Acidobacteria Actinobacteria Micrococcales MicrococcalesIncertaeSedis Timonella Timonella sp Otu0727
Jonesiaceae bacterium iMX7 KF913834.1: 1.. 1348
Jonesia sp. Z4 KF740586.1: 1.. 1500
Actinomyces graevenitzii (T) type strain: CCUG 27294 AJ540309.1:1..1413
Actinomyces graevenitzii C83 EF473992.1: 1.. 820
Acidobacteria Actinobacteria Actinomycetales Actinomycetaceae Actinomyces Actinomyces graevenitzii Otu3845
agricultural soil bacterium SI-14 AJ252581.1: 1.. 999
Cellulomonas humilata (T) NCTC 25174 X82449.1:1..1402
Acidobacteria Actinobacteria Micrococcales Cellulomonadaceae Cellulomonas Cellulomonas humilata Otu3548
Acidobacteria Actinobacteria Actinomycetales Actinomycetaceae Actinobaculum Actinobaculum massiliense Otu3584
Acidobacteria Actinobacteria Actinomycetales Actinomycetaceae Actinobaculum Actinobaculum massiliense Otu0154
Acidobacteria Actinobacteria Actinomycetales Actinomycetaceae Actinobaculum Actinobaculum massiliense Otu3901
Actinobaculum massiliense CIP 107404 AF487679.1: 1.. 1504
Actinobaculum massiliense CIP 107404 AF487679.1: 1.. 1504
Actinobaculum massiliense NML 70284 FJ711191.1: 1.. 1473
Actinobaculum massiliense 8 FJ390117.1: 1.. 1223

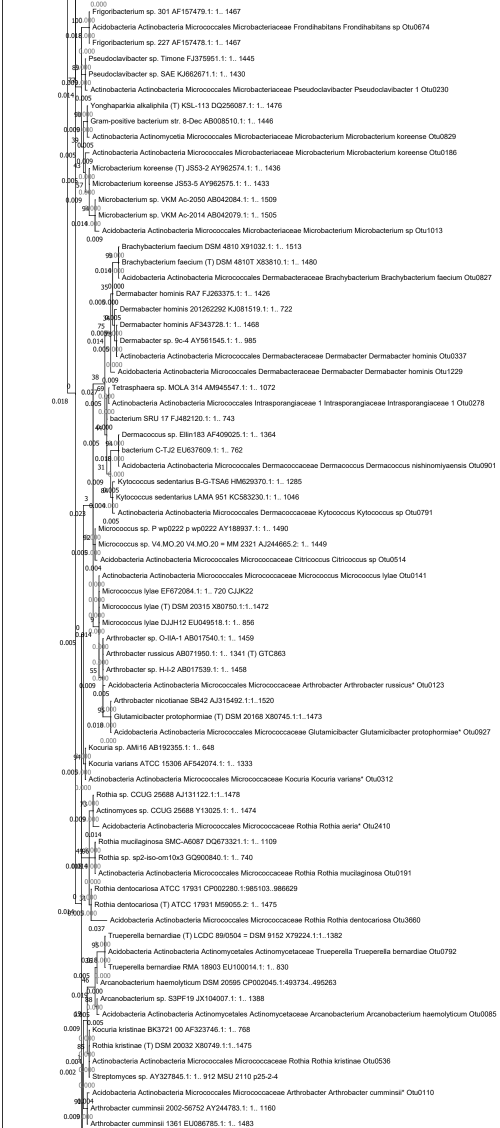
Frigoribacterium sp. 301 AF157479.1: 1.. 1467
Acidobacteria Actinobacteria Micrococcales Microbacteriaceae Frondihabitans Frondihabitans sp Otu0674
Frigoribacterium sp. 227 AF157478.1: 1.. 1467
Pseudoclavibacter sp. Timone FJ375951.1: 1.. 1445
Pseudoclavibacter sp. SAE KJ662671.1: 1.. 1430
Actinobacteria Actinobacteria Micrococcales Microbacteriaceae Pseudoclavibacter Pseudoclavibacter 1 Otu0230
Yonghaparkia alkaliphila (T) KSL-113 DQ256087.1: 1.. 1476
Gram-positive bacterium str. 8-Dec AB008510.1: 1.. 1446
Actinobacteria Actinomycetia Micrococcales Microbacteriaceae Microbacterium Microbacterium koreense Otu0829
Actinobacteria Actinobacteria Micrococcales Microbacteriaceae Microbacterium Microbacterium koreense Otu0186
Microbacterium koreense (T) JS53-2 AY962574.1: 1.. 1436
Microbacterium koreense JS53-5 AY962575.1: 1.. 1433
Microbacterium sp. VKM Ac-2050 AB042084.1: 1.. 1509
Microbacterium sp. VKM Ac-2014 AB042079.1: 1.. 1505
Acidobacteria Actinobacteria Micrococcales Microbacteriaceae Microbacterium Microbacterium sp Otu1013
Brachybacterium faecium DSM 4810 X91032.1: 1.. 1513
Brachybacterium faecium (T) DSM 4810T X83810.1: 1.. 1480
Acidobacteria Actinobacteria Micrococcales Dermabacteraceae Brachybacterium Brachybacterium faecium Otu0827
Dermabacter hominis RA7 FJ263375.1: 1.. 1426
Dermabacter hominis 201262292 KJ081519.1: 1.. 722
Dermabacter hominis AF343728.1: 1.. 1468
Dermabacter sp. 9c-4 AY561545.1: 1.. 985
Actinobacteria Actinobacteria Micrococcales Dermabacteraceae Dermabacter Dermabacter hominis Otu0337
Acidobacteria Actinobacteria Micrococcales Dermabacteraceae Dermabacter Dermabacter hominis Otu1229
Tetrasphaera sp. MOLA 314 AM945547.1: 1.. 1072
Actinobacteria Actinobacteria Micrococcales Intrasporangiaceae 1 Intrasporangiaceae Intrasporangiaceae 1 Otu0278
bacterium SRU 17 FJ482120.1: 1.. 743
Dermacoccus sp. Ellin183 AF409025.1: 1.. 1364
bacterium C-TJ2 EU637609.1: 1.. 762
Acidobacteria Actinobacteria Micrococcales Dermacoccaceae Dermacoccus Dermacoccus nishinomiyaensis Otu0901
Kytococcus sedentarius B-G-TSA6 HM629370.1: 1.. 1285
Kytococcus sedentarius LAMA 951 KC583230.1: 1.. 1046
Actinobacteria Actinobacteria Micrococcales Dermacoccaceae Kytococcus Kytococcus sp Otu0791
Micrococcus sp. P wp0222 p wp0222 AY188937.1: 1.. 1490
Micrococcus sp. V4.MO.20 V4.MO.20 = MM 2321 AJ244665.2: 1.. 1449
Acidobacteria Actinobacteria Micrococcales Micrococcaceae Citricoccus Citricoccus sp Otu0514
Actinobacteria Actinobacteria Micrococcales Micrococcaceae Micrococcus Micrococcus lylae Otu0141
Micrococcus lylae EF672084.1: 1.. 720 CJJK22
Micrococcus lylae (T) DSM 20315 X80750.1:1..1472
Micrococcus lylae DJJH12 EU049518.1: 1.. 856
Arthrobacter sp. O-IIA-1 AB017540.1: 1.. 1459
Arthrobacter russicus AB071950.1: 1.. 1341 (T) GTC863
Arthrobacter sp. H-I-2 AB017539.1: 1.. 1458
Acidobacteria Actinobacteria Micrococcales Micrococcaceae Arthrobacter Arthrobacter russicus* Otu0123
Arthrobacter nicotianae SB42 AJ315492.1:1..1520
Glutamicibacter protophormiae (T) DSM 20168 X80745.1:1..1473
Acidobacteria Actinobacteria Micrococcales Micrococcaceae Glutamicibacter Glutamicibacter protophormiae* Otu0927
Kocuria sp. AMi16 AB192355.1: 1.. 648
Kocuria varians ATCC 15306 AF542074.1: 1.. 1333
Actinobacteria Actinobacteria Micrococcales Micrococcaceae Kocuria Kocuria varians* Otu0312
Rothia sp. CCUG 25688 AJ131122.1:1..1478
Actinomyces sp. CCUG 25688 Y13025.1: 1.. 1474
Acidobacteria Actinobacteria Micrococcales Micrococcaceae Rothia Rothia aeria* Otu2410
Rothia mucilaginosa SMC-A6087 DQ673321.1: 1.. 1109
Rothia sp. sp2-iso-om10x3 GQ900840.1: 1.. 740
Actinobacteria Actinobacteria Micrococcales Micrococcaceae Rothia Rothia mucilaginosa Otu0191
Rothia dentocariosa ATCC 17931 CP002280.1:985103..986629
Rothia dentocariosa (T) ATCC 17931 M59055.2: 1.. 1475
Acidobacteria Actinobacteria Micrococcales Micrococcaceae Rothia Rothia dentocariosa Otu3660
Trueperella bernardiae (T) LCDC 89/0504 = DSM 9152 X79224.1:1..1382
Acidobacteria Actinobacteria Actinomycetales Actinomycetaceae Trueperella Trueperella bernardiae Otu0792
Trueperella bernardiae RMA 18903 EU100014.1: 1.. 830
Arcanobacterium haemolyticum DSM 20595 CP002045.1:493734..495263
Arcanobacterium sp. S3PF19 JX104007.1: 1.. 1388
Acidobacteria Actinobacteria Actinomycetales Actinomycetaceae Arcanobacterium Arcanobacterium haemolyticum Otu0085
Kocuria kristinae BK3721 00 AF323746.1: 1.. 768
Rothia kristinae (T) DSM 20032 X80749.1:1..1475
Actinobacteria Actinobacteria Micrococcales Micrococcaceae Rothia Rothia kristinae Otu0536
Streptomyces sp. AY327845.1: 1.. 912 MSU 2110 p25-2-4
Acidobacteria Actinobacteria Micrococcales Micrococcaceae Arthrobacter Arthrobacter cumminsii* Otu0110
Arthrobacter cumminsii 2002-56752 AY244783.1: 1.. 1160
Arthrobacter cumminsii 1361 EU086785.1: 1.. 1483

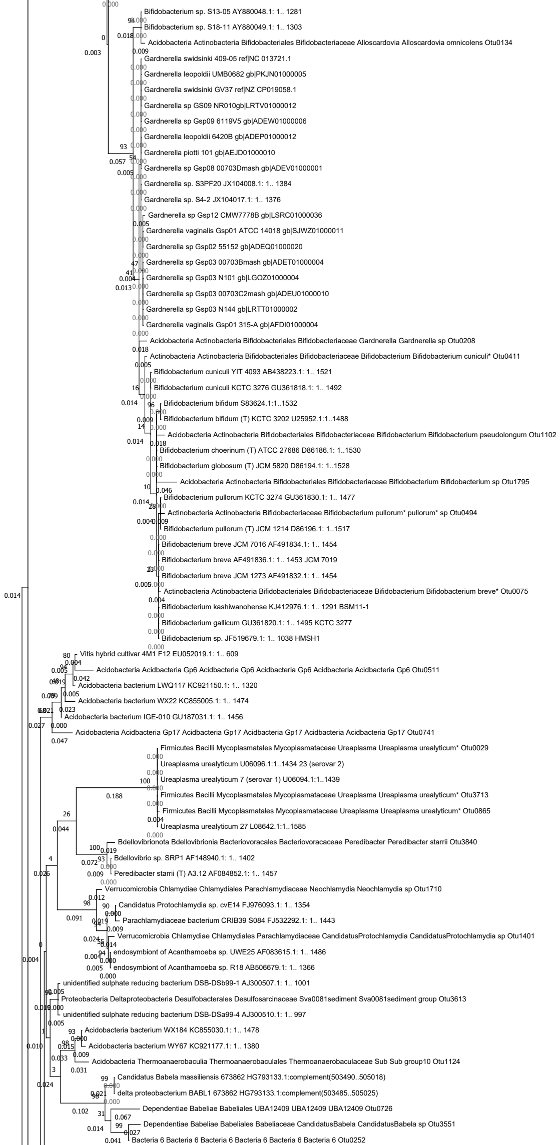

Bifidobacterium sp. S13-05 AY880048.1: 1.. 1281
Bifidobacterium sp. S18-11 AY880049.1: 1.. 1303
Acidobacteria Actinobacteria Bifidobacteriales Bifidobacteriaceae Alloscardovia Alloscardovia omnicolens Otu0134
Gardnerella swidsinki 409-05 ref|NC 013721.1
Gardnerella leopoldii UMB0682 gb|PKJN01000005
Gardnerella swidsinki GV37 ref|NZ CP019058.1
Gardnerella sp GS09 NR010gb|LRTV01000012
Gardnerella sp Gsp09 6119V5 gb|ADEW01000006
Gardnerella leopoldii 6420B gb|ADEP01000012
Gardnerella piotti 101 gb|AEJD01000010
Gardnerella sp Gsp08 00703Dmash gb|ADEV01000001
Gardnerella sp. S3PF20 JX104008.1: 1.. 1384
Gardnerella sp. S4-2 JX104017.1: 1.. 1376
Gardnerella sp Gsp12 CMW7778B gb|LSRC01000036
Gardnerella vaginalis Gsp01 ATCC 14018 gb|SJWZ01000011
Gardnerella sp Gsp02 55152 gb|ADEQ01000020
Gardnerella sp Gsp03 00703Bmash gb|ADET01000004
Gardnerella sp Gsp03 N101 gb|LGOZ01000004
Gardnerella sp Gsp03 00703C2mash gb|ADEU01000010
Gardnerella sp Gsp03 N144 gb|LRTT01000002
Gardnerella vaginalis Gsp01 315-A gb|AFDI01000004
Acidobacteria Actinobacteria Bifidobacteriales Bifidobacteriaceae Gardnerella Gardnerella sp Otu0208
Actinobacteria Actinobacteria Bifidobacteriales Bifidobacteriaceae Bifidobacterium Bifidobacterium cuniculi* Otu0411
Bifidobacterium cuniculi YIT 4093 AB438223.1: 1.. 1521
Bifidobacterium cuniculi KCTC 3276 GU361818.1: 1.. 1492
Bifidobacterium bifidum S83624.1:1..1532
Bifidobacterium bifidum (T) KCTC 3202 U25952.1:1..1488
Acidobacteria Actinobacteria Bifidobacteriales Bifidobacteriaceae Bifidobacterium Bifidobacterium pseudolongum Otu1102
Bifidobacterium choerinum (T) ATCC 27686 D86186.1: 1..1530
Bifidobacterium globosum (T) JCM 5820 D86194.1: 1..1528
Acidobacteria Actinobacteria Bifidobacteriales Bifidobacteriaceae Bifidobacterium Bifidobacterium sp Otu1795
Bifidobacterium pullorum KCTC 3274 GU361830.1: 1.. 1477
Actinobacteria Actinobacteria Bifidobacteriaceae Bifidobacterium pullorum* pullorum* sp Otu0494
Bifidobacterium pullorum (T) JCM 1214 D86196.1: 1..1517
Bifidobacterium breve JCM 7016 AF491834.1: 1.. 1454
Bifidobacterium breve AF491836.1: 1.. 1453 JCM 7019
Bifidobacterium breve JCM 1273 AF491832.1: 1.. 1454
Actinobacteria Actinobacteria Bifidobacteriales Bifidobacteriaceae Bifidobacterium Bifidobacterium breve* Otu0075
Bifidobacterium kashiwanohense KJ412976.1: 1.. 1291 BSM11-1
Bifidobacterium gallicum GU361820.1: 1.. 1495 KCTC 3277
Bifidobacterium sp. JF519679.1: 1.. 1038 HMSH1
Vitis hybrid cultivar 4M1 F12 EU052019.1: 1.. 609
Acidobacteria Acidbacteria Gp6 Acidbacteria Gp6 Acidbacteria Gp6 Acidbacteria Acidbacteria Gp6 Otu0511
Acidobacteria bacterium LWQ117 KC921150.1: 1.. 1320
Acidobacteria bacterium WX22 KC855005.1: 1.. 1474
Acidobacteria bacterium IGE-010 GU187031.1: 1.. 1456
Acidobacteria Acidbacteria Gp17 Acidbacteria Gp17 Acidbacteria Gp17 Acidbacteria Acidbacteria Gp17 Otu0741
Firmicutes Bacilli Mycoplasmatales Mycoplasmataceae Ureaplasma Ureaplasma urealyticum* Otu0029
Ureaplasma urealyticum U06096.1:1..1434 23 (serovar 2)
Ureaplasma urealyticum 7 (serovar 1) U06094.1:1..1439
Firmicutes Bacilli Mycoplasmatales Mycoplasmataceae Ureaplasma Ureaplasma urealyticum* Otu3713
Firmicutes Bacilli Mycoplasmatales Mycoplasmataceae Ureaplasma Ureaplasma urealyticum* Otu0865
Ureaplasma urealyticum 27 L08642.1:1..1585
Bdellovibrionota Bdellovibrionia Bacteriovoracales Bacteriovoracaceae Peredibacter Peredibacter starrii Otu3840
Bdellovibrio sp. SRP1 AF148940.1: 1.. 1402
Peredibacter starrii (T) A3.12 AF084852.1: 1.. 1457
Verrucomicrobia Chlamydiae Chlamydiales Parachlamydiaceae Neochlamydia Neochlamydia sp Otu1710
Candidatus Protochlamydia sp. cvE14 FJ976093.1: 1.. 1354
Parachlamydiaceae bacterium CRIB39 S084 FJ532292.1: 1.. 1443
Verrucomicrobia Chlamydiae Chlamydiales Parachlamydiaceae CandidatusProtochlamydia CandidatusProtochlamydia sp Otu1401
endosymbiont of Acanthamoeba sp. UWE25 AF083615.1: 1.. 1486
endosymbiont of Acanthamoeba sp. R18 AB506679.1: 1.. 1366
unidentified sulphate reducing bacterium DSB-DSb99-1 AJ300507.1: 1.. 1001
Proteobacteria Deltaproteobacteria Desulfobacterales Desulfosarcinaceae Sva0081sediment Sva0081sediment group Otu3613
unidentified sulphate reducing bacterium DSB-DSa99-4 AJ300510.1: 1.. 997
Acidobacteria bacterium WX184 KC855030.1: 1.. 1478
Acidobacteria bacterium WY67 KC921177.1: 1.. 1380
Acidobacteria Thermoanaerobaculia Thermoanaerobaculales Thermoanaerobaculaceae Sub Sub group10 Otu1124
Candidatus Babela massiliensis 673862 HG793133.1:complement(503490..505018)
delta proteobacterium BABL1 673862 HG793133.1:complement(503485..505025)
Dependentiae Babeliae Babeliales UBA12409 UBA12409 UBA12409 Otu0726
Dependentiae Babeliae Babeliales Babeliaceae CandidatusBabela CandidatusBabela sp Otu3551
Bacteria 6 Bacteria 6 Bacteria 6 Bacteria 6 Bacteria 6 Bacteria 6 Otu0252

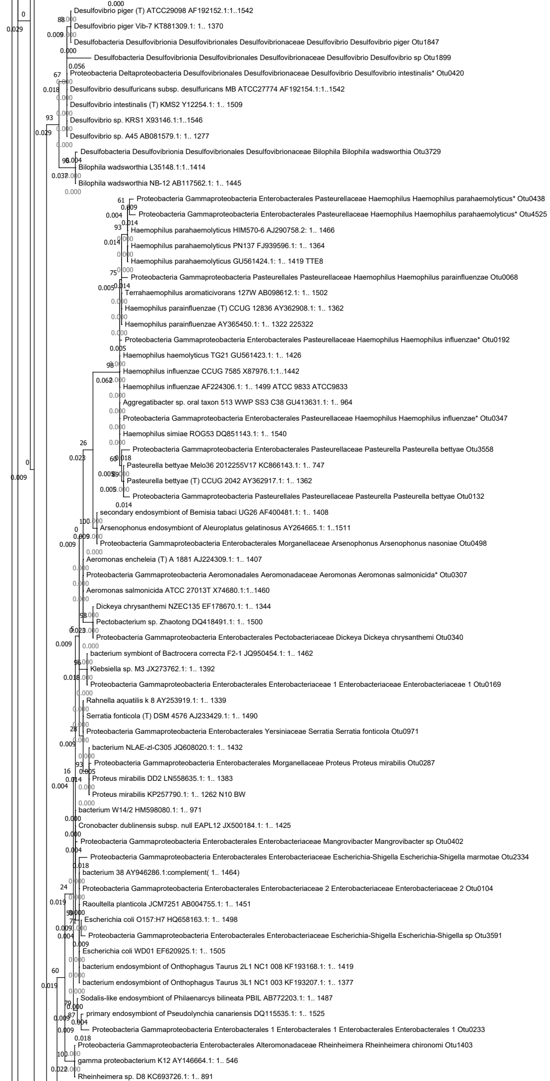

Desulfovibrio piger (T) ATCC29098 AF192152.1:1..1542
Desulfovibrio piger Vib-7 KT881309.1: 1.. 1370
Desulfobacteria Desulfovibrionia Desulfovibrionales Desulfovibrionaceae Desulfovibrio Desulfovibrio piger Otu1847
Desulfobacteria Desulfovibrionia Desulfovibrionales Desulfovibrionaceae Desulfovibrio Desulfovibrio sp Otu1899
Proteobacteria Deltaproteobacteria Desulfovibrionales Desulfovibrionaceae Desulfovibrio Desulfovibrio intestinalis* Otu0420
Desulfovibrio desulfuricans subsp. desulfuricans MB ATCC27774 AF192154.1:1..1542
Desulfovibrio intestinalis (T) KMS2 Y12254.1: 1.. 1509
Desulfovibrio sp. KRS1 X93146.1:1..1546
Desulfovibrio sp. A45 AB081579.1: 1.. 1277
Desulfobacteria Desulfovibrionia Desulfovibrionales Desulfovibrionaceae Bilophila Bilophila wadsworthia Otu3729
Bilophila wadsworthia L35148.1:1..1414
Bilophila wadsworthia NB-12 AB117562.1: 1.. 1445
Proteobacteria Gammaproteobacteria Enterobacterales Pasteurellaceae Haemophilus Haemophilus parahaemolyticus* Otu0438
Proteobacteria Gammaproteobacteria Enterobacterales Pasteurellaceae Haemophilus Haemophilus parahaemolyticus* Otu4525
Haemophilus parahaemolyticus HIM570-6 AJ290758.2: 1.. 1466
Haemophilus parahaemolyticus PN137 FJ939596.1: 1.. 1364
Haemophilus parahaemolyticus GU561424.1: 1.. 1419 TTE8
Proteobacteria Gammaproteobacteria Pasteurellales Pasteurellaceae Haemophilus Haemophilus parainfluenzae Otu0068
Terrahaemophilus aromaticivorans 127W AB098612.1: 1.. 1502
Haemophilus parainfluenzae (T) CCUG 12836 AY362908.1: 1.. 1362
Haemophilus parainfluenzae AY365450.1: 1.. 1322 225322
Proteobacteria Gammaproteobacteria Enterobacterales Pasteurellaceae Haemophilus Haemophilus influenzae* Otu0192
Haemophilus haemolyticus TG21 GU561423.1: 1.. 1426
Haemophilus influenzae CCUG 7585 X87976.1:1..1442
Haemophilus influenzae AF224306.1: 1.. 1499 ATCC 9833 ATCC9833
Aggregatibacter sp. oral taxon 513 WWP SS3 C38 GU413631.1: 1.. 964
Proteobacteria Gammaproteobacteria Enterobacterales Pasteurellaceae Haemophilus Haemophilus influenzae* Otu0347
Haemophilus simiae ROG53 DQ851143.1: 1.. 1540
Proteobacteria Gammaproteobacteria Enterobacterales Pasteurellaceae Pasteurella Pasteurella bettyae Otu3558
Pasteurella bettyae Melo36 2012255V17 KC866143.1: 1.. 747
Pasteurella bettyae (T) CCUG 2042 AY362917.1: 1.. 1362
Proteobacteria Gammaproteobacteria Pasteurellales Pasteurellaceae Pasteurella Pasteurella bettyae Otu0132
secondary endosymbiont of Bemisia tabaci UG26 AF400481.1: 1.. 1408
Arsenophonus endosymbiont of Aleuroplatus gelatinosus AY264665.1: 1..1511
Proteobacteria Gammaproteobacteria Enterobacterales Morganellaceae Arsenophonus Arsenophonus nasoniae Otu0498
Aeromonas encheleia (T) A 1881 AJ224309.1: 1.. 1407
Proteobacteria Gammaproteobacteria Aeromonadales Aeromonadaceae Aeromonas Aeromonas salmonicida* Otu0307
Aeromonas salmonicida ATCC 27013T X74680.1:1..1460
Dickeya chrysanthemi NZEC135 EF178670.1: 1.. 1344
Pectobacterium sp. Zhaotong DQ418491.1: 1.. 1500
Proteobacteria Gammaproteobacteria Enterobacterales Pectobacteriaceae Dickeya Dickeya chrysanthemi Otu0340
bacterium symbiont of Bactrocera correcta F2-1 JQ950454.1: 1.. 1462
Klebsiella sp. M3 JX273762.1: 1.. 1392
Proteobacteria Gammaproteobacteria Enterobacterales Enterobacteriaceae 1 Enterobacteriaceae Enterobacteriaceae 1 Otu0169
Rahnella aquatilis k 8 AY253919.1: 1.. 1339
Serratia fonticola (T) DSM 4576 AJ233429.1: 1.. 1490
Proteobacteria Gammaproteobacteria Enterobacterales Yersiniaceae Serratia Serratia fonticola Otu0971
bacterium NLAE-zl-C305 JQ608020.1: 1.. 1432
Proteobacteria Gammaproteobacteria Enterobacterales Morganellaceae Proteus Proteus mirabilis Otu0287
Proteus mirabilis DD2 LN558635.1: 1.. 1383
Proteus mirabilis KP257790.1: 1.. 1262 N10 BW
bacterium W14/2 HM598080.1: 1.. 971
Cronobacter dublinensis subsp. null EAPL12 JX500184.1: 1.. 1425
Proteobacteria Gammaproteobacteria Enterobacterales Enterobacteriaceae Mangrovibacter Mangrovibacter sp Otu0402
Proteobacteria Gammaproteobacteria Enterobacterales Enterobacteriaceae Escherichia-Shigella Escherichia-Shigella marmotae Otu2334
bacterium 38 AY946286.1:complement(1.. 1464)
Proteobacteria Gammaproteobacteria Enterobacterales Enterobacteriaceae 2 Enterobacteriaceae Enterobacteriaceae 2 Otu0104
Raoultella planticola JCM7251 AB004755.1: 1.. 1451
Escherichia coli O157:H7 HQ658163.1: 1.. 1498
Proteobacteria Gammaproteobacteria Enterobacterales Enterobacteriaceae Escherichia-Shigella Escherichia-Shigella sp Otu3591
Escherichia coli WD01 EF620925.1: 1.. 1505
bacterium endosymbiont of Onthophagus Taurus 2L1 NC1 008 KF193168.1: 1.. 1419
bacterium endosymbiont of Onthophagus Taurus 3L1 NC1 003 KF193207.1: 1.. 1377
Sodalis-like endosymbiont of Philaenarcys bilineata PBIL AB772203.1: 1.. 1487
primary endosymbiont of Pseudolynchia canariensis DQ115535.1: 1.. 1525
Proteobacteria Gammaproteobacteria Enterobacterales 1 Enterobacterales 1 Enterobacterales Enterobacterales 1 Otu0233
Proteobacteria Gammaproteobacteria Enterobacterales Alteromonadaceae Rheinheimera Rheinheimera chironomi Otu1403
gamma proteobacterium K12 AY146664.1: 1.. 546
Rheinheimera sp. D8 KC693726.1: 1.. 891

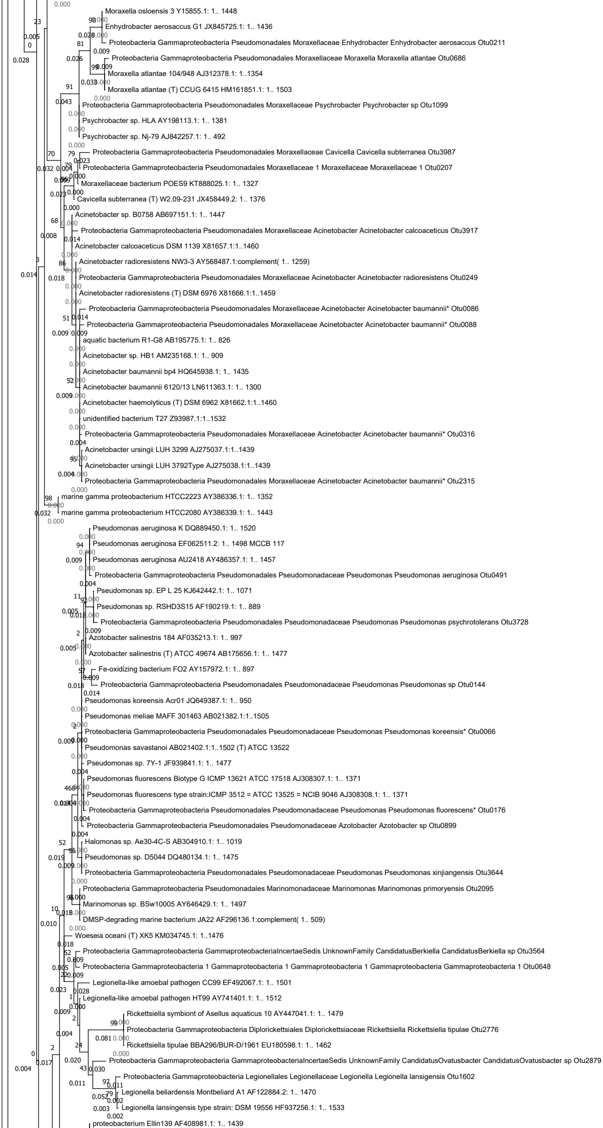
Moraxella osloensis 3 Y15855.1: 1.. 1448
Enhydrobacter aerosaccus G1 JX845725.1: 1.. 1436
Proteobacteria Gammaproteobacteria Pseudomonadales Moraxellaceae Enhydrobacter Enhydrobacter aerosaccus Otu0211
Proteobacteria Gammaproteobacteria Pseudomonadales Moraxellaceae Moraxella Moraxella atlantae Otu0686
Moraxella atlantae 104/948 AJ312378.1: 1..1354
Moraxella atlantae (T) CCUG 6415 HM161851.1: 1.. 1503
Proteobacteria Gammaproteobacteria Pseudomonadales Moraxellaceae Psychrobacter Psychrobacter sp Otu1099
Psychrobacter sp. HLA AY198113.1: 1.. 1381
Psychrobacter sp. Nj-79 AJ842257.1: 1.. 492
Proteobacteria Gammaproteobacteria Pseudomonadales Moraxellaceae Cavicella Cavicella subterranea Otu3987
Proteobacteria Gammaproteobacteria Pseudomonadales Moraxellaceae 1 Moraxellaceae Moraxellaceae 1 Otu0207
Moraxellaceae bacterium POES9 KT888025.1: 1.. 1327
Cavicella subterranea (T) W2.09-231 JX458449.2: 1.. 1376
Acinetobacter sp. B0758 AB697151.1: 1.. 1447
Proteobacteria Gammaproteobacteria Pseudomonadales Moraxellaceae Acinetobacter Acinetobacter calcoaceticus Otu3917
Acinetobacter calcoaceticus DSM 1139 X81657.1:1..1460
Acinetobacter radioresistens NW3-3 AY568487.1:complement(1.. 1259)
Proteobacteria Gammaproteobacteria Pseudomonadales Moraxellaceae Acinetobacter Acinetobacter radioresistens Otu0249
Acinetobacter radioresistens (T) DSM 6976 X81666.1:1..1459
Proteobacteria Gammaproteobacteria Pseudomonadales Moraxellaceae Acinetobacter Acinetobacter baumannii* Otu0086
Proteobacteria Gammaproteobacteria Pseudomonadales Moraxellaceae Acinetobacter Acinetobacter baumannii* Otu0088
aquatic bacterium R1-G8 AB195775.1: 1.. 826
Acinetobacter sp. HB1 AM235168.1: 1.. 909
Acinetobacter baumannii bp4 HQ645938.1: 1.. 1435
Acinetobacter baumannii 6120/13 LN611363.1: 1.. 1300
Acinetobacter haemolyticus (T) DSM 6962 X81662.1:1..1460
unidentified bacterium T27 Z93987.1:1..1532
Proteobacteria Gammaproteobacteria Pseudomonadales Moraxellaceae Acinetobacter Acinetobacter baumannii* Otu0316
Acinetobacter ursingii LUH 3299 AJ275037.1:1..1439
Acinetobacter ursingii LUH 3792Type AJ275038.1:1..1439
Proteobacteria Gammaproteobacteria Pseudomonadales Moraxellaceae Acinetobacter Acinetobacter baumannii* Otu2315
marine gamma proteobacterium HTCC2223 AY386336.1: 1.. 1352
marine gamma proteobacterium HTCC2080 AY386339.1: 1.. 1443
Pseudomonas aeruginosa K DQ889450.1: 1.. 1520
Pseudomonas aeruginosa EF062511.2: 1.. 1498 MCCB 117
Pseudomonas aeruginosa AU2418 AY486357.1: 1.. 1457
Proteobacteria Gammaproteobacteria Pseudomonadales Pseudomonadaceae Pseudomonas Pseudomonas aeruginosa Otu0491
Pseudomonas sp. EP L 25 KJ642442.1: 1.. 1071
Pseudomonas sp. RSHD3S15 AF190219.1: 1.. 889
Proteobacteria Gammaproteobacteria Pseudomonadales Pseudomonadaceae Pseudomonas Pseudomonas psychrotolerans Otu3728
Azotobacter salinestris 184 AF035213.1: 1.. 997
Azotobacter salinestris (T) ATCC 49674 AB175656.1: 1.. 1477
Fe-oxidizing bacterium FO2 AY157972.1: 1.. 897
Proteobacteria Gammaproteobacteria Pseudomonadales Pseudomonadaceae Pseudomonas Pseudomonas sp Otu0144
Pseudomonas koreensis Acr01 JQ649387.1: 1.. 950
Pseudomonas meliae MAFF 301463 AB021382.1:1..1505
Proteobacteria Gammaproteobacteria Pseudomonadales Pseudomonadaceae Pseudomonas Pseudomonas koreensis* Otu0066
Pseudomonas savastanoi AB021402.1:1..1502 (T) ATCC 13522
Pseudomonas sp. 7Y-1 JF939841.1: 1.. 1477
Pseudomonas fluorescens Biotype G ICMP 13621 ATCC 17518 AJ308307.1: 1.. 1371
Pseudomonas fluorescens type strain:ICMP 3512 = ATCC 13525 = NCIB 9046 AJ308308.1: 1.. 1371
Proteobacteria Gammaproteobacteria Pseudomonadales Pseudomonadaceae Pseudomonas Pseudomonas fluorescens* Otu0176
Proteobacteria Gammaproteobacteria Pseudomonadales Pseudomonadaceae Azotobacter Azotobacter sp Otu0899
Halomonas sp. Ae30-4C-S AB304910.1: 1.. 1019
Pseudomonas sp. D5044 DQ480134.1: 1.. 1475
Proteobacteria Gammaproteobacteria Pseudomonadales Pseudomonadaceae Pseudomonas Pseudomonas xinjiangensis Otu3644
Proteobacteria Gammaproteobacteria Pseudomonadales Marinomonadaceae Marinomonas Marinomonas primoryensis Otu2095
Marinomonas sp. BSw10005 AY646429.1: 1.. 1497
DMSP-degrading marine bacterium JA22 AF296136.1:complement(1.. 509)
Woeseia oceani (T) XK5 KM034745.1: 1..1476
Proteobacteria Gammaproteobacteria GammaproteobacteriaIncertaeSedis UnknownFamily CandidatusBerkiella CandidatusBerkiella sp Otu3564
Proteobacteria Gammaproteobacteria 1 Gammaproteobacteria 1 Gammaproteobacteria 1 Gammaproteobacteria Gammaproteobacteria 1 Otu0648
Legionella-like amoebal pathogen CC99 EF492067.1: 1.. 1501
Legionella-like amoebal pathogen HT99 AY741401.1: 1.. 1512
Rickettsiella symbiont of Asellus aquaticus 10 AY447041.1: 1.. 1479
Proteobacteria Gammaproteobacteria Diplorickettsiales Diplorickettsiaceae Rickettsiella Rickettsiella tipulae Otu2776
Rickettsiella tipulae BBA296/BUR-D/1961 EU180598.1: 1.. 1462
Proteobacteria Gammaproteobacteria GammaproteobacteriaIncertaeSedis UnknownFamily CandidatusOvatusbacter CandidatusOvatusbacter sp Otu2879
Proteobacteria Gammaproteobacteria Legionellales Legionellaceae Legionella Legionella lansigensis Otu1602
Legionella beliardensis Montbeliard A1 AF122884.2: 1.. 1470
Legionella lansingensis type strain: DSM 19556 HF937256.1: 1.. 1533
proteobacterium Ellin139 AF408981.1: 1.. 1439

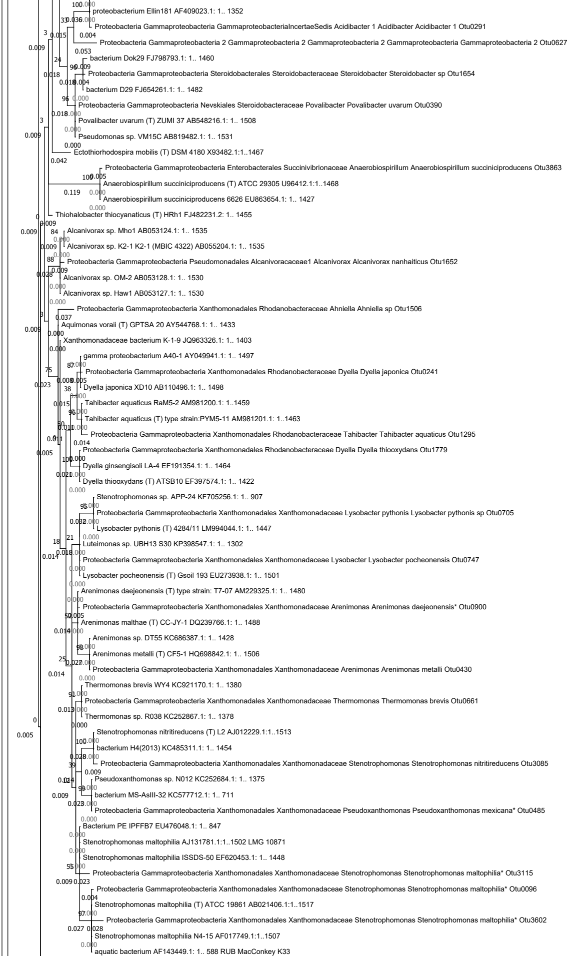
proteobacterium Ellin181 AF409023.1: 1.. 1352
Proteobacteria Gammaproteobacteria GammaproteobacteriaIncertaeSedis Acidibacter 1 Acidibacter Acidibacter 1 Otu0291
Proteobacteria Gammaproteobacteria 2 Gammaproteobacteria 2 Gammaproteobacteria 2 Gammaproteobacteria Gammaproteobacteria 2 Otu0627
bacterium Dok29 FJ798793.1: 1.. 1460
Proteobacteria Gammaproteobacteria Steroidobacterales Steroidobacteraceae Steroidobacter Steroidobacter sp Otu1654
bacterium D29 FJ654261.1: 1.. 1482
Proteobacteria Gammaproteobacteria Nevskiales Steroidobacteraceae Povalibacter Povalibacter uvarum Otu0390
Povalibacter uvarum (T) ZUMI 37 AB548216.1: 1.. 1508
Pseudomonas sp. VM15C AB819482.1: 1.. 1531
Ectothiorhodospira mobilis (T) DSM 4180 X93482.1:1..1467
Proteobacteria Gammaproteobacteria Enterobacterales Succinivibrionaceae Anaerobiospirillum Anaerobiospirillum succiniciproducens Otu3863
Anaerobiospirillum succiniciproducens (T) ATCC 29305 U96412.1:1..1468
Anaerobiospirillum succiniciproducens 6626 EU863654.1: 1.. 1427
Thiohalobacter thiocyanaticus (T) HRh1 FJ482231.2: 1.. 1455
Alcanivorax sp. Mho1 AB053124.1: 1.. 1535
Alcanivorax sp. K2-1 K2-1 (MBIC 4322) AB055204.1: 1.. 1535
Proteobacteria Gammaproteobacteria Pseudomonadales Alcanivoracaceae1 Alcanivorax Alcanivorax nanhaiticus Otu1652
Alcanivorax sp. OM-2 AB053128.1: 1.. 1530
Alcanivorax sp. Haw1 AB053127.1: 1.. 1530
Proteobacteria Gammaproteobacteria Xanthomonadales Rhodanobacteraceae Ahniella Ahniella sp Otu1506
Aquimonas voraii (T) GPTSA 20 AY544768.1: 1.. 1433
Xanthomonadaceae bacterium K-1-9 JQ963326.1: 1.. 1403
gamma proteobacterium A40-1 AY049941.1: 1.. 1497
Proteobacteria Gammaproteobacteria Xanthomonadales Rhodanobacteraceae Dyella Dyella japonica Otu0241
Dyella japonica XD10 AB110496.1: 1.. 1498
Tahibacter aquaticus RaM5-2 AM981200.1: 1..1459
Tahibacter aquaticus (T) type strain:PYM5-11 AM981201.1: 1..1463
Proteobacteria Gammaproteobacteria Xanthomonadales Rhodanobacteraceae Tahibacter Tahibacter aquaticus Otu1295
Proteobacteria Gammaproteobacteria Xanthomonadales Rhodanobacteraceae Dyella Dyella thiooxydans Otu1779
Dyella ginsengisoli LA-4 EF191354.1: 1.. 1464
Dyella thiooxydans (T) ATSB10 EF397574.1: 1.. 1422
Stenotrophomonas sp. APP-24 KF705256.1: 1.. 907
Proteobacteria Gammaproteobacteria Xanthomonadales Xanthomonadaceae Lysobacter pythonis Lysobacter pythonis sp Otu0705
Lysobacter pythonis (T) 4284/11 LM994044.1: 1.. 1447
Luteimonas sp. UBH13 S30 KP398547.1: 1.. 1302
Proteobacteria Gammaproteobacteria Xanthomonadales Xanthomonadaceae Lysobacter Lysobacter pocheonensis Otu0747
Lysobacter pocheonensis (T) Gsoil 193 EU273938.1: 1.. 1501
Arenimonas daejeonensis (T) type strain: T7-07 AM229325.1: 1.. 1480
Proteobacteria Gammaproteobacteria Xanthomonadales Xanthomonadaceae Arenimonas Arenimonas daejeonensis* Otu0900
Arenimonas malthae (T) CC-JY-1 DQ239766.1: 1.. 1488
Arenimonas sp. DT55 KC686387.1: 1.. 1428
Arenimonas metalli (T) CF5-1 HQ698842.1: 1.. 1506
Proteobacteria Gammaproteobacteria Xanthomonadales Xanthomonadaceae Arenimonas Arenimonas metalli Otu0430
Thermomonas brevis WY4 KC921170.1: 1.. 1380
Proteobacteria Gammaproteobacteria Xanthomonadales Xanthomonadaceae Thermomonas Thermomonas brevis Otu0661
Thermomonas sp. R038 KC252867.1: 1.. 1378
Stenotrophomonas nitritireducens (T) L2 AJ012229.1:1..1513
bacterium H4(2013) KC485311.1: 1.. 1454
Proteobacteria Gammaproteobacteria Xanthomonadales Xanthomonadaceae Stenotrophomonas Stenotrophomonas nitritireducens Otu3085
Pseudoxanthomonas sp. N012 KC252684.1: 1.. 1375
bacterium MS-AsIII-32 KC577712.1: 1.. 711
Proteobacteria Gammaproteobacteria Xanthomonadales Xanthomonadaceae Pseudoxanthomonas Pseudoxanthomonas mexicana* Otu0485
Bacterium PE IPFFB7 EU476048.1: 1.. 847
Stenotrophomonas maltophilia AJ131781.1:1..1502 LMG 10871
Stenotrophomonas maltophilia ISSDS-50 EF620453.1: 1.. 1448
Proteobacteria Gammaproteobacteria Xanthomonadales Xanthomonadaceae Stenotrophomonas Stenotrophomonas maltophilia* Otu3115
Proteobacteria Gammaproteobacteria Xanthomonadales Xanthomonadaceae Stenotrophomonas Stenotrophomonas maltophilia* Otu0096
Stenotrophomonas maltophilia (T) ATCC 19861 AB021406.1:1..1517
Proteobacteria Gammaproteobacteria Xanthomonadales Xanthomonadaceae Stenotrophomonas Stenotrophomonas maltophilia* Otu3602
Stenotrophomonas maltophilia N4-15 AF017749.1:1..1507
aquatic bacterium AF143449.1: 1.. 588 RUB MacConkey K33

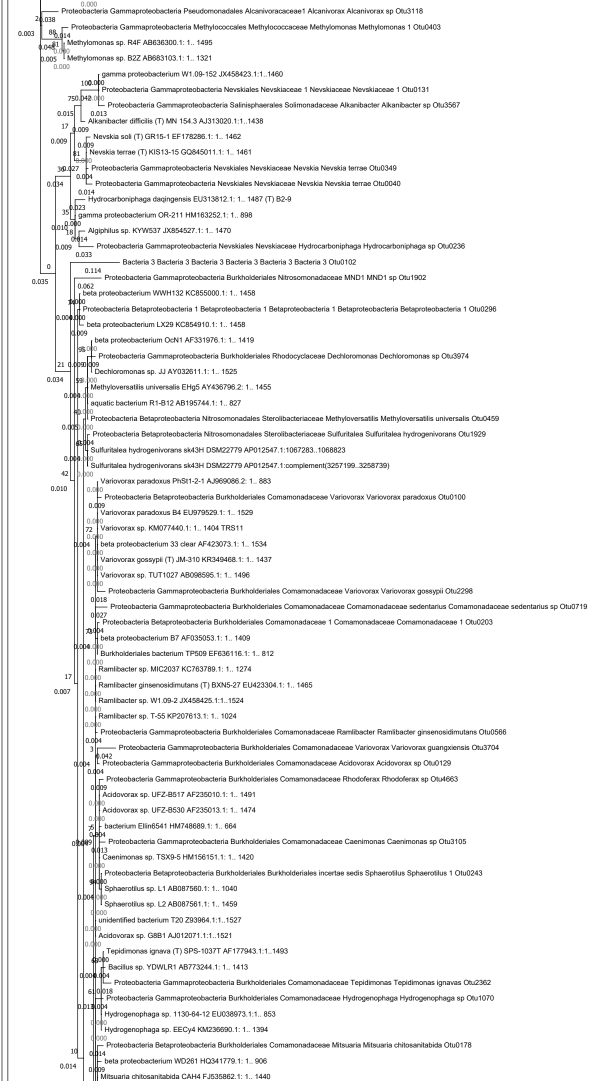
Proteobacteria Gammaproteobacteria Pseudomonadales Alcanivoracaceae1 Alcanivorax Alcanivorax sp Otu3118
Proteobacteria Gammaproteobacteria Methylococcales Methylococcaceae Methylomonas Methylomonas 1 Otu0403
Methylomonas sp. R4F AB636300.1: 1.. 1495
Methylomonas sp. B2Z AB683103.1: 1.. 1321
gamma proteobacterium W1.09-152 JX458423.1:1..1460
Proteobacteria Gammaproteobacteria Nevskiales Nevskiaceae 1 Nevskiaceae Nevskiaceae 1 Otu0131
Proteobacteria Gammaproteobacteria Salinisphaerales Solimonadaceae Alkanibacter Alkanibacter sp Otu3567
Alkanibacter difficilis (T) MN 154.3 AJ313020.1:1..1438
Nevskia soli (T) GR15-1 EF178286.1: 1.. 1462
Nevskia terrae (T) KIS13-15 GQ845011.1: 1.. 1461
Proteobacteria Gammaproteobacteria Nevskiales Nevskiaceae Nevskia Nevskia terrae Otu0349
Proteobacteria Gammaproteobacteria Nevskiales Nevskiaceae Nevskia Nevskia terrae Otu0040
Hydrocarboniphaga daqingensis EU313812.1: 1.. 1487 (T) B2-9
gamma proteobacterium OR-211 HM163252.1: 1.. 898
Algiphilus sp. KYW537 JX854527.1: 1.. 1470
Proteobacteria Gammaproteobacteria Nevskiales Nevskiaceae Hydrocarboniphaga Hydrocarboniphaga sp Otu0236
Bacteria 3 Bacteria 3 Bacteria 3 Bacteria 3 Bacteria 3 Bacteria 3 Otu0102
Proteobacteria Gammaproteobacteria Burkholderiales Nitrosomonadaceae MND1 MND1 sp Otu1902
beta proteobacterium WWH132 KC855000.1: 1.. 1458
Proteobacteria Betaproteobacteria 1 Betaproteobacteria 1 Betaproteobacteria 1 Betaproteobacteria Betaproteobacteria 1 Otu0296
beta proteobacterium LX29 KC854910.1: 1.. 1458
beta proteobacterium OcN1 AF331976.1: 1.. 1419
Proteobacteria Gammaproteobacteria Burkholderiales Rhodocyclaceae Dechloromonas Dechloromonas sp Otu3974
Dechloromonas sp. JJ AY032611.1: 1.. 1525
Methyloversatilis universalis EHg5 AY436796.2: 1.. 1455
aquatic bacterium R1-B12 AB195744.1: 1.. 827
Proteobacteria Betaproteobacteria Nitrosomonadales Sterolibacteriaceae Methyloversatilis Methyloversatilis universalis Otu0459
Proteobacteria Betaproteobacteria Nitrosomonadales Sterolibacteriaceae Sulfuritalea Sulfuritalea hydrogenivorans Otu1929
Sulfuritalea hydrogenivorans sk43H DSM22779 AP012547.1:1067283..1068823
Sulfuritalea hydrogenivorans sk43H DSM22779 AP012547.1:complement(3257199..3258739)
Variovorax paradoxus PhSt1-2-1 AJ969086.2: 1.. 883
Proteobacteria Betaproteobacteria Burkholderiales Comamonadaceae Variovorax Variovorax paradoxus Otu0100
Variovorax paradoxus B4 EU979529.1: 1.. 1529
Variovorax sp. KM077440.1: 1.. 1404 TRS11
beta proteobacterium 33 clear AF423073.1: 1.. 1534
Variovorax gossypii (T) JM-310 KR349468.1: 1.. 1437
Variovorax sp. TUT1027 AB098595.1: 1.. 1496
Proteobacteria Gammaproteobacteria Burkholderiales Comamonadaceae Variovorax Variovorax gossypii Otu2298
Proteobacteria Gammaproteobacteria Burkholderiales Comamonadaceae Comamonadaceae sedentarius Comamonadaceae sedentarius sp Otu0719
Proteobacteria Betaproteobacteria Burkholderiales Comamonadaceae 1 Comamonadaceae Comamonadaceae 1 Otu0203
beta proteobacterium B7 AF035053.1: 1.. 1409
Burkholderiales bacterium TP509 EF636116.1: 1.. 812
Ramlibacter sp. MIC2037 KC763789.1: 1.. 1274
Ramlibacter ginsenosidimutans (T) BXN5-27 EU423304.1: 1.. 1465
Ramlibacter sp. W1.09-2 JX458425.1:1..1524
Ramlibacter sp. T-55 KP207613.1: 1.. 1024
Proteobacteria Gammaproteobacteria Burkholderiales Comamonadaceae Ramlibacter Ramlibacter ginsenosidimutans Otu0566
Proteobacteria Gammaproteobacteria Burkholderiales Comamonadaceae Variovorax Variovorax guangxiensis Otu3704
Proteobacteria Gammaproteobacteria Burkholderiales Comamonadaceae Acidovorax Acidovorax sp Otu0129
Proteobacteria Gammaproteobacteria Burkholderiales Comamonadaceae Rhodoferax Rhodoferax sp Otu4663
Acidovorax sp. UFZ-B517 AF235010.1: 1.. 1491
Acidovorax sp. UFZ-B530 AF235013.1: 1.. 1474
bacterium Ellin6541 HM748689.1: 1.. 664
Proteobacteria Gammaproteobacteria Burkholderiales Comamonadaceae Caenimonas Caenimonas sp Otu3105
Caenimonas sp. TSX9-5 HM156151.1: 1.. 1420
Proteobacteria Betaproteobacteria Burkholderiales Burkholderiales incertae sedis Sphaerotilus Sphaerotilus 1 Otu0243
Sphaerotilus sp. L1 AB087560.1: 1.. 1040
Sphaerotilus sp. L2 AB087561.1: 1.. 1459
unidentified bacterium T20 Z93964.1:1..1527
Acidovorax sp. G8B1 AJ012071.1:1..1521
Tepidimonas ignava (T) SPS-1037T AF177943.1:1..1493
Bacillus sp. YDWLR1 AB773244.1: 1.. 1413
Proteobacteria Gammaproteobacteria Burkholderiales Comamonadaceae Tepidimonas Tepidimonas ignavas Otu2362
Proteobacteria Gammaproteobacteria Burkholderiales Comamonadaceae Hydrogenophaga Hydrogenophaga sp Otu1070
Hydrogenophaga sp. 1130-64-12 EU038973.1:1.. 853
Hydrogenophaga sp. EECy4 KM236690.1: 1.. 1394
Proteobacteria Betaproteobacteria Burkholderiales Comamonadaceae Mitsuaria Mitsuaria chitosanitabida Otu0178
beta proteobacterium WD261 HQ341779.1: 1.. 906
Mitsuaria chitosanitabida CAH4 FJ535862.1: 1.. 1440

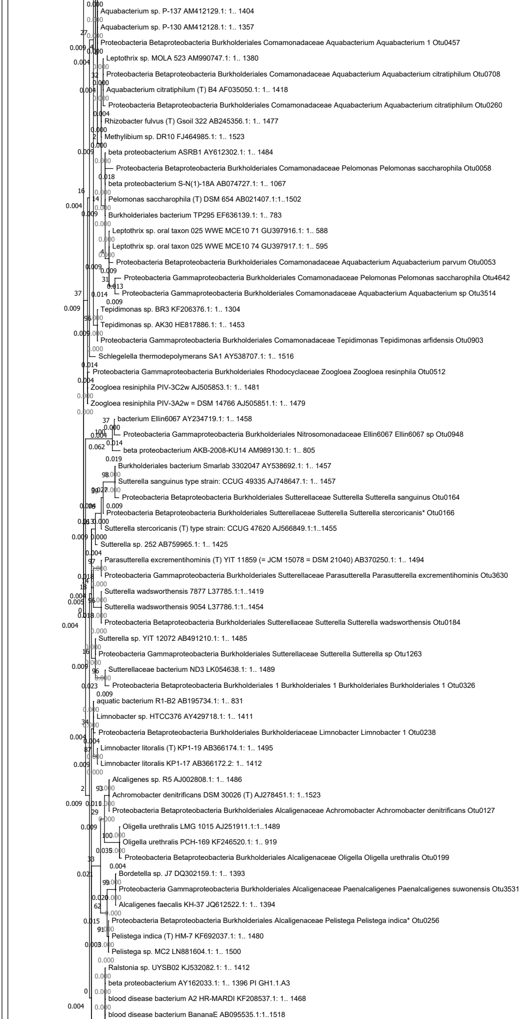
Aquabacterium sp. P-137 AM412129.1: 1.. 1404
Aquabacterium sp. P-130 AM412128.1: 1.. 1357
Proteobacteria Betaproteobacteria Burkholderiales Comamonadaceae Aquabacterium Aquabacterium 1 Otu0457
Leptothrix sp. MOLA 523 AM990747.1: 1.. 1380
Proteobacteria Betaproteobacteria Burkholderiales Comamonadaceae Aquabacterium Aquabacterium citratiphilum Otu0708
Aquabacterium citratiphilum (T) B4 AF035050.1: 1.. 1418
Proteobacteria Betaproteobacteria Burkholderiales Comamonadaceae Aquabacterium Aquabacterium citratiphilum Otu0260
Rhizobacter fulvus (T) Gsoil 322 AB245356.1: 1.. 1477
Methylibium sp. DR10 FJ464985.1: 1.. 1523
beta proteobacterium ASRB1 AY612302.1: 1.. 1484
Proteobacteria Betaproteobacteria Burkholderiales Comamonadaceae Pelomonas Pelomonas saccharophila Otu0058
beta proteobacterium S-N(1)-18A AB074727.1: 1.. 1067
Pelomonas saccharophila (T) DSM 654 AB021407.1:1..1502
Burkholderiales bacterium TP295 EF636139.1: 1.. 783
Leptothrix sp. oral taxon 025 WWE MCE10 71 GU397916.1: 1.. 588
Leptothrix sp. oral taxon 025 WWE MCE10 74 GU397917.1: 1.. 595
Proteobacteria Betaproteobacteria Burkholderiales Comamonadaceae Aquabacterium Aquabacterium parvum Otu0053
Proteobacteria Gammaproteobacteria Burkholderiales Comamonadaceae Pelomonas Pelomonas saccharophila Otu4642
Proteobacteria Gammaproteobacteria Burkholderiales Comamonadaceae Aquabacterium Aquabacterium sp Otu3514
Tepidimonas sp. BR3 KF206376.1: 1.. 1304
Tepidimonas sp. AK30 HE817886.1: 1.. 1453
Proteobacteria Gammaproteobacteria Burkholderiales Comamonadaceae Tepidimonas Tepidimonas arfidensis Otu0903
Schlegelella thermodepolymerans SA1 AY538707.1: 1.. 1516
Proteobacteria Gammaproteobacteria Burkholderiales Rhodocyclaceae Zoogloea Zoogloea resinphila Otu0512
Zoogloea resiniphila PIV-3C2w AJ505853.1: 1.. 1481
Zoogloea resiniphila PIV-3A2w = DSM 14766 AJ505851.1: 1.. 1479
bacterium Ellin6067 AY234719.1: 1.. 1458
Proteobacteria Gammaproteobacteria Burkholderiales Nitrosomonadaceae Ellin6067 Ellin6067 sp Otu0948
beta proteobacterium AKB-2008-KU14 AM989130.1: 1.. 805
Burkholderiales bacterium Smarlab 3302047 AY538692.1: 1.. 1457
Sutterella sanguinus type strain: CCUG 49335 AJ748647.1: 1.. 1457
Proteobacteria Betaproteobacteria Burkholderiales Sutterellaceae Sutterella Sutterella sanguinus Otu0164
Proteobacteria Betaproteobacteria Burkholderiales Sutterellaceae Sutterella Sutterella stercoricanis* Otu0166
Sutterella stercoricanis (T) type strain: CCUG 47620 AJ566849.1:1..1455
Sutterella sp. 252 AB759965.1: 1.. 1425
Parasutterella excrementihominis (T) YIT 11859 (= JCM 15078 = DSM 21040) AB370250.1: 1.. 1494
Proteobacteria Gammaproteobacteria Burkholderiales Sutterellaceae Parasutterella Parasutterella excrementihominis Otu3630
Sutterella wadsworthensis 7877 L37785.1:1..1419
Sutterella wadsworthensis 9054 L37786.1:1..1454
Proteobacteria Betaproteobacteria Burkholderiales Sutterellaceae Sutterella Sutterella wadsworthensis Otu0184
Sutterella sp. YIT 12072 AB491210.1: 1.. 1485
Proteobacteria Gammaproteobacteria Burkholderiales Sutterellaceae Sutterella Sutterella sp Otu1263
Sutterellaceae bacterium ND3 LK054638.1: 1.. 1489
Proteobacteria Betaproteobacteria Burkholderiales 1 Burkholderiales 1 Burkholderiales Burkholderiales 1 Otu0326
aquatic bacterium R1-B2 AB195734.1: 1.. 831
Limnobacter sp. HTCC376 AY429718.1: 1.. 1411
Proteobacteria Betaproteobacteria Burkholderiales Burkholderiaceae Limnobacter Limnobacter 1 Otu0238
Limnobacter litoralis (T) KP1-19 AB366174.1: 1.. 1495
Limnobacter litoralis KP1-17 AB366172.2: 1.. 1412
Alcaligenes sp. R5 AJ002808.1: 1.. 1486
Achromobacter denitrificans DSM 30026 (T) AJ278451.1: 1..1523
Proteobacteria Betaproteobacteria Burkholderiales Alcaligenaceae Achromobacter Achromobacter denitrificans Otu0127
Oligella urethralis LMG 1015 AJ251911.1:1..1489
Oligella urethralis PCH-169 KF246520.1: 1.. 919
Proteobacteria Betaproteobacteria Burkholderiales Alcaligenaceae Oligella Oligella urethralis Otu0199
Bordetella sp. J7 DQ302159.1: 1.. 1393
Proteobacteria Gammaproteobacteria Burkholderiales Alcaligenaceae Paenalcaligenes Paenalcaligenes suwonensis Otu3531
Alcaligenes faecalis KH-37 JQ612522.1: 1.. 1394
Proteobacteria Betaproteobacteria Burkholderiales Alcaligenaceae Pelistega Pelistega indica* Otu0256
Pelistega indica (T) HM-7 KF692037.1: 1.. 1480
Pelistega sp. MC2 LN881604.1: 1.. 1500
Ralstonia sp. UYSB02 KJ532082.1: 1.. 1412
beta proteobacterium AY162033.1: 1.. 1396 PI GH1.1.A3
blood disease bacterium A2 HR-MARDI KF208537.1: 1.. 1468
blood disease bacterium BananaE AB095535.1:1..1518

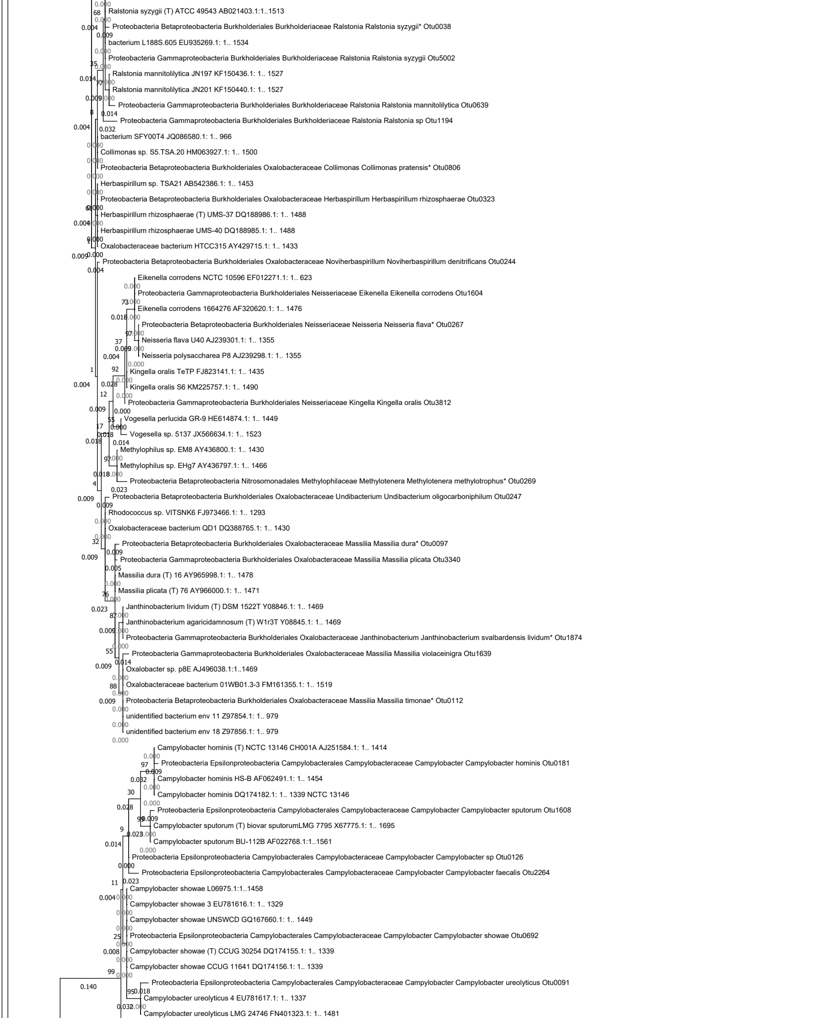
Ralstonia syzygii (T) ATCC 49543 AB021403.1:1..1513
Proteobacteria Betaproteobacteria Burkholderiales Burkholderiaceae Ralstonia Ralstonia syzygii* Otu0038
bacterium L188S.605 EU935269.1: 1.. 1534
Proteobacteria Gammaproteobacteria Burkholderiales Burkholderiaceae Ralstonia Ralstonia syzygii Otu5002
Ralstonia mannitolilytica JN197 KF150436.1: 1.. 1527
Ralstonia mannitolilytica JN201 KF150440.1: 1.. 1527
Proteobacteria Gammaproteobacteria Burkholderiales Burkholderiaceae Ralstonia Ralstonia mannitolilytica Otu0639
Proteobacteria Gammaproteobacteria Burkholderiales Burkholderiaceae Ralstonia Ralstonia sp Otu1194
bacterium SFY00T4 JQ086580.1: 1.. 966
Collimonas sp. S5.TSA.20 HM063927.1: 1.. 1500
Proteobacteria Betaproteobacteria Burkholderiales Oxalobacteraceae Collimonas Collimonas pratensis* Otu0806
Herbaspirillum sp. TSA21 AB542386.1: 1.. 1453
Proteobacteria Betaproteobacteria Burkholderiales Oxalobacteraceae Herbaspirillum Herbaspirillum rhizosphaerae Otu0323
Herbaspirillum rhizosphaerae (T) UMS-37 DQ188986.1: 1.. 1488
Herbaspirillum rhizosphaerae UMS-40 DQ188985.1: 1.. 1488
Oxalobacteraceae bacterium HTCC315 AY429715.1: 1.. 1433
Proteobacteria Betaproteobacteria Burkholderiales Oxalobacteraceae Noviherbaspirillum Noviherbaspirillum denitrificans Otu0244
Eikenella corrodens NCTC 10596 EF012271.1: 1.. 623
Proteobacteria Gammaproteobacteria Burkholderiales Neisseriaceae Eikenella Eikenella corrodens Otu1604
Eikenella corrodens 1664276 AF320620.1: 1.. 1476
Proteobacteria Betaproteobacteria Burkholderiales Neisseriaceae Neisseria Neisseria flava* Otu0267
Neisseria flava U40 AJ239301.1: 1.. 1355
Neisseria polysaccharea P8 AJ239298.1: 1.. 1355
Kingella oralis TeTP FJ823141.1: 1.. 1435
Kingella oralis S6 KM225757.1: 1.. 1490
Proteobacteria Gammaproteobacteria Burkholderiales Neisseriaceae Kingella Kingella oralis Otu3812
Vogesella perlucida GR-9 HE614874.1: 1.. 1449
Vogesella sp. 5137 JX566634.1: 1.. 1523
Methylophilus sp. EM8 AY436800.1: 1.. 1430
Methylophilus sp. EHg7 AY436797.1: 1.. 1466
Proteobacteria Betaproteobacteria Nitrosomonadales Methylophilaceae Methylotenera Methylotenera methylotrophus* Otu0269
Proteobacteria Betaproteobacteria Burkholderiales Oxalobacteraceae Undibacterium Undibacterium oligocarboniphilum Otu0247
Rhodococcus sp. VITSNK6 FJ973466.1: 1.. 1293
Oxalobacteraceae bacterium QD1 DQ388765.1: 1.. 1430
Proteobacteria Betaproteobacteria Burkholderiales Oxalobacteraceae Massilia Massilia dura* Otu0097
Proteobacteria Gammaproteobacteria Burkholderiales Oxalobacteraceae Massilia Massilia plicata Otu3340
Massilia dura (T) 16 AY965998.1: 1.. 1478
Massilia plicata (T) 76 AY966000.1: 1.. 1471
Janthinobacterium lividum (T) DSM 1522T Y08846.1: 1.. 1469
Janthinobacterium agaricidamnosum (T) W1r3T Y08845.1: 1.. 1469
Proteobacteria Gammaproteobacteria Burkholderiales Oxalobacteraceae Janthinobacterium Janthinobacterium svalbardensis lividum* Otu1874
Proteobacteria Gammaproteobacteria Burkholderiales Oxalobacteraceae Massilia Massilia violaceinigra Otu1639
Oxalobacter sp. p8E AJ496038.1:1..1469
Oxalobacteraceae bacterium 01WB01.3-3 FM161355.1: 1.. 1519
Proteobacteria Betaproteobacteria Burkholderiales Oxalobacteraceae Massilia Massilia timonae* Otu0112
unidentified bacterium env 11 Z97854.1: 1.. 979
unidentified bacterium env 18 Z97856.1: 1.. 979
Campylobacter hominis (T) NCTC 13146 CH001A AJ251584.1: 1.. 1414
Proteobacteria Epsilonproteobacteria Campylobacterales Campylobacteraceae Campylobacter Campylobacter hominis Otu0181
Campylobacter hominis HS-B AF062491.1: 1.. 1454
Campylobacter hominis DQ174182.1: 1.. 1339 NCTC 13146
Proteobacteria Epsilonproteobacteria Campylobacterales Campylobacteraceae Campylobacter Campylobacter sputorum Otu1608
Campylobacter sputorum (T) biovar sputorumLMG 7795 X67775.1: 1.. 1695
Campylobacter sputorum BU-112B AF022768.1:1..1561
Proteobacteria Epsilonproteobacteria Campylobacterales Campylobacteraceae Campylobacter Campylobacter sp Otu0126
Proteobacteria Epsilonproteobacteria Campylobacterales Campylobacteraceae Campylobacter Campylobacter faecalis Otu2264
Campylobacter showae L06975.1:1..1458
Campylobacter showae 3 EU781616.1: 1.. 1329
Campylobacter showae UNSWCD GQ167660.1: 1.. 1449
Proteobacteria Epsilonproteobacteria Campylobacterales Campylobacteraceae Campylobacter Campylobacter showae Otu0692
Campylobacter showae (T) CCUG 30254 DQ174155.1: 1.. 1339
Campylobacter showae CCUG 11641 DQ174156.1: 1.. 1339
Proteobacteria Epsilonproteobacteria Campylobacterales Campylobacteraceae Campylobacter Campylobacter ureolyticus Otu0091
Campylobacter ureolyticus 4 EU781617.1: 1.. 1337
Campylobacter ureolyticus LMG 24746 FN401323.1: 1.. 1481

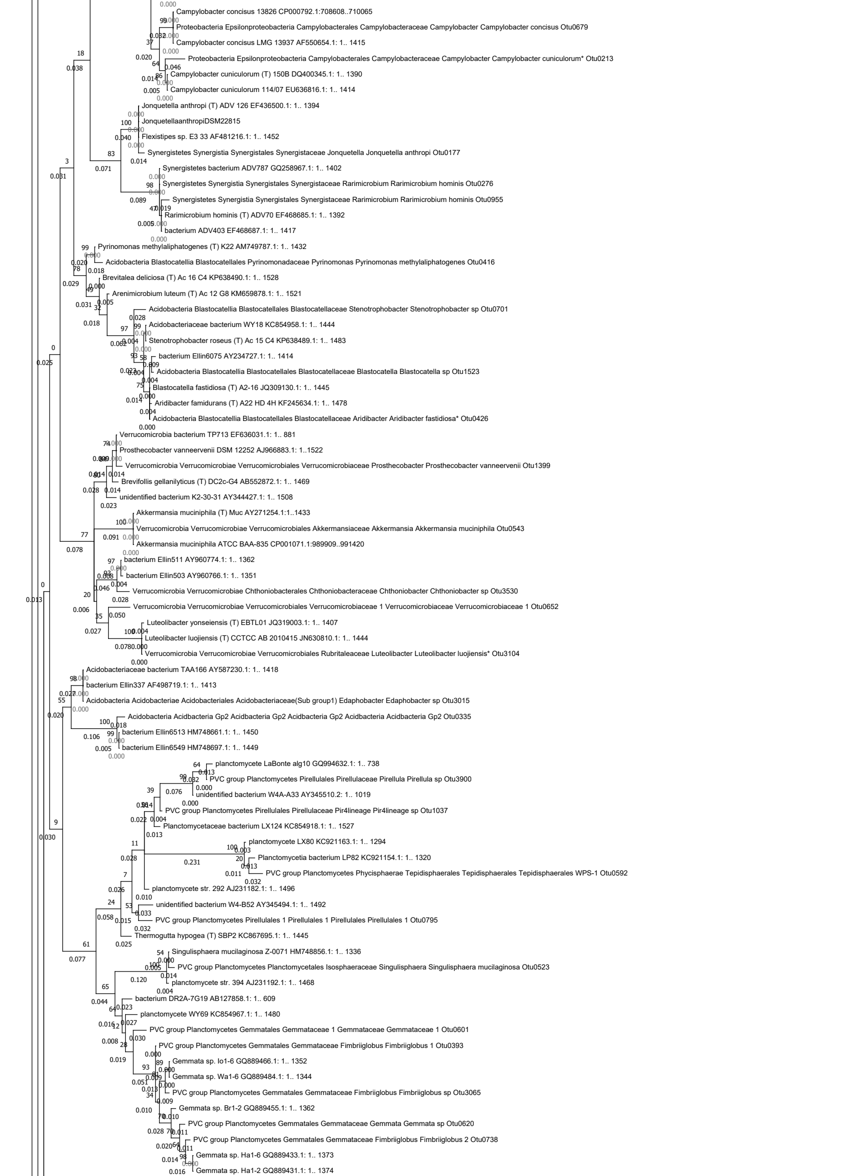
Campylobacter concisus 13826 CP000792.1:708608..710065
Proteobacteria Epsilonproteobacteria Campylobacterales Campylobacteraceae Campylobacter Campylobacter concisus Otu0679
Campylobacter concisus LMG 13937 AF550654.1: 1.. 1415
Proteobacteria Epsilonproteobacteria Campylobacterales Campylobacteraceae Campylobacter Campylobacter cuniculorum* Otu0213
Campylobacter cuniculorum (T) 150B DQ400345.1: 1.. 1390
Campylobacter cuniculorum 114/07 EU636816.1: 1.. 1414
Jonquetella anthropi (T) ADV 126 EF436500.1: 1.. 1394
JonquetellaanthropiDSM22815
Flexistipes sp. E3 33 AF481216.1: 1.. 1452
Synergistetes Synergistia Synergistales Synergistaceae Jonquetella Jonquetella anthropi Otu0177
Synergistetes bacterium ADV787 GQ258967.1: 1.. 1402
Synergistetes Synergistia Synergistales Synergistaceae Rarimicrobium Rarimicrobium hominis Otu0276
Synergistetes Synergistia Synergistales Synergistaceae Rarimicrobium Rarimicrobium hominis Otu0955
Rarimicrobium hominis (T) ADV70 EF468685.1: 1.. 1392
bacterium ADV403 EF468687.1: 1.. 1417
Pyrinomonas methylaliphatogenes (T) K22 AM749787.1: 1.. 1432
Acidobacteria Blastocatellia Blastocatellales Pyrinomonadaceae Pyrinomonas Pyrinomonas methylaliphatogenes Otu0416
Brevitalea deliciosa (T) Ac 16 C4 KP638490.1: 1.. 1528
Arenimicrobium luteum (T) Ac 12 G8 KM659878.1: 1.. 1521
Acidobacteria Blastocatellia Blastocatellales Blastocatellaceae Stenotrophobacter Stenotrophobacter sp Otu0701
Acidobacteriaceae bacterium WY18 KC854958.1: 1.. 1444
Stenotrophobacter roseus (T) Ac 15 C4 KP638489.1: 1.. 1483
bacterium Ellin6075 AY234727.1: 1.. 1414
Acidobacteria Blastocatellia Blastocatellales Blastocatellaceae Blastocatella Blastocatella sp Otu1523
Blastocatella fastidiosa (T) A2-16 JQ309130.1: 1.. 1445
Aridibacter famidurans (T) A22 HD 4H KF245634.1: 1.. 1478
Acidobacteria Blastocatellia Blastocatellales Blastocatellaceae Aridibacter Aridibacter fastidiosa* Otu0426
Verrucomicrobia bacterium TP713 EF636031.1: 1.. 881
Prosthecobacter vanneervenii DSM 12252 AJ966883.1: 1..1522
Verrucomicrobia Verrucomicrobiae Verrucomicrobiales Verrucomicrobiaceae Prosthecobacter Prosthecobacter vanneervenii Otu1399
Brevifollis gellanilyticus (T) DC2c-G4 AB552872.1: 1.. 1469
unidentified bacterium K2-30-31 AY344427.1: 1.. 1508
Akkermansia muciniphila (T) Muc AY271254.1:1..1433
Verrucomicrobia Verrucomicrobiae Verrucomicrobiales Akkermansiaceae Akkermansia Akkermansia muciniphila Otu0543
Akkermansia muciniphila ATCC BAA-835 CP001071.1:989909..991420
bacterium Ellin511 AY960774.1: 1.. 1362
bacterium Ellin503 AY960766.1: 1.. 1351
Verrucomicrobia Verrucomicrobiae Chthoniobacterales Chthoniobacteraceae Chthoniobacter Chthoniobacter sp Otu3530
Verrucomicrobia Verrucomicrobiae Verrucomicrobiales Verrucomicrobiaceae 1 Verrucomicrobiaceae Verrucomicrobiaceae 1 Otu0652
Luteolibacter yonseiensis (T) EBTL01 JQ319003.1: 1.. 1407
Luteolibacter luojiensis (T) CCTCC AB 2010415 JN630810.1: 1.. 1444
Verrucomicrobia Verrucomicrobiae Verrucomicrobiales Rubritaleaceae Luteolibacter Luteolibacter luojiensis* Otu3104
Acidobacteriaceae bacterium TAA166 AY587230.1: 1.. 1418
bacterium Ellin337 AF498719.1: 1.. 1413
Acidobacteria Acidobacteriae Acidobacteriales Acidobacteriaceae(Sub group1) Edaphobacter Edaphobacter sp Otu3015
Acidobacteria Acidbacteria Gp2 Acidbacteria Gp2 Acidbacteria Gp2 Acidbacteria Acidbacteria Gp2 Otu0335
bacterium Ellin6513 HM748661.1: 1.. 1450
bacterium Ellin6549 HM748697.1: 1.. 1449
planctomycete LaBonte alg10 GQ994632.1: 1.. 738
PVC group Planctomycetes Pirellulales Pirellulaceae Pirellula Pirellula sp Otu3900
unidentified bacterium W4A-A33 AY345510.2: 1.. 1019
PVC group Planctomycetes Pirellulales Pirellulaceae Pir4lineage Pir4lineage sp Otu1037
Planctomycetaceae bacterium LX124 KC854918.1: 1.. 1527
planctomycete LX80 KC921163.1: 1.. 1294
Planctomycetia bacterium LP82 KC921154.1: 1.. 1320
PVC group Planctomycetes Phycisphaerae Tepidisphaerales Tepidisphaerales Tepidisphaerales WPS-1 Otu0592
planctomycete str. 292 AJ231182.1: 1.. 1496
unidentified bacterium W4-B52 AY345494.1: 1.. 1492
PVC group Planctomycetes Pirellulales 1 Pirellulales 1 Pirellulales Pirellulales 1 Otu0795
Thermogutta hypogea (T) SBP2 KC867695.1: 1.. 1445
Singulisphaera mucilaginosa Z-0071 HM748856.1: 1.. 1336
PVC group Planctomycetes Planctomycetales Isosphaeraceae Singulisphaera Singulisphaera mucilaginosa Otu0523
planctomycete str. 394 AJ231192.1: 1.. 1468
bacterium DR2A-7G19 AB127858.1: 1.. 609
planctomycete WY69 KC854967.1: 1.. 1480
PVC group Planctomycetes Gemmatales Gemmataceae 1 Gemmataceae Gemmataceae 1 Otu0601
PVC group Planctomycetes Gemmatales Gemmataceae Fimbriiglobus Fimbriiglobus 1 Otu0393
Gemmata sp. Io1-6 GQ889466.1: 1.. 1352
Gemmata sp. Wa1-6 GQ889484.1: 1.. 1344
PVC group Planctomycetes Gemmatales Gemmataceae Fimbriiglobus Fimbriiglobus sp Otu3065
Gemmata sp. Br1-2 GQ889455.1: 1.. 1362
PVC group Planctomycetes Gemmatales Gemmataceae Gemmata Gemmata sp Otu0620
PVC group Planctomycetes Gemmatales Gemmataceae Fimbriiglobus Fimbriiglobus 2 Otu0738
Gemmata sp. Ha1-6 GQ889433.1: 1.. 1373
Gemmata sp. Ha1-2 GQ889431.1: 1.. 1374

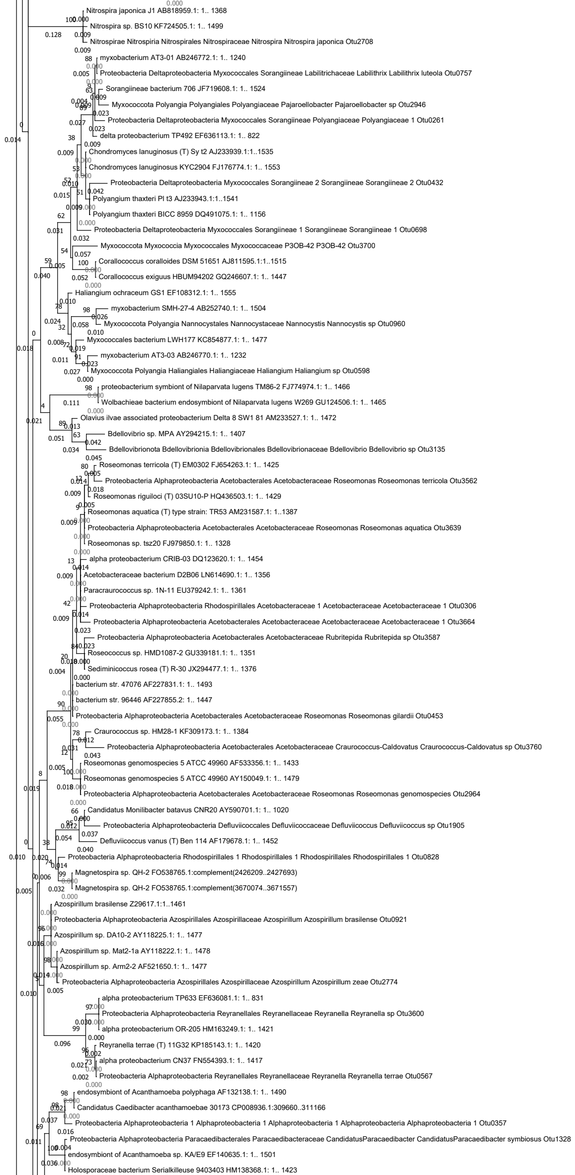
Nitrospira japonica J1 AB818959.1: 1.. 1368
Nitrospira sp. BS10 KF724505.1: 1.. 1499
Nitrospirae Nitrospiria Nitrospirales Nitrospiraceae Nitrospira Nitrospira japonica Otu2708
myxobacterium AT3-01 AB246772.1: 1.. 1240
Proteobacteria Deltaproteobacteria Myxococcales Sorangiineae Labilitrichaceae Labilithrix Labilithrix luteola Otu0757
Sorangiineae bacterium 706 JF719608.1: 1.. 1524
Myxococcota Polyangia Polyangiales Polyangiaceae Pajaroellobacter Pajaroellobacter sp Otu2946
Proteobacteria Deltaproteobacteria Myxococcales Sorangiineae Polyangiaceae Polyangiaceae 1 Otu0261
delta proteobacterium TP492 EF636113.1: 1.. 822
Chondromyces lanuginosus (T) Sy t2 AJ233939.1:1..1535
Chondromyces lanuginosus KYC2904 FJ176774.1: 1.. 1553
Proteobacteria Deltaproteobacteria Myxococcales Sorangiineae 2 Sorangiineae Sorangiineae 2 Otu0432
Polyangium thaxteri Pl t3 AJ233943.1:1..1541
Polyangium thaxteri BICC 8959 DQ491075.1: 1.. 1156
Proteobacteria Deltaproteobacteria Myxococcales Sorangiineae 1 Sorangiineae Sorangiineae 1 Otu0698
Myxococcota Myxococcia Myxococcales Myxococcaceae P3OB-42 P3OB-42 Otu3700
Corallococcus coralloides DSM 51651 AJ811595.1:1..1515
Corallococcus exiguus HBUM94202 GQ246607.1: 1.. 1447
Haliangium ochraceum GS1 EF108312.1: 1.. 1555
myxobacterium SMH-27-4 AB252740.1: 1.. 1504
Myxococcota Polyangia Nannocystales Nannocystaceae Nannocystis Nannocystis sp Otu0960
Myxococcales bacterium LWH177 KC854877.1: 1.. 1477
myxobacterium AT3-03 AB246770.1: 1.. 1232
Myxococcota Polyangia Haliangiales Haliangiaceae Haliangium Haliangium sp Otu0598
proteobacterium symbiont of Nilaparvata lugens TM86-2 FJ774974.1: 1.. 1466
Wolbachieae bacterium endosymbiont of Nilaparvata lugens W269 GU124506.1: 1.. 1465
Olavius ilvae associated proteobacterium Delta 8 SW1 81 AM233527.1: 1.. 1472
Bdellovibrio sp. MPA AY294215.1: 1.. 1407
Bdellovibrionota Bdellovibrionia Bdellovibrionales Bdellovibrionaceae Bdellovibrio Bdellovibrio sp Otu3135
Roseomonas terricola (T) EM0302 FJ654263.1: 1.. 1425
Proteobacteria Alphaproteobacteria Acetobacterales Acetobacteraceae Roseomonas Roseomonas terricola Otu3562
Roseomonas riguiloci (T) 03SU10-P HQ436503.1: 1.. 1429
Roseomonas aquatica (T) type strain: TR53 AM231587.1: 1..1387
Proteobacteria Alphaproteobacteria Acetobacterales Acetobacteraceae Roseomonas Roseomonas aquatica Otu3639
Roseomonas sp. tsz20 FJ979850.1: 1.. 1328
alpha proteobacterium CRIB-03 DQ123620.1: 1.. 1454
Acetobacteraceae bacterium D2B06 LN614690.1: 1.. 1356
Paracraurococcus sp. 1N-11 EU379242.1: 1.. 1361
Proteobacteria Alphaproteobacteria Rhodospirillales Acetobacteraceae 1 Acetobacteraceae Acetobacteraceae 1 Otu0306
Proteobacteria Alphaproteobacteria Acetobacterales Acetobacteraceae Acetobacteraceae Acetobacteraceae 1 Otu3664
Proteobacteria Alphaproteobacteria Acetobacterales Acetobacteraceae Rubritepida Rubritepida sp Otu3587
Roseococcus sp. HMD1087-2 GU339181.1: 1.. 1351
Sediminicoccus rosea (T) R-30 JX294477.1: 1.. 1376
bacterium str. 47076 AF227831.1: 1.. 1493
bacterium str. 96446 AF227855.2: 1.. 1447
Proteobacteria Alphaproteobacteria Acetobacterales Acetobacteraceae Roseomonas Roseomonas gilardii Otu0453
Craurococcus sp. HM28-1 KF309173.1: 1.. 1384
Proteobacteria Alphaproteobacteria Acetobacterales Acetobacteraceae Craurococcus-Caldovatus Craurococcus-Caldovatus sp Otu3760
Roseomonas genomospecies 5 ATCC 49960 AF533356.1: 1.. 1433
Roseomonas genomospecies 5 ATCC 49960 AY150049.1: 1.. 1479
Proteobacteria Alphaproteobacteria Acetobacterales Acetobacteraceae Roseomonas Roseomonas genomospecies Otu2964
Candidatus Monilibacter batavus CNR20 AY590701.1: 1.. 1020
Proteobacteria Alphaproteobacteria Defluviicoccales Defluviicoccaceae Defluviicoccus Defluviicoccus sp Otu1905
Defluviicoccus vanus (T) Ben 114 AF179678.1: 1.. 1452
Proteobacteria Alphaproteobacteria Rhodospirillales 1 Rhodospirillales 1 Rhodospirillales Rhodospirillales 1 Otu0828
Magnetospira sp. QH-2 FO538765.1:complement(2426209..2427693)
Magnetospira sp. QH-2 FO538765.1:complement(3670074..3671557)
Azospirillum brasilense Z29617.1:1..1461
Proteobacteria Alphaproteobacteria Azospirillales Azospirillaceae Azospirillum Azospirillum brasilense Otu0921
Azospirillum sp. DA10-2 AY118225.1: 1.. 1477
Azospirillum sp. Mat2-1a AY118222.1: 1.. 1478
Azospirillum sp. Arm2-2 AF521650.1: 1.. 1477
Proteobacteria Alphaproteobacteria Azospirillales Azospirillaceae Azospirillum Azospirillum zeae Otu2774
alpha proteobacterium TP633 EF636081.1: 1.. 831
Proteobacteria Alphaproteobacteria Reyranellales Reyranellaceae Reyranella Reyranella sp Otu3600
alpha proteobacterium OR-205 HM163249.1: 1.. 1421
Reyranella terrae (T) 11G32 KP185143.1: 1.. 1420
alpha proteobacterium CN37 FN554393.1: 1.. 1417
Proteobacteria Alphaproteobacteria Reyranellales Reyranellaceae Reyranella Reyranella terrae Otu0567
endosymbiont of Acanthamoeba polyphaga AF132138.1: 1.. 1490
Candidatus Caedibacter acanthamoebae 30173 CP008936.1:309660..311166
Proteobacteria Alphaproteobacteria 1 Alphaproteobacteria 1 Alphaproteobacteria 1 Alphaproteobacteria Alphaproteobacteria 1 Otu0357
Proteobacteria Alphaproteobacteria Paracaedibacterales Paracaedibacteraceae CandidatusParacaedibacter CandidatusParacaedibacter symbiosus Otu1328
endosymbiont of Acanthamoeba sp. KA/E9 EF140635.1: 1.. 1501
Holosporaceae bacterium Serialkilleuse 9403403 HM138368.1: 1.. 1423

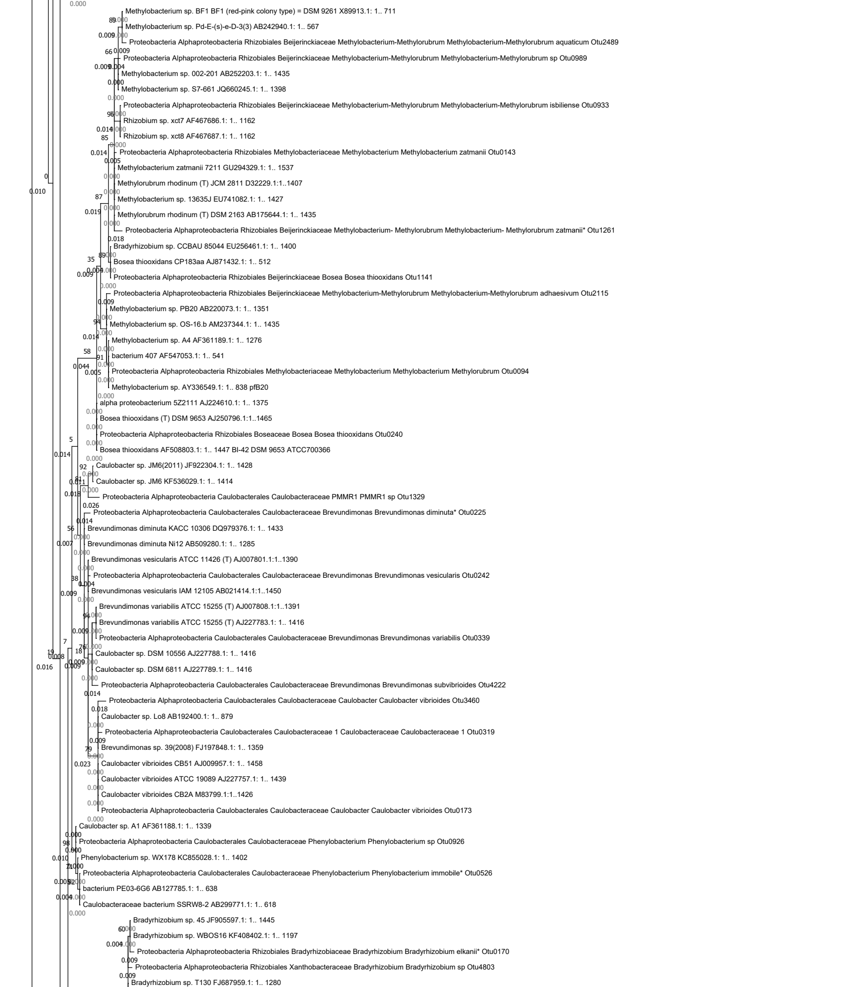

Methylobacterium sp. BF1 BF1 (red-pink colony type) = DSM 9261 X89913.1: 1.. 711
Methylobacterium sp. Pd-E-(s)-e-D-3(3) AB242940.1: 1.. 567
Proteobacteria Alphaproteobacteria Rhizobiales Beijerinckiaceae Methylobacterium-Methylorubrum Methylobacterium-Methylorubrum aquaticum Otu2489
Proteobacteria Alphaproteobacteria Rhizobiales Beijerinckiaceae Methylobacterium-Methylorubrum Methylobacterium-Methylorubrum sp Otu0989
Methylobacterium sp. 002-201 AB252203.1: 1.. 1435
Methylobacterium sp. S7-661 JQ660245.1: 1.. 1398
Proteobacteria Alphaproteobacteria Rhizobiales Beijerinckiaceae Methylobacterium-Methylorubrum Methylobacterium-Methylorubrum isbiliense Otu0933
Rhizobium sp. xct7 AF467686.1: 1.. 1162
Rhizobium sp. xct8 AF467687.1: 1.. 1162
Proteobacteria Alphaproteobacteria Rhizobiales Methylobacteriaceae Methylobacterium Methylobacterium zatmanii Otu0143
Methylobacterium zatmanii 7211 GU294329.1: 1.. 1537
Methylorubrum rhodinum (T) JCM 2811 D32229.1:1..1407
Methylobacterium sp. 13635J EU741082.1: 1.. 1427
Methylorubrum rhodinum (T) DSM 2163 AB175644.1: 1.. 1435
Proteobacteria Alphaproteobacteria Rhizobiales Beijerinckiaceae Methylobacterium- Methylorubrum Methylobacterium- Methylorubrum zatmanii* Otu1261
Bradyrhizobium sp. CCBAU 85044 EU256461.1: 1.. 1400
Bosea thiooxidans CP183aa AJ871432.1: 1.. 512
Proteobacteria Alphaproteobacteria Rhizobiales Beijerinckiaceae Bosea Bosea thiooxidans Otu1141
Proteobacteria Alphaproteobacteria Rhizobiales Beijerinckiaceae Methylobacterium-Methylorubrum Methylobacterium-Methylorubrum adhaesivum Otu2115
Methylobacterium sp. PB20 AB220073.1: 1.. 1351
Methylobacterium sp. OS-16.b AM237344.1: 1.. 1435
Methylobacterium sp. A4 AF361189.1: 1.. 1276
bacterium 407 AF547053.1: 1.. 541
Proteobacteria Alphaproteobacteria Rhizobiales Methylobacteriaceae Methylobacterium Methylobacterium Methylorubrum Otu0094
Methylobacterium sp. AY336549.1: 1.. 838 pfB20
alpha proteobacterium 5Z2111 AJ224610.1: 1.. 1375
Bosea thiooxidans (T) DSM 9653 AJ250796.1:1..1465
Proteobacteria Alphaproteobacteria Rhizobiales Boseaceae Bosea Bosea thiooxidans Otu0240
Bosea thiooxidans AF508803.1: 1.. 1447 BI-42 DSM 9653 ATCC700366
Caulobacter sp. JM6(2011) JF922304.1: 1.. 1428
Caulobacter sp. JM6 KF536029.1: 1.. 1414
Proteobacteria Alphaproteobacteria Caulobacterales Caulobacteraceae PMMR1 PMMR1 sp Otu1329
Proteobacteria Alphaproteobacteria Caulobacterales Caulobacteraceae Brevundimonas Brevundimonas diminuta* Otu0225
Brevundimonas diminuta KACC 10306 DQ979376.1: 1.. 1433
Brevundimonas diminuta Ni12 AB509280.1: 1.. 1285
Brevundimonas vesicularis ATCC 11426 (T) AJ007801.1:1..1390
Proteobacteria Alphaproteobacteria Caulobacterales Caulobacteraceae Brevundimonas Brevundimonas vesicularis Otu0242
Brevundimonas vesicularis IAM 12105 AB021414.1:1..1450
Brevundimonas variabilis ATCC 15255 (T) AJ007808.1:1..1391
Brevundimonas variabilis ATCC 15255 (T) AJ227783.1: 1.. 1416
Proteobacteria Alphaproteobacteria Caulobacterales Caulobacteraceae Brevundimonas Brevundimonas variabilis Otu0339
Caulobacter sp. DSM 10556 AJ227788.1: 1.. 1416
Caulobacter sp. DSM 6811 AJ227789.1: 1.. 1416
Proteobacteria Alphaproteobacteria Caulobacterales Caulobacteraceae Brevundimonas Brevundimonas subvibrioides Otu4222
Proteobacteria Alphaproteobacteria Caulobacterales Caulobacteraceae Caulobacter Caulobacter vibrioides Otu3460
Caulobacter sp. Lo8 AB192400.1: 1.. 879
Proteobacteria Alphaproteobacteria Caulobacterales Caulobacteraceae 1 Caulobacteraceae Caulobacteraceae 1 Otu0319
Brevundimonas sp. 39(2008) FJ197848.1: 1.. 1359
Caulobacter vibrioides CB51 AJ009957.1: 1.. 1458
Caulobacter vibrioides ATCC 19089 AJ227757.1: 1.. 1439
Caulobacter vibrioides CB2A M83799.1:1..1426
Proteobacteria Alphaproteobacteria Caulobacterales Caulobacteraceae Caulobacter Caulobacter vibrioides Otu0173
Caulobacter sp. A1 AF361188.1: 1.. 1339
Proteobacteria Alphaproteobacteria Caulobacterales Caulobacteraceae Phenylobacterium Phenylobacterium sp Otu0926
Phenylobacterium sp. WX178 KC855028.1: 1.. 1402
Proteobacteria Alphaproteobacteria Caulobacterales Caulobacteraceae Phenylobacterium Phenylobacterium immobile* Otu0526
bacterium PE03-6G6 AB127785.1: 1.. 638
Caulobacteraceae bacterium SSRW8-2 AB299771.1: 1.. 618
Bradyrhizobium sp. 45 JF905597.1: 1.. 1445
Bradyrhizobium sp. WBOS16 KF408402.1: 1.. 1197
Proteobacteria Alphaproteobacteria Rhizobiales Bradyrhizobiaceae Bradyrhizobium Bradyrhizobium elkanii* Otu0170
Proteobacteria Alphaproteobacteria Rhizobiales Xanthobacteraceae Bradyrhizobium Bradyrhizobium sp Otu4803
Bradyrhizobium sp. T130 FJ687959.1: 1.. 1280

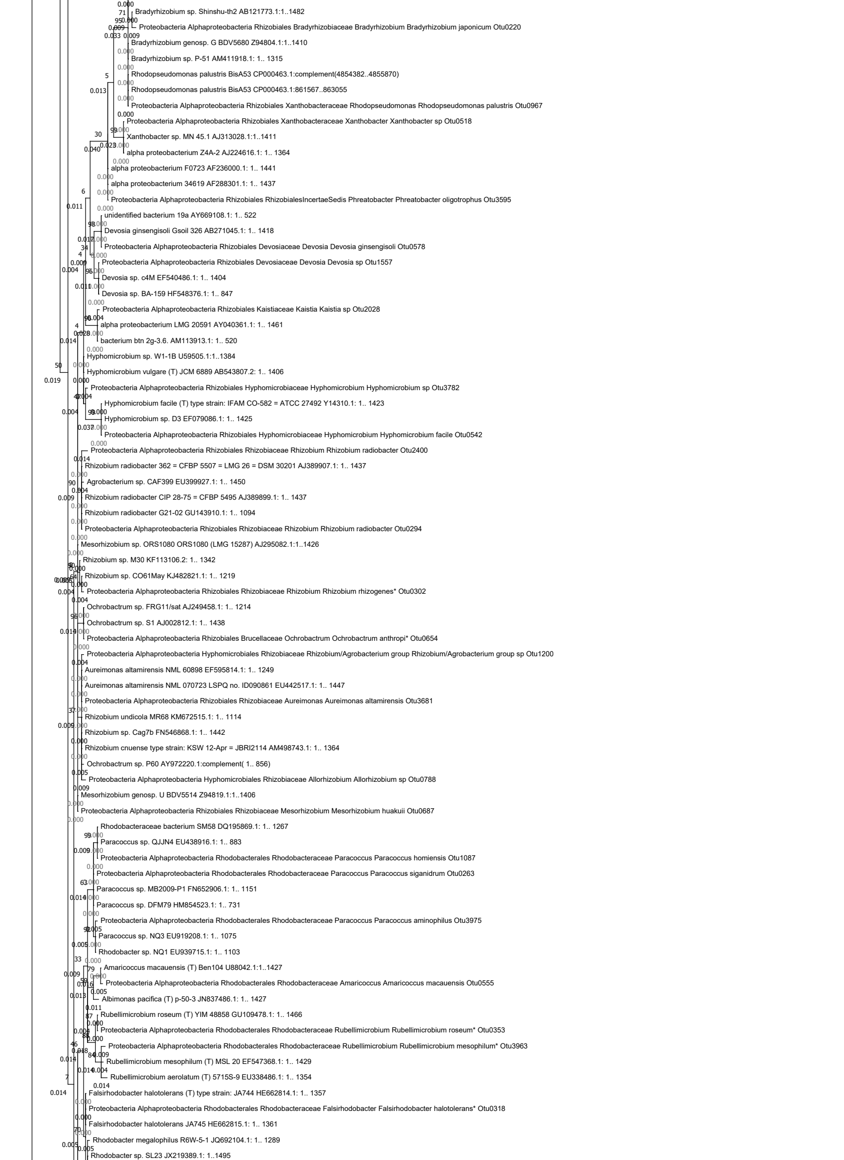
Bradyrhizobium sp. Shinshu-th2 AB121773.1:1..1482
Proteobacteria Alphaproteobacteria Rhizobiales Bradyrhizobiaceae Bradyrhizobium Bradyrhizobium japonicum Otu0220
Bradyrhizobium genosp. G BDV5680 Z94804.1:1..1410
Bradyrhizobium sp. P-51 AM411918.1: 1.. 1315
Rhodopseudomonas palustris BisA53 CP000463.1:complement(4854382..4855870)
Rhodopseudomonas palustris BisA53 CP000463.1:861567..863055
Proteobacteria Alphaproteobacteria Rhizobiales Xanthobacteraceae Rhodopseudomonas Rhodopseudomonas palustris Otu0967
Proteobacteria Alphaproteobacteria Rhizobiales Xanthobacteraceae Xanthobacter Xanthobacter sp Otu0518
Xanthobacter sp. MN 45.1 AJ313028.1:1..1411
alpha proteobacterium Z4A-2 AJ224616.1: 1.. 1364
alpha proteobacterium F0723 AF236000.1: 1.. 1441
alpha proteobacterium 34619 AF288301.1: 1.. 1437
Proteobacteria Alphaproteobacteria Rhizobiales RhizobialesIncertaeSedis Phreatobacter Phreatobacter oligotrophus Otu3595
unidentified bacterium 19a AY669108.1: 1.. 522
Devosia ginsengisoli Gsoil 326 AB271045.1: 1.. 1418
Proteobacteria Alphaproteobacteria Rhizobiales Devosiaceae Devosia Devosia ginsengisoli Otu0578
Proteobacteria Alphaproteobacteria Rhizobiales Devosiaceae Devosia Devosia sp Otu1557
Devosia sp. c4M EF540486.1: 1.. 1404
Devosia sp. BA-159 HF548376.1: 1.. 847
Proteobacteria Alphaproteobacteria Rhizobiales Kaistiaceae Kaistia Kaistia sp Otu2028
alpha proteobacterium LMG 20591 AY040361.1: 1.. 1461
bacterium btn 2g-3.6. AM113913.1: 1.. 520
Hyphomicrobium sp. W1-1B U59505.1:1..1384
Hyphomicrobium vulgare (T) JCM 6889 AB543807.2: 1.. 1406
Proteobacteria Alphaproteobacteria Rhizobiales Hyphomicrobiaceae Hyphomicrobium Hyphomicrobium sp Otu3782
Hyphomicrobium facile (T) type strain: IFAM CO-582 = ATCC 27492 Y14310.1: 1.. 1423
Hyphomicrobium sp. D3 EF079086.1: 1.. 1425
Proteobacteria Alphaproteobacteria Rhizobiales Hyphomicrobiaceae Hyphomicrobium Hyphomicrobium facile Otu0542
Proteobacteria Alphaproteobacteria Rhizobiales Rhizobiaceae Rhizobium Rhizobium radiobacter Otu2400
Rhizobium radiobacter 362 = CFBP 5507 = LMG 26 = DSM 30201 AJ389907.1: 1.. 1437
Agrobacterium sp. CAF399 EU399927.1: 1.. 1450
Rhizobium radiobacter CIP 28-75 = CFBP 5495 AJ389899.1: 1.. 1437
Rhizobium radiobacter G21-02 GU143910.1: 1.. 1094
Proteobacteria Alphaproteobacteria Rhizobiales Rhizobiaceae Rhizobium Rhizobium radiobacter Otu0294
Mesorhizobium sp. ORS1080 ORS1080 (LMG 15287) AJ295082.1:1..1426
Rhizobium sp. M30 KF113106.2: 1.. 1342
Rhizobium sp. CO61May KJ482821.1: 1.. 1219
Proteobacteria Alphaproteobacteria Rhizobiales Rhizobiaceae Rhizobium Rhizobium rhizogenes* Otu0302
Ochrobactrum sp. FRG11/sat AJ249458.1: 1.. 1214
Ochrobactrum sp. S1 AJ002812.1: 1.. 1438
Proteobacteria Alphaproteobacteria Rhizobiales Brucellaceae Ochrobactrum Ochrobactrum anthropi* Otu0654
Proteobacteria Alphaproteobacteria Hyphomicrobiales Rhizobiaceae Rhizobium/Agrobacterium group Rhizobium/Agrobacterium group sp Otu1200
Aureimonas altamirensis NML 60898 EF595814.1: 1.. 1249
Aureimonas altamirensis NML 070723 LSPQ no. ID090861 EU442517.1: 1.. 1447
Proteobacteria Alphaproteobacteria Rhizobiales Rhizobiaceae Aureimonas Aureimonas altamirensis Otu3681
Rhizobium undicola MR68 KM672515.1: 1.. 1114
Rhizobium sp. Cag7b FN546868.1: 1.. 1442
Rhizobium cnuense type strain: KSW 12-Apr = JBRI2114 AM498743.1: 1.. 1364
Ochrobactrum sp. P60 AY972220.1:complement(1.. 856)
Proteobacteria Alphaproteobacteria Hyphomicrobiales Rhizobiaceae Allorhizobium Allorhizobium sp Otu0788
Mesorhizobium genosp. U BDV5514 Z94819.1:1..1406
Proteobacteria Alphaproteobacteria Rhizobiales Rhizobiaceae Mesorhizobium Mesorhizobium huakuii Otu0687
Rhodobacteraceae bacterium SM58 DQ195869.1: 1.. 1267
Paracoccus sp. QJJN4 EU438916.1: 1.. 883
Proteobacteria Alphaproteobacteria Rhodobacterales Rhodobacteraceae Paracoccus Paracoccus homiensis Otu1087
Proteobacteria Alphaproteobacteria Rhodobacterales Rhodobacteraceae Paracoccus Paracoccus siganidrum Otu0263
Paracoccus sp. MB2009-P1 FN652906.1: 1.. 1151
Paracoccus sp. DFM79 HM854523.1: 1.. 731
Proteobacteria Alphaproteobacteria Rhodobacterales Rhodobacteraceae Paracoccus Paracoccus aminophilus Otu3975
Paracoccus sp. NQ3 EU919208.1: 1.. 1075
Rhodobacter sp. NQ1 EU939715.1: 1.. 1103
Amaricoccus macauensis (T) Ben104 U88042.1:1..1427
Proteobacteria Alphaproteobacteria Rhodobacterales Rhodobacteraceae Amaricoccus Amaricoccus macauensis Otu0555
Albimonas pacifica (T) p-50-3 JN837486.1: 1.. 1427
Rubellimicrobium roseum (T) YIM 48858 GU109478.1: 1.. 1466
Proteobacteria Alphaproteobacteria Rhodobacterales Rhodobacteraceae Rubellimicrobium Rubellimicrobium roseum* Otu0353
Proteobacteria Alphaproteobacteria Rhodobacterales Rhodobacteraceae Rubellimicrobium Rubellimicrobium mesophilum* Otu3963
Rubellimicrobium mesophilum (T) MSL 20 EF547368.1: 1.. 1429
Rubellimicrobium aerolatum (T) 5715S-9 EU338486.1: 1.. 1354
Falsirhodobacter halotolerans (T) type strain: JA744 HE662814.1: 1.. 1357
Proteobacteria Alphaproteobacteria Rhodobacterales Rhodobacteraceae Falsirhodobacter Falsirhodobacter halotolerans* Otu0318
Falsirhodobacter halotolerans JA745 HE662815.1: 1.. 1361
Rhodobacter megalophilus R6W-5-1 JQ692104.1: 1.. 1289
Rhodobacter sp. SL23 JX219389.1: 1..1495

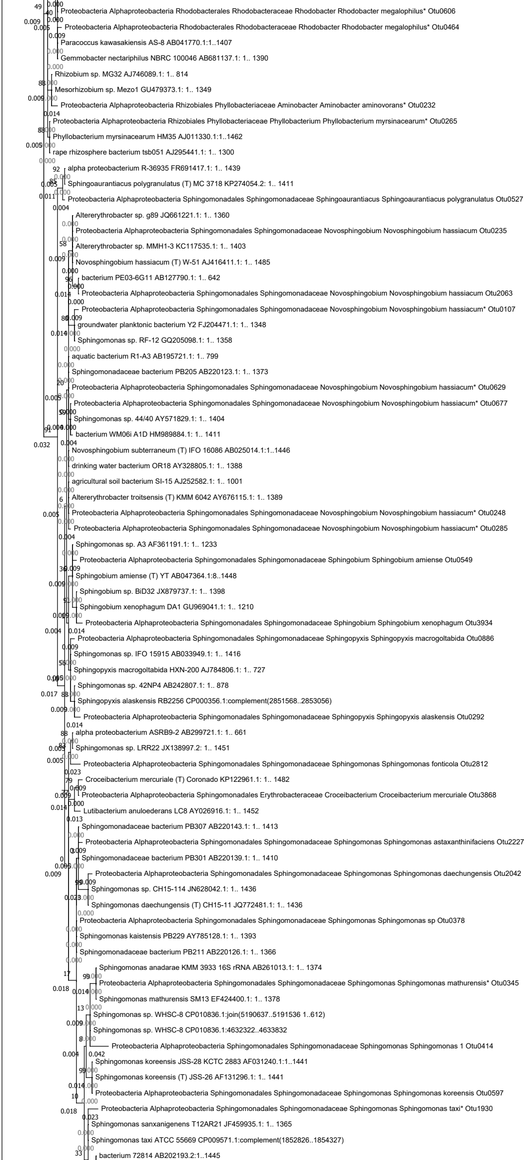
Proteobacteria Alphaproteobacteria Rhodobacterales Rhodobacteraceae Rhodobacter Rhodobacter megalophilus* Otu0606
Proteobacteria Alphaproteobacteria Rhodobacterales Rhodobacteraceae Rhodobacter Rhodobacter megalophilus* Otu0464
Paracoccus kawasakiensis AS-8 AB041770.1:1..1407
Gemmobacter nectariphilus NBRC 100046 AB681137.1: 1.. 1390
Rhizobium sp. MG32 AJ746089.1: 1.. 814
Mesorhizobium sp. Mezo1 GU479373.1: 1.. 1349
Proteobacteria Alphaproteobacteria Rhizobiales Phyllobacteriaceae Aminobacter Aminobacter aminovorans* Otu0232
Proteobacteria Alphaproteobacteria Rhizobiales Phyllobacteriaceae Phyllobacterium Phyllobacterium myrsinacearum* Otu0265
Phyllobacterium myrsinacearum HM35 AJ011330.1:1..1462
rape rhizosphere bacterium tsb051 AJ295441.1: 1.. 1300
alpha proteobacterium R-36935 FR691417.1: 1.. 1439
Sphingoaurantiacus polygranulatus (T) MC 3718 KP274054.2: 1.. 1411
Proteobacteria Alphaproteobacteria Sphingomonadales Sphingomonadaceae Sphingoaurantiacus Sphingoaurantiacus polygranulatus Otu0527
Altererythrobacter sp. g89 JQ661221.1: 1.. 1360
Proteobacteria Alphaproteobacteria Sphingomonadales Sphingomonadaceae Novosphingobium Novosphingobium hassiacum Otu0235
Altererythrobacter sp. MMH1-3 KC117535.1: 1.. 1403
Novosphingobium hassiacum (T) W-51 AJ416411.1: 1.. 1485
bacterium PE03-6G11 AB127790.1: 1.. 642
Proteobacteria Alphaproteobacteria Sphingomonadales Sphingomonadaceae Novosphingobium Novosphingobium hassiacum Otu2063
Proteobacteria Alphaproteobacteria Sphingomonadales Sphingomonadaceae Novosphingobium Novosphingobium hassiacum* Otu0107
groundwater planktonic bacterium Y2 FJ204471.1: 1.. 1348
Sphingomonas sp. RF-12 GQ205098.1: 1.. 1358
aquatic bacterium R1-A3 AB195721.1: 1.. 799
Sphingomonadaceae bacterium PB205 AB220123.1: 1.. 1373
Proteobacteria Alphaproteobacteria Sphingomonadales Sphingomonadaceae Novosphingobium Novosphingobium hassiacum* Otu0629
Proteobacteria Alphaproteobacteria Sphingomonadales Sphingomonadaceae Novosphingobium Novosphingobium hassiacum* Otu0677
Sphingomonas sp. 44/40 AY571829.1: 1.. 1404
bacterium WM06i A1D HM989884.1: 1.. 1411
Novosphingobium subterraneum (T) IFO 16086 AB025014.1:1..1446
drinking water bacterium OR18 AY328805.1: 1.. 1388
agricultural soil bacterium SI-15 AJ252582.1: 1.. 1001
Altererythrobacter troitsensis (T) KMM 6042 AY676115.1: 1.. 1389
Proteobacteria Alphaproteobacteria Sphingomonadales Sphingomonadaceae Novosphingobium Novosphingobium hassiacum* Otu0248
Proteobacteria Alphaproteobacteria Sphingomonadales Sphingomonadaceae Novosphingobium Novosphingobium hassiacum* Otu0285
Sphingomonas sp. A3 AF361191.1: 1.. 1233
Proteobacteria Alphaproteobacteria Sphingomonadales Sphingomonadaceae Sphingobium Sphingobium amiense Otu0549
Sphingobium amiense (T) YT AB047364.1:8..1448
Sphingobium sp. BiD32 JX879737.1: 1.. 1398
Sphingobium xenophagum DA1 GU969041.1: 1.. 1210
Proteobacteria Alphaproteobacteria Sphingomonadales Sphingomonadaceae Sphingobium Sphingobium xenophagum Otu3934
Proteobacteria Alphaproteobacteria Sphingomonadales Sphingomonadaceae Sphingopyxis Sphingopyxis macrogoltabida Otu0886
Sphingomonas sp. IFO 15915 AB033949.1: 1.. 1416
Sphingopyxis macrogoltabida HXN-200 AJ784806.1: 1.. 727
Sphingomonas sp. 42NP4 AB242807.1: 1.. 878
Sphingopyxis alaskensis RB2256 CP000356.1:complement(2851568..2853056)
Proteobacteria Alphaproteobacteria Sphingomonadales Sphingomonadaceae Sphingopyxis Sphingopyxis alaskensis Otu0292
alpha proteobacterium ASRB9-2 AB299721.1: 1.. 661
Sphingomonas sp. LRR22 JX138997.2: 1.. 1451
Proteobacteria Alphaproteobacteria Sphingomonadales Sphingomonadaceae Sphingomonas Sphingomonas fonticola Otu2812
Croceibacterium mercuriale (T) Coronado KP122961.1: 1.. 1482
Proteobacteria Alphaproteobacteria Sphingomonadales Erythrobacteraceae Croceibacterium Croceibacterium mercuriale Otu3868
Lutibacterium anuloederans LC8 AY026916.1: 1.. 1452
Sphingomonadaceae bacterium PB307 AB220143.1: 1.. 1413
Proteobacteria Alphaproteobacteria Sphingomonadales Sphingomonadaceae Sphingomonas Sphingomonas astaxanthinifaciens Otu2227
Sphingomonadaceae bacterium PB301 AB220139.1: 1.. 1410
Proteobacteria Alphaproteobacteria Sphingomonadales Sphingomonadaceae Sphingomonas Sphingomonas daechungensis Otu2042
Sphingomonas sp. CH15-114 JN628042.1: 1.. 1436
Sphingomonas daechungensis (T) CH15-11 JQ772481.1: 1.. 1436
Proteobacteria Alphaproteobacteria Sphingomonadales Sphingomonadaceae Sphingomonas Sphingomonas sp Otu0378
Sphingomonas kaistensis PB229 AY785128.1: 1.. 1393
Sphingomonadaceae bacterium PB211 AB220126.1: 1.. 1366
Sphingomonas anadarae KMM 3933 16S rRNA AB261013.1: 1.. 1374
Proteobacteria Alphaproteobacteria Sphingomonadales Sphingomonadaceae Sphingomonas Sphingomonas mathurensis* Otu0345
Sphingomonas mathurensis SM13 EF424400.1: 1.. 1378
Sphingomonas sp. WHSC-8 CP010836.1:join(5190637..5191536 1..612)
Sphingomonas sp. WHSC-8 CP010836.1:4632322..4633832
Proteobacteria Alphaproteobacteria Sphingomonadales Sphingomonadaceae Sphingomonas Sphingomonas 1 Otu0414
Sphingomonas koreensis JSS-28 KCTC 2883 AF031240.1:1..1441
Sphingomonas koreensis (T) JSS-26 AF131296.1: 1.. 1441
Proteobacteria Alphaproteobacteria Sphingomonadales Sphingomonadaceae Sphingomonas Sphingomonas koreensis Otu0597
Proteobacteria Alphaproteobacteria Sphingomonadales Sphingomonadaceae Sphingomonas Sphingomonas taxi* Otu1930
Sphingomonas sanxanigenens T12AR21 JF459935.1: 1.. 1365
Sphingomonas taxi ATCC 55669 CP009571.1:complement(1852826..1854327)
bacterium 72814 AB202193.2:1..1445

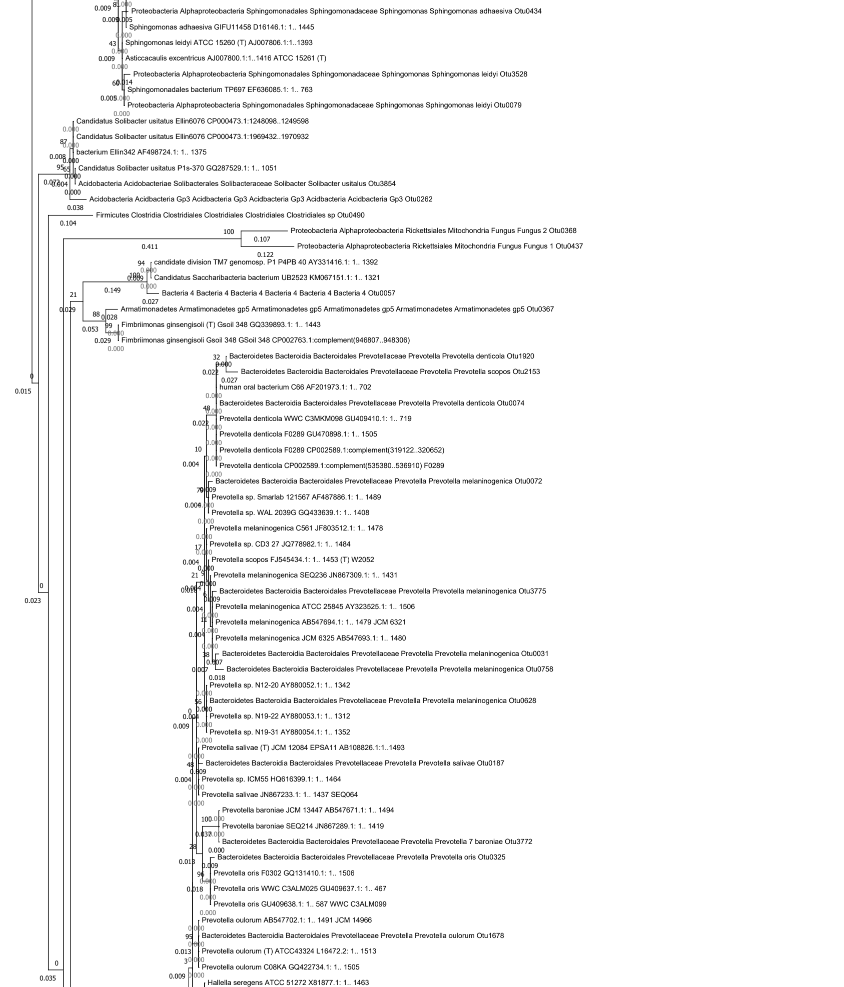

Proteobacteria Alphaproteobacteria Sphingomonadales Sphingomonadaceae Sphingomonas Sphingomonas adhaesiva Otu0434
Sphingomonas adhaesiva GIFU11458 D16146.1: 1.. 1445
Sphingomonas leidyi ATCC 15260 (T) AJ007806.1:1..1393
Asticcacaulis excentricus AJ007800.1:1..1416 ATCC 15261 (T)
Proteobacteria Alphaproteobacteria Sphingomonadales Sphingomonadaceae Sphingomonas Sphingomonas leidyi Otu3528
Sphingomonadales bacterium TP697 EF636085.1: 1.. 763
Proteobacteria Alphaproteobacteria Sphingomonadales Sphingomonadaceae Sphingomonas Sphingomonas leidyi Otu0079
Candidatus Solibacter usitatus Ellin6076 CP000473.1:1248098..1249598
Candidatus Solibacter usitatus Ellin6076 CP000473.1:1969432..1970932
bacterium Ellin342 AF498724.1: 1.. 1375
Candidatus Solibacter usitatus P1s-370 GQ287529.1: 1.. 1051
Acidobacteria Acidobacteriae Solibacterales Solibacteraceae Solibacter Solibacter usitalus Otu3854
Acidobacteria Acidbacteria Gp3 Acidbacteria Gp3 Acidbacteria Gp3 Acidbacteria Acidbacteria Gp3 Otu0262
Firmicutes Clostridia Clostridiales Clostridiales Clostridiales Clostridiales sp Otu0490
Proteobacteria Alphaproteobacteria Rickettsiales Mitochondria Fungus Fungus 2 Otu0368
Proteobacteria Alphaproteobacteria Rickettsiales Mitochondria Fungus Fungus 1 Otu0437
candidate division TM7 genomosp. P1 P4PB 40 AY331416.1: 1.. 1392
Candidatus Saccharibacteria bacterium UB2523 KM067151.1: 1.. 1321
Bacteria 4 Bacteria 4 Bacteria 4 Bacteria 4 Bacteria 4 Bacteria 4 Otu0057
Armatimonadetes Armatimonadetes gp5 Armatimonadetes gp5 Armatimonadetes gp5 Armatimonadetes gp5 Otu0367
Fimbriimonas ginsengisoli (T) Gsoil 348 GQ339893.1: 1.. 1443
Fimbriimonas ginsengisoli Gsoil 348 GSoil 348 CP002763.1:complement(946807..948306)
Bacteroidetes Bacteroidia Bacteroidales Prevotellaceae Prevotella Prevotella denticola Otu1920
Bacteroidetes Bacteroidia Bacteroidales Prevotellaceae Prevotella Prevotella scopos Otu2153
human oral bacterium C66 AF201973.1: 1.. 702
Bacteroidetes Bacteroidia Bacteroidales Prevotellaceae Prevotella Prevotella denticola Otu0074
Prevotella denticola WWC C3MKM098 GU409410.1: 1.. 719
Prevotella denticola F0289 GU470898.1: 1.. 1505
Prevotella denticola F0289 CP002589.1:complement(319122..320652)
Prevotella denticola CP002589.1:complement(535380..536910) F0289
Bacteroidetes Bacteroidia Bacteroidales Prevotellaceae Prevotella Prevotella melaninogenica Otu0072
Prevotella sp. Smarlab 121567 AF487886.1: 1.. 1489
Prevotella sp. WAL 2039G GQ433639.1: 1.. 1408
Prevotella melaninogenica C561 JF803512.1: 1.. 1478
Prevotella sp. CD3 27 JQ778982.1: 1.. 1484
Prevotella scopos FJ545434.1: 1.. 1453 (T) W2052
Prevotella melaninogenica SEQ236 JN867309.1: 1.. 1431
Bacteroidetes Bacteroidia Bacteroidales Prevotellaceae Prevotella Prevotella melaninogenica Otu3775
Prevotella melaninogenica ATCC 25845 AY323525.1: 1.. 1506
Prevotella melaninogenica AB547694.1: 1.. 1479 JCM 6321
Prevotella melaninogenica JCM 6325 AB547693.1: 1.. 1480
Bacteroidetes Bacteroidia Bacteroidales Prevotellaceae Prevotella Prevotella melaninogenica Otu0031
Bacteroidetes Bacteroidia Bacteroidales Prevotellaceae Prevotella Prevotella melaninogenica Otu0758
Prevotella sp. N12-20 AY880052.1: 1.. 1342
Bacteroidetes Bacteroidia Bacteroidales Prevotellaceae Prevotella Prevotella melaninogenica Otu0628
Prevotella sp. N19-22 AY880053.1: 1.. 1312
Prevotella sp. N19-31 AY880054.1: 1.. 1352
Prevotella salivae (T) JCM 12084 EPSA11 AB108826.1:1..1493
Bacteroidetes Bacteroidia Bacteroidales Prevotellaceae Prevotella Prevotella salivae Otu0187
Prevotella sp. ICM55 HQ616399.1: 1.. 1464
Prevotella salivae JN867233.1: 1.. 1437 SEQ064
Prevotella baroniae JCM 13447 AB547671.1: 1.. 1494
Prevotella baroniae SEQ214 JN867289.1: 1.. 1419
Bacteroidetes Bacteroidia Bacteroidales Prevotellaceae Prevotella Prevotella 7 baroniae Otu3772
Bacteroidetes Bacteroidia Bacteroidales Prevotellaceae Prevotella Prevotella oris Otu0325
Prevotella oris F0302 GQ131410.1: 1.. 1506
Prevotella oris WWC C3ALM025 GU409637.1: 1.. 467
Prevotella oris GU409638.1: 1.. 587 WWC C3ALM099
Prevotella oulorum AB547702.1: 1.. 1491 JCM 14966
Bacteroidetes Bacteroidia Bacteroidales Prevotellaceae Prevotella Prevotella oulorum Otu1678
Prevotella oulorum (T) ATCC43324 L16472.2: 1.. 1513
Prevotella oulorum C08KA GQ422734.1: 1.. 1505
Hallella seregens ATCC 51272 X81877.1: 1.. 1463

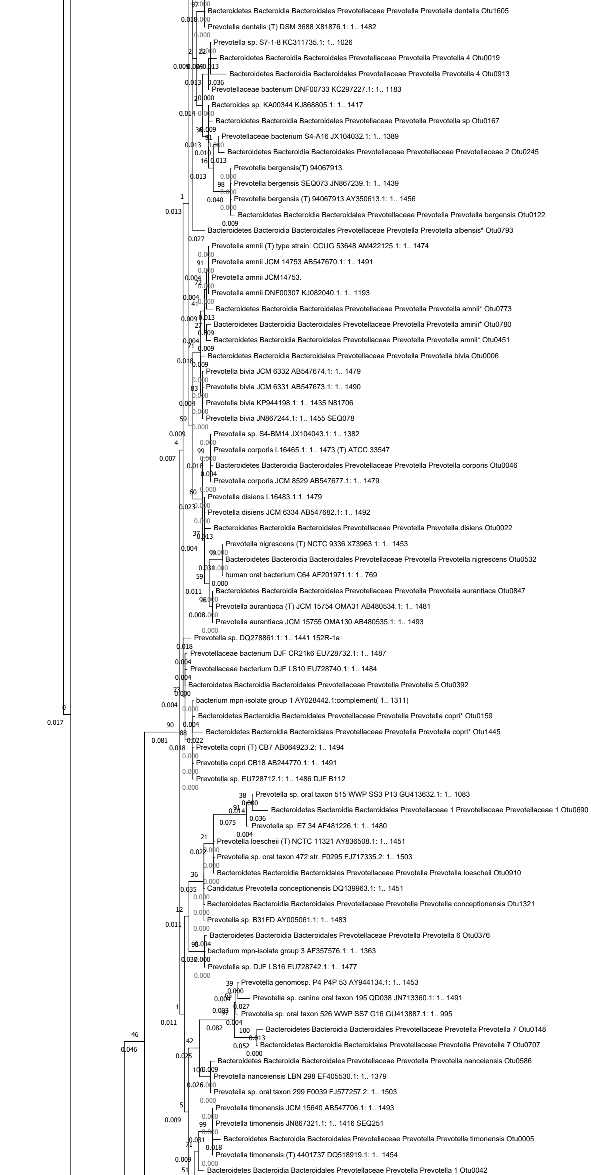

Bacteroidetes Bacteroidia Bacteroidales Prevotellaceae Prevotella Prevotella dentalis Otu1605
Prevotella dentalis (T) DSM 3688 X81876.1: 1.. 1482
Prevotella sp. S7-1-8 KC311735.1: 1.. 1026
Bacteroidetes Bacteroidia Bacteroidales Prevotellaceae Prevotella Prevotella 4 Otu0019
Bacteroidetes Bacteroidia Bacteroidales Prevotellaceae Prevotella Prevotella 4 Otu0913
Prevotellaceae bacterium DNF00733 KC297227.1: 1.. 1183
Bacteroides sp. KA00344 KJ868805.1: 1.. 1417
Bacteroidetes Bacteroidia Bacteroidales Prevotellaceae Prevotella Prevotella sp Otu0167
Prevotellaceae bacterium S4-A16 JX104032.1: 1.. 1389
Bacteroidetes Bacteroidia Bacteroidales Prevotellaceae Prevotellaceae Prevotellaceae 2 Otu0245
Prevotella bergensis(T) 94067913.
Prevotella bergensis SEQ073 JN867239.1: 1.. 1439
Prevotella bergensis (T) 94067913 AY350613.1: 1.. 1456
Bacteroidetes Bacteroidia Bacteroidales Prevotellaceae Prevotella Prevotella bergensis Otu0122
Bacteroidetes Bacteroidia Bacteroidales Prevotellaceae Prevotella Prevotella albensis* Otu0793
Prevotella amnii (T) type strain: CCUG 53648 AM422125.1: 1.. 1474
Prevotella amnii JCM 14753 AB547670.1: 1.. 1491
Prevotella amnii JCM14753.
Prevotella amnii DNF00307 KJ082040.1: 1.. 1193
Bacteroidetes Bacteroidia Bacteroidales Prevotellaceae Prevotella Prevotella amnii* Otu0773
Bacteroidetes Bacteroidia Bacteroidales Prevotellaceae Prevotella Prevotella aminii* Otu0780
Bacteroidetes Bacteroidia Bacteroidales Prevotellaceae Prevotella Prevotella amnii* Otu0451
Bacteroidetes Bacteroidia Bacteroidales Prevotellaceae Prevotella Prevotella bivia Otu0006
Prevotella bivia JCM 6332 AB547674.1: 1.. 1479
Prevotella bivia JCM 6331 AB547673.1: 1.. 1490
Prevotella bivia KP944198.1: 1.. 1435 N81706
Prevotella bivia JN867244.1: 1.. 1455 SEQ078
Prevotella sp. S4-BM14 JX104043.1: 1.. 1382
Prevotella corporis L16465.1: 1.. 1473 (T) ATCC 33547
Bacteroidetes Bacteroidia Bacteroidales Prevotellaceae Prevotella Prevotella corporis Otu0046
Prevotella corporis JCM 8529 AB547677.1: 1.. 1479
Prevotella disiens L16483.1:1..1479
Prevotella disiens JCM 6334 AB547682.1: 1.. 1492
Bacteroidetes Bacteroidia Bacteroidales Prevotellaceae Prevotella Prevotella disiens Otu0022
Prevotella nigrescens (T) NCTC 9336 X73963.1: 1.. 1453
Bacteroidetes Bacteroidia Bacteroidales Prevotellaceae Prevotella Prevotella nigrescens Otu0532
human oral bacterium C64 AF201971.1: 1.. 769
Bacteroidetes Bacteroidia Bacteroidales Prevotellaceae Prevotella Prevotella aurantiaca Otu0847
Prevotella aurantiaca (T) JCM 15754 OMA31 AB480534.1: 1.. 1481
Prevotella aurantiaca JCM 15755 OMA130 AB480535.1: 1.. 1493
Prevotella sp. DQ278861.1: 1.. 1441 152R-1a
Prevotellaceae bacterium DJF CR21k6 EU728732.1: 1.. 1487
Prevotellaceae bacterium DJF LS10 EU728740.1: 1.. 1484
Bacteroidetes Bacteroidia Bacteroidales Prevotellaceae Prevotella Prevotella 5 Otu0392
bacterium mpn-isolate group 1 AY028442.1:complement(1.. 1311)
Bacteroidetes Bacteroidia Bacteroidales Prevotellaceae Prevotella Prevotella copri* Otu0159
Bacteroidetes Bacteroidia Bacteroidales Prevotellaceae Prevotella Prevotella copri* Otu1445
Prevotella copri (T) CB7 AB064923.2: 1.. 1494
Prevotella copri CB18 AB244770.1: 1.. 1491
Prevotella sp. EU728712.1: 1.. 1486 DJF B112
Prevotella sp. oral taxon 515 WWP SS3 P13 GU413632.1: 1.. 1083
Bacteroidetes Bacteroidia Bacteroidales Prevotellaceae 1 Prevotellaceae Prevotellaceae 1 Otu0690
Prevotella sp. E7 34 AF481226.1: 1.. 1480
Prevotella loescheii (T) NCTC 11321 AY836508.1: 1.. 1451
Prevotella sp. oral taxon 472 str. F0295 FJ717335.2: 1.. 1503
Bacteroidetes Bacteroidia Bacteroidales Prevotellaceae Prevotella Prevotella loescheii Otu0910
Candidatus Prevotella conceptionensis DQ139963.1: 1.. 1451
Bacteroidetes Bacteroidia Bacteroidales Prevotellaceae Prevotella Prevotella conceptionensis Otu1321
Prevotella sp. B31FD AY005061.1: 1.. 1483
Bacteroidetes Bacteroidia Bacteroidales Prevotellaceae Prevotella Prevotella 6 Otu0376
bacterium mpn-isolate group 3 AF357576.1: 1.. 1363
Prevotella sp. DJF LS16 EU728742.1: 1.. 1477
Prevotella genomosp. P4 P4P 53 AY944134.1: 1.. 1453
Prevotella sp. canine oral taxon 195 QD038 JN713360.1: 1.. 1491
Prevotella sp. oral taxon 526 WWP SS7 G16 GU413887.1: 1.. 995
Bacteroidetes Bacteroidia Bacteroidales Prevotellaceae Prevotella Prevotella 7 Otu0148
Bacteroidetes Bacteroidia Bacteroidales Prevotellaceae Prevotella Prevotella 7 Otu0707
Bacteroidetes Bacteroidia Bacteroidales Prevotellaceae Prevotella Prevotella nanceiensis Otu0586
Prevotella nanceiensis LBN 298 EF405530.1: 1.. 1379
Prevotella sp. oral taxon 299 F0039 FJ577257.2: 1.. 1503
Prevotella timonensis JCM 15640 AB547706.1: 1.. 1493
Prevotella timonensis JN867321.1: 1.. 1416 SEQ251
Bacteroidetes Bacteroidia Bacteroidales Prevotellaceae Prevotella Prevotella timonensis Otu0005
Prevotella timonensis (T) 4401737 DQ518919.1: 1.. 1454
Bacteroidetes Bacteroidia Bacteroidales Prevotellaceae Prevotella Prevotella 1 Otu0042

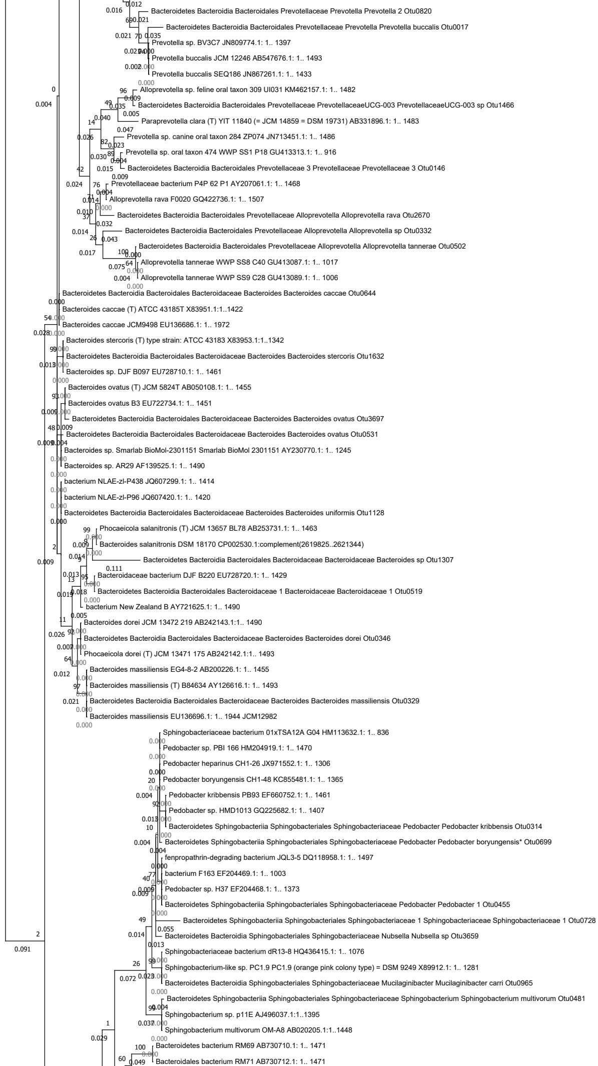

Bacteroidetes Bacteroidia Bacteroidales Prevotellaceae Prevotella Prevotella 2 Otu0820
Bacteroidetes Bacteroidia Bacteroidales Prevotellaceae Prevotella Prevotella buccalis Otu0017
Prevotella sp. BV3C7 JN809774.1: 1.. 1397
Prevotella buccalis JCM 12246 AB547676.1: 1.. 1493
Prevotella buccalis SEQ186 JN867261.1: 1.. 1433
Alloprevotella sp. feline oral taxon 309 UI031 KM462157.1: 1.. 1482
Bacteroidetes Bacteroidia Bacteroidales Prevotellaceae PrevotellaceaeUCG-003 PrevotellaceaeUCG-003 sp Otu1466
Paraprevotella clara (T) YIT 11840 (= JCM 14859 = DSM 19731) AB331896.1: 1.. 1483
Prevotella sp. canine oral taxon 284 ZP074 JN713451.1: 1.. 1486
Prevotella sp. oral taxon 474 WWP SS1 P18 GU413313.1: 1.. 916
Bacteroidetes Bacteroidia Bacteroidales Prevotellaceae 3 Prevotellaceae Prevotellaceae 3 Otu0146
Prevotellaceae bacterium P4P 62 P1 AY207061.1: 1.. 1468
Alloprevotella rava F0020 GQ422736.1: 1.. 1507
Bacteroidetes Bacteroidia Bacteroidales Prevotellaceae Alloprevotella Alloprevotella rava Otu2670
Bacteroidetes Bacteroidia Bacteroidales Prevotellaceae Alloprevotella Alloprevotella sp Otu0332
Bacteroidetes Bacteroidia Bacteroidales Prevotellaceae Alloprevotella Alloprevotella tannerae Otu0502
Alloprevotella tannerae WWP SS8 C40 GU413087.1: 1.. 1017
Alloprevotella tannerae WWP SS9 C28 GU413089.1: 1.. 1006
Bacteroidetes Bacteroidia Bacteroidales Bacteroidaceae Bacteroides Bacteroides caccae Otu0644
Bacteroides caccae (T) ATCC 43185T X83951.1:1..1422
Bacteroides caccae JCM9498 EU136686.1: 1.. 1972
Bacteroides stercoris (T) type strain: ATCC 43183 X83953.1:1..1342
Bacteroidetes Bacteroidia Bacteroidales Bacteroidaceae Bacteroides Bacteroides stercoris Otu1632
Bacteroides sp. DJF B097 EU728710.1: 1.. 1461
Bacteroides ovatus (T) JCM 5824T AB050108.1: 1.. 1455
Bacteroides ovatus B3 EU722734.1: 1.. 1451
Bacteroidetes Bacteroidia Bacteroidales Bacteroidaceae Bacteroides Bacteroides ovatus Otu3697
Bacteroidetes Bacteroidia Bacteroidales Bacteroidaceae Bacteroides Bacteroides ovatus Otu0531
Bacteroides sp. Smarlab BioMol-2301151 Smarlab BioMol 2301151 AY230770.1: 1.. 1245
Bacteroides sp. AR29 AF139525.1: 1.. 1490
bacterium NLAE-zl-P438 JQ607299.1: 1.. 1414
bacterium NLAE-zl-P96 JQ607420.1: 1.. 1420
Bacteroidetes Bacteroidia Bacteroidales Bacteroidaceae Bacteroides Bacteroides uniformis Otu1128
Phocaeicola salanitronis (T) JCM 13657 BL78 AB253731.1: 1.. 1463
Bacteroides salanitronis DSM 18170 CP002530.1:complement(2619825..2621344)
Bacteroidetes Bacteroidia Bacteroidales Bacteroidaceae Bacteroidaceae Bacteroides sp Otu1307
Bacteroidaceae bacterium DJF B220 EU728720.1: 1.. 1429
Bacteroidetes Bacteroidia Bacteroidales Bacteroidaceae 1 Bacteroidaceae Bacteroidaceae 1 Otu0519
bacterium New Zealand B AY721625.1: 1.. 1490
Bacteroides dorei JCM 13472 219 AB242143.1:1.. 1490
Bacteroidetes Bacteroidia Bacteroidales Bacteroidaceae Bacteroides Bacteroides dorei Otu0346
Phocaeicola dorei (T) JCM 13471 175 AB242142.1:1.. 1493
Bacteroides massiliensis EG4-8-2 AB200226.1: 1.. 1455
Bacteroides massiliensis (T) B84634 AY126616.1: 1.. 1493
Bacteroidetes Bacteroidia Bacteroidales Bacteroidaceae Bacteroides Bacteroides massiliensis Otu0329
Bacteroides massiliensis EU136696.1: 1.. 1944 JCM12982
Sphingobacteriaceae bacterium 01xTSA12A G04 HM113632.1: 1.. 836
Pedobacter sp. PBI 166 HM204919.1: 1.. 1470
Pedobacter heparinus CH1-26 JX971552.1: 1.. 1306
Pedobacter boryungensis CH1-48 KC855481.1: 1.. 1365
Pedobacter kribbensis PB93 EF660752.1: 1.. 1461
Pedobacter sp. HMD1013 GQ225682.1: 1.. 1407
Bacteroidetes Sphingobacteriia Sphingobacteriales Sphingobacteriaceae Pedobacter Pedobacter kribbensis Otu0314
Bacteroidetes Sphingobacteriia Sphingobacteriales Sphingobacteriaceae Pedobacter Pedobacter boryungensis* Otu0699
fenpropathrin-degrading bacterium JQL3-5 DQ118958.1: 1.. 1497
bacterium F163 EF204469.1: 1.. 1003
Pedobacter sp. H37 EF204468.1: 1.. 1373
Bacteroidetes Sphingobacteriia Sphingobacteriales Sphingobacteriaceae Pedobacter Pedobacter 1 Otu0455
Bacteroidetes Sphingobacteriia Sphingobacteriales Sphingobacteriaceae 1 Sphingobacteriaceae Sphingobacteriaceae 1 Otu0728
Bacteroidetes Bacteroidia Sphingobacteriales Sphingobacteriaceae Nubsella Nubsella sp Otu3659
Sphingobacteriaceae bacterium dR13-8 HQ436415.1: 1.. 1076
Sphingobacterium-like sp. PC1.9 PC1.9 (orange pink colony type) = DSM 9249 X89912.1: 1.. 1281
Bacteroidetes Bacteroidia Sphingobacteriales Sphingobacteriaceae Mucilaginibacter Mucilaginibacter carri Otu0965
Bacteroidetes Sphingobacteriia Sphingobacteriales Sphingobacteriaceae Sphingobacterium Sphingobacterium multivorum Otu0481
Sphingobacterium sp. p11E AJ496037.1:1..1395
Sphingobacterium multivorum OM-A8 AB020205.1:1..1448
Bacteroidetes bacterium RM69 AB730710.1: 1.. 1471
Bacteroidales bacterium RM71 AB730712.1: 1.. 1471

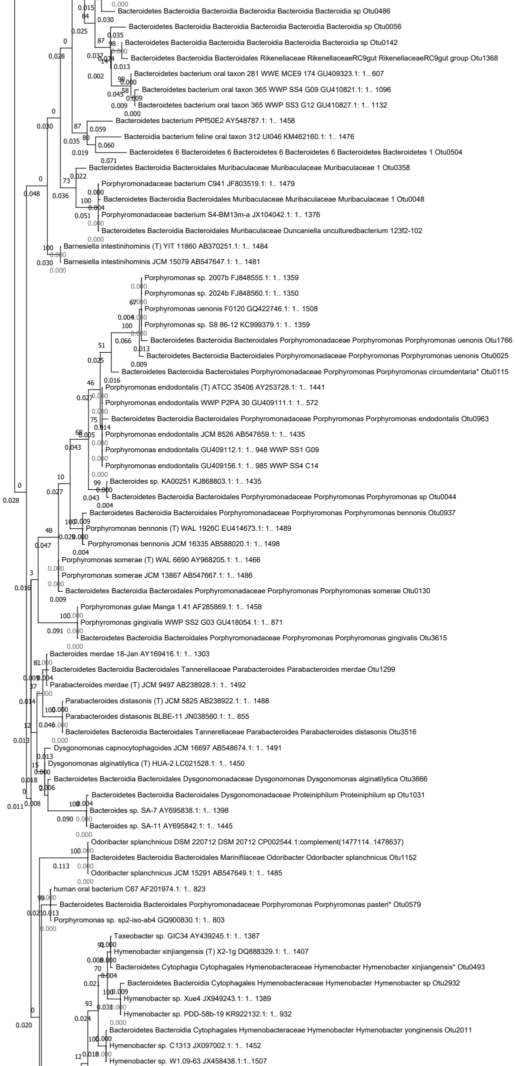

Bacteroidetes Bacteroidia Bacteroidia Bacteroidia Bacteroidia Bacteroidia sp Otu0486
Bacteroidetes Bacteroidia Bacteroidia Bacteroidia Bacteroidia Bacteroidia sp Otu0056
Bacteroidetes Bacteroidia Bacteroidia Bacteroidia Bacteroidia Bacteroidia sp Otu0142
Bacteroidetes Bacteroidia Bacteroidales Rikenellaceae RikenellaceaeRC9gut RikenellaceaeRC9gut group Otu1368
Bacteroidetes bacterium oral taxon 281 WWE MCE9 174 GU409323.1: 1.. 607
Bacteroidetes bacterium oral taxon 365 WWP SS4 G09 GU410821.1: 1.. 1096
Bacteroidetes bacterium oral taxon 365 WWP SS3 G12 GU410827.1: 1.. 1132
Bacteroidetes bacterium PPf50E2 AY548787.1: 1.. 1458
Bacteroidia bacterium feline oral taxon 312 UI046 KM462160.1: 1.. 1476
Bacteroidetes 6 Bacteroidetes 6 Bacteroidetes 6 Bacteroidetes 6 Bacteroidetes Bacteroidetes 1 Otu0504
Bacteroidetes Bacteroidia Bacteroidales Muribaculaceae Muribaculaceae Muribaculaceae 1 Otu0358
Porphyromonadaceae bacterium C941 JF803519.1: 1.. 1479
Bacteroidetes Bacteroidia Bacteroidales Muribaculaceae Muribaculaceae Muribaculaceae 1 Otu0048
Porphyromonadaceae bacterium S4-BM13m-a JX104042.1: 1.. 1376
Bacteroidetes Bacteroidia Bacteroidales Muribaculaceae Duncaniella unculturedbacterium 123f2-102
Barnesiella intestinihominis (T) YIT 11860 AB370251.1: 1.. 1484
Barnesiella intestinihominis JCM 15079 AB547647.1: 1.. 1481
Porphyromonas sp. 2007b FJ848555.1: 1.. 1359
Porphyromonas sp. 2024b FJ848560.1: 1.. 1350
Porphyromonas uenonis F0120 GQ422746.1: 1.. 1508
Porphyromonas sp. S8 86-12 KC999379.1: 1.. 1359
Bacteroidetes Bacteroidia Bacteroidales Porphyromonadaceae Porphyromonas Porphyromonas uenonis Otu1766
Bacteroidetes Bacteroidia Bacteroidales Porphyromonadaceae Porphyromonas Porphyromonas uenonis Otu0025
Bacteroidetes Bacteroidia Bacteroidales Porphyromonadaceae Porphyromonas Porphyromonas circumdentaria* Otu0115
Porphyromonas endodontalis (T) ATCC 35406 AY253728.1: 1.. 1441
Porphyromonas endodontalis WWP P2PA 30 GU409111.1: 1.. 572
Bacteroidetes Bacteroidia Bacteroidales Porphyromonadaceae Porphyromonas Porphyromonas endodontalis Otu0963
Porphyromonas endodontalis JCM 8526 AB547659.1: 1.. 1435
Porphyromonas endodontalis GU409112.1: 1.. 948 WWP SS1 G09
Porphyromonas endodontalis GU409156.1: 1.. 985 WWP SS4 C14
Bacteroides sp. KA00251 KJ868803.1: 1.. 1435
Bacteroidetes Bacteroidia Bacteroidales Porphyromonadaceae Porphyromonas Porphyromonas sp Otu0044
Bacteroidetes Bacteroidia Bacteroidales Porphyromonadaceae Porphyromonas Porphyromonas bennonis Otu0937
Porphyromonas bennonis (T) WAL 1926C EU414673.1: 1.. 1489
Porphyromonas bennonis JCM 16335 AB588020.1: 1.. 1498
Porphyromonas somerae (T) WAL 6690 AY968205.1: 1.. 1466
Porphyromonas somerae JCM 13867 AB547667.1: 1.. 1486
Bacteroidetes Bacteroidia Bacteroidales Porphyromonadaceae Porphyromonas Porphyromonas somerae Otu0130
Porphyromonas gulae Manga 1.41 AF285869.1: 1.. 1458
Porphyromonas gingivalis WWP SS2 G03 GU418054.1: 1.. 871
Bacteroidetes Bacteroidia Bacteroidales Porphyromonadaceae Porphyromonas Porphyromonas gingivalis Otu3615
Bacteroides merdae 18-Jan AY169416.1: 1.. 1303
Bacteroidetes Bacteroidia Bacteroidales Tannerellaceae Parabacteroides Parabacteroides merdae Otu1299
Parabacteroides merdae (T) JCM 9497 AB238928.1: 1.. 1492
Parabacteroides distasonis (T) JCM 5825 AB238922.1: 1.. 1488
Parabacteroides distasonis BLBE-11 JN038560.1: 1.. 855
Bacteroidetes Bacteroidia Bacteroidales Tannerellaceae Parabacteroides Parabacteroides distasonis Otu3516
Dysgonomonas capnocytophagoides JCM 16697 AB548674.1: 1.. 1491
Dysgonomonas alginatilytica (T) HUA-2 LC021528.1: 1.. 1450
Bacteroidetes Bacteroidia Bacteroidales Dysgonomonadaceae Dysgonomonas Dysgonomonas alginatilytica Otu3666
Bacteroidetes Bacteroidia Bacteroidales Dysgonomonadaceae Proteiniphilum Proteiniphilum sp Otu1031
Bacteroides sp. SA-7 AY695838.1: 1.. 1398
Bacteroides sp. SA-11 AY695842.1: 1.. 1445
Odoribacter splanchnicus DSM 220712 DSM 20712 CP002544.1:complement(1477114..1478637)
Bacteroidetes Bacteroidia Bacteroidales Marinifilaceae Odoribacter Odoribacter splanchnicus Otu1152
Odoribacter splanchnicus JCM 15291 AB547649.1: 1.. 1485
human oral bacterium C67 AF201974.1: 1.. 823
Bacteroidetes Bacteroidia Bacteroidales Porphyromonadaceae Porphyromonas Porphyromonas pasteri* Otu0579
Porphyromonas sp. sp2-iso-ab4 GQ900830.1: 1.. 803
Taxeobacter sp. GIC34 AY439245.1: 1.. 1387
Hymenobacter xinjiangensis (T) X2-1g DQ888329.1: 1.. 1407
Bacteroidetes Cytophagia Cytophagales Hymenobacteraceae Hymenobacter Hymenobacter xinjiangensis* Otu0493
Bacteroidetes Bacteroidia Cytophagales Hymenobacteraceae Hymenobacter Hymenobacter sp Otu2932
Hymenobacter sp. Xue4 JX949243.1: 1.. 1389
Hymenobacter sp. PDD-58b-19 KR922132.1: 1.. 932
Bacteroidetes Bacteroidia Cytophagales Hymenobacteraceae Hymenobacter Hymenobacter yonginensis Otu2011
Hymenobacter sp. C1313 JX097002.1: 1.. 1452
Hymenobacter sp. W1.09-63 JX458438.1:1..1507

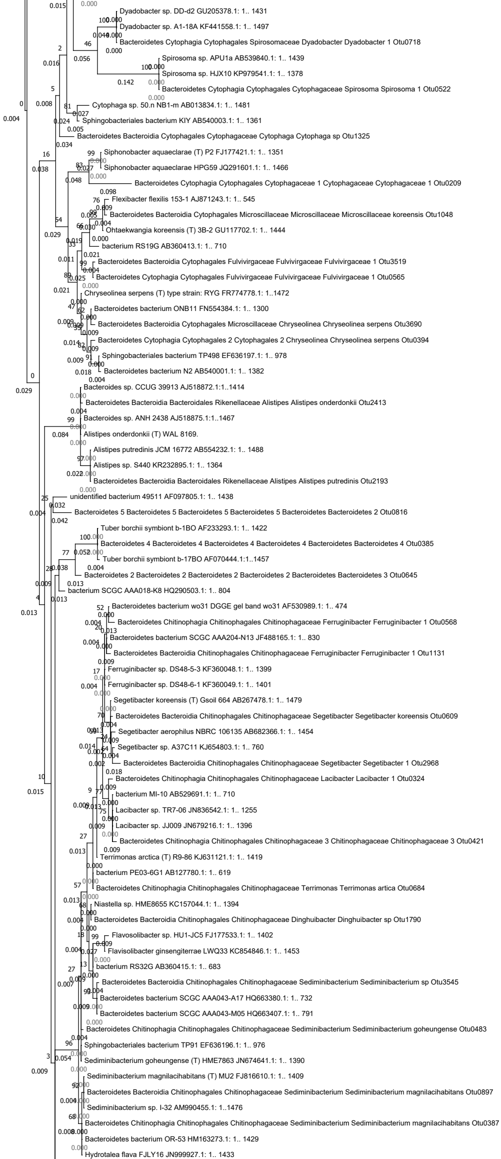

Dyadobacter sp. DD-d2 GU205378.1: 1.. 1431
Dyadobacter sp. A1-18A KF441558.1: 1.. 1497
Bacteroidetes Cytophagia Cytophagales Spirosomaceae Dyadobacter Dyadobacter 1 Otu0718
Spirosoma sp. APU1a AB539840.1: 1.. 1439
Spirosoma sp. HJX10 KP979541.1: 1.. 1378
Bacteroidetes Cytophagia Cytophagales Cytophagaceae Spirosoma Spirosoma 1 Otu0522
Cytophaga sp. 50.n NB1-m AB013834.1: 1.. 1481
Sphingobacteriales bacterium KIY AB540003.1: 1.. 1361
Bacteroidetes Bacteroidia Cytophagales Cytophagaceae Cytophaga Cytophaga sp Otu1325
Siphonobacter aquaeclarae (T) P2 FJ177421.1: 1.. 1351
Siphonobacter aquaeclarae HPG59 JQ291601.1: 1.. 1466
Bacteroidetes Cytophagia Cytophagales Cytophagaceae 1 Cytophagaceae Cytophagaceae 1 Otu0209
Flexibacter flexilis 153-1 AJ871243.1: 1.. 545
Bacteroidetes Bacteroidia Cytophagales Microscillaceae Microscillaceae Microscillaceae koreensis Otu1048
Ohtaekwangia koreensis (T) 3B-2 GU117702.1: 1.. 1444
bacterium RS19G AB360413.1: 1.. 710
Bacteroidetes Bacteroidia Cytophagales Fulvivirgaceae Fulvivirgaceae Fulvivirgaceae 1 Otu3519
Bacteroidetes Cytophagia Cytophagales Fulvivirgaceae Fulvivirgaceae Fulvivirgaceae 1 Otu0565
Chryseolinea serpens (T) type strain: RYG FR774778.1: 1..1472
Bacteroidetes bacterium ONB11 FN554384.1: 1.. 1300
Bacteroidetes Bacteroidia Cytophagales Microscillaceae Chryseolinea Chryseolinea serpens Otu3690
Bacteroidetes Cytophagia Cytophagales 2 Cytophagales 2 Chryseolinea Chryseolinea serpens Otu0394
Sphingobacteriales bacterium TP498 EF636197.1: 1.. 978
Bacteroidetes bacterium N2 AB540001.1: 1.. 1382
Bacteroides sp. CCUG 39913 AJ518872.1:1..1414
Bacteroidetes Bacteroidia Bacteroidales Rikenellaceae Alistipes Alistipes onderdonkii Otu2413
Bacteroides sp. ANH 2438 AJ518875.1:1..1467
Alistipes onderdonkii (T) WAL 8169.
Alistipes putredinis JCM 16772 AB554232.1: 1.. 1488
Alistipes sp. S440 KR232895.1: 1.. 1364
Bacteroidetes Bacteroidia Bacteroidales Rikenellaceae Alistipes Alistipes putredinis Otu2193
unidentified bacterium 49511 AF097805.1: 1.. 1438
Bacteroidetes 5 Bacteroidetes 5 Bacteroidetes 5 Bacteroidetes 5 Bacteroidetes Bacteroidetes 2 Otu0816
Tuber borchii symbiont b-1BO AF233293.1: 1.. 1422
Bacteroidetes 4 Bacteroidetes 4 Bacteroidetes 4 Bacteroidetes 4 Bacteroidetes Bacteroidetes 4 Otu0385
Tuber borchii symbiont b-17BO AF070444.1:1..1457
Bacteroidetes 2 Bacteroidetes 2 Bacteroidetes 2 Bacteroidetes 2 Bacteroidetes Bacteroidetes 3 Otu0645
bacterium SCGC AAA018-K8 HQ290503.1: 1.. 804
Bacteroidetes bacterium wo31 DGGE gel band wo31 AF530989.1: 1.. 474
Bacteroidetes Chitinophagia Chitinophagales Chitinophagaceae Ferruginibacter Ferruginibacter 1 Otu0568
Bacteroidetes bacterium SCGC AAA204-N13 JF488165.1: 1.. 830
Bacteroidetes Bacteroidia Chitinophagales Chitinophagaceae Ferruginibacter Ferruginibacter 1 Otu1131
Ferruginibacter sp. DS48-5-3 KF360048.1: 1.. 1399
Ferruginibacter sp. DS48-6-1 KF360049.1: 1.. 1401
Segetibacter koreensis (T) Gsoil 664 AB267478.1: 1.. 1479
Bacteroidetes Bacteroidia Chitinophagales Chitinophagaceae Segetibacter Segetibacter koreensis Otu0609
Segetibacter aerophilus NBRC 106135 AB682366.1: 1.. 1454
Segetibacter sp. A37C11 KJ654803.1: 1.. 760
Bacteroidetes Bacteroidia Chitinophagales Chitinophagaceae Segetibacter Segetibacter 1 Otu2968
Bacteroidetes Chitinophagia Chitinophagales Chitinophagaceae Lacibacter Lacibacter 1 Otu0324
bacterium MI-10 AB529691.1: 1.. 710
Lacibacter sp. TR7-06 JN836542.1: 1.. 1255
Lacibacter sp. JJ009 JN679216.1: 1.. 1396
Bacteroidetes Chitinophagia Chitinophagales Chitinophagaceae 3 Chitinophagaceae Chitinophagaceae 3 Otu0421
Terrimonas arctica (T) R9-86 KJ631121.1: 1.. 1419
bacterium PE03-6G1 AB127780.1: 1.. 619
Bacteroidetes Chitinophagia Chitinophagales Chitinophagaceae Terrimonas Terrimonas artica Otu0684
Niastella sp. HME8655 KC157044.1: 1.. 1394
Bacteroidetes Bacteroidia Chitinophagales Chitinophagaceae Dinghuibacter Dinghuibacter sp Otu1790
Flavosolibacter sp. HU1-JC5 FJ177533.1: 1.. 1402
Flavisolibacter ginsengiterrae LWQ33 KC854846.1: 1.. 1453
bacterium RS32G AB360415.1: 1.. 683
Bacteroidetes Bacteroidia Chitinophagales Chitinophagaceae Sediminibacterium Sediminibacterium sp Otu3545
Bacteroidetes bacterium SCGC AAA043-A17 HQ663380.1: 1.. 732
Bacteroidetes bacterium SCGC AAA043-M05 HQ663407.1: 1.. 791
Bacteroidetes Chitinophagia Chitinophagales Chitinophagaceae Sediminibacterium Sediminibacterium goheungense Otu0483
Sphingobacteriales bacterium TP91 EF636196.1: 1.. 976
Sediminibacterium goheungense (T) HME7863 JN674641.1: 1.. 1390
Sediminibacterium magnilacihabitans (T) MU2 FJ816610.1: 1.. 1409
Bacteroidetes Bacteroidia Chitinophagales Chitinophagaceae Sediminibacterium Sediminibacterium magnilacihabitans Otu0897
Sediminibacterium sp. I-32 AM990455.1: 1..1476
Bacteroidetes Chitinophagia Chitinophagales Chitinophagaceae Sediminibacterium Sediminibacterium magnilacihabitans Otu0387
Bacteroidetes bacterium OR-53 HM163273.1: 1.. 1429
Hydrotalea flava FJLY16 JN999927.1: 1.. 1433

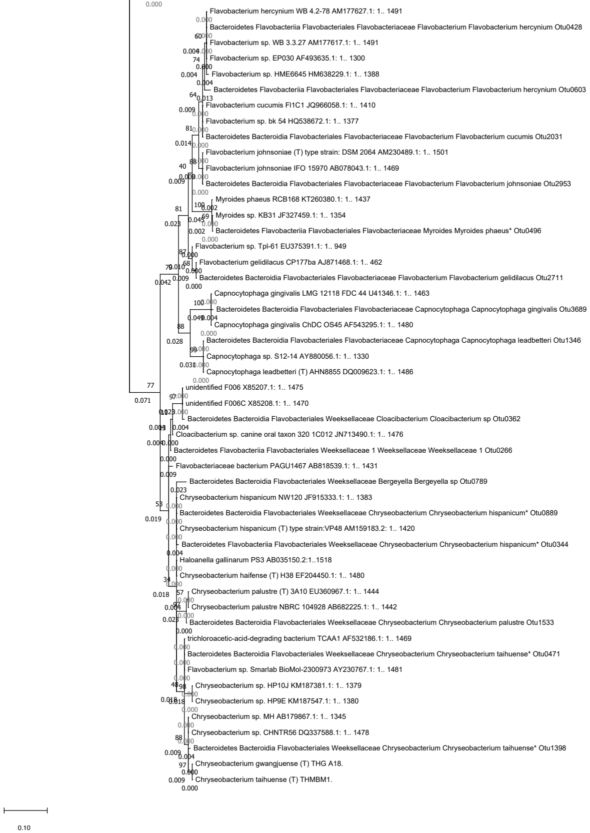
Flavobacterium hercynium WB 4.2-78 AM177627.1: 1.. 1491
Bacteroidetes Flavobacteriia Flavobacteriales Flavobacteriaceae Flavobacterium Flavobacterium hercynium Otu0428
Flavobacterium sp. WB 3.3.27 AM177617.1: 1.. 1491
Flavobacterium sp. EP030 AF493635.1: 1.. 1300
Flavobacterium sp. HME6645 HM638229.1: 1.. 1388
Bacteroidetes Flavobacteriia Flavobacteriales Flavobacteriaceae Flavobacterium Flavobacterium hercynium Otu0603
Flavobacterium cucumis Fl1C1 JQ966058.1: 1.. 1410
Flavobacterium sp. bk 54 HQ538672.1: 1.. 1377
Bacteroidetes Bacteroidia Flavobacteriales Flavobacteriaceae Flavobacterium Flavobacterium cucumis Otu2031
Flavobacterium johnsoniae (T) type strain: DSM 2064 AM230489.1: 1.. 1501
Flavobacterium johnsoniae IFO 15970 AB078043.1: 1.. 1469
Bacteroidetes Bacteroidia Flavobacteriales Flavobacteriaceae Flavobacterium Flavobacterium johnsoniae Otu2953
Myroides phaeus RCB168 KT260380.1: 1.. 1437
Myroides sp. KB31 JF327459.1: 1.. 1354
Bacteroidetes Flavobacteriia Flavobacteriales Flavobacteriaceae Myroides Myroides phaeus* Otu0496
Flavobacterium sp. Tpl-61 EU375391.1: 1.. 949
Flavobacterium gelidilacus CP177ba AJ871468.1: 1.. 462
Bacteroidetes Bacteroidia Flavobacteriales Flavobacteriaceae Flavobacterium Flavobacterium gelidilacus Otu2711
Capnocytophaga gingivalis LMG 12118 FDC 44 U41346.1: 1.. 1463
Bacteroidetes Bacteroidia Flavobacteriales Flavobacteriaceae Capnocytophaga Capnocytophaga gingivalis Otu3689
Capnocytophaga gingivalis ChDC OS45 AF543295.1: 1.. 1480
Bacteroidetes Bacteroidia Flavobacteriales Flavobacteriaceae Capnocytophaga Capnocytophaga leadbetteri Otu1346
Capnocytophaga sp. S12-14 AY880056.1: 1.. 1330
Capnocytophaga leadbetteri (T) AHN8855 DQ009623.1: 1.. 1486
unidentified F006 X85207.1: 1.. 1475
unidentified F006C X85208.1: 1.. 1470
Bacteroidetes Bacteroidia Flavobacteriales Weeksellaceae Cloacibacterium Cloacibacterium sp Otu0362
Cloacibacterium sp. canine oral taxon 320 1C012 JN713490.1: 1.. 1476
Bacteroidetes Flavobacteriia Flavobacteriales Weeksellaceae 1 Weeksellaceae Weeksellaceae 1 Otu0266
Flavobacteriaceae bacterium PAGU1467 AB818539.1: 1.. 1431
Bacteroidetes Bacteroidia Flavobacteriales Weeksellaceae Bergeyella Bergeyella sp Otu0789
Chryseobacterium hispanicum NW120 JF915333.1: 1.. 1383
Bacteroidetes Bacteroidia Flavobacteriales Weeksellaceae Chryseobacterium Chryseobacterium hispanicum* Otu0889
Chryseobacterium hispanicum (T) type strain:VP48 AM159183.2: 1.. 1420
Bacteroidetes Flavobacteriia Flavobacteriales Weeksellaceae Chryseobacterium Chryseobacterium hispanicum* Otu0344
Haloanella gallinarum PS3 AB035150.2:1..1518
Chryseobacterium haifense (T) H38 EF204450.1: 1.. 1480
Chryseobacterium palustre (T) 3A10 EU360967.1: 1.. 1444
Chryseobacterium palustre NBRC 104928 AB682225.1: 1.. 1442
Bacteroidetes Bacteroidia Flavobacteriales Weeksellaceae Chryseobacterium Chryseobacterium palustre Otu1533
trichloroacetic-acid-degrading bacterium TCAA1 AF532186.1: 1.. 1469
Bacteroidetes Bacteroidia Flavobacteriales Weeksellaceae Chryseobacterium Chryseobacterium taihuense* Otu0471
Flavobacterium sp. Smarlab BioMol-2300973 AY230767.1: 1.. 1481
Chryseobacterium sp. HP10J KM187381.1: 1.. 1379
Chryseobacterium sp. HP9E KM187547.1: 1.. 1380
Chryseobacterium sp. MH AB179867.1: 1.. 1345
Chryseobacterium sp. CHNTR56 DQ337588.1: 1.. 1478
Bacteroidetes Bacteroidia Flavobacteriales Weeksellaceae Chryseobacterium Chryseobacterium taihuense* Otu1398
Chryseobacterium gwangjuense (T) THG A18.
Chryseobacterium taihuense (T) THMBM1.
0.10

Figure 2b. Phylogenetic tree of all retained OTUs from MicrobiomeAnalyst

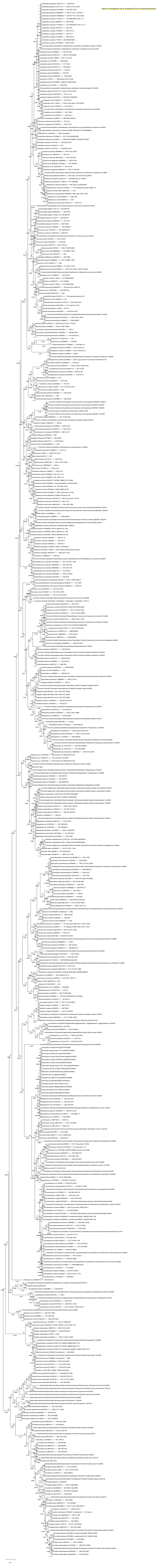

Supplement: S2 Fig — (PDF) [file pone.0272012.s002.pdf]
